# Supplementary material for: Teaching Trauma-Informed Care: A Symposium for Medical Students
Source: MedEdPORTAL. 2020 Dec 30;16:11061. doi: 10.15766/mep_2374-8265.11061 (PMC7780743; doi:10.15766/mep_2374-8265.11061)
Supplement: Supplementary file 1 — TIC-S PowerPoint.pptxStress Health Self-Care Tool.pdfFacilitator Guide.docxEvaluation.docxFacilitator Prep Slides.pptx [file mep_2374-8265.11061-s001.zip › _Educational_Summary_Report_11061.pdf]

MedEdPORTAL® The AAMC Journal of Teaching and Learning Resources

Original Publication Open Access

Teaching Trauma-Informed Care: A Symposium for Medical Students

Binny Chokshi, MD*, Kimberly Walsh, MPA, RN, Danielle Dooley, MD, MPhil, Olanrewaju Falusi, MD, Lawrence Deyton, MD, MSPH, Lee Beers, MD

*Corresponding author: bchokshi@cnmc.org

Abstract

Introduction: A large body of evidence links exposure to childhood trauma with negative health outcomes. Training future physicians to recognize and respond to trauma is paramount, and engaging medical students in the preclinical years affords the opportunity to foster the development of a trauma-informed lens that can then be solidified during clinical clerkships. **Methods:** We developed and implemented a 4-hour trauma-informed care (TIC) symposium for 179 second-year medical students at the George Washington University School of Medicine and Health Sciences during the Patients, Populations, and Systems course. The symposium included three interactive didactic sessions focusing on the connection between trauma and health and TIC principles. A facilitated small-group discussion allowed students to apply TIC principles to a patient case, followed by reflection and evaluation. **Results:** The overall rating of the TIC symposium was 4 out of 5. Strengths included integration of a small-group case with discussion on application of TIC in practice, experience of the lecturers and small-group facilitators, and review of research relating adversity to specific health outcomes. Suggestions for improvement included incorporating role-play and standardized patients. Content analysis of student reflections mapped to the domains of physician competency. **Discussion:** A 4-hour symposium can affect student knowledge and understanding of TIC. Teaching TIC presents an opportunity to prepare medical students for a career in medicine through cultivation of required physician competencies. Next steps include enhanced opportunities to practice TIC and follow-up analysis of participants to determine behavior change during clinical years.

Keywords
Trauma-Informed Care, Adverse Childhood Experiences, Case-Based Learning, Public Health Education, Psychology & Behavioral Science, Communication Skills, Editor's Choice

Educational Objectives

By the end of this activity, learners will be able to:

1. Define trauma and the connection between trauma, childhood adversity, and health.
2. Describe the importance and components of a trauma-informed approach to care.
3. Recognize how to address the health effects of trauma exposure for individual patients and within health systems.
4. Practice employing a trauma-informed approach to patient care.

Citation:
Chokshi B, Walsh K, Dooley D, Falusi O, Deyton L, Beers L. Teaching trauma-informed care: a symposium for medical students. *MedEdPORTAL*. 2020;16:11061.
https://doi.org/10.15766/mep_2374-8265.11061

Introduction

The effect of childhood traumatic exposures on health outcomes throughout the life span is well recognized among pediatric practitioners.[1-4] Given the large body of evidence that links exposure to childhood trauma with negative health outcomes, training the future workforce of physicians on strategies to mitigate the effects of trauma is paramount. Increasing the ability of health care providers to recognize and respond to childhood trauma, such as adverse childhood experiences (ACEs), not only can buffer the long-term negative physical and mental health impacts of traumatic exposures[5] but also can increase patient-centered care[6] and improve patient care indicators.[7] Despite these benefits, health care practitioners have reported discomfort in discussing trauma with their patients,[8] and the impact of traumatic exposures is not routinely addressed.[9]

A trauma-informed approach to care has been employed by health professionals in social work and mental health, but it has not been routinely practiced in medical settings.[10,11] In medicine, trauma-informed care is a shift from the current approach to

Copyright © 2020 Chokshi et al. This is an open-access publication distributed under the terms of the Creative Commons Attribution license.

patient care, which places diagnoses in the context of behaviors, and instead prioritizes understanding medical presentations and diagnoses in the context of life events. A trauma-informed approach to care takes into account the high rates of trauma across populations, focuses on recognition of how traumatic exposures can manifest in patient behaviors and medical and mental health diagnoses, and actively seeks to prevent retraumatization.[12] The central tenet of trauma-informed care is to consider what happened to a person, instead of what is wrong with a person.[12]

It is imperative to incorporate education in trauma-informed care into undergraduate medical education to allow medical trainees to include trauma-informed care principles as they develop their clinical reasoning skills and bedside manner. Additionally, it helps medical students develop a trauma-informed framework that they can then apply and solidify during their clinical years, when they have the opportunity to recognize and potentially respond to traumatic exposures in their patients. Lastly, in the era of coronavirus (COVID-19), employing a trauma-informed approach to care is of even graver importance; the reverberations of the overactivity of the biological stress response during this time will affect populations in both the near and the distant future. As medical students will be on the front lines of caring for populations affected by COVID-19, the need for their education in trauma-informed care is particularly critical.

Current opportunities and models to educate physicians and physician-trainees in the principles of a trauma-informed approach to care are limited.[6,9,13] *MedEdPORTAL* has published two resources directly related to trauma-informed care. One centers on the provision of trauma-informed care to a specific population, female sexual assault survivors.[14] The second focuses on one component of a trauma-informed approach, teaching trauma-informed physical examination techniques to medical students.[15] *MedEdPORTAL* has also published two resources dealing with ACEs that include content related to trauma-informed care. One describes a mandatory 3-hour workshop for first-year medical students at Rutgers New Jersey School of Medicine.[16] This curriculum combines didactics with small-group discussion, and while it does discuss how trauma-informed care can benefit patients, the primary focus is on childhood adversity, specifically, screening for ACEs and the buffering impact of resilience. The second is a 25-minute self-directed module for pediatric residents embedded into an advocacy rotation.[17] That module focuses on defining ACEs and their effect on health, equipping clinicians with tools to recognize how ACEs may contribute to a patient's presentation, and helping students reframe interactions with a trauma-informed lens.[17] While there is a focus on the specifics of the trauma-informed approach, that 25-minute module is concise and therefore lacks a component of interactive discussion and application.[17]

We created an educational intervention, a trauma-informed care symposium (TIC-S), for medical students that focuses specifically on a trauma-informed approach to care. TIC-S is distinct from existing curricula in trauma-informed care and ACEs as it focuses broadly on the effect of trauma and discusses the consideration of the myriad of traumatic exposures beyond the original 10 ACEs, such as racism, gender discrimination, and neighborhood violence. There is also a shift away from the calculation of an ACE score and from encouraging the disclosure of traumatic events. This is in direct response to the changing landscape in the field of childhood adversity, which cautions against the use of ACE questionnaires as screening tools and highlights the potential unintended consequences of screening for adversity.[18,19] The ACE score was not intended to be used as a diagnostic tool or to be the basis of treatment plans.[20] Given the ubiquitous nature of traumatic exposures and the potential for health care settings to be retraumatizing, TIC-S urges students to consider and employ universal trauma precautions for all populations in all clinical interactions.[21] Regarding educational methods, TIC-S is rooted in adult learning principles. We foster active learning through the use of prework to activate learners, Poll Everywhere during didactics, and interactive case-based discussion. In addition, we include a reflection component to help learners solidify their knowledge and brainstorm a plan for how they will incorporate that knowledge in practice.[22]

We integrated this mandatory 4-hour TIC-S into the Patients, Populations, and Systems (PPS) course at the George Washington University School of Medicine and Health Sciences (GW SMHS). PPS is a longitudinal required course series during the first and second years that provides foundational knowledge in the area of public and population health. The course includes didactics that are complemented by interactive small-group sessions. Each small group of 24 students is facilitated by two clinical public health mentors. Mentors are selected through a competitive application process. The GW SMHS faculty members who lead these clinical public health efforts have exceptional clinical experience and collectively provide GW students with deep and wide knowledge and experience in public and population health, health policy, leadership, and advocacy.

Copyright © 2020 Chokshi et al. This is an open-access publication distributed under the terms of the Creative Commons Attribution license.

Methods

Curricular Content

Prior to implementing TIC-S, we surveyed the GW SMHS preclinical curricular database to determine areas where students were exposed to the topics of childhood adversity and trauma-informed care. These included a lecture in the musculoskeletal course focused on child abuse and maltreatment, a lecture during the cardiopulmonary block focused on stress and resilience, and a lecture during the gastroenterology block focused on stress and its relationship to gastrointestinal pathology. Symposium materials were then developed based on the Substance Abuse and Mental Health Services Administration's trauma-informed approach,[12] a review of existing models for trauma-informed care education,[15,16] discussion with content experts through the National Collaborative on Trauma-Informed Health Care Education and Research (TIHCER), of which one author (Binny Chokshi) was a member, and discussion with authors of the THEN curriculum.[23]

Prework: Students were directed to the Centers for Disease Control and Prevention website on childhood adversity[24] and asked to answer three questions, as shown in Appendix A. Students were also given an optional assignment to review Nadine Burke Harris's TED talk on childhood adversity.[25]

Didactic lectures: The didactic component was split into three interactive mini-lectures. For ease of use, these have been combined into one PowerPoint in Appendix A.

1. The Science of Childhood Adversity and the Link to Poor Health Outcomes: This presentation incorporated the prework and reviewed the ACE Study.[1] It then reviewed the physiologic mechanisms by which exposure to trauma, such as ACEs, leads to poor health outcomes, including epigenetic modifications.[16] The section ended with a review of the life course perspective.
2. Trauma-Informed Approach to Clinical Care: This presentation introduced trauma-informed care, gave specific methods for applying it to patient care along with examples, and emphasized the concept of universal trauma precautions. Topics included language to use to informally screen for stress/trauma without a focus on disclosures and how to perform a trauma-informed physical examination,[15] underscoring the importance of collaborating with all patients, explaining questions and procedures, and asking for permission.
3. Partnering With Patients: Building Resiliency and Beyond: This presentation discussed the concept of building resiliency in patients, highlighted a medical home approach to adversity and trauma,[16] and reviewed the Stress Health Self-Care Tool[26] (Appendix B) developed by the Center for Youth Wellness as a way to partner with patients in building resiliency. The presentation ended with a review of how to advocate for a trauma-informed approach on a national level by using the specific example of authors Danielle Dooley and Olanrewaju Falusi advocating for youths detained at the United States–Mexico border.

All mini-lectures included active learning, using Poll Everywhere, and were delivered by the authors Binny Chokshi, Olanrewaju Falusi, Danielle Dooley, and Lee Beers, who were general pediatricians with clinical, educational, and community research experience in the field of childhood adversity and trauma-informed care.

Small-group case-based discussion: Following the didactics, students separated into their PPS small groups and worked through a patient case, as shown in Appendix C, facilitated by their small-group mentors. The case focused on a 40-year-old woman with an extensive history of traumatic exposures and asked students to consider the scenario from the perspectives of both patient and provider. The case discussion questions included questions utilized in the THEN curriculum[23] and asked students to discern the utility of patient tools, such as the Stress Health Self-Care Tool.[26] The case ended with a focus on the barriers to employing a trauma-informed approach to care and a discussion of the potential patient outcomes if a trauma-informed lens was not applied. Following this, students were allotted 30 minutes of class time to complete an evaluation that included a reflective component (Appendix D).

Faculty preparation: The PPS small groups were facilitated by GW SMHS clinical public health faculty mentors who had extensive experience working at the intersection of public health and medicine. Faculty participated in a 30-minute interactive session (led by author Binny Chokshi) to review the TIC-S content and cases to ensure that all felt equipped to facilitate the small-group sessions (Appendix E). Faculty were also given an answer key for the small-group case, which included points to foster discussion (Appendix C).

Evaluation

The evaluation (Appendix D) was electronically implemented and anonymously collected through the Qualtrics platform. The evaluation was developed by authors Binny Chokshi and Kimberly Walsh based on a literature review of similar curricular

Copyright © 2020 Chokshi et al. This is an open-access publication distributed under the terms of the Creative Commons Attribution license.

evaluations and pilot tested with authors Olanrewaju Falusi and Danielle Dooley for content validity. The evaluation included five Likert-scale questions related to TIC-S's effect on learner knowledge and plans to utilize that knowledge in practice. Four short-answer questions sought student feedback on curricular elements, and two reflection questions asked students to discuss a patient case in which trauma may have played a role in the patient's diagnosis and how the information learned during TIC-S would affect their patient care rotations. As the first was simply a recall question to serve as a primer, only the second question was included in our analysis. As part of an ongoing research grant through Children's National Hospital, the evaluation included one question related to trafficked youth and adolescents, which would be eliminated when the symposium is replicated. The George Washington University Institutional Review Board deemed our study exempt from review.

Data Analysis

We used descriptive statistics to analyze data for the five Likert-scale questions. Three authors (Binny Chokshi, Danielle Dooley, and Kimberly Walsh) independently reviewed the short-answer questions and then came together to discuss and create descriptive frequencies for the most common answer choices. Lastly, three authors (Binny Chokshi, Olanrewaju Falusi, and Kimberly Walsh), with the use of NVIVO software, performed content analysis of one reflection question by first individually reviewing and coding the student responses and then coming together for discussion. In early discussion, we recognized that the content of the student reflections mapped to four of the six Accreditation Council for Graduate Medical Education (ACGME) domains of competence: (1) patient care and procedural skills, (2) medical knowledge, (3) interpersonal and communication skills, and (4) professionalism.[27,28] To systematically organize the content of the student reflections, we utilized the definitions of the competencies listed in Table 1. Binny Chokshi and Kimberly Walsh individually mapped each student reflection to the competencies and then came together to reconcile any inconsistencies.

Results

A total of 179 GW SMHS students participated in TIC-S. The overall rating of TIC-S on a 5-point scale (1 = *Strongly Disagree,* 5 = *Strongly Agree*) was 4. Responses to the four Likert-scale questions are shown in the Figure, which indicates the raw numbers of responses. In summary, 175 participants (92%) agreed or strongly agreed that TIC-S increased their knowledge of the relationship between ACEs and health outcomes, and 167 participants (93%) indicated that TIC-S increased their understanding of trauma-informed care. In addition, 156 participants (87%) agreed or strongly agreed that they had a better understanding of how to incorporate trauma-informed practices during patient interactions, and 162 participants (91%) agreed or strongly agreed that they planned to use the concepts learned in TIC-S during their clinical rotations.

Respondents indicated that the strengths of the training included small-group discussion, the experiences of the lecturers, the examples of application, and the overview of the epidemiology linking adversity to poor health outcomes (Table 2). Suggestions for improvement to TIC-S fell into two categories: (1) additional opportunities for practice, such as through the use of standardized patients, question practice with role-play, or additional case studies, and (2) changes to the symposium format, including decreasing lecture time and including real patients (Table 2). Students reported clinical considerations, the prevalence of ACEs and ACE-related health outcome statistics, and the data on life expectancy as what they found most surprising (Table 2).

As stated above, descriptive content analysis of students' responses regarding their plans to translate TIC-S content into clinical practice mapped to four of six ACGME core domains of competence.[28] Specifically, as shown in Table 1, 132 out of 179 (73%) mapped to patient care and procedural skills, 76 out of 179 (42%) to medical knowledge, 120 out of 179 (67%) to interpersonal and communication skills, and 58 out of 179 (35%) to professionalism. Table 1 includes the AGCME definitions of these four domains as well as discipline-specific examples of subcompetencies within each domain of competence relevant to TIC-S.[27,29-31] In addition, Table 1 contains representative quotations extracted from student reflections.

Discussion

To effectively address the effects of childhood adversity, health care providers must not only understand its impact on an individual's health and well-being but also learn the skills to employ a trauma-informed approach to care, which acknowledges the events, experiences, and effects of individual trauma. Our experience demonstrates that a 4-hour required medical student symposium, TIC-S, can increase students' knowledge and understanding of trauma-informed care and their desire to consider trauma during clinical interactions. TIC-S can also reinforce the domains of physician competence delineated by the ACGME.[28]

Copyright © 2020 Chokshi et al. This is an open-access publication distributed under the terms of the Creative Commons Attribution license.

Table 1. Descriptive Content Analysis of the Reflection Question "How Will the Information You Learned in the Symposium Today Affect Your Patient Care Rotations?"

Competency	Description and Subcompetencies	No.	Representative Quotes
Patient care and procedural skills	Provide patient-centered care that is compassionate, appropriate, and effective for the treatment of health problems and promotion of health.<br>• Gather essential and accurate information about the patient.<br>• Interview patients and families about the particulars of the medical condition for which they seek care, with specific attention to behavioral, psychosocial, environmental, and family unit correlates of disease.<br>• Counsel and educate patients and their families.	132	"I will definitely be aware of ACEs when taking a patient history and know how to address it as part of the patient visit. I will also use some of the scripts to lean into discussing the ACEs and how they affect the health of the patient."<br>"I will know that our job is not to have patients expose their trauma because we cannot treat the trauma specifically. Our role is to explain the science behind ACEs and help empower patients by understanding how their ACEs may affect their health outcome."<br>"I will use the information I learned today to make sure to not look at a patient's health and medical conditions with a solely science and clinically oriented mind. I will make sure to ask questions about any patient experiences that could be affecting their health."
Medical knowledge	Demonstrate knowledge of established and evolving biomedical, clinical, epidemiological, and social behavioral sciences, as well as application of this knowledge to patient care.<br>• Demonstrate an investigatory and analytic approach to clinical situations.<br>• Apply principles of social-behavioral sciences to provision of patient care, including assessment of the impact of psychosocial-cultural influences on health, disease, compliance, care seeking, and barriers to and attitudes towards care.	76	"One example of how this will impact my patient care rotations is to be aware of the impact of psychosocial impacts such as adverse childhood experiences on medical care and patient health outcomes. This may manifest in itself while discussing treatment plans to account for these factors. A second example is to have this knowledge to alter communication with the patient during the history and physical exam. Since the incidence of ACEs are so high, it is important to ask questions that take into account these factors and to perform physical exam maneuvers taking into account a potential for past abuse and physical/emotional triggers."<br>"I will approach all patients with a trauma informed lens, using the universal precautions. It is not my goal to discover what the trauma was, just use a trauma lens to provide the best possible treatment for my patients."<br>"I will be more aware of how trauma can affect health and consider how this relates to my specific patients. I will also use the techniques learned today to help patients manage stress and other challenges to decrease the effects of trauma on their health."
Interpersonal and communication skills	Demonstrate interpersonal and communication skills that result in the effective exchange of information and collaboration with patients, their families, and health professionals.<br>• Demonstrate sensitivity, honesty, and compassion in difficult conversations (e.g., about issues such as death, end-of-life issues, adverse events, bad news, disclosure of errors, and other sensitive topics).<br>• Demonstrate insight and understanding about emotions and human responses to emotions that allow one to develop and manage interpersonal interactions.	120	"I will be more mindful of what people would consider 'noncompliant patients.' It's always better to ask questions than assume a patient is not following a plan because they simply don't want to."<br>"I will be more cognizant of assuming all patients have experienced some type of trauma. I will remember to use outside resources and other specially trained professionals to help address trauma related issues that I am not equipped to deal with."<br>"In rotations, I want to be a part of a care team that asks for permission when I interview and do a PE [physical exam]. Also I want to have an asset based approach to care and acknowledge the strengths that the patient has."
Professionalism	Demonstrate a commitment to carrying out professional responsibilities and adherence to ethical principles.<br>• Demonstrate compassion, integrity, and respect for others.<br>• Demonstrate sensitivity and responsiveness to a diverse patient population including but not limited to diversity in gender, age, culture, race, religion, disabilities, and sexual orientation.	63	"It will affect me by being able to acknowledge the effect of life stressors, or in this situation, traumas. Acknowledging these, such as poverty, sexual abuse, physical abuse, etc., [can] then help me connect with my patient better and further improve their treatment."<br>"I will be extra sensitive and take a thorough history. I will always emphasize that my office is a safe space and how my patients can disclose anything, but disclosure isn't the goal. The goal is to provide her comprehensive medical care and ensure her safety."<br>"I will not make judgments or assumptions on other people without first getting a patient history. I will be more empathetic to those around me—as Great Gatsby opens with 'Not everyone in life has the same privileges as you' (paraphrasing here). Thank you and I think that it will definitely make a small difference in a more caring and sensitive way I will interact [with] patients."

Abbreviation: ACE, adverse childhood experience.

While TIC-S begins with a discussion of the original ACE Study and the link between adversity and health outcomes, it goes on to develop an overall focus on the relationship between stress and health across the life span. It urges participants to recognize that adversity is not limited to the 10 traumas analyzed in the landmark ACE Study[1] and shifts the focus away from screening for, or attempting to obtain disclosure of, specific traumas. Instead, TIC-S calls for universal trauma precautions

Copyright © 2020 Chokshi et al. This is an open-access publication distributed under the terms of the Creative Commons Attribution license.

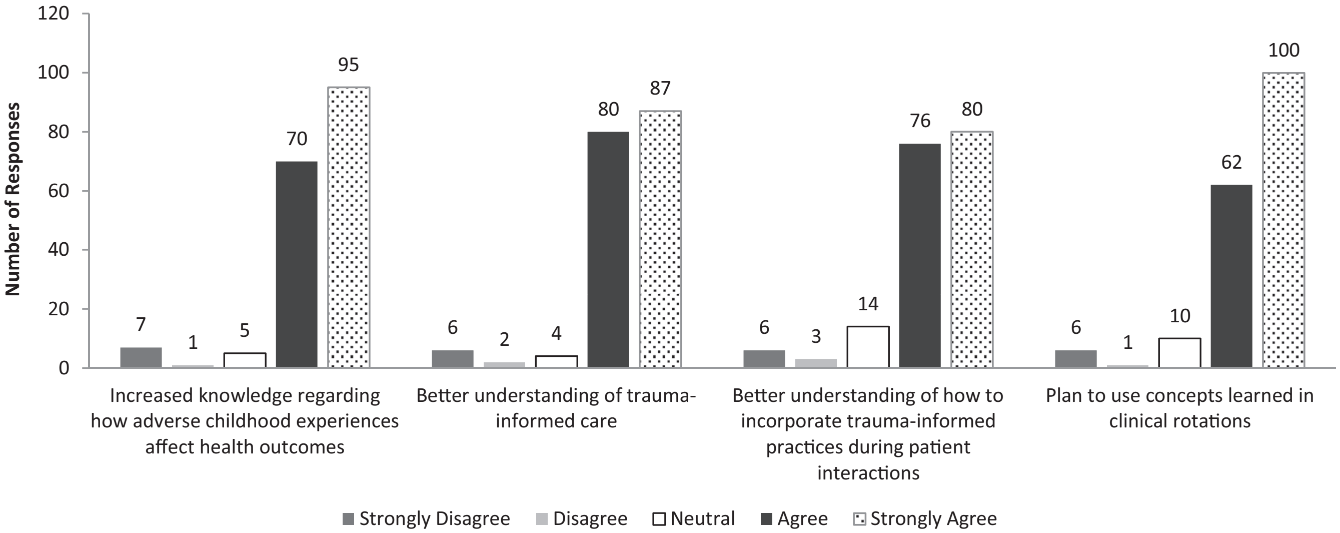

Figure. Responses from trauma-informed care symposium participants (N = 179) indicating their agreement with evaluation statements, with ratings based on a 5-point Likert scale.

and the consideration of a trauma-informed approach in all clinical interactions. It features specific directions for how to apply trauma-informed care in practice, including language to informally screen for stress and trauma and steps to prevent retraumatization of patients, such as through the use of a trauma-informed physical examination approach. TIC-S also trains students on strategies for collaborating with patients to build resiliency, both through counseling and educating patients on the relationship between stress and health and also through the use of tools such as the Stress Health Self-Care Tool.[26]

Our evaluation results highlight that the ACEs science was surprising and riveting for students and therefore needs to be included to build student motivation in understanding and employing a trauma-informed approach to care. In addition, students appreciated active learning opportunities, such as small groups and case-based discussion. The experience of lecturers can also help to build support for the content. Lastly, student reflections regarding how they planned to apply the knowledge learned mapped to four of six ACGME domains of competence.[28] As medical education has moved towards competency-based education, there is specific emphasis on curricula and evaluation methods that help to increase exposure to and attainment of these competencies. While there are currently no agreed-upon competencies or milestones for medical students, the ACGME competency domains serve as a roadmap for undergraduate medical educators to ensure that students graduate with the skills to excel in residency and beyond. That student reflections on implementing learning from TIC-S mapped to four of six physician domains of competence lends credence to the importance of trauma-informed education for all medical students. Education in trauma-informed care is an innovative way to assist medical students in attaining required competencies. In addition, our evaluation results highlight that a focus on universal trauma precautions, instead of on patient-specific adversity, helps make this symposium broadly applicable across competencies by concentrating on an overall approach to patient care versus one relevant only in specific situations and settings.

Based on our evaluation results, our primary lesson learned is that we need to include additional opportunities for practice and feedback within TIC-S. In the next iteration, we plan to include an example role-play with a patient during the large-group session and to build in opportunities for practice and feedback with standardized patients during the small-group session. We also plan to collaborate with leadership for the clinical skills course at GW SMHS with the expectation of incorporating trauma-informed physical examination techniques as a complement to TIC-S. In addition, GW SMHS is home to a large simulation center, and we hope to continue to collaborate with members of the TIHCER community to cocreate simulation cases appropriate for the medical student level that can be piloted at GW SMHS. Lastly, practicing trauma-informed care has implications for vicarious trauma, secondary traumatic stress, compassion fatigue, and burnout. We plan to incorporate a focus on these issues in future iterations of TIC-S.

The limitations of our symposium include that we did not directly evaluate our learning objectives and, importantly, that we do not have data to support sustained knowledge acquisition or

Copyright © 2020 Chokshi et al. This is an open-access publication distributed under the terms of the Creative Commons Attribution license.

Table 2. Student Evaluations of Trauma-Informed Care Symposium (N = 179)

Question and Themes	No.[a]	Representative Quotes
Which parts of the symposium were most helpful to increase your knowledge on adverse childhood experiences and trauma informed care?		
Small-group discussion	69	"The small group session helped pull everything together and apply what was learned in the lecture."
Lecturer's experiences	38	"I really enjoyed hearing from the lecturers and listening to their responses to questions asked by the class."
Application examples	58	"I thought it was helpful to learn specifically how to approach ACEs and trauma during a patient encounter."
Epidemiology presentations and statistics	69	"I appreciated the statistics and the material presented on how ACEs affect different dimensions of a person's health."
What changes to the symposium would you recommend to benefit student learning?		
Practice opportunities:		
Standardized patients	4	"I would recommend maybe bringing in [a standardized patient] to demonstrate real-time how to talk to a patient appropriately to uncover these details from their past."
Questions with role-play	14	"Potentially a chance to practice asking the probing or intro questions to learn about a patient's trauma."
Additional case studies	15	"More patient examples would be helpful to solidify concepts."
Symposium format:		
Reduce lecture time	29	"More time spent on the role of physicians and less time establishing the legitimacy of ACEs effects on health."
Inclusion of real patients	8	"Opportunities to talk to people with ACEs and practice the recommended strategies rather than just discussing them."
What surprised you most about what you learned today?		
Clinical considerations	29	"I was surprised that the incidence of ACEs was 64%. I didn't expect this figure to be as high. Due to the weight of this figure it is important to take these into account during all patient encounters as it is impossible to know whether your patient was also impacted by ACEs."
ACE connection to health outcomes	34	"The decrease in lifespan associated with ACEs as well as the increase in chronic conditions such as COPD [chronic obstructive pulmonary disease], asthma, and cancer associated with experiencing ACEs."
ACE prevalence and statistics	34	"How the data really deeply shows that these ACEs really have a huge effect on a patient's health, way bigger than I would have assumed. It was helpful that today we were not just informed of this but also told in very practical ways how we could help in the future."
Life expectancy data	69	"The 20 years of life expectancy lost with 6+ ACES really resonated with me and was something I hadn't fully internalized. I also liked the dose-response curve graph showing that the # of ACEs you are exposed to basically directly correlates with outcomes. I think that's really compelling evidence."

Abbreviation: ACE, adverse childhood experience.
[a]Indicates the number of responses fitting the theme.

an effect on behavior change. For further evaluation of TIC-S, we plan to include pre-post assessment of knowledge, attitudes, practice, and confidence related to trauma-informed care. In addition, we would like to include follow-up with participants at an interval of 6-9 months to discern if and how they are able to apply a trauma-informed approach to patient cases during their clinical rotations. We also hope that this qualitative inquiry will lend insight into how trauma-informed care training may continue in the clinical years symposium.

As pervasive as trauma is, we believe that our symposium is generalizable to any institution seeking to train the future workforce of physicians in the ideals of a trauma-informed approach to care. Our symposium materials can easily be adapted for an existing course. Our systems-level example of advocacy and policy for federal detention of migrant children can be considered an optional component of the symposium and adapted to the expertise of the lecturers. We chose to include it in TIC-S given the PPS course focus on patients, populations, and systems. In the current medical and public health climate, institutions could choose to link the content from TIC-S to processes and systems that have been developed in response to the stress-related health effects of COVID-19 or the traumatic effects of systemic and institutional racism.

A challenge of the TIC-S implementation was finding protected time in an already busy academic calendar. At our institution, TIC-S fell between two exams for the medical students. We urge other institutions to consider optimal placement in order to ensure the best success and receipt of the symposium.

Copyright © 2020 Chokshi et al. This is an open-access publication distributed under the terms of the Creative Commons Attribution license.

In addition, although we were able to leverage the baseline knowledge of adversity and trauma-informed care of our institution's clinical public health mentors, they still required a brief preparatory session to be able to lead the small-group sessions. If other institutions lack this faculty expertise, the small-group case can easily be adapted for a large-group format to decrease the number of faculty required. Lastly, with any education related to trauma-informed care, instructors must consider the potential effect of this sensitive topic on students. At GWU SMHS, the PPS syllabus included direct contact information for a student affairs dean and 24/7 health line for students in the event of a distressing reaction to presented materials.

Based on our experience and student responses, we recommend that trauma-informed education be required for all preclinical medical students and that accrediting bodies such as the Liaison Committee on Medical Education urge medical schools to include and prioritize these important curricula.

Appendices

A. TIC-S PowerPoint.pptx

B. Stress Health Self-Care Tool.pdf

C. Facilitator Guide.docx

D. Evaluation.docx

E. Facilitator Prep Slides.pptx

All appendices are peer reviewed as integral parts of the Original Publication.

Binny Chokshi, MD: Pediatrician and Co-Medical Director Healthy Generations, Children's National Hospital; Assistant Professor of Pediatrics, George Washington University School of Medicine and Health Sciences; ORCID: https://orcid.org/0000-0001-9670-910X

Kimberly Walsh, MPA, RN: Clinical Research Coordinator, Children's National Hospital

Danielle Dooley, MD, MPhil: Pediatrician and Medical Director of Community Affairs and Population Health, Child Health Advocacy Institute, Children's National Hospital; Assistant Professor of Pediatrics and Co-Course Director, Patients Populations and Systems, George Washington University School of Medicine and Health Sciences

Olanrewaju Falusi, MD: Pediatrician and Medical Director Advocacy Education, Child Health Advocacy Institute, Children's National Hospital; Assistant Professor of Pediatrics, George Washington University School of Medicine and Health Sciences

Lawrence Deyton, MD, MSPH: Senior Associate Dean of Clinical Public Health, Murdock Head Professor of Medicine and Health Policy, and Professor of Medicine, George Washington University School of Medicine and Health Sciences

Lee Beers, MD: Pediatrician and Medical Director for Municipal and Regional Affairs, Child Health Advocacy Institute, Children's National Hospital; Co-Director Early Childhood Innovation Network, Children's National Hospital; Associate Professor of Pediatrics, George Washington University School of Medicine and Health Sciences

Acknowledgments
The authors thank Ruchi Parikh, MBA, for her assistance with image adaption; Lisa Tuchman, MD, for her support of curriculum development; Cara Lichtenstein, MD, for her review of the manuscript; and the members of the National Collaborative on Trauma-Informed Health Care Education and Research, whose gracious sharing of resources and insight was critical to the success of this symposium.

Disclosures
None to report.

Funding/Support
None to report.

Ethical Approval
The George Washington University Institutional Review Board approved this study.

References

1. Felitti VJ, Anda RF, Nordenberg D, et al. Relationship of childhood abuse and household dysfunction to many of the leading causes of death in adults: the Adverse Childhood Experiences (ACE) Study. *Am J Prev Med*. 1998;14(4):245-258. https://doi.org/10.1016/S0749-3797(98)00017-8

2. Shonkoff JP, Garner AS; Committee on Psychosocial Aspects of Child and Family Health; Committee on Early Childhood, Adoption, and Dependent Care; Section on Developmental and Behavioral Pediatrics. The lifelong effects of early childhood adversity and toxic stress. *Pediatrics*. 2012;129(1):e232-e246. https://doi.org/10.1542/peds.2011-2663

3. Smith JP, Smith GC. Long-term economic costs of psychological problems during childhood. *Soc Sci Med*. 2010;71(1):110-115. https://doi.org/10.1016/j.socscimed.2010.02.046

4. Bethell CD, Newacheck P, Hawes E, Halfon N. Adverse childhood experiences: assessing the impact on health and school engagement and the mitigating role of resilience. *Health Aff (Millwood)*. 2014;33(12):2106-2115. https://doi.org/10.1377/hlthaff.2014.0914

5. Goldstein E, Murray-García J, Sciolla AF, Topitzes J. Medical students' perspectives on trauma-informed care training. *Perm J*. 2018;22:17-126. https://doi.org/10.7812/TPP/17-126

6. Green BL, Saunders PA, Power E, et al. Trauma-informed medical care: CME communication training for primary care providers. *Fam Med*. 2015;47(1):7-14.

Copyright © 2020 Chokshi et al. This is an open-access publication distributed under the terms of the Creative Commons Attribution license.

7. Felitti VJ, Anda RF. The lifelong effects of adverse childhood experiences. In: Chadwick DL, Giardino AP, Alexander R, Thackeray JD, Esernio-Jenssen D, eds. *Chadwick's Child Maltreatment: Vol. 2. Sexual Abuse and Psychological Maltreatment*. 4th ed. STM Learning; 2014:203-215.
8. Green BL, Kaltman S, Frank L, et al. Primary care providers' experiences with trauma patients: a qualitative study. *Psychol Trauma*. 2011;3(1):37-41. https://doi.org/10.1037/a0020097
9. Oral R, Ramirez M, Coohey C, et al. Adverse childhood experiences and trauma informed care: the future of health care. *Pediatr Res*. 2016;79(1-2):227-233. https://doi.org/10.1038/pr.2015.197
10. Koetting C. Trauma-informed care: helping patients with a painful past. *J Christ Nurs*. 2016;33(4):206-213. https://doi.org/10.1097/CNJ.0000000000000315
11. Marsac ML, Kassam-Adams N, Hildenbrand AK, et al. Implementing a trauma-informed approach in pediatric health care networks. *JAMA Pediatr*. 2016;170(1):70-77. https://doi.org/10.1001/jamapediatrics.2015.2206
12. Substance Abuse and Mental Health Services Administration. *SAMHSA's Concept of Trauma and Guidance for a Trauma-Informed Approach*. Substance Abuse and Mental Health Services Administration; 2014. HHS publication (SMA) 14-4884.
13. Li Y, Cannon LM, Coolidge EM, Darling-Fisher CS, Pardee M, Kuzma EK. Current state of trauma-informed education in the health sciences: lessons for nursing. *J Nurs Educ*. 2019;58(2): 93-101. https://doi.org/10.3928/01484834-20190122-06
14. Siegel M, Chen Gonzalez E, Wijesekera O, et al. On-the-go training: downloadable modules to train medical students to care for adult female sexual assault survivors. *MedEdPORTAL*. 2017; 13:10656. https://doi.org/10.15766/mep_2374-8265.10656
15. Elisseou S, Puranam S, Nandi M. A novel, trauma-informed physical examination curriculum for first-year medical students. *MedEdPORTAL*. 2019;15:10799. https://doi.org/10.15766/mep_2374-8265.10799
16. Pletcher BA, O'Connor M, Swift-Taylor ME, DallaPiazza M. Adverse childhood experiences: a case-based workshop introducing medical students to trauma-informed care. *MedEdPORTAL*. 2019;15:10803. https://doi.org/10.15766/mep_2374-8265.10803
17. Schmitz A, Light S, Barry C, Hodges K. Adverse childhood experiences and trauma-informed care: an online module for pediatricians. *MedEdPORTAL*. 2019;15:10851. https://doi.org/10.15766/mep_2374-8265.10851
18. Finkelhor D. Screening for adverse childhood experiences (ACEs): cautions and suggestions. *Child Abuse Negl*. 2018;85:174-179. https://doi.org/10.1016/j.chiabu.2017.07.016
19. Campbell TL. Screening for adverse childhood experiences (ACEs) in primary care: a cautionary note. *JAMA*. 2020;323(23):2379-2380. https://doi.org/10.1001/jama.2020.4365
20. Anda RF, Porter LE, Brown DW. Inside the adverse childhood experience score: strengths, limitations, and misapplications. *Am J Prev Med*. 2020;59(2):293-295. https://doi.org/10.1016/j.amepre.2020.01.009
21. Racine N, Killam T, Madigan S. Trauma-informed care as a universal precaution: beyond the adverse childhood experiences questionnaire. *JAMA Pediatr*. 2020;174(1):5-6. https://doi.org/10.1001/jamapediatrics.2019.3866
22. Song P, Stewart R. Reflective writing in medical education. *Med Teach*. 2012;34(11):955-956. https://doi.org/10.3109/0142159X.2012.716552
23. Rush P, Stillerman A. Core concepts. THEN Center. Accessed April 30, 2020. https://www.thencenter.org/coreconcepts
24. About the CDC–Kaiser ACE Study. Centers for Disease Control and Prevention. Accessed February 21, 2020. https://www.cdc.gov/violenceprevention/childabuseandneglect/acestudy/about.html
25. Harris NB. How childhood trauma affects health across a lifetime. Video. TED. September 2014. Accessed February 21, 2020. https://www.ted.com/talks/nadine_burke_harris_how_childhood_childhood_trauma_affects_health_across_a_lifetime
26. Center for Youth Wellness, Bayview Child Health Center. *Stress Health Self-Care Tool*. Center for Youth Wellness, Bayview Child Health Center; 2018.
27. Englander R, Cameron T, Ballard AJ, Dodge J, Bull J, Aschenbrener CA. Toward a common taxonomy of competency domains for the health professions and competencies for physicians. *Acad Med*. 2013;88(8):1088-1094. https://doi.org/10.1097/ACM.0b013e31829a3b2b
28. Accreditation Council for Graduate Medical Education. *ACGME Common Program Requirements (Residency)*. Accreditation Council for Graduate Medical Education; 2019. Accessed March 10, 2020. https://www.acgme.org/Portals/0/PFAssets/ProgramRequirements/CPRResidency2019.pdf
29. *ACGME Program Requirements for Graduate Medical Education in Pediatrics*. Accreditation Council for Graduate Medical Education; 2020. https://www.acgme.org/Portals/0/PFAssets/ProgramRequirements/320_Pediatrics_2020.pdf?ver=2020-06-29-162726-647
30. *ACGME Program Requirements for Graduate Medical Education in Internal Medicine*. Accreditation Council for Graduate Medical Education; 2020. https://www.acgme.org/Portals/0/PFAssets/ProgramRequirements/140_InternalMedicine_2020.pdf?ver=2020-06-29-161610-040
31. *ACGME Program Requirements for Graduate Medical Education in Family Medicine*. Accreditation Council for Graduate Medical Education; 2020. https://www.acgme.org/Portals/0/PFAssets/ProgramRequirements/120_FamilyMedicine_2020.pdf?ver=2020-06-29-161615-367

Received: May 12, 2020
Accepted: September 11, 2020
Published: December 30, 2020

Copyright © 2020 Chokshi et al. This is an open-access publication distributed under the terms of the Creative Commons Attribution license.
