# Supplementary material for: Teaching Trauma-Informed Care: A Symposium for Medical Students
Source: MedEdPORTAL. 2020 Dec 30;16:11061. doi: 10.15766/mep_2374-8265.11061 (PMC7780743; doi:10.15766/mep_2374-8265.11061)

## Slide 1
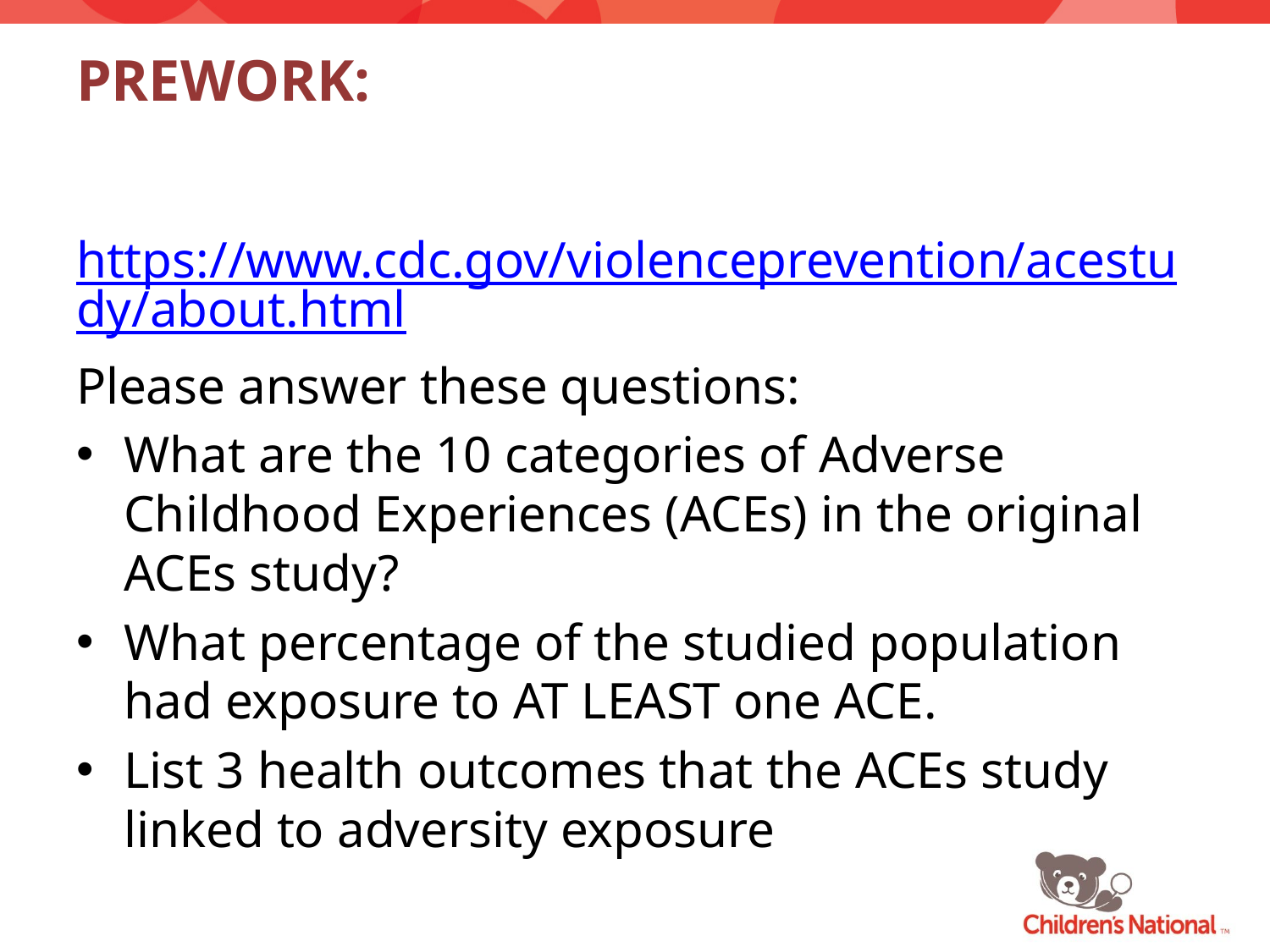

# PREWORK:
https://www.cdc.gov/violenceprevention/acestudy/about.html
Please answer these questions:
What are the 10 categories of Adverse Childhood Experiences (ACEs) in the original ACEs study?
What percentage of the studied population had exposure to AT LEAST one ACE.
List 3 health outcomes that the ACEs study linked to adversity exposure

## Slide 2
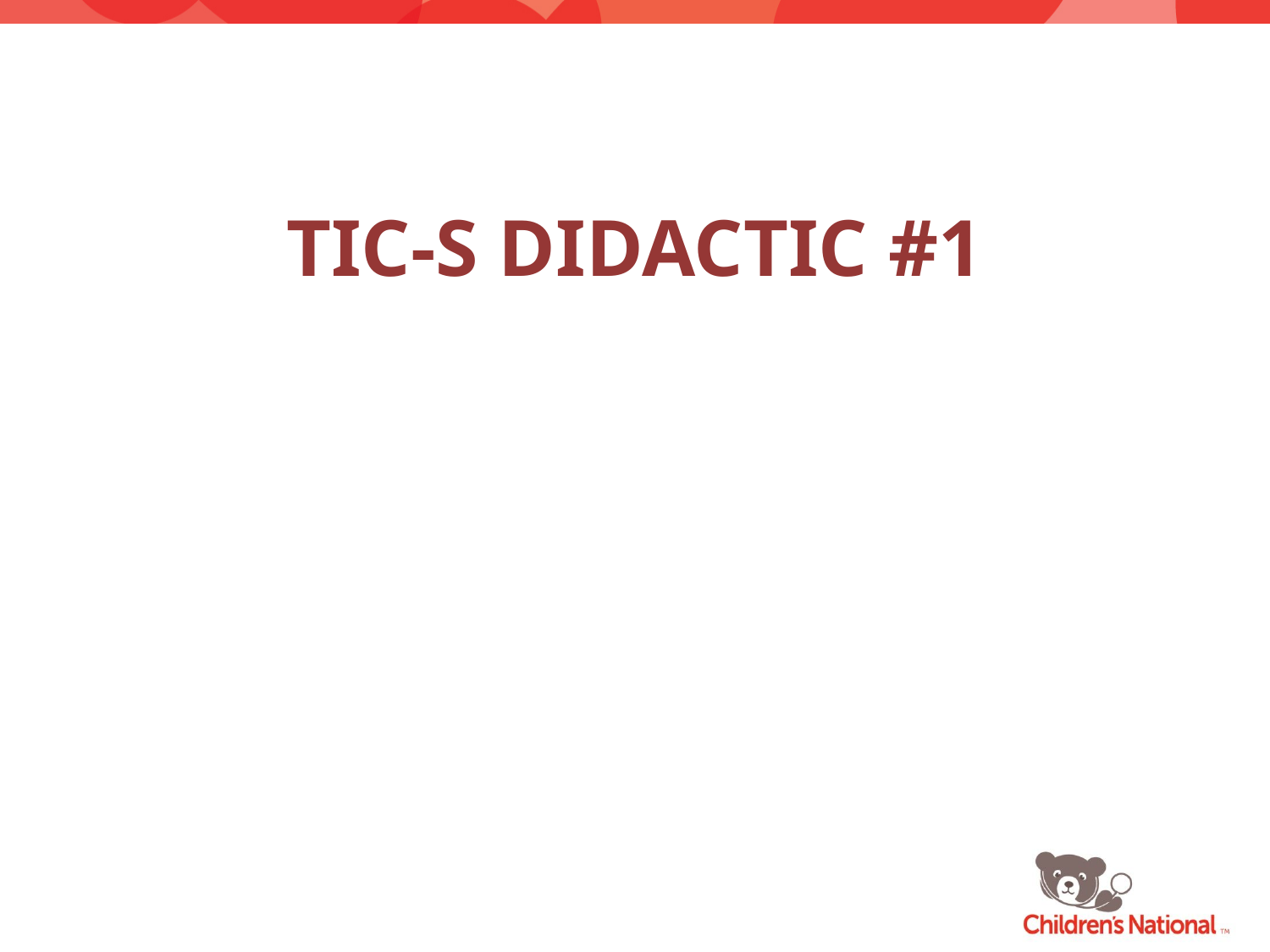

TIC-S DIDACTIC #1

## Slide 3
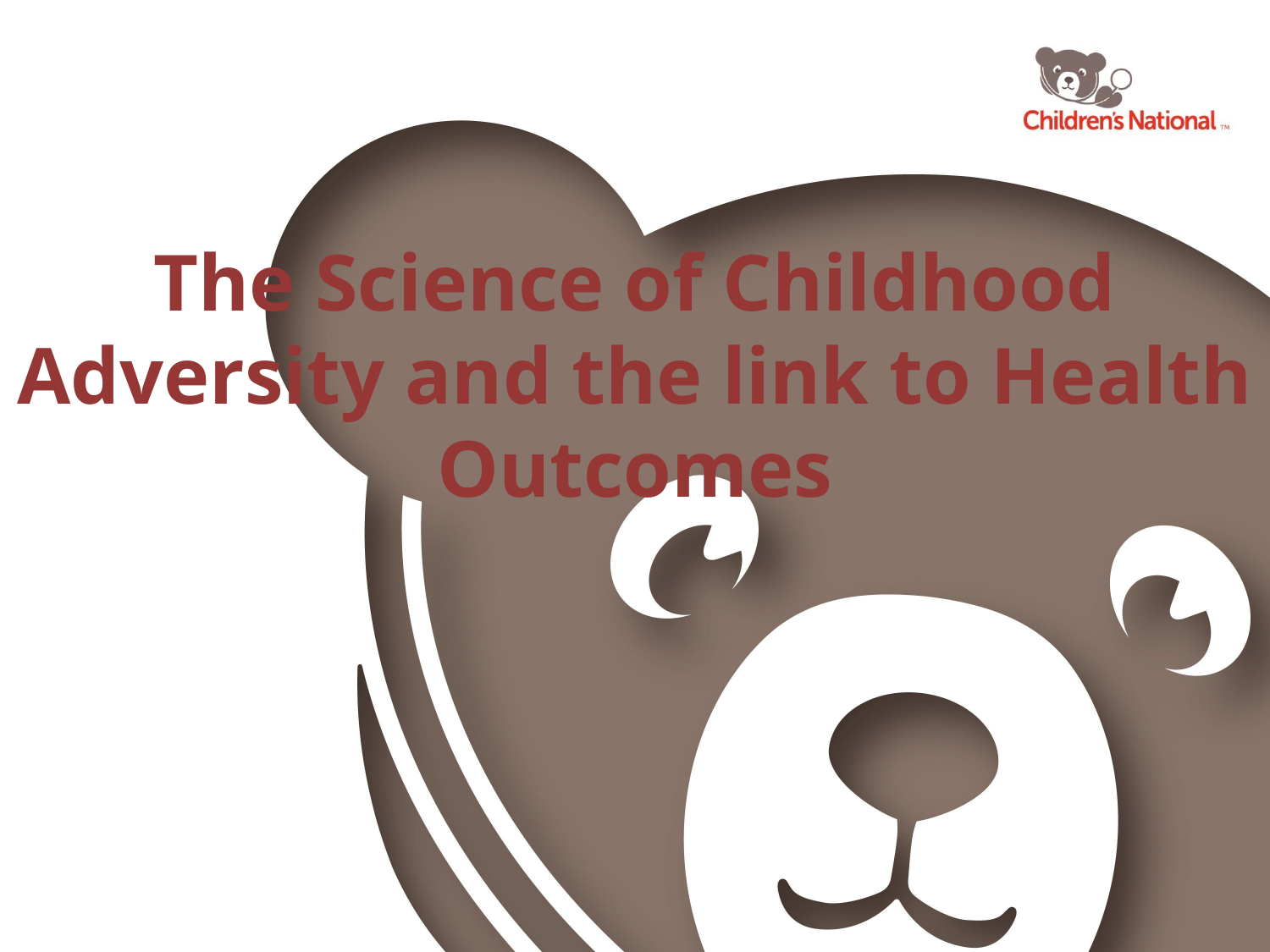

# The Science of Childhood Adversity and the link to Health Outcomes

## Slide 4
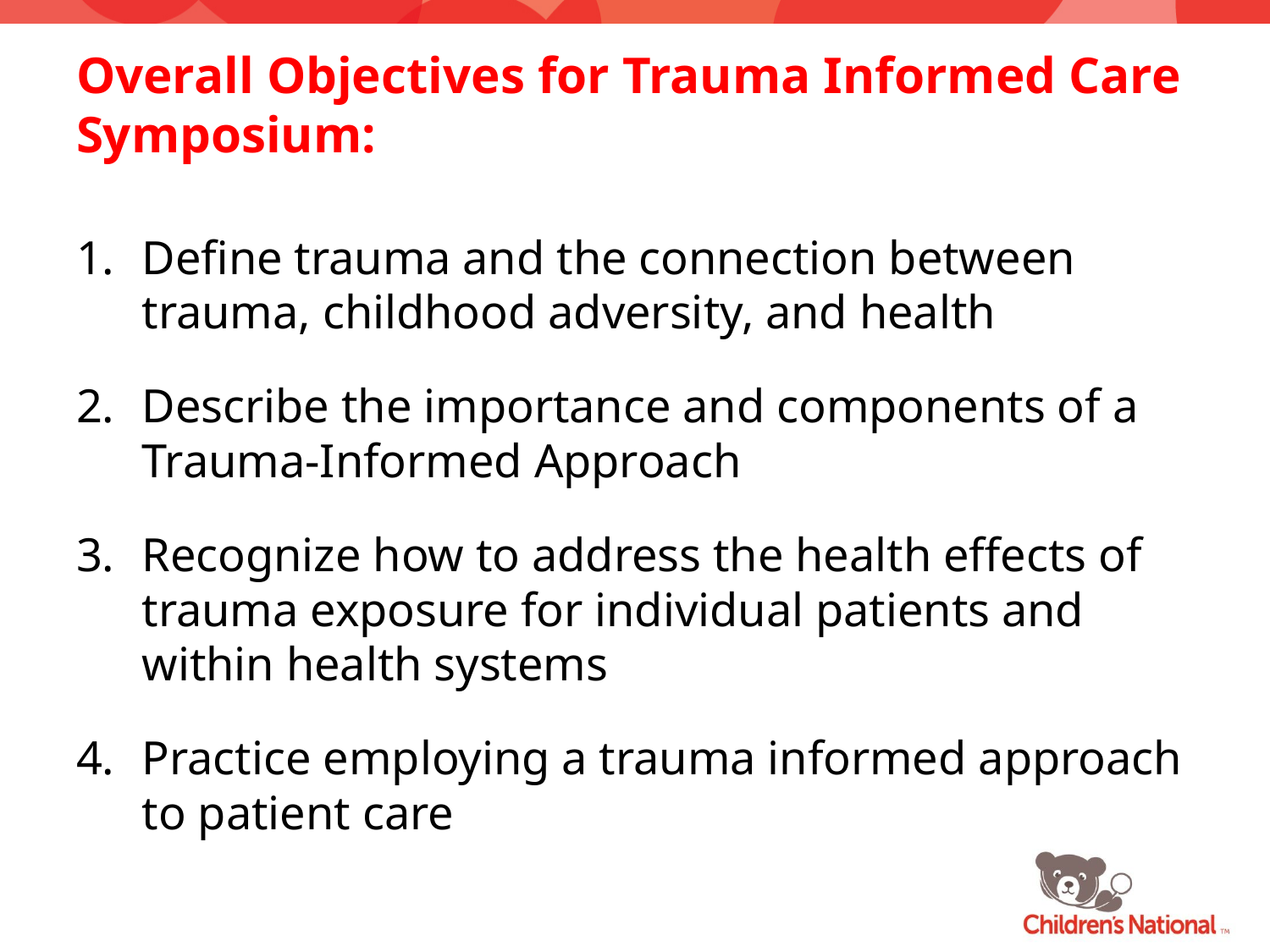

# Overall Objectives for Trauma Informed Care Symposium:
Define trauma and the connection between trauma, childhood adversity, and health
Describe the importance and components of a Trauma-Informed Approach
Recognize how to address the health effects of trauma exposure for individual patients and within health systems
Practice employing a trauma informed approach to patient care

## Slide 5
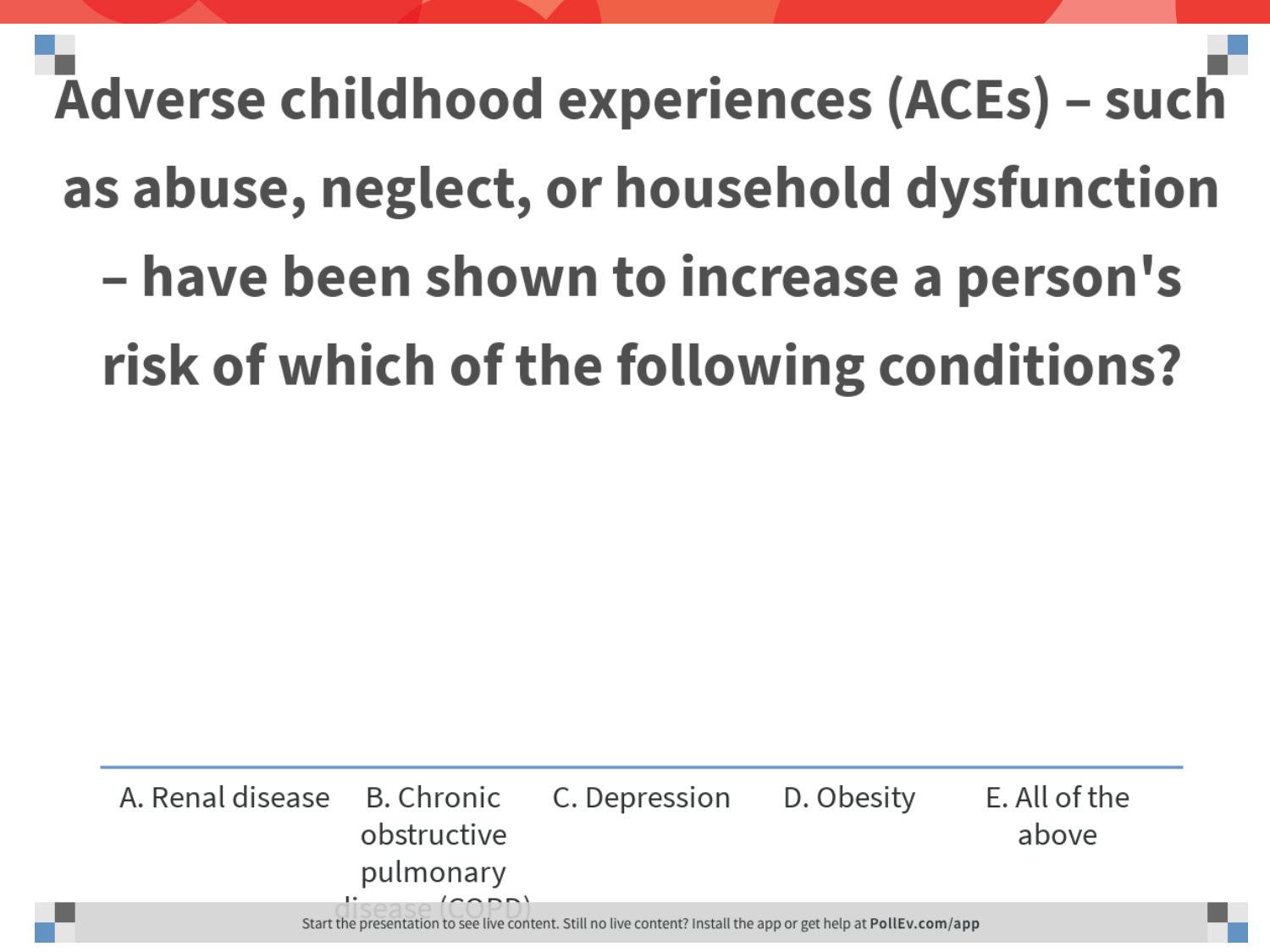

August 31, 2020

## Slide 6
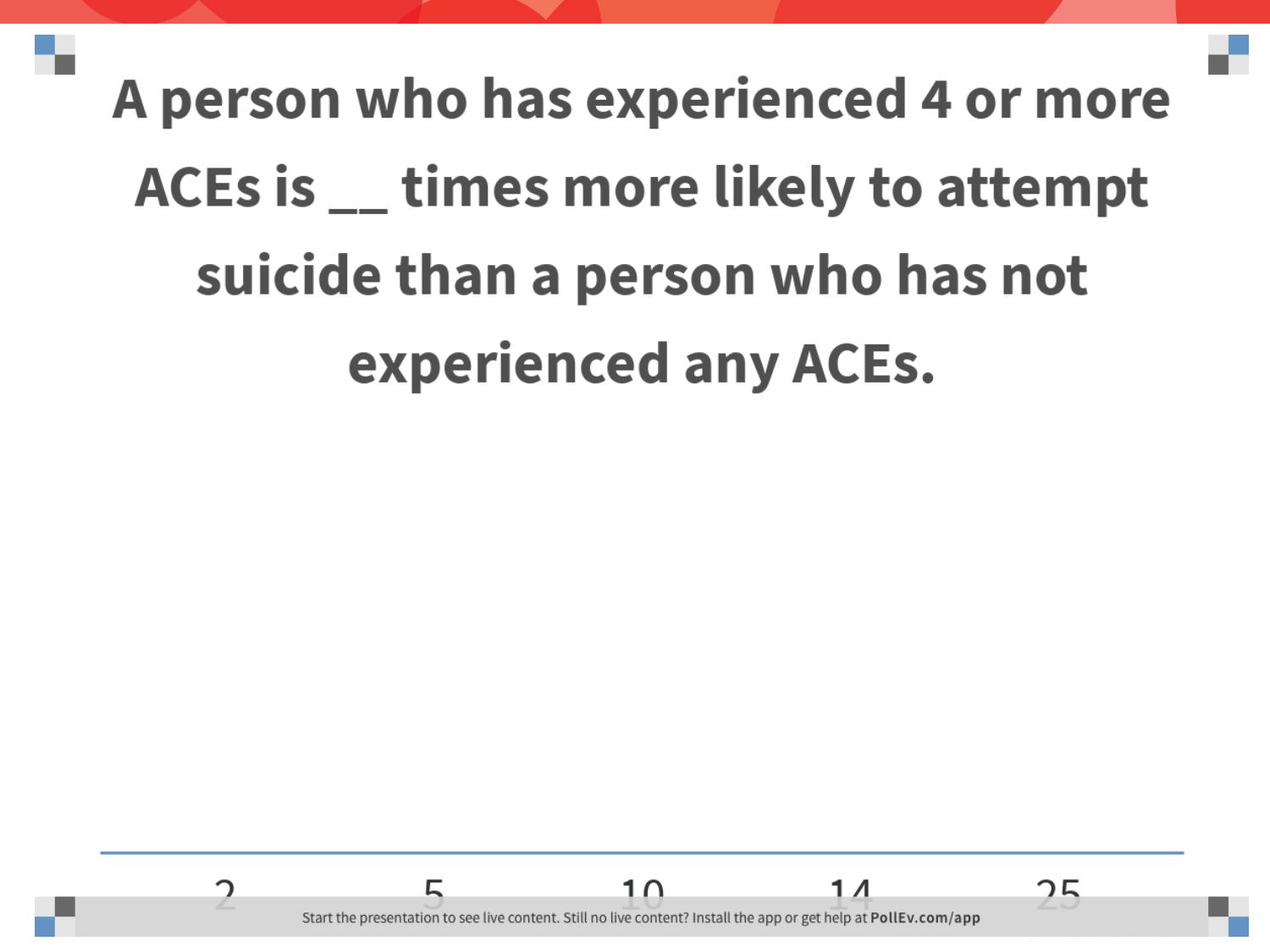

August 31, 2020

## Slide 7
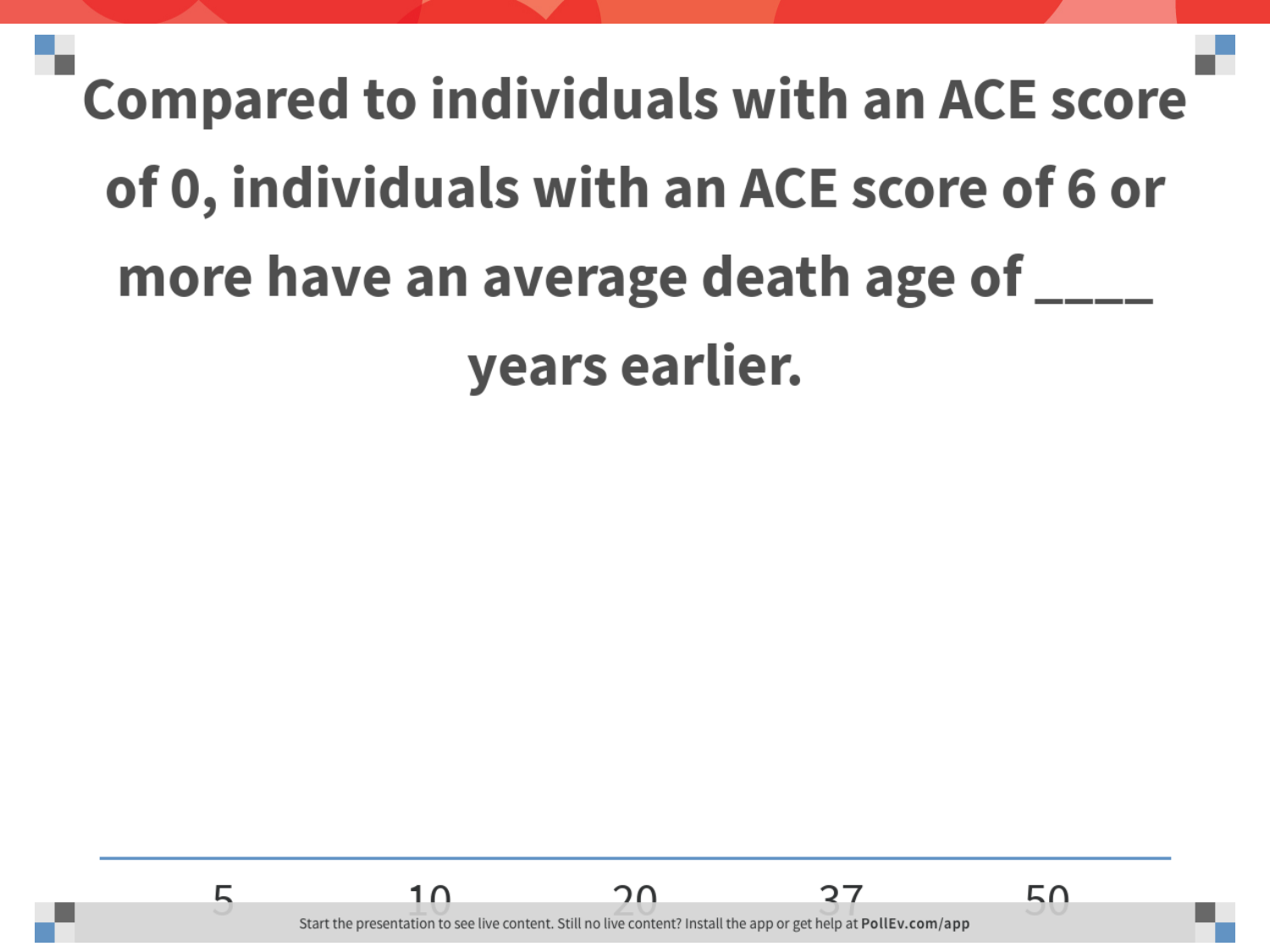

August 31, 2020

## Slide 8
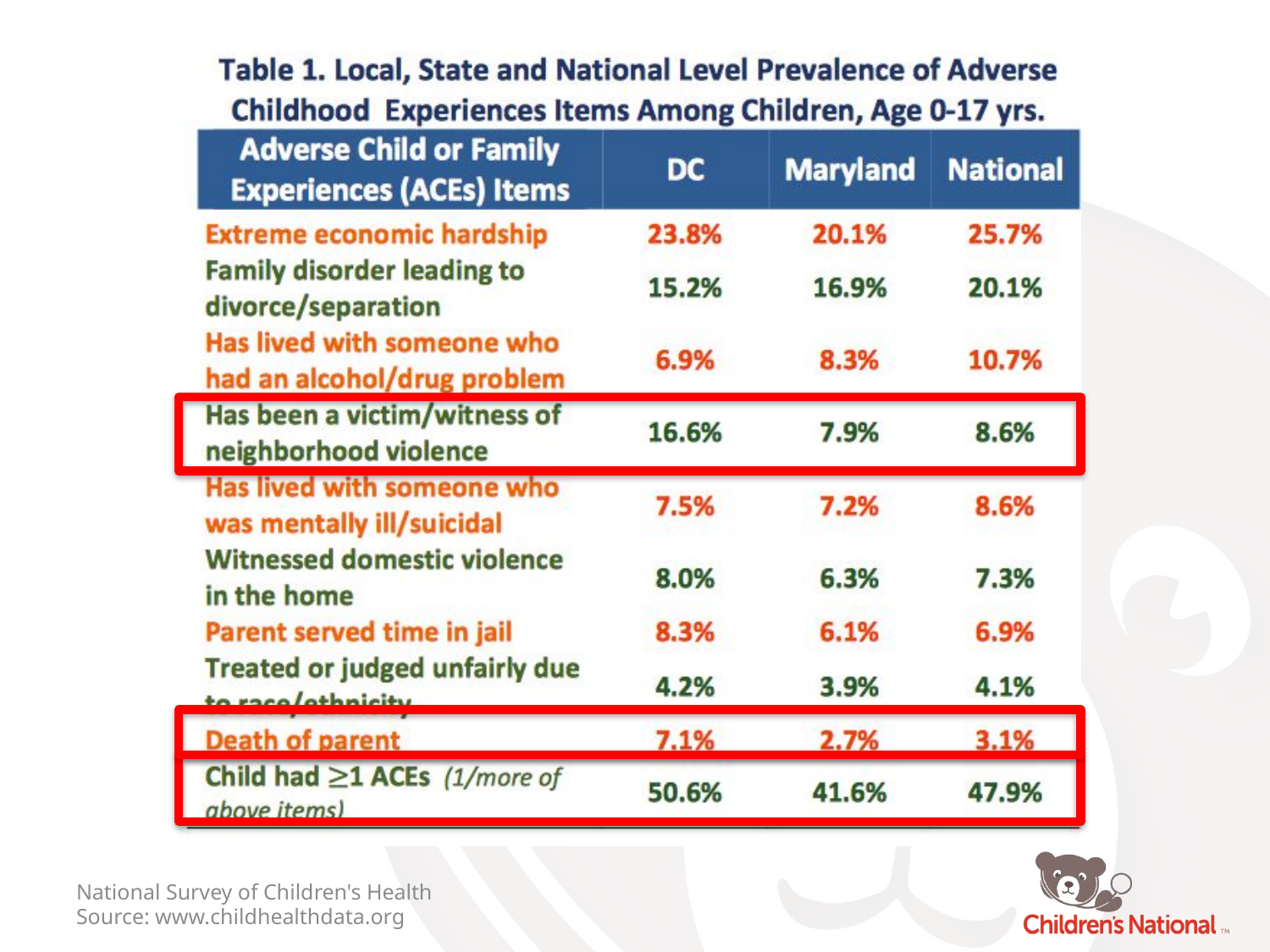

National Survey of Children's Health
Source: www.childhealthdata.org

## Slide 9
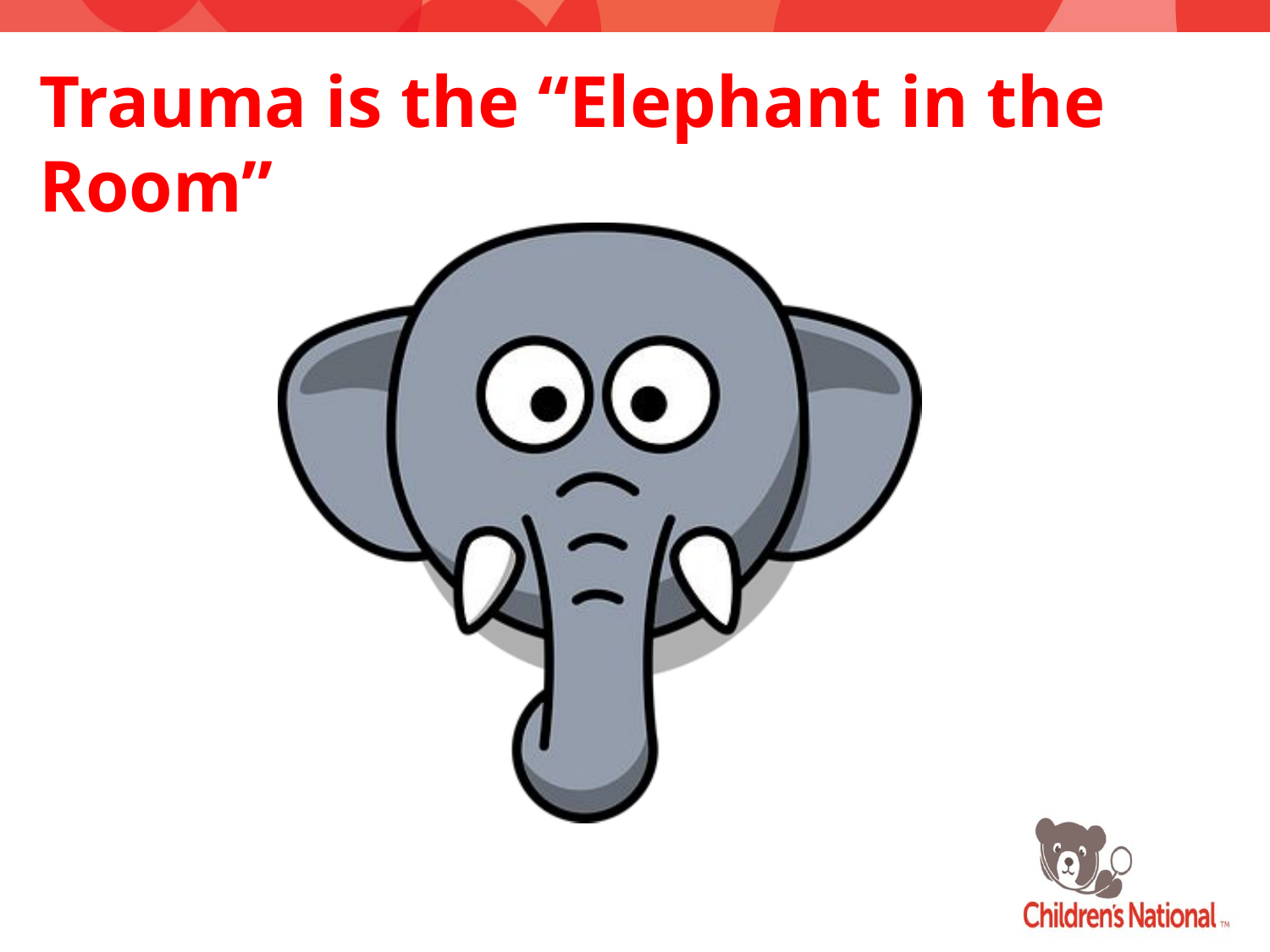

# Trauma is the “Elephant in the Room”

## Slide 10
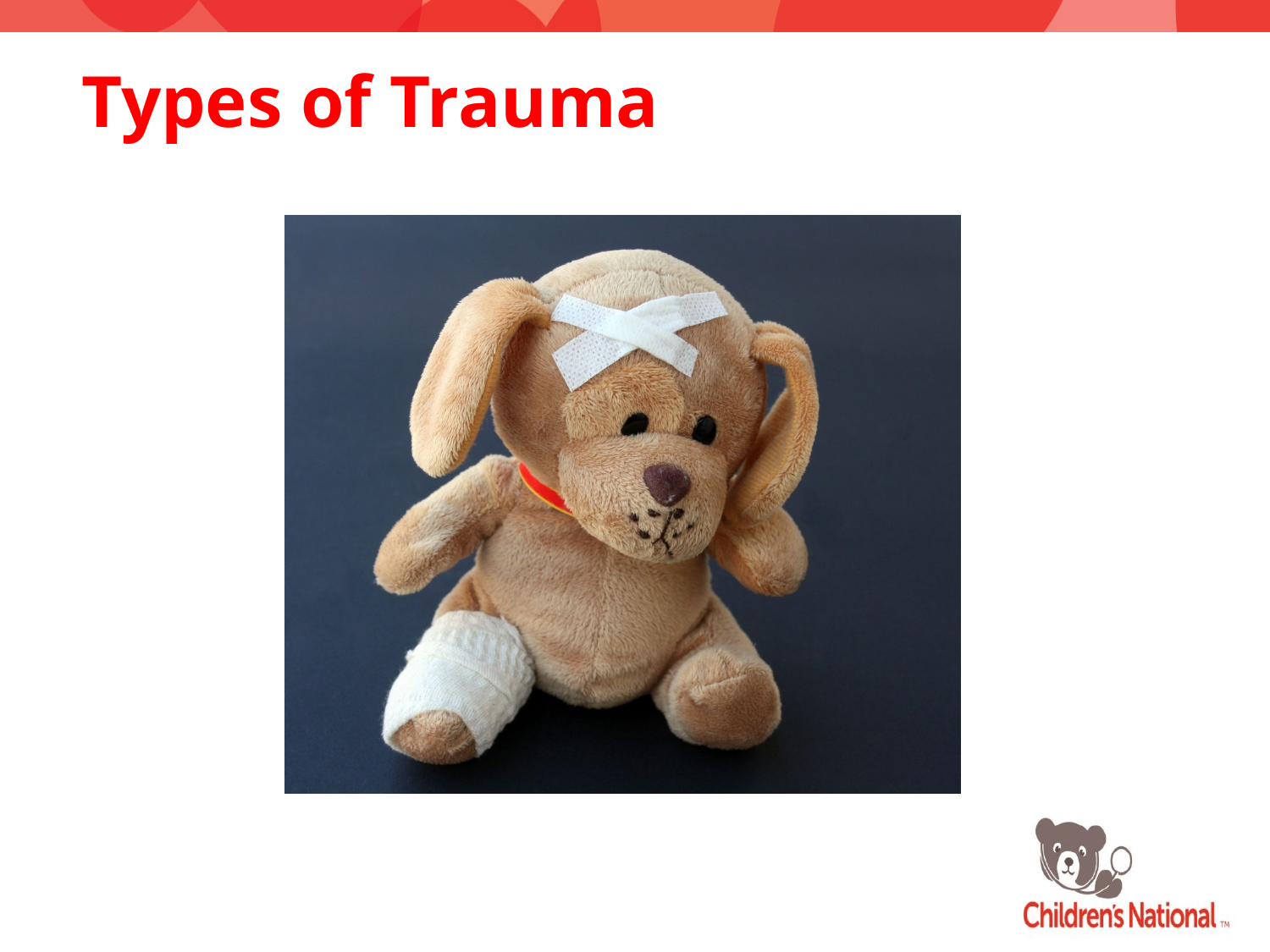

# Types of Trauma

## Slide 11
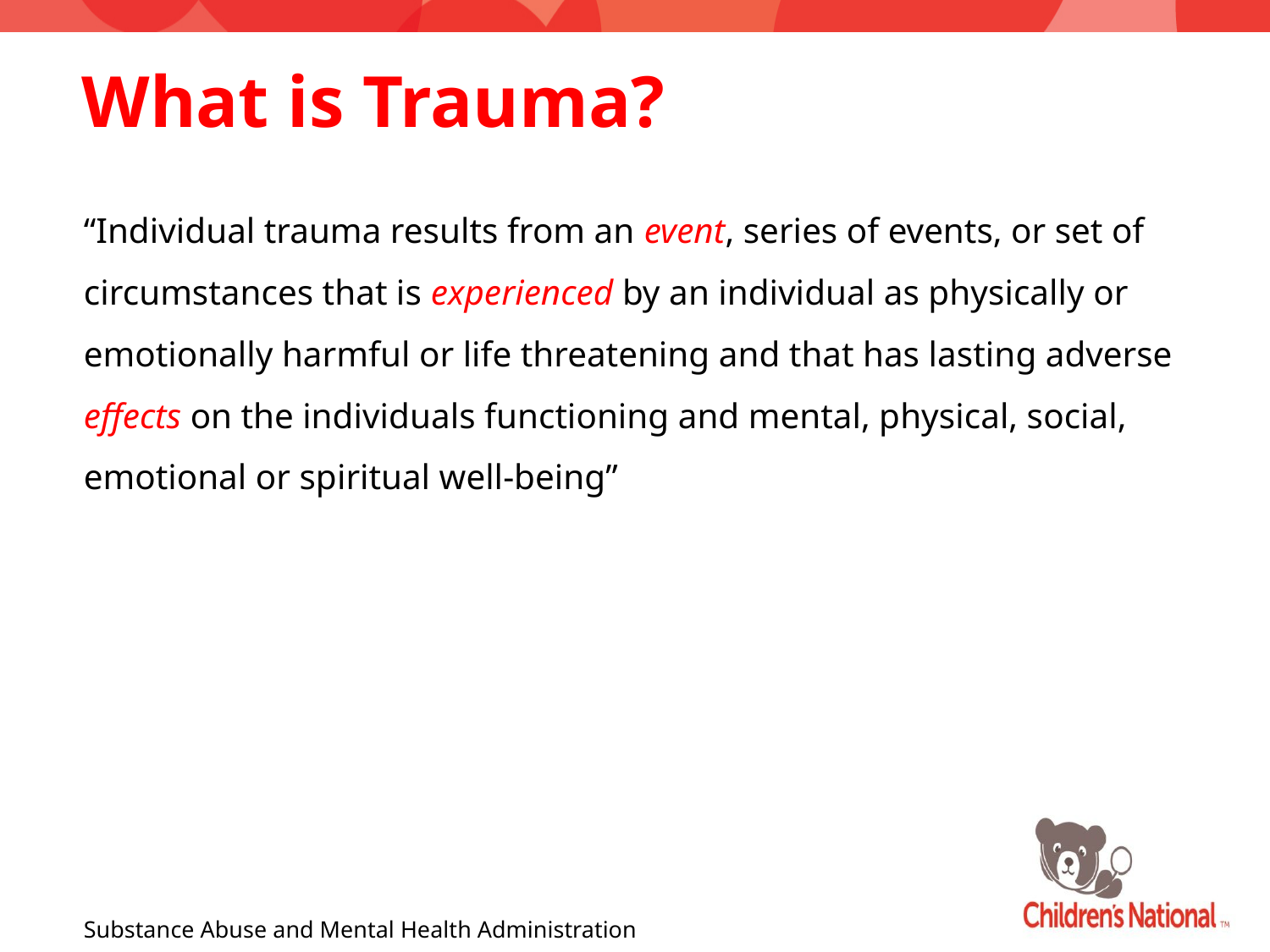

# What is Trauma?
“Individual trauma results from an event, series of events, or set of circumstances that is experienced by an individual as physically or emotionally harmful or life threatening and that has lasting adverse effects on the individuals functioning and mental, physical, social, emotional or spiritual well-being”
Substance Abuse and Mental Health Administration

## Slide 12
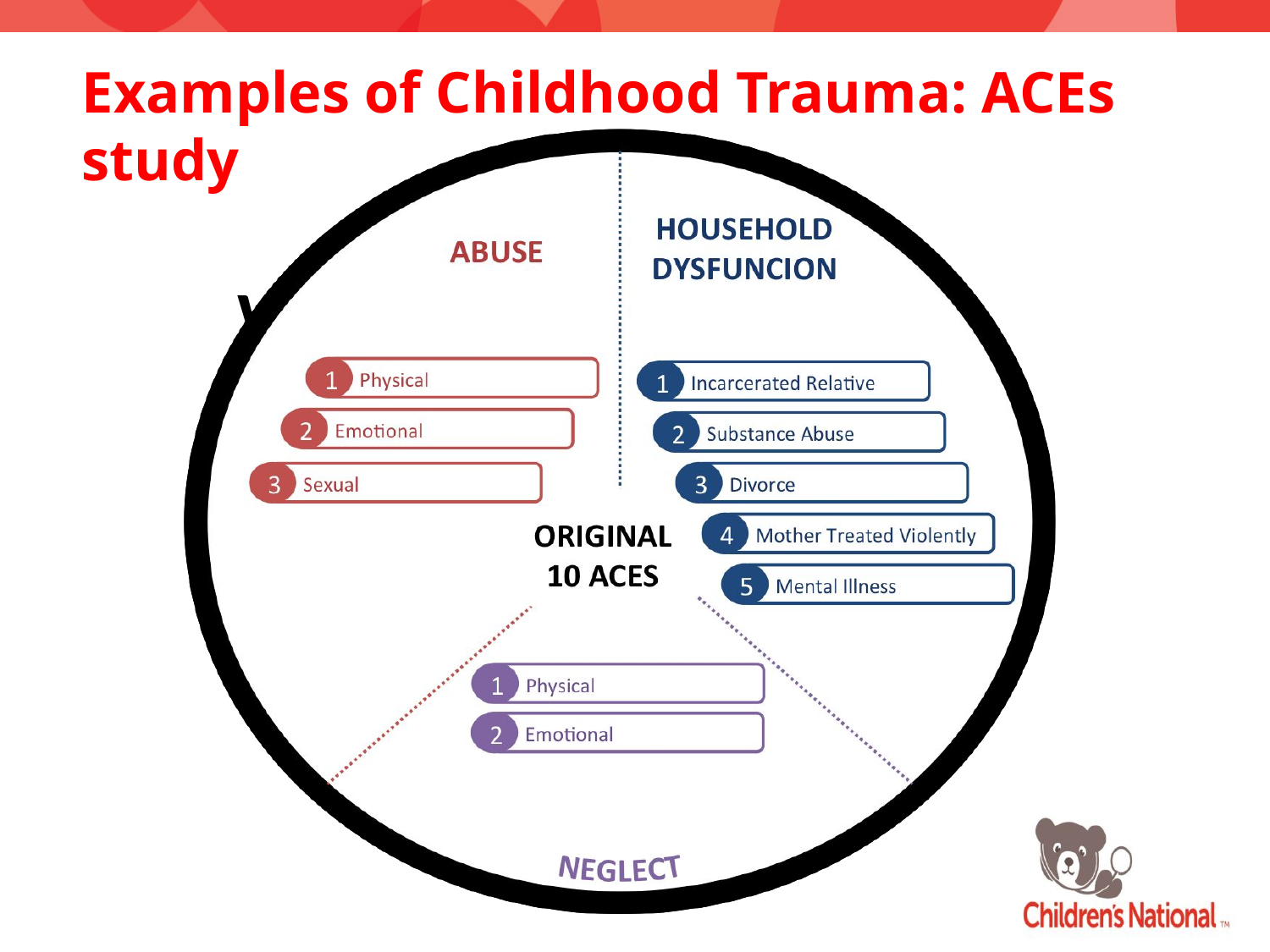

# Examples of Childhood Trauma: ACEs study
What are the 10 Adverse Childhood Experiences in the Original ACEs study?

## Slide 13
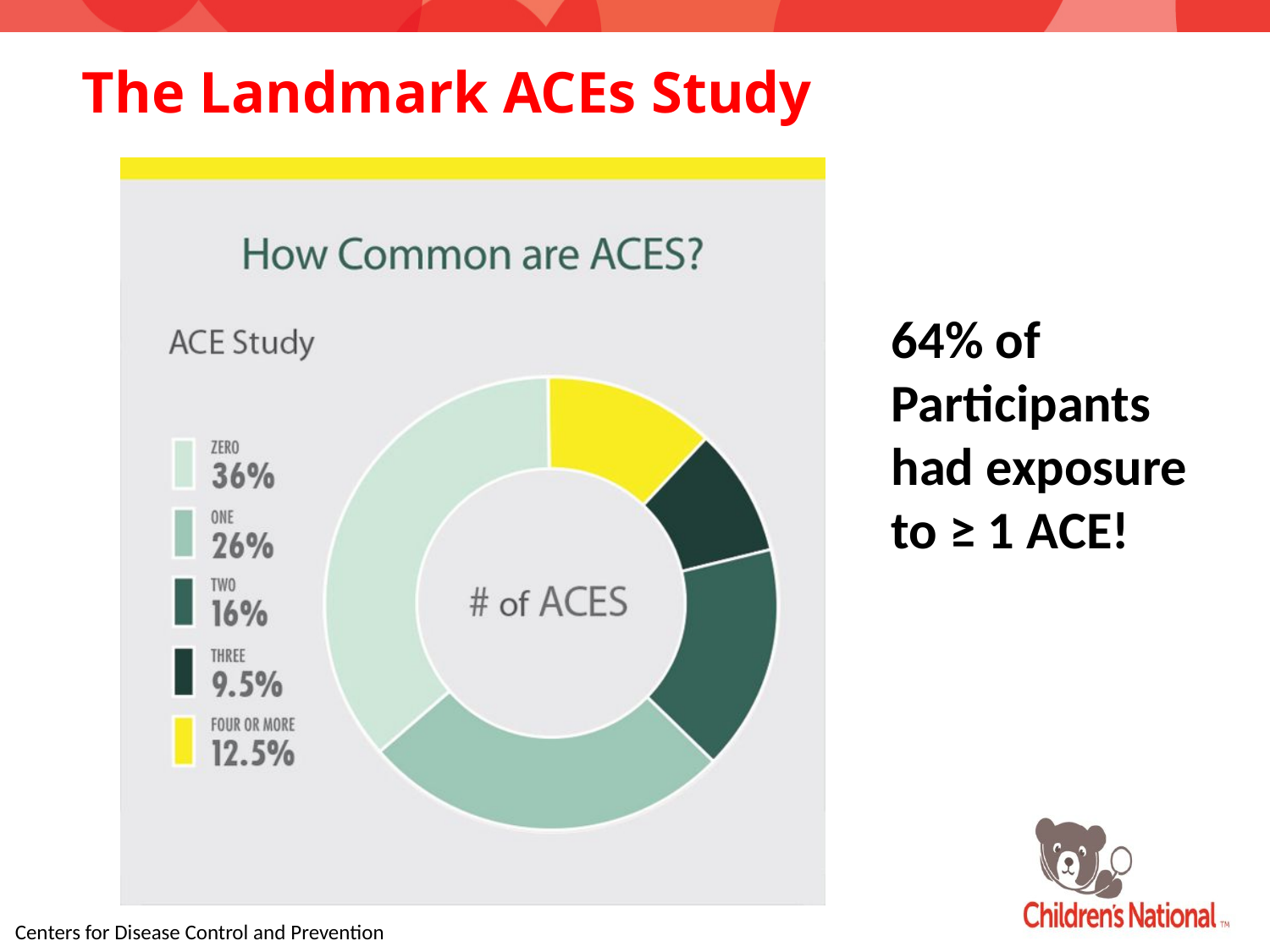

# The Landmark ACEs Study
64% of Participants had exposure to ≥ 1 ACE!
Centers for Disease Control and Prevention

## Slide 14
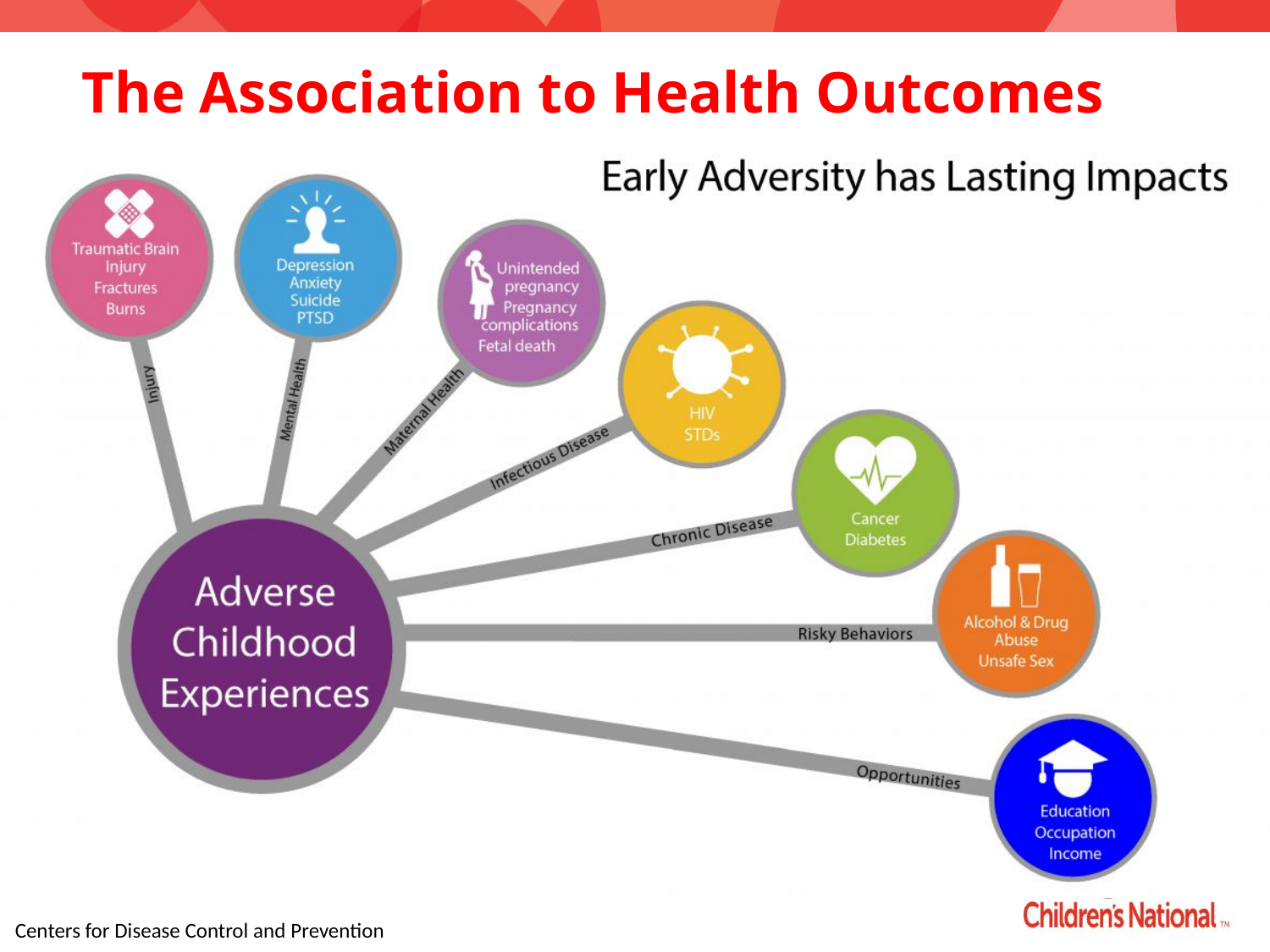

# The Association to Health Outcomes
Centers for Disease Control and Prevention

## Slide 15
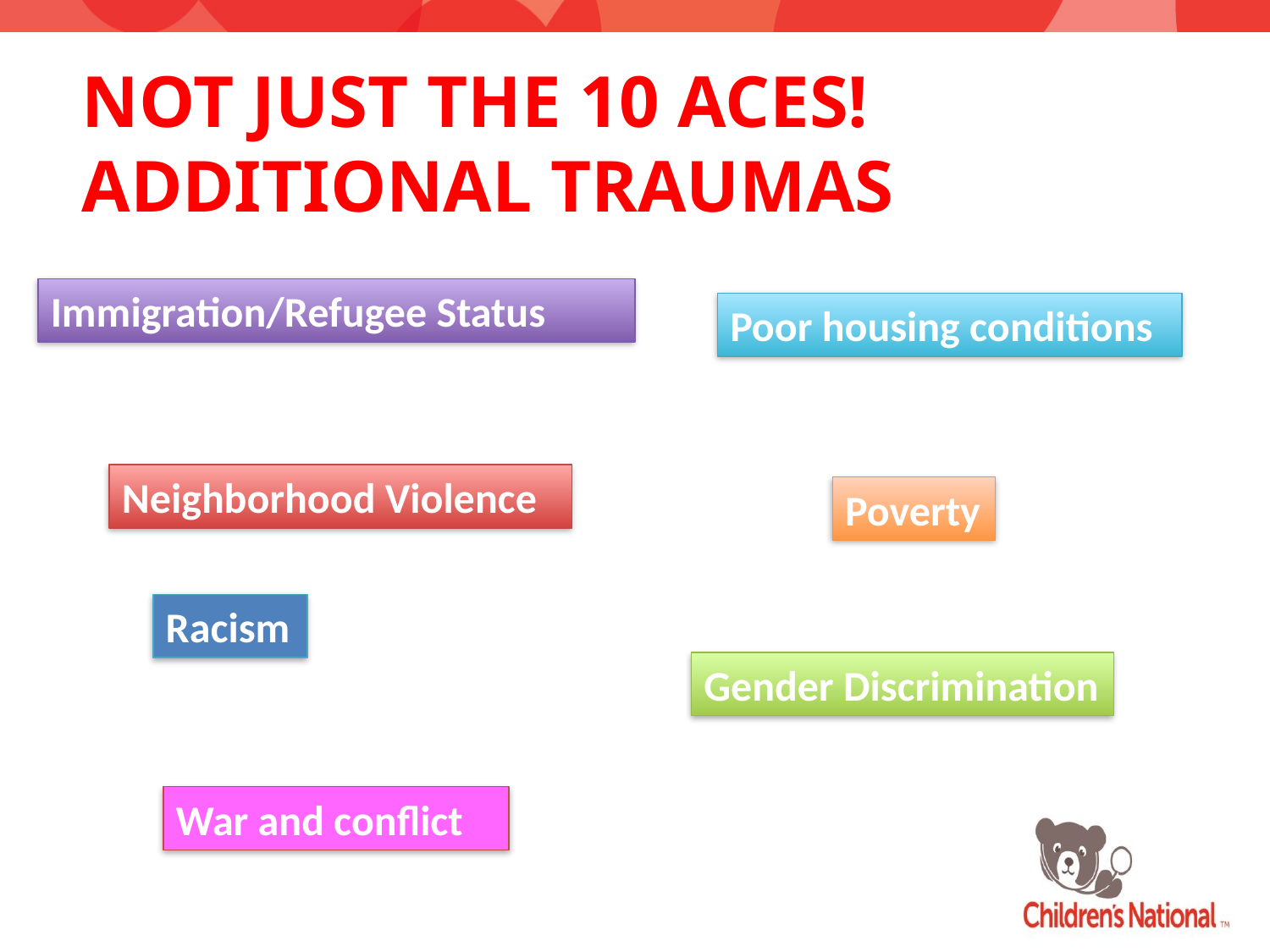

# Not just the 10 ACEs! Additional traumas
Immigration/Refugee Status
Poor housing conditions
Neighborhood Violence
Poverty
Racism
Gender Discrimination
War and conflict

## Slide 16
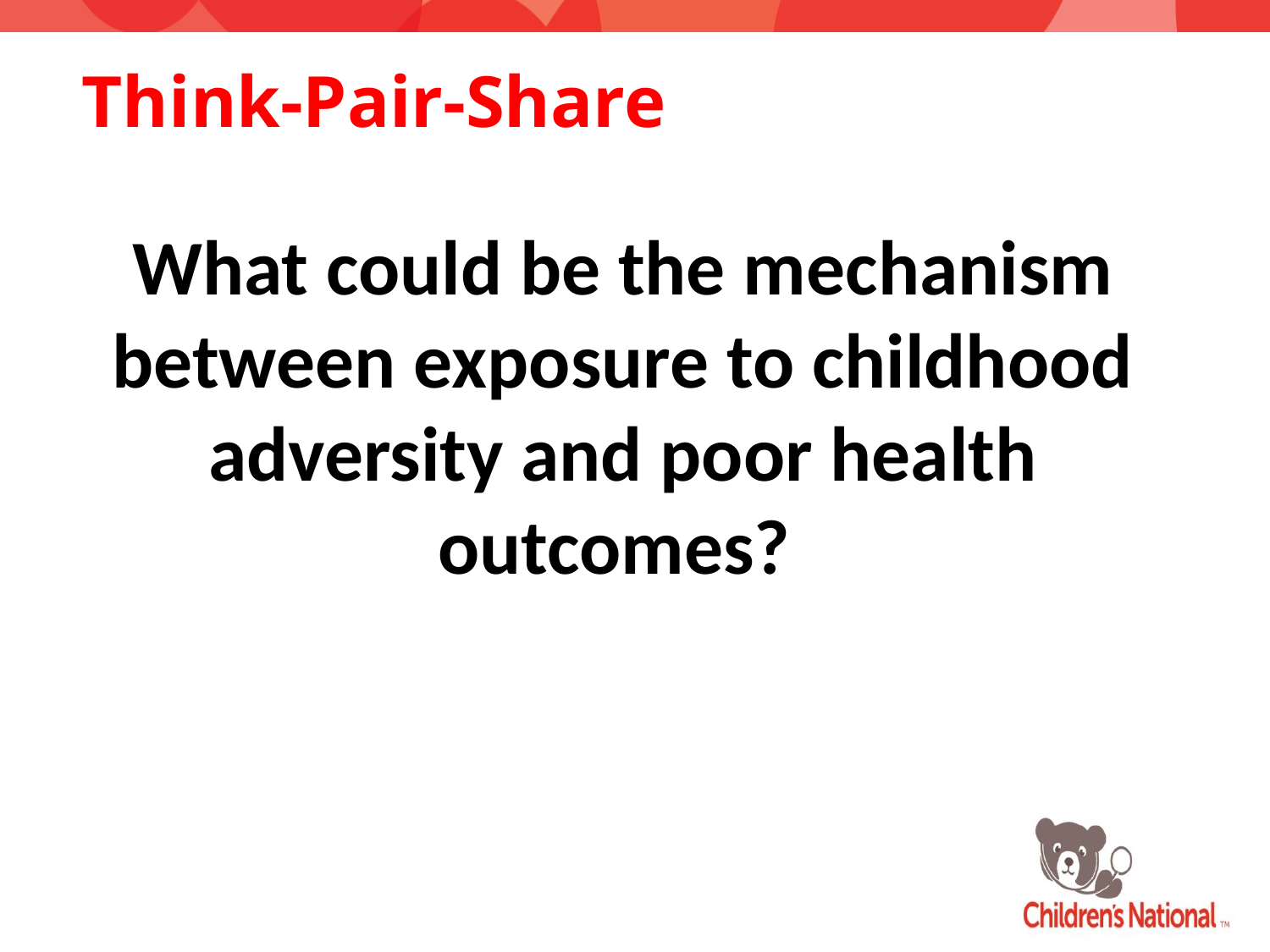

# Think-Pair-Share
What could be the mechanism between exposure to childhood adversity and poor health outcomes?

## Slide 17
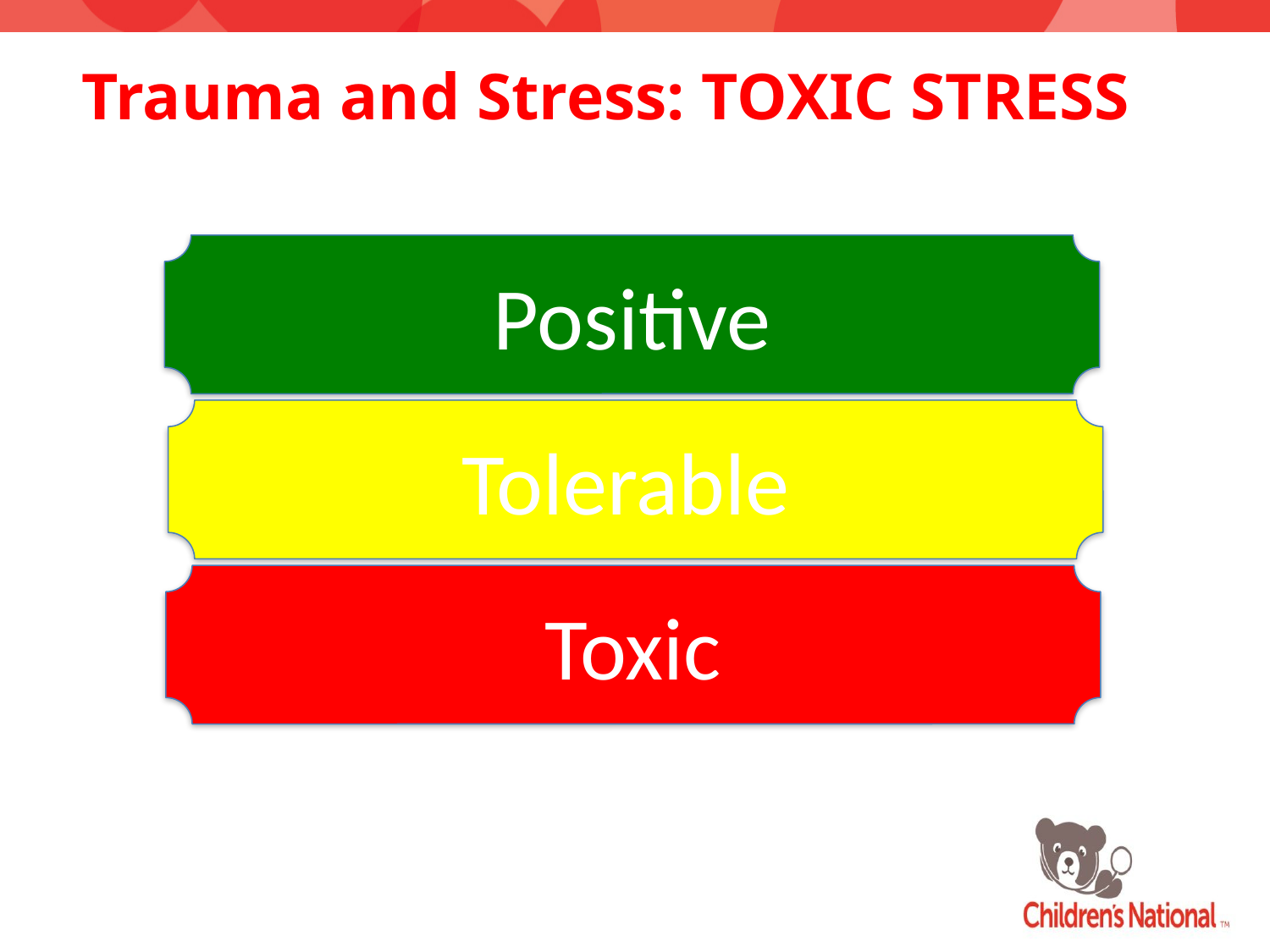

# Trauma and Stress: TOXIC STRESS
Positive
Tolerable
Toxic

## Slide 18
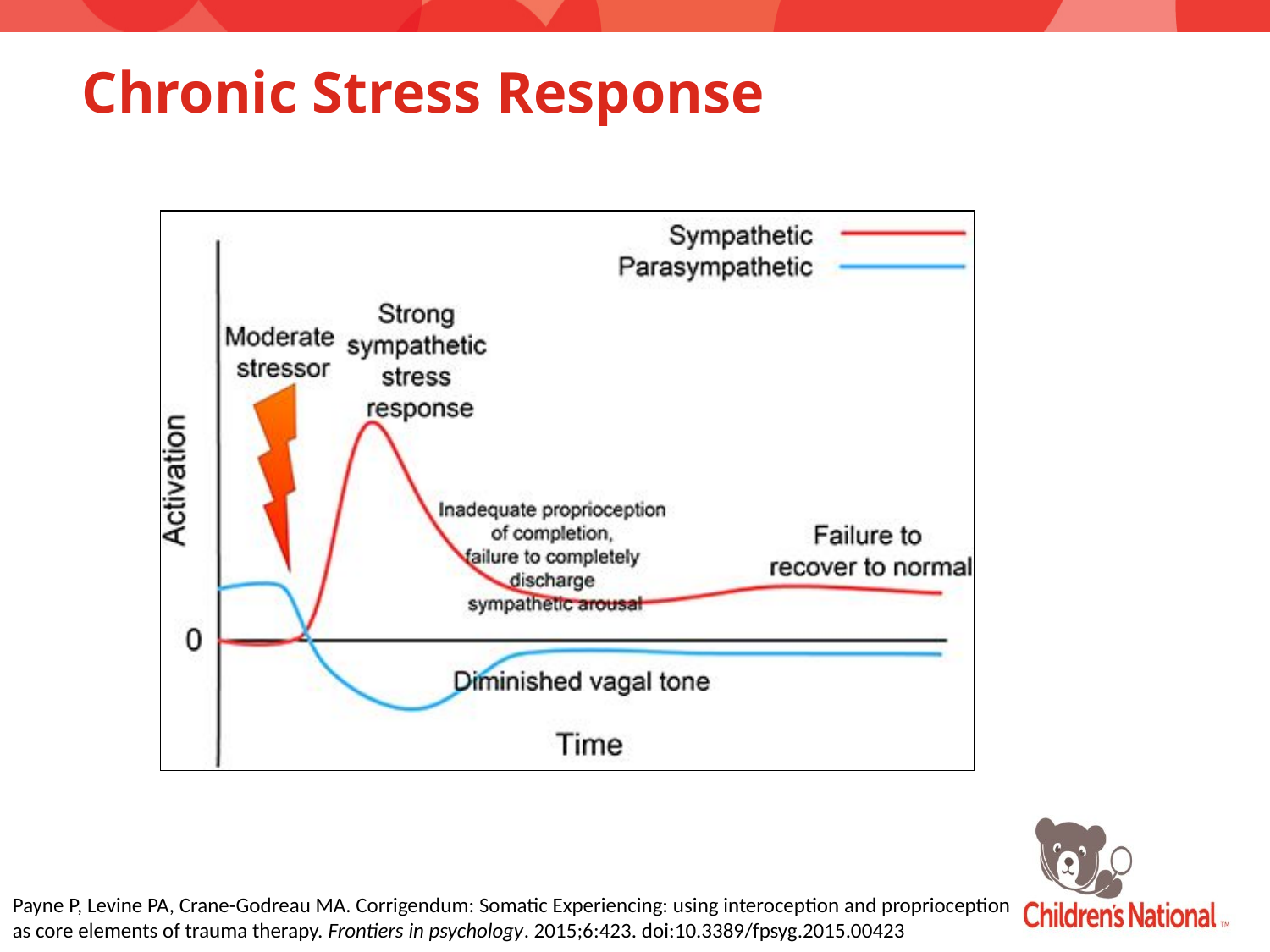

# Chronic Stress Response
Payne P, Levine PA, Crane-Godreau MA. Corrigendum: Somatic Experiencing: using interoception and proprioception as core elements of trauma therapy. Frontiers in psychology. 2015;6:423. doi:10.3389/fpsyg.2015.00423

## Slide 19
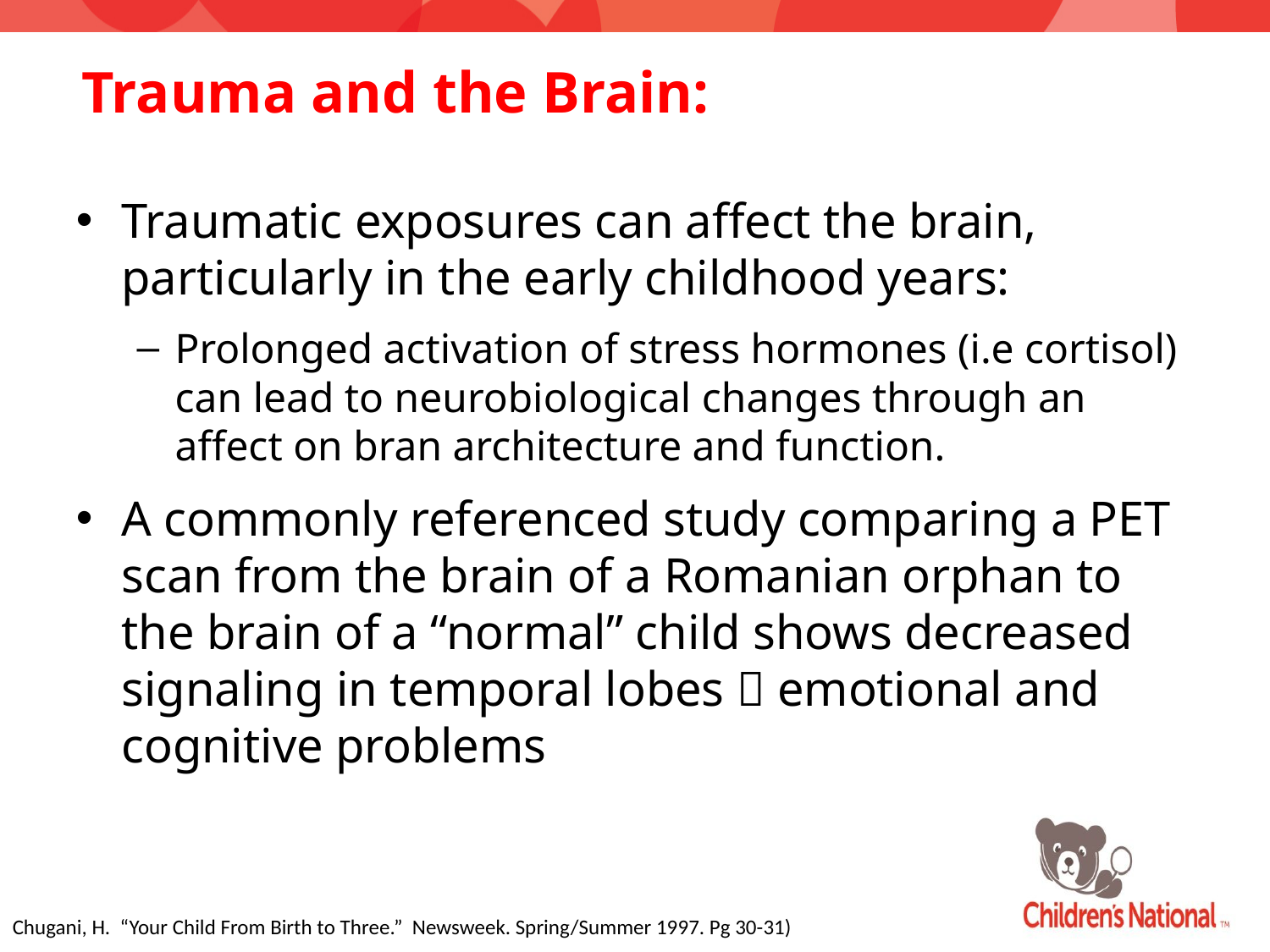

# Trauma and the Brain:
Traumatic exposures can affect the brain, particularly in the early childhood years:
Prolonged activation of stress hormones (i.e cortisol) can lead to neurobiological changes through an affect on bran architecture and function.
A commonly referenced study comparing a PET scan from the brain of a Romanian orphan to the brain of a “normal” child shows decreased signaling in temporal lobes  emotional and cognitive problems
Chugani, H. “Your Child From Birth to Three.” Newsweek. Spring/Summer 1997. Pg 30-31)

## Slide 20
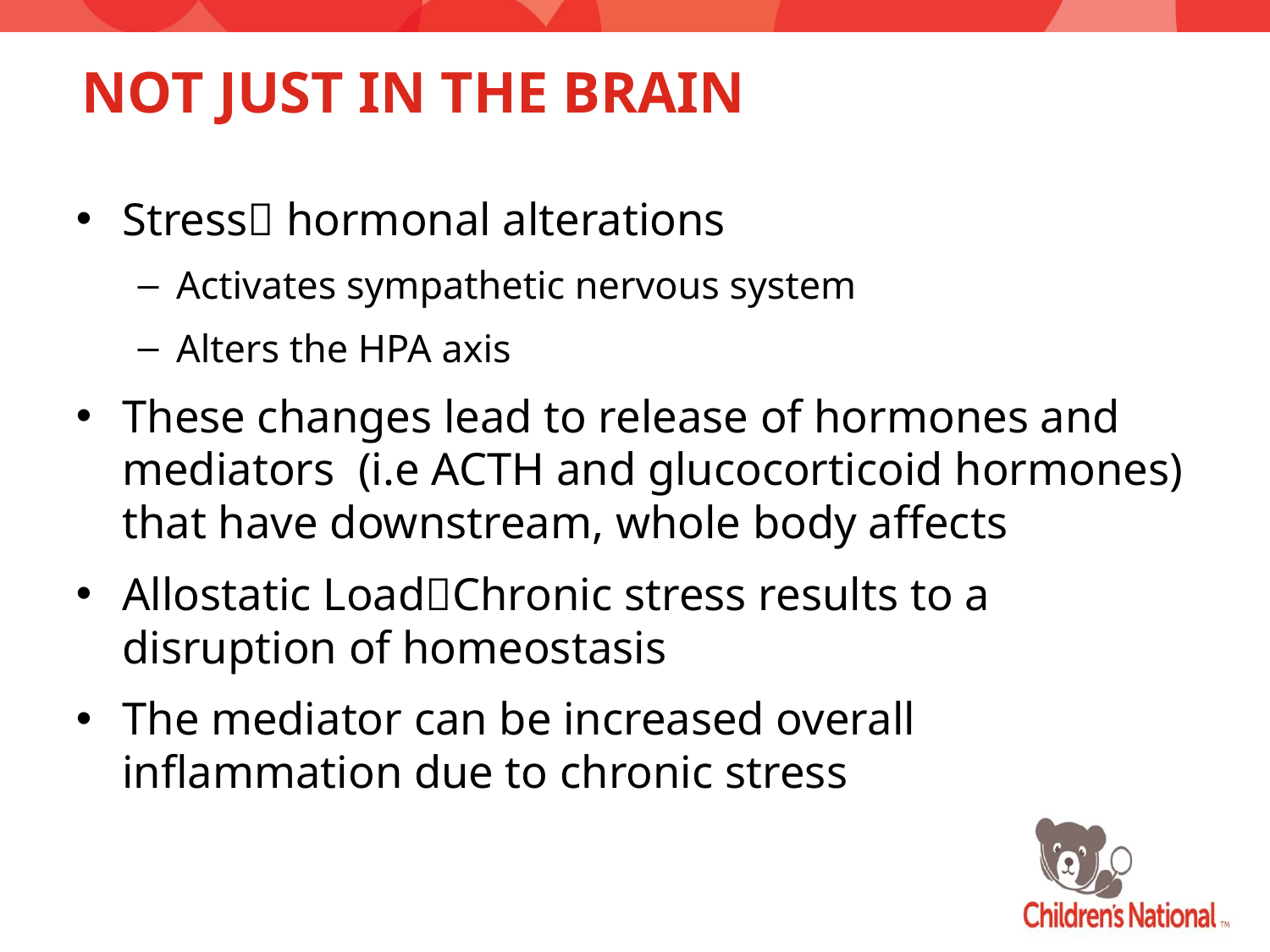

# NOT JUST IN THE BRAIN
Stress hormonal alterations
Activates sympathetic nervous system
Alters the HPA axis
These changes lead to release of hormones and mediators (i.e ACTH and glucocorticoid hormones) that have downstream, whole body affects
Allostatic LoadChronic stress results to a disruption of homeostasis
The mediator can be increased overall inflammation due to chronic stress

## Slide 21
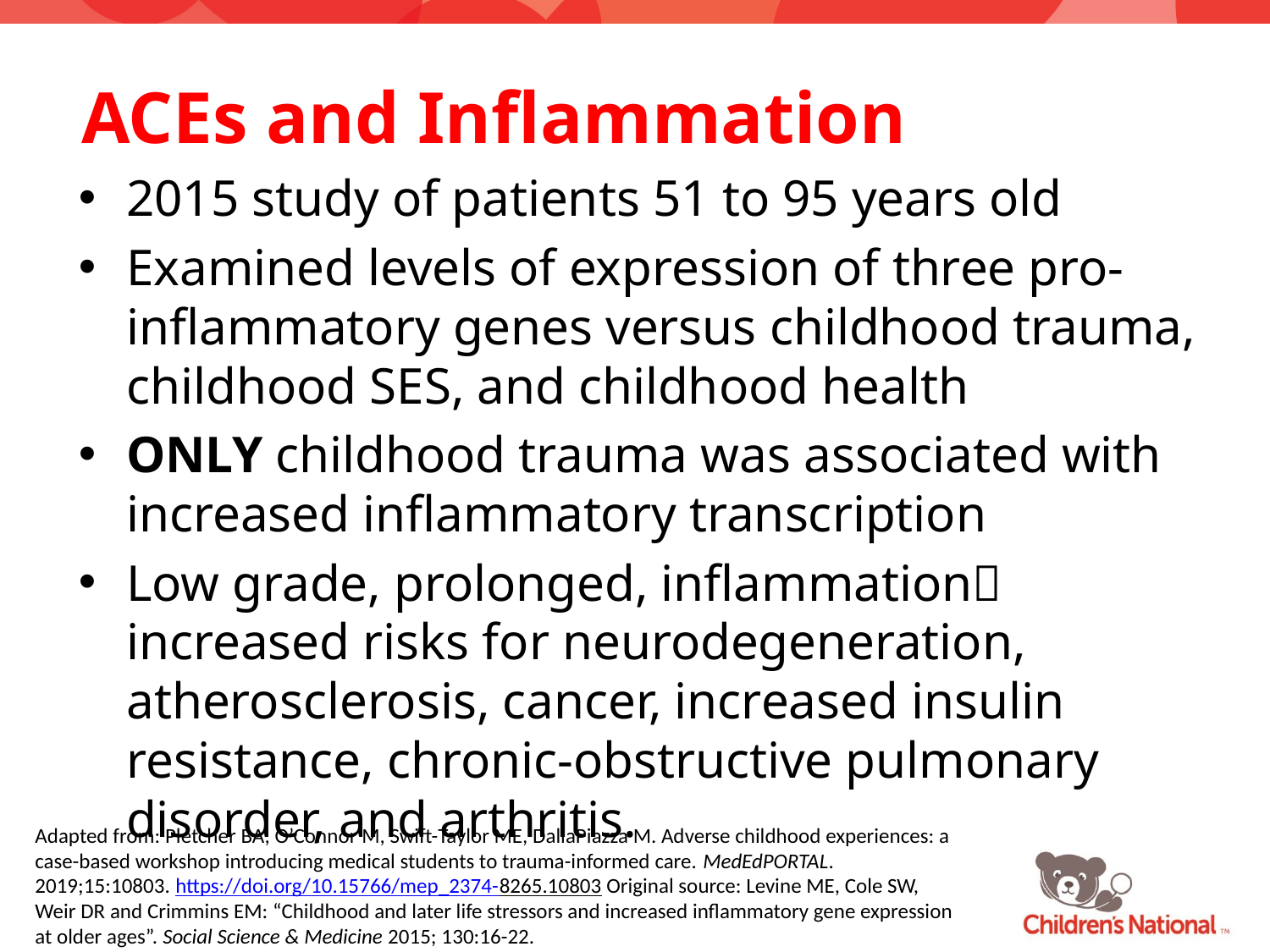

# ACEs and Inflammation
2015 study of patients 51 to 95 years old
Examined levels of expression of three pro-inflammatory genes versus childhood trauma, childhood SES, and childhood health
ONLY childhood trauma was associated with increased inflammatory transcription
Low grade, prolonged, inflammation increased risks for neurodegeneration, atherosclerosis, cancer, increased insulin resistance, chronic-obstructive pulmonary disorder, and arthritis.
Adapted from: Pletcher BA, O’Connor M, Swift-Taylor ME, DallaPiazza M. Adverse childhood experiences: a case-based workshop introducing medical students to trauma-informed care. MedEdPORTAL. 2019;15:10803. https://doi.org/10.15766/mep_2374-8265.10803 Original source: Levine ME, Cole SW, Weir DR and Crimmins EM: “Childhood and later life stressors and increased inflammatory gene expression at older ages”. Social Science & Medicine 2015; 130:16-22.

## Slide 22
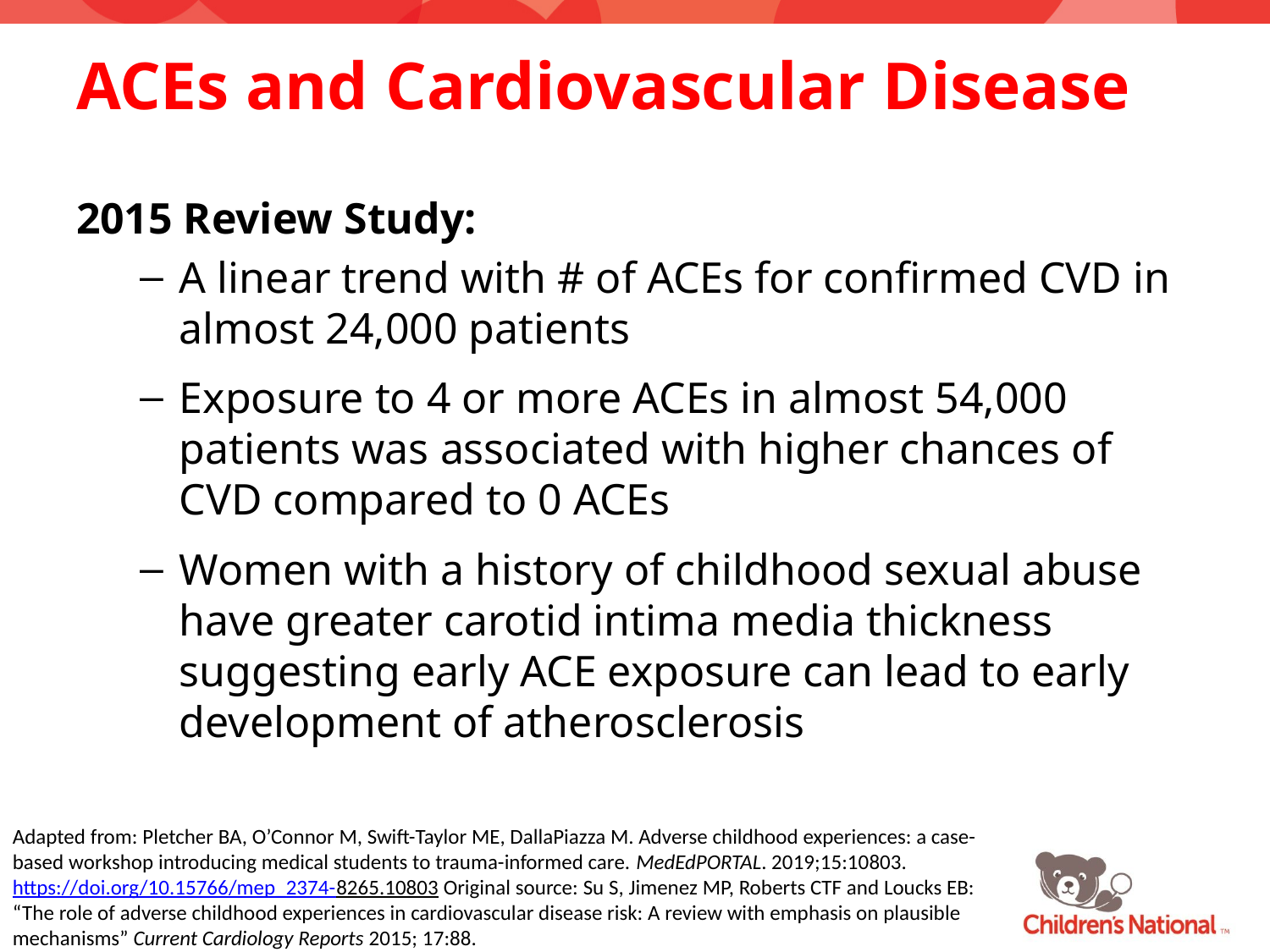

# ACEs and Cardiovascular Disease
2015 Review Study:
A linear trend with # of ACEs for confirmed CVD in almost 24,000 patients
Exposure to 4 or more ACEs in almost 54,000 patients was associated with higher chances of CVD compared to 0 ACEs
Women with a history of childhood sexual abuse have greater carotid intima media thickness suggesting early ACE exposure can lead to early development of atherosclerosis
Adapted from: Pletcher BA, O’Connor M, Swift-Taylor ME, DallaPiazza M. Adverse childhood experiences: a case-based workshop introducing medical students to trauma-informed care. MedEdPORTAL. 2019;15:10803. https://doi.org/10.15766/mep_2374-8265.10803 Original source: Su S, Jimenez MP, Roberts CTF and Loucks EB: “The role of adverse childhood experiences in cardiovascular disease risk: A review with emphasis on plausible mechanisms” Current Cardiology Reports 2015; 17:88.

## Slide 23
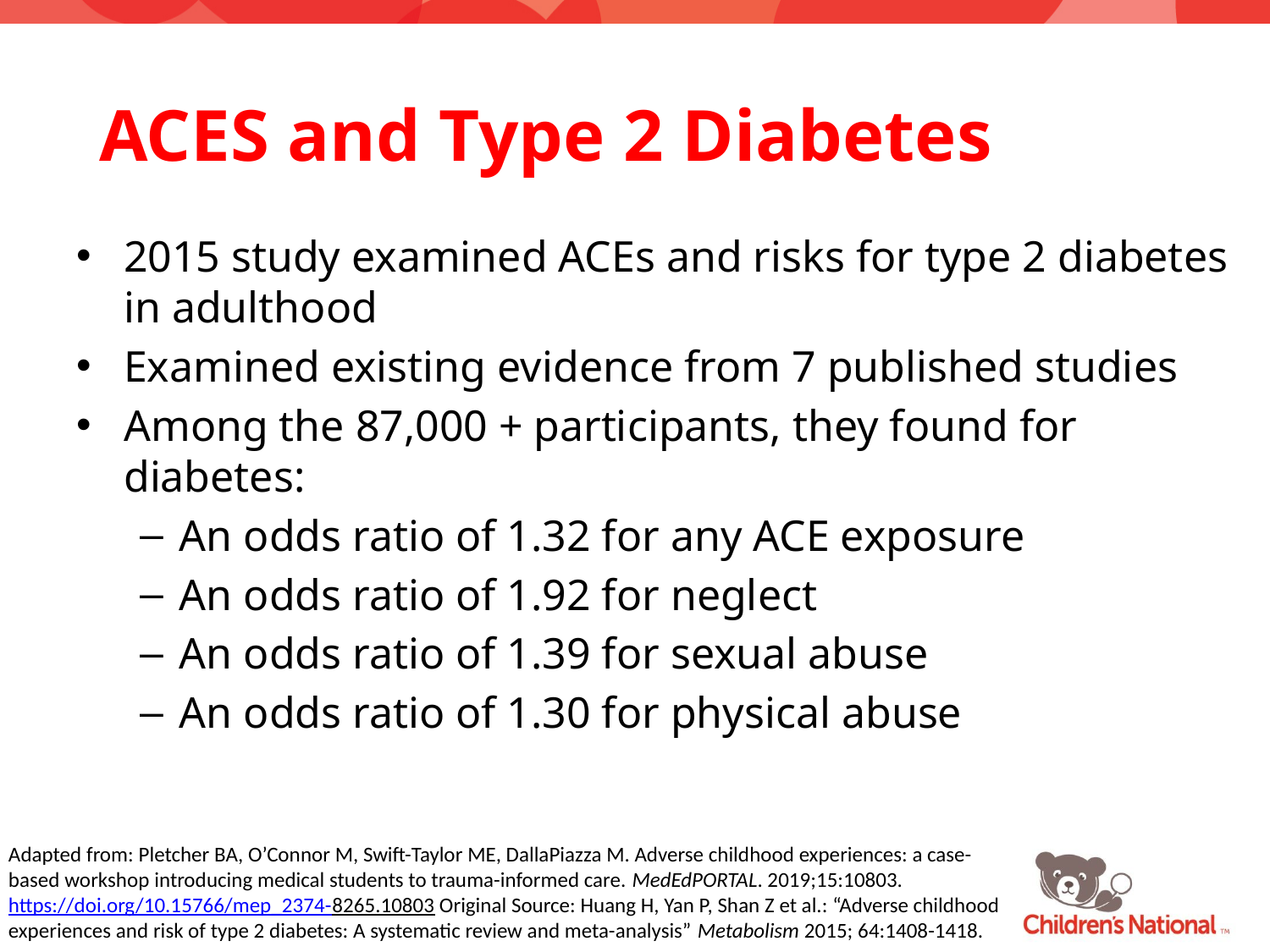

# ACES and Type 2 Diabetes
2015 study examined ACEs and risks for type 2 diabetes in adulthood
Examined existing evidence from 7 published studies
Among the 87,000 + participants, they found for diabetes:
An odds ratio of 1.32 for any ACE exposure
An odds ratio of 1.92 for neglect
An odds ratio of 1.39 for sexual abuse
An odds ratio of 1.30 for physical abuse
Adapted from: Pletcher BA, O’Connor M, Swift-Taylor ME, DallaPiazza M. Adverse childhood experiences: a case-based workshop introducing medical students to trauma-informed care. MedEdPORTAL. 2019;15:10803. https://doi.org/10.15766/mep_2374-8265.10803 Original Source: Huang H, Yan P, Shan Z et al.: “Adverse childhood experiences and risk of type 2 diabetes: A systematic review and meta-analysis” Metabolism 2015; 64:1408-1418.

## Slide 24
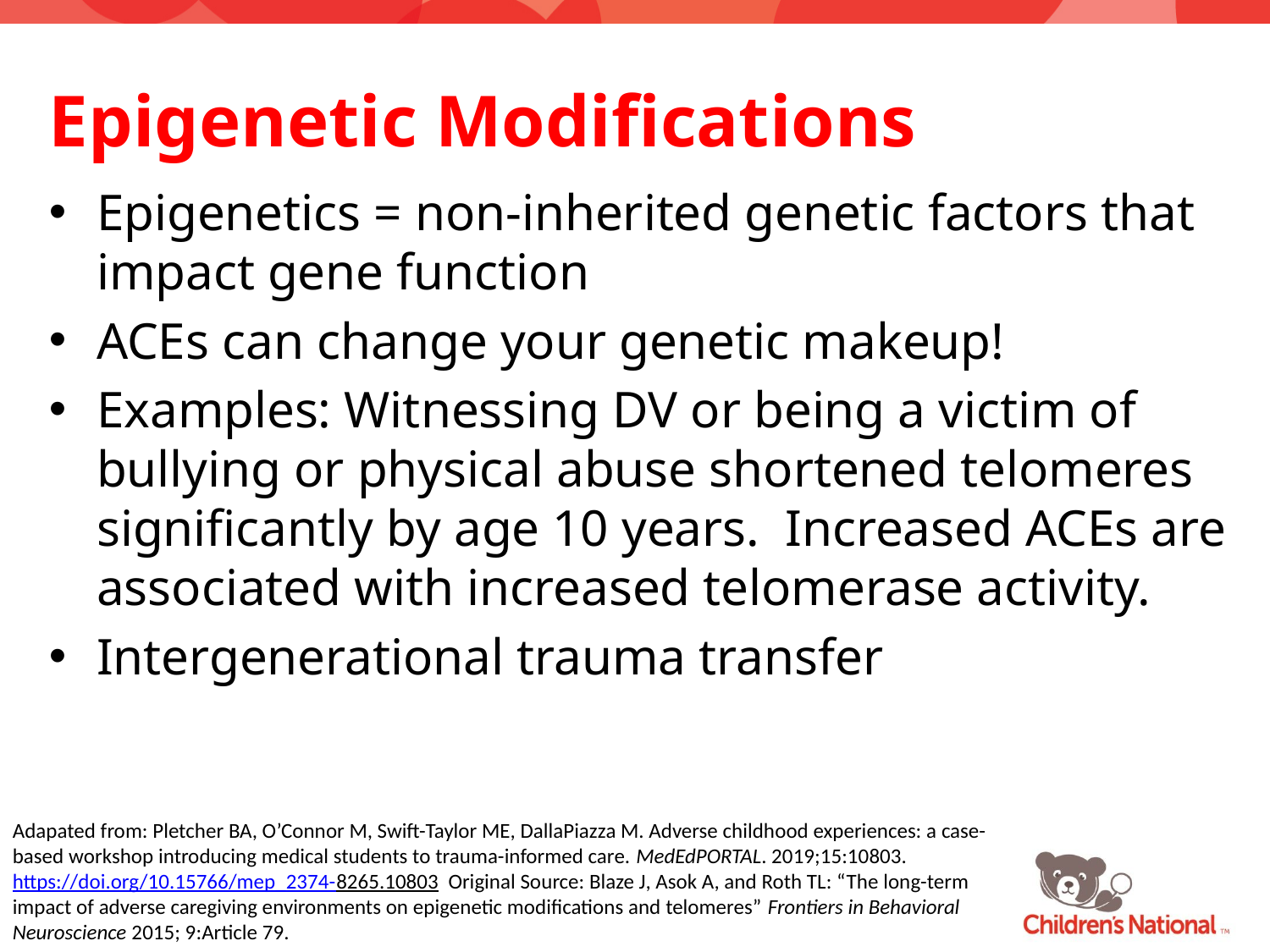

# Epigenetic Modifications
Epigenetics = non-inherited genetic factors that impact gene function
ACEs can change your genetic makeup!
Examples: Witnessing DV or being a victim of bullying or physical abuse shortened telomeres significantly by age 10 years. Increased ACEs are associated with increased telomerase activity.
Intergenerational trauma transfer
Adapated from: Pletcher BA, O’Connor M, Swift-Taylor ME, DallaPiazza M. Adverse childhood experiences: a case-based workshop introducing medical students to trauma-informed care. MedEdPORTAL. 2019;15:10803. https://doi.org/10.15766/mep_2374-8265.10803 Original Source: Blaze J, Asok A, and Roth TL: “The long-term impact of adverse caregiving environments on epigenetic modifications and telomeres” Frontiers in Behavioral Neuroscience 2015; 9:Article 79.

## Slide 25
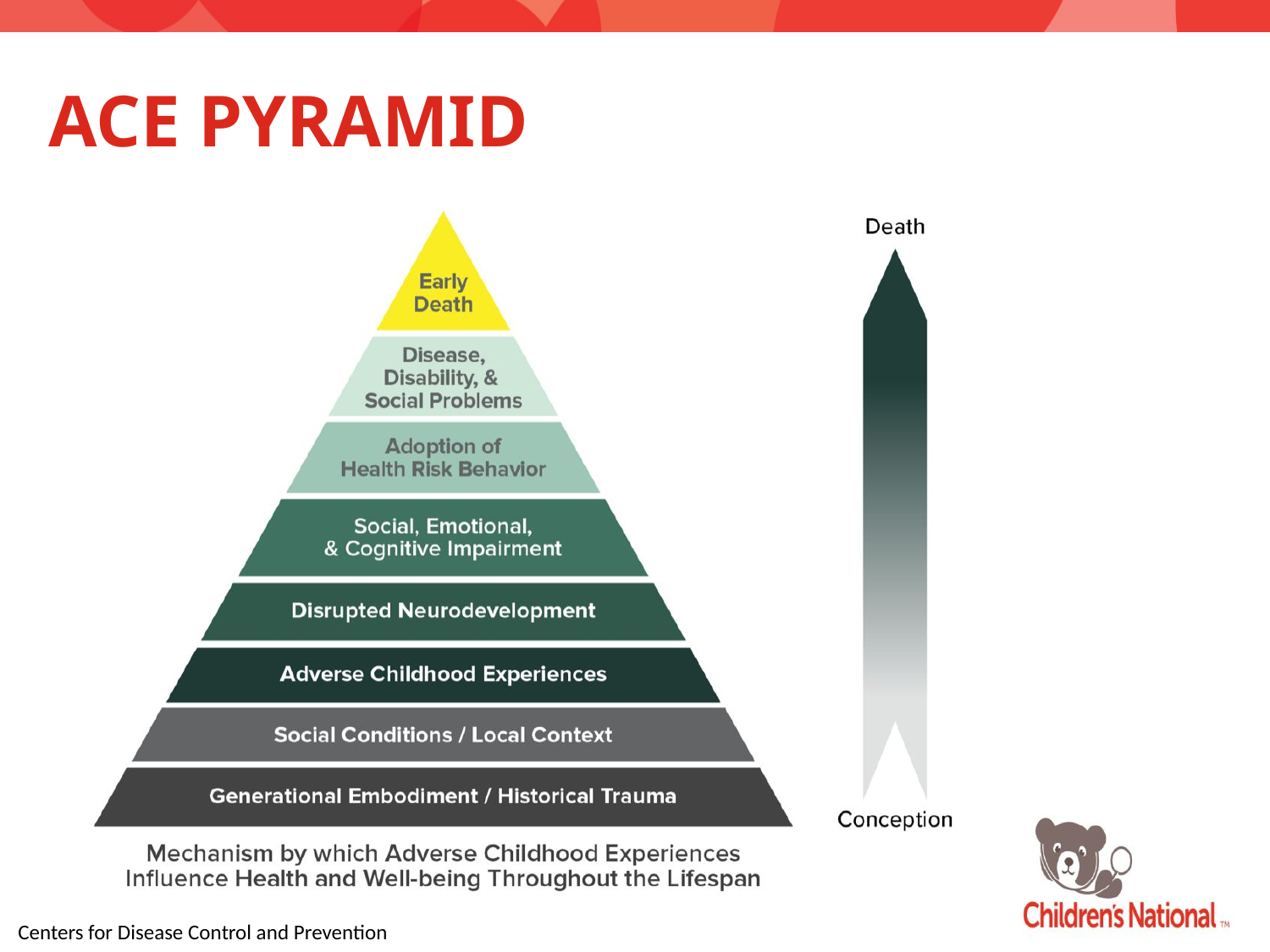

# ACE PYRAMID
Centers for Disease Control and Prevention

## Slide 26
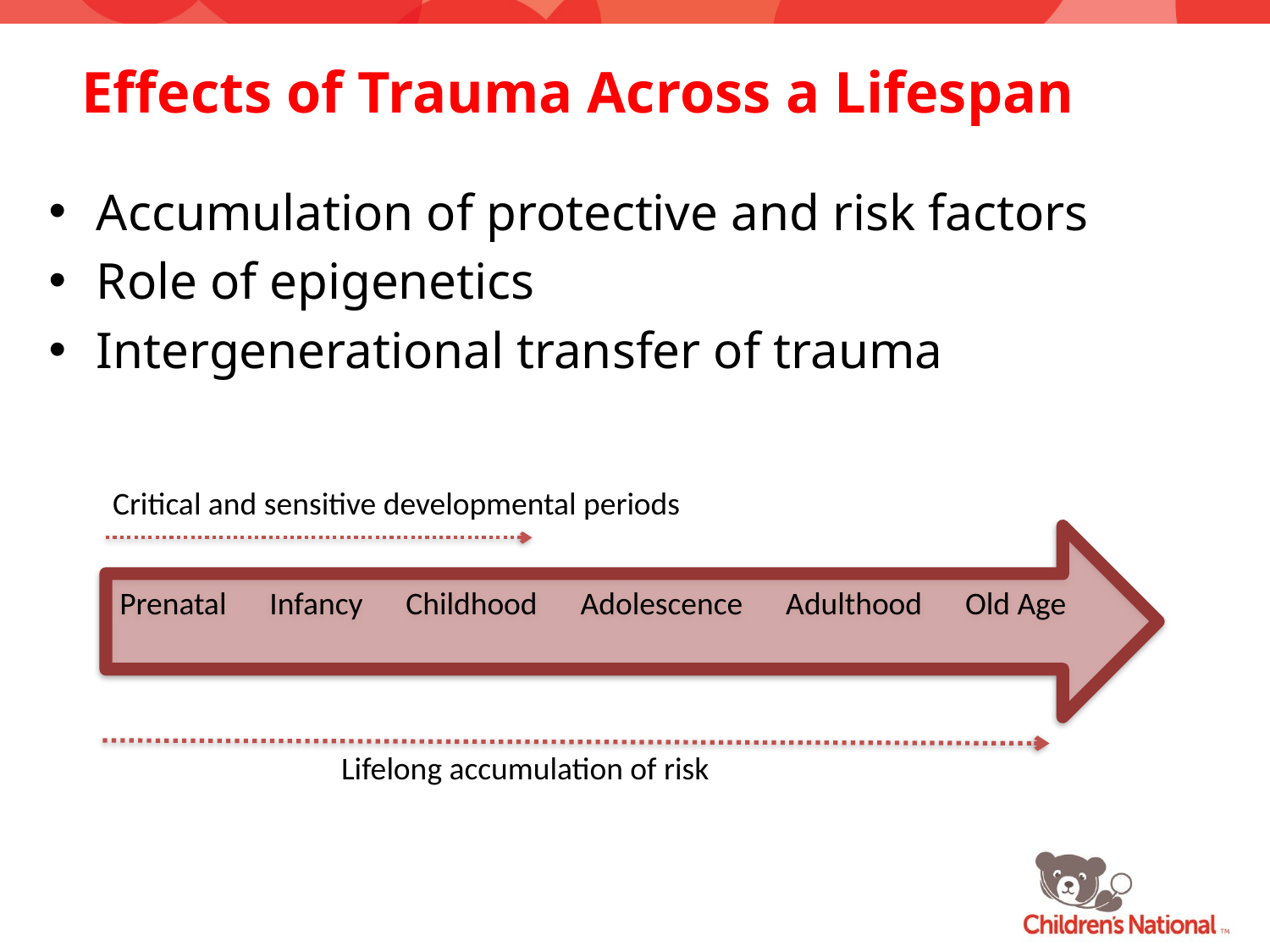

Effects of Trauma Across a Lifespan
Accumulation of protective and risk factors
Role of epigenetics
Intergenerational transfer of trauma
Critical and sensitive developmental periods
Prenatal Infancy Childhood Adolescence Adulthood Old Age
Lifelong accumulation of risk

## Slide 27
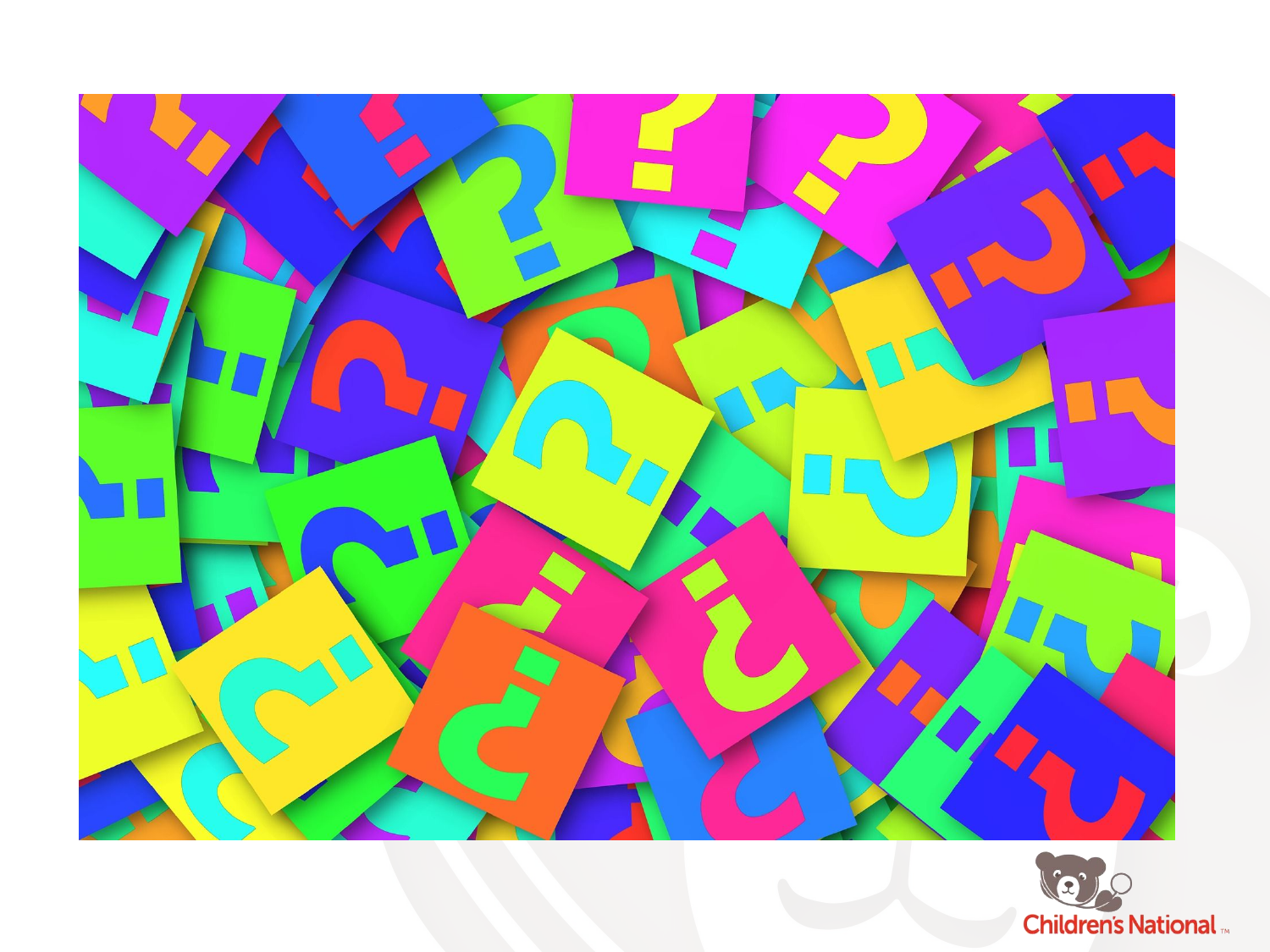

#

## Slide 28
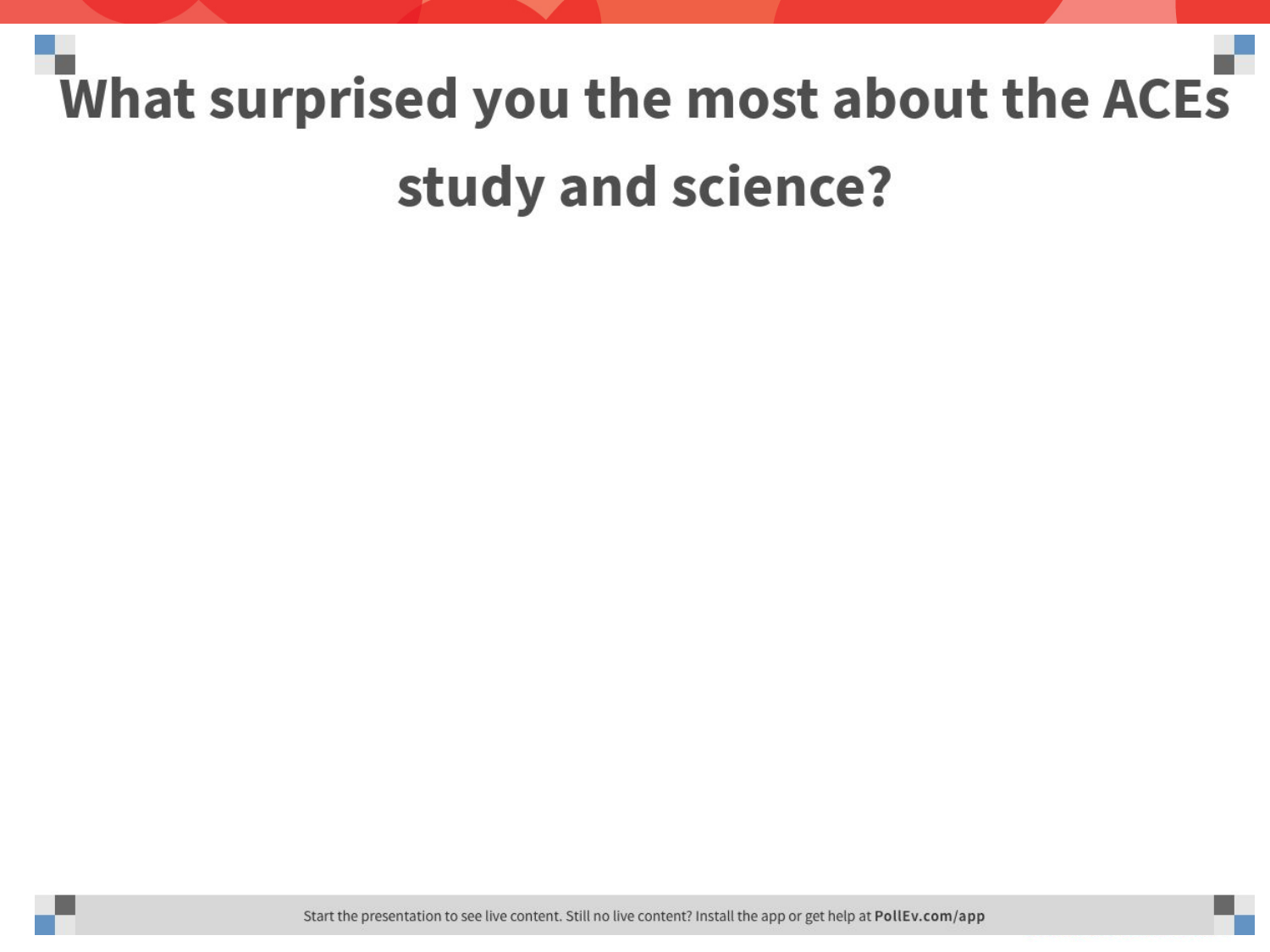

August 31, 2020

## Slide 29
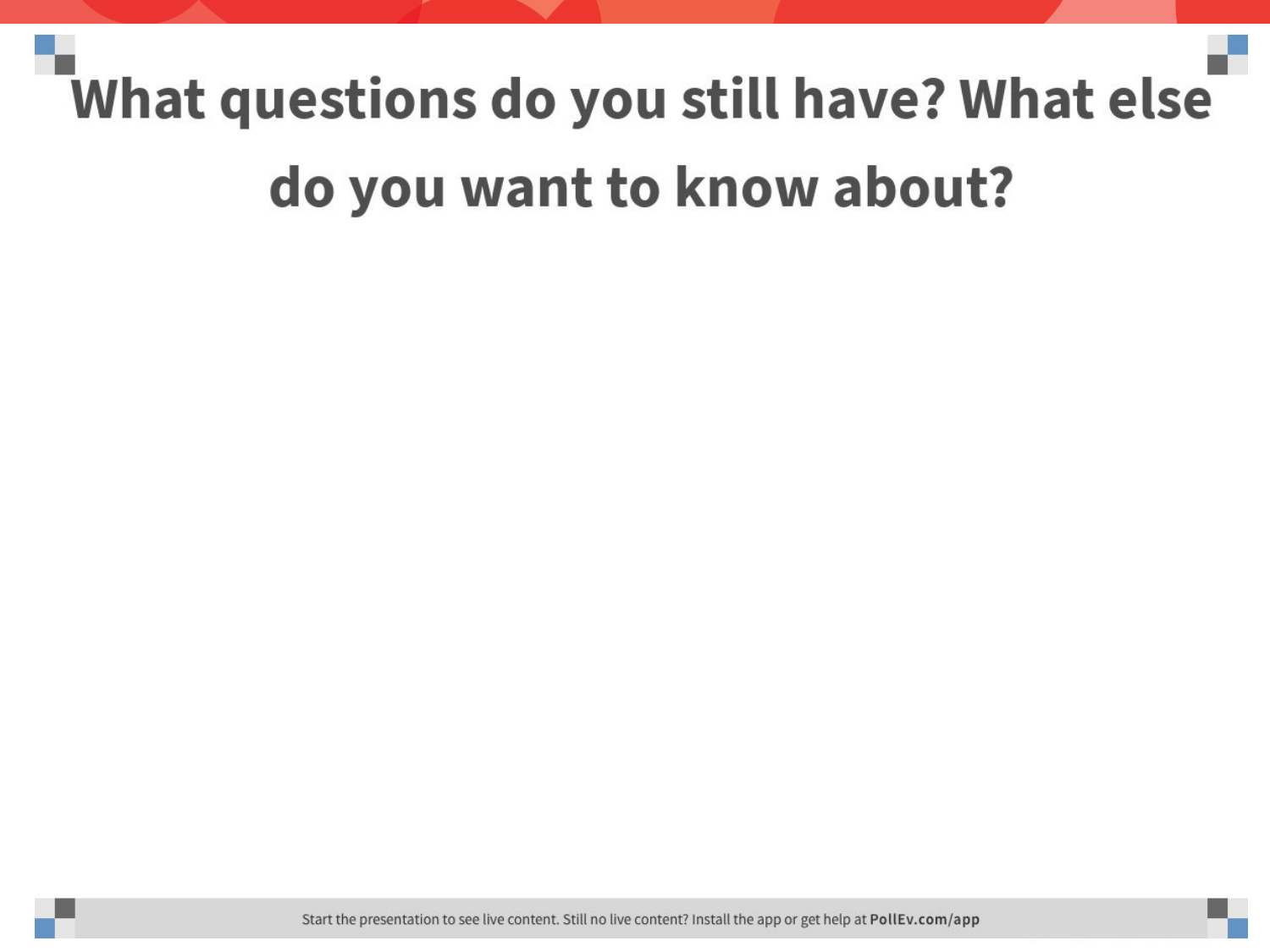

August 31, 2020

## Slide 30
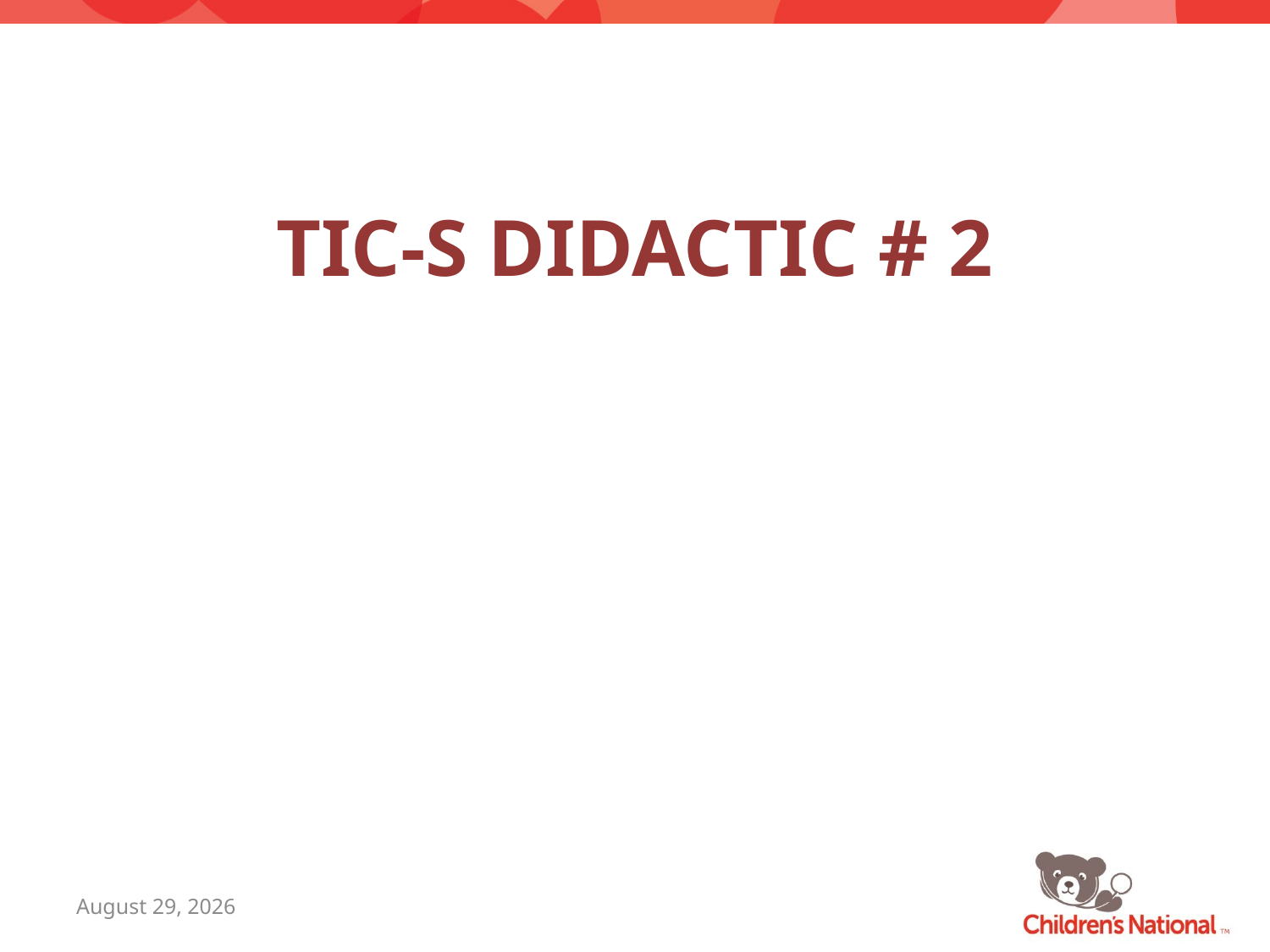

TIC-S DIDACTIC # 2
August 31, 2020

## Slide 31
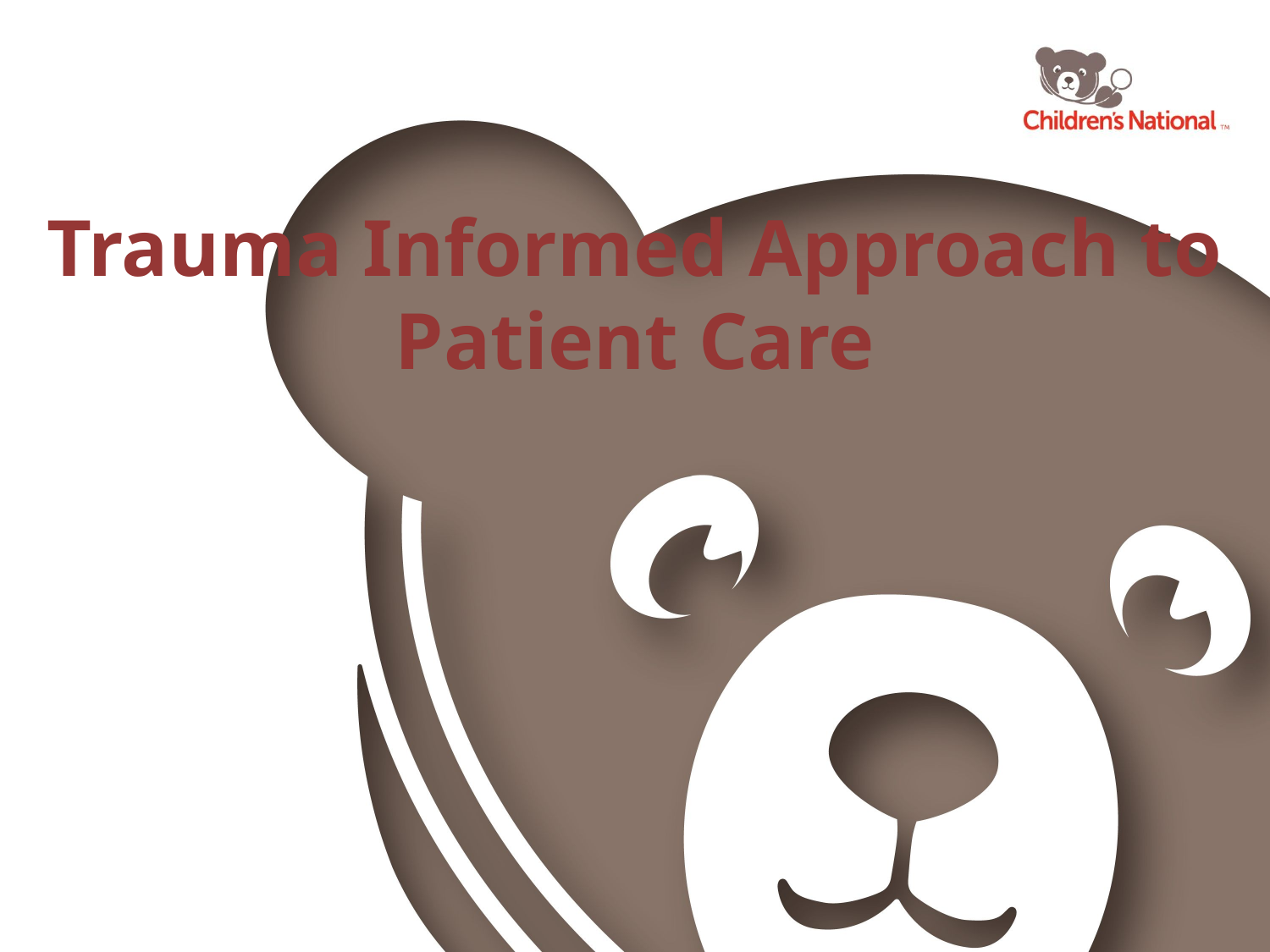

Trauma Informed Approach to Patient Care

## Slide 32
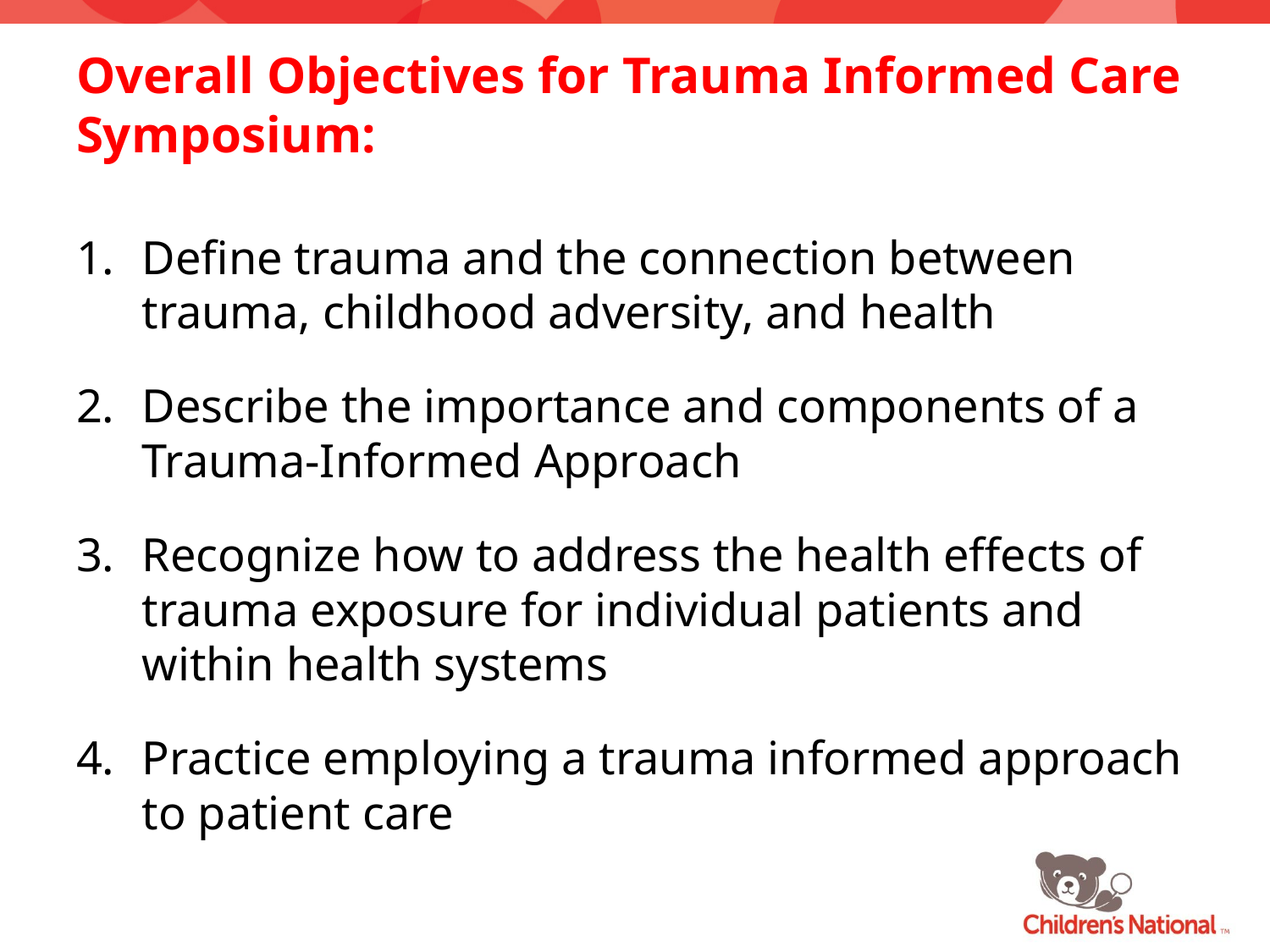

# Overall Objectives for Trauma Informed Care Symposium:
Define trauma and the connection between trauma, childhood adversity, and health
Describe the importance and components of a Trauma-Informed Approach
Recognize how to address the health effects of trauma exposure for individual patients and within health systems
Practice employing a trauma informed approach to patient care

## Slide 33
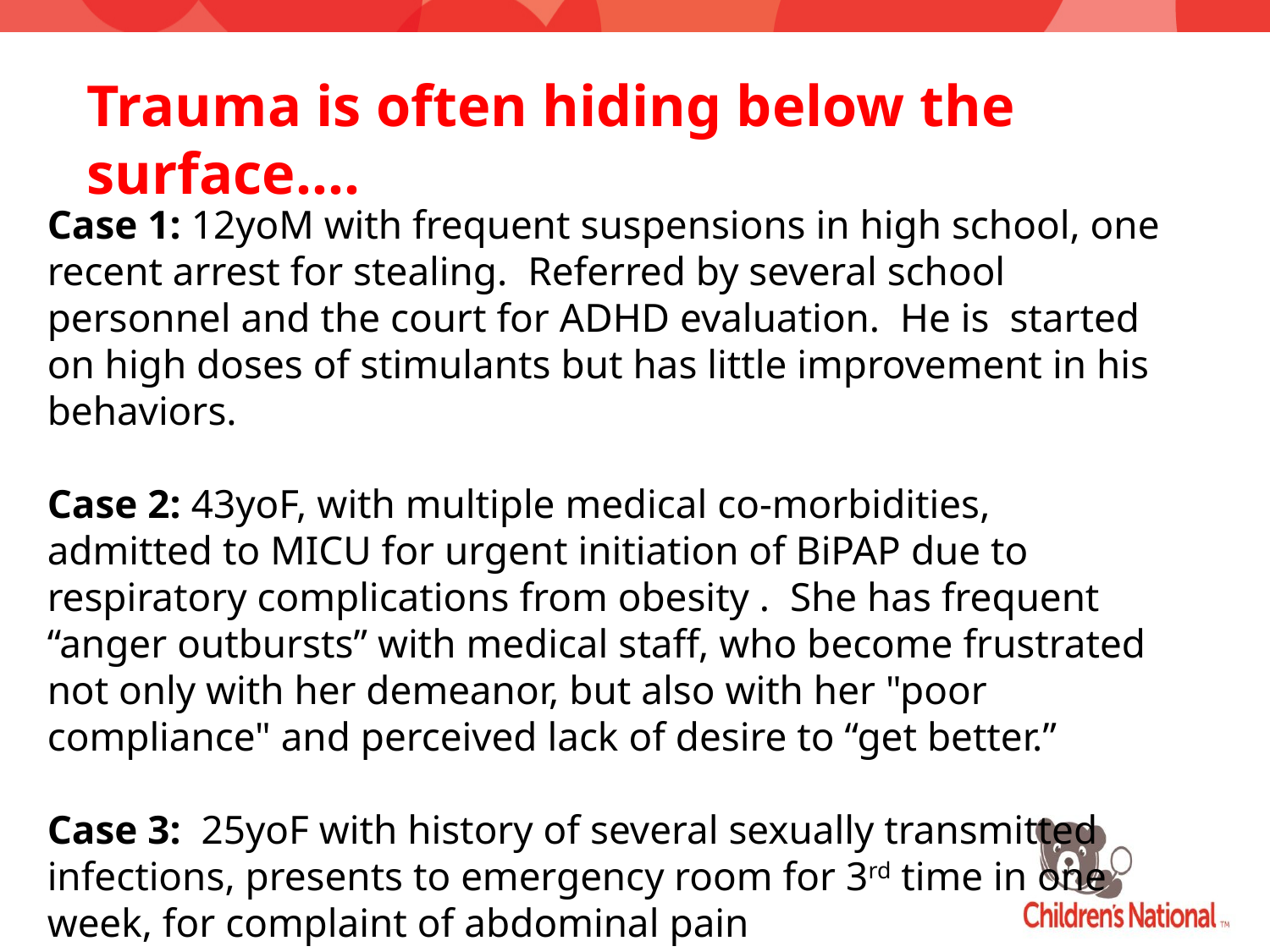

# Trauma is often hiding below the surface….
Case 1: 12yoM with frequent suspensions in high school, one recent arrest for stealing. Referred by several school personnel and the court for ADHD evaluation. He is started on high doses of stimulants but has little improvement in his behaviors.
Case 2: 43yoF, with multiple medical co-morbidities, admitted to MICU for urgent initiation of BiPAP due to respiratory complications from obesity .  She has frequent “anger outbursts” with medical staff, who become frustrated not only with her demeanor, but also with her "poor compliance" and perceived lack of desire to “get better.”
Case 3: 25yoF with history of several sexually transmitted infections, presents to emergency room for 3rd time in one week, for complaint of abdominal pain

## Slide 34
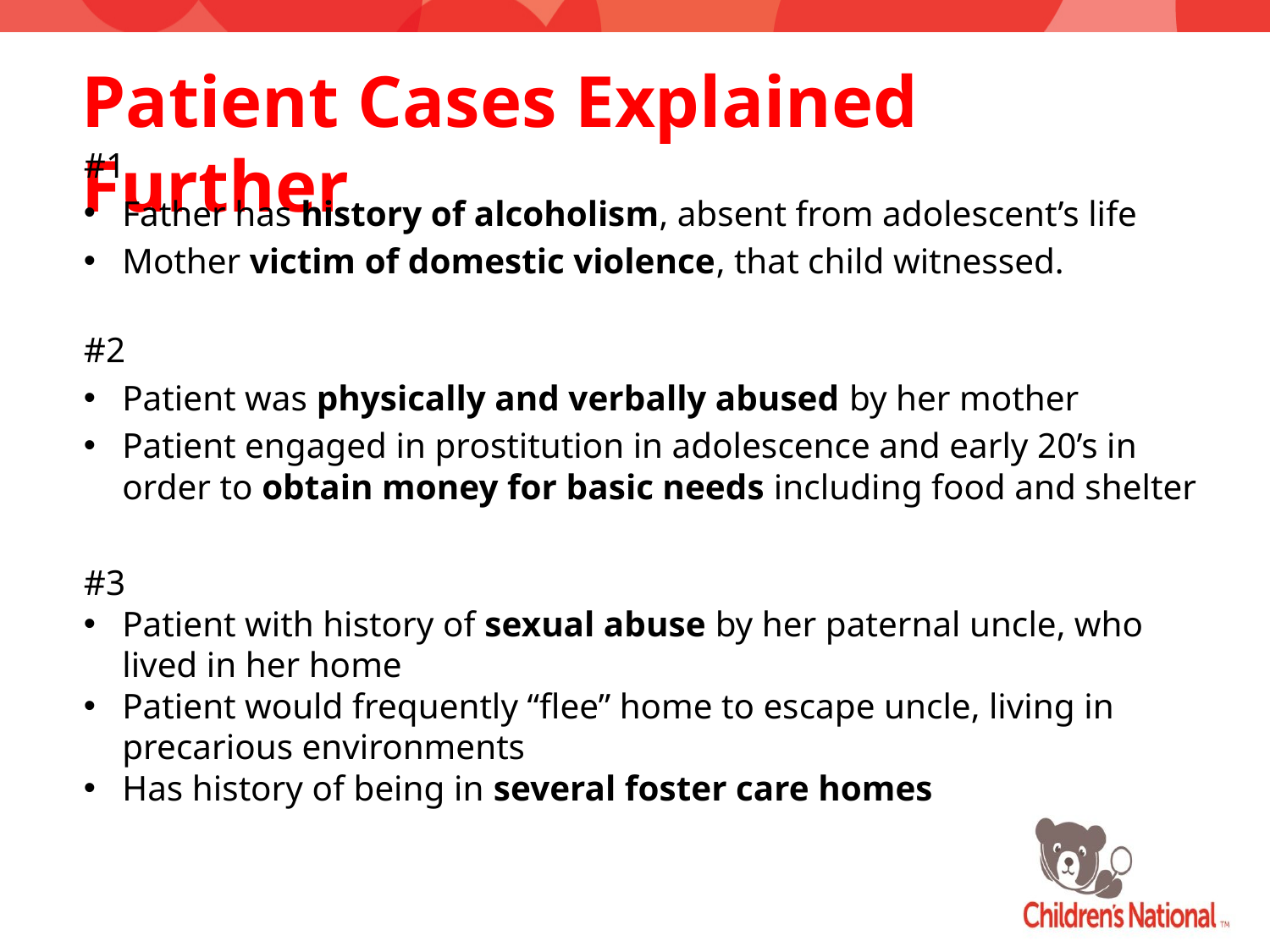

# Patient Cases Explained Further
#1
Father has history of alcoholism, absent from adolescent’s life
Mother victim of domestic violence, that child witnessed.
#2
Patient was physically and verbally abused by her mother
Patient engaged in prostitution in adolescence and early 20’s in order to obtain money for basic needs including food and shelter
#3
Patient with history of sexual abuse by her paternal uncle, who lived in her home
Patient would frequently “flee” home to escape uncle, living in precarious environments
Has history of being in several foster care homes

## Slide 35
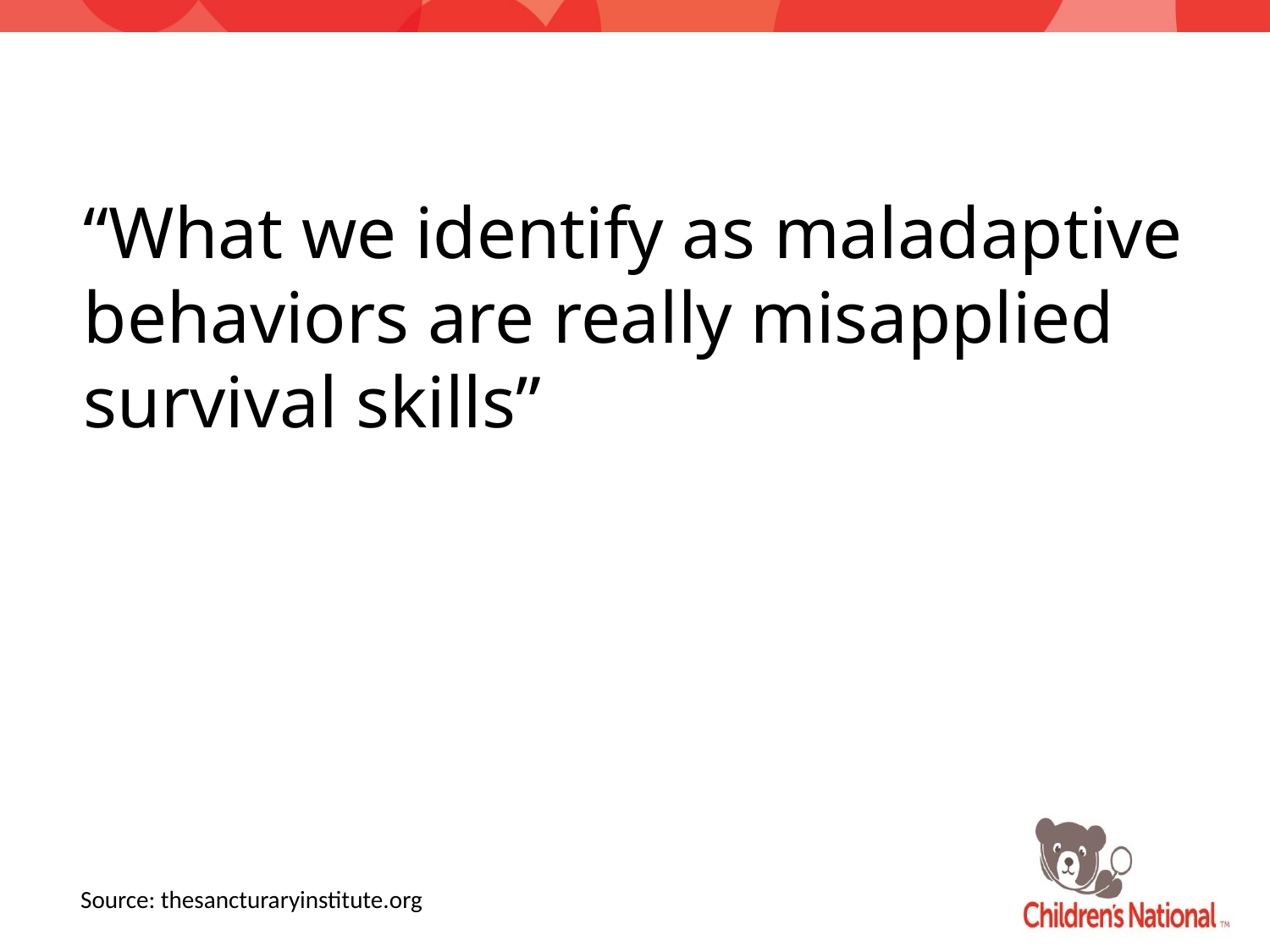

“What we identify as maladaptive behaviors are really misapplied survival skills”
Source: thesancturaryinstitute.org

## Slide 36
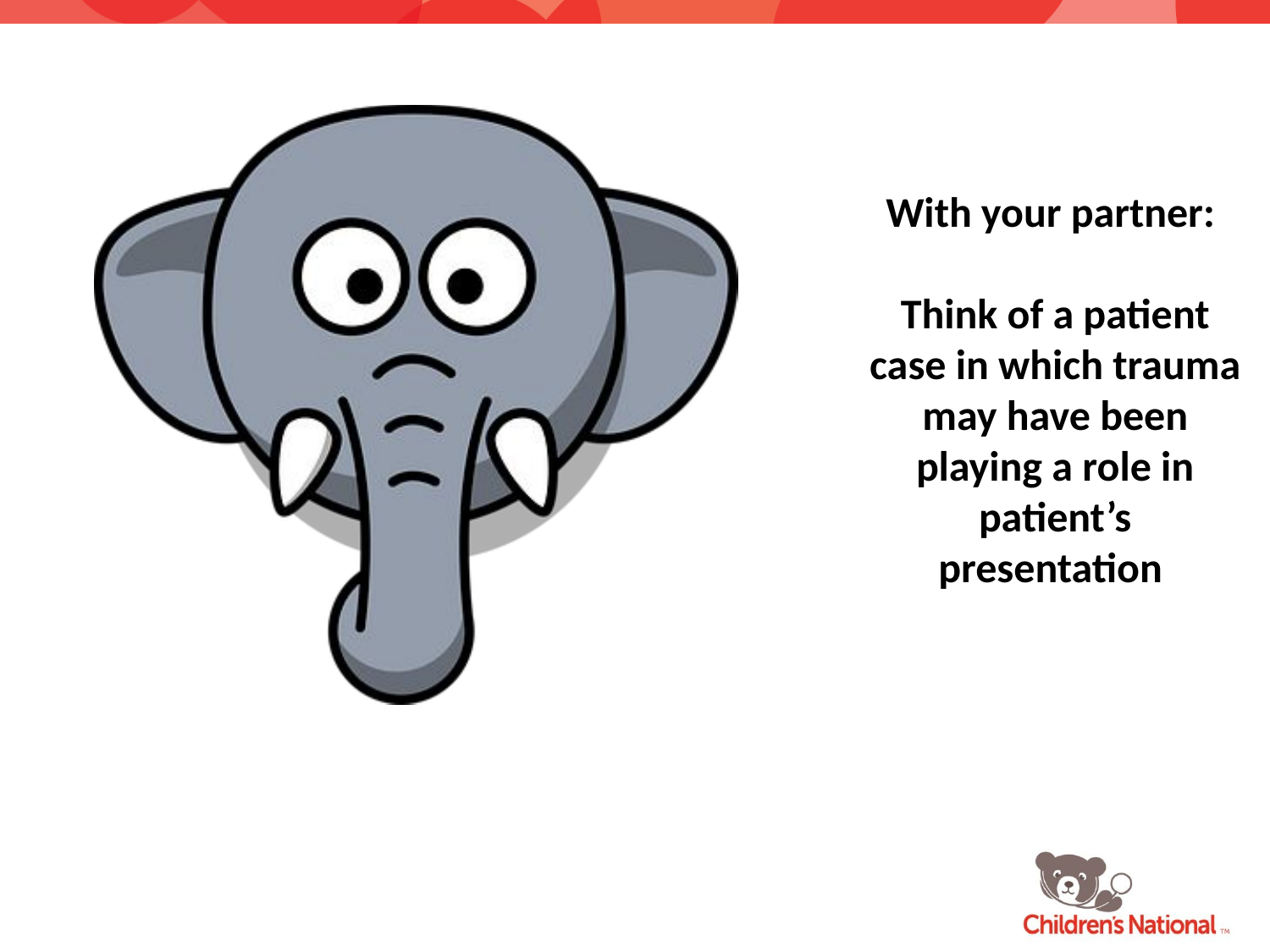

With your partner:
Think of a patient case in which trauma may have been playing a role in patient’s presentation

## Slide 37
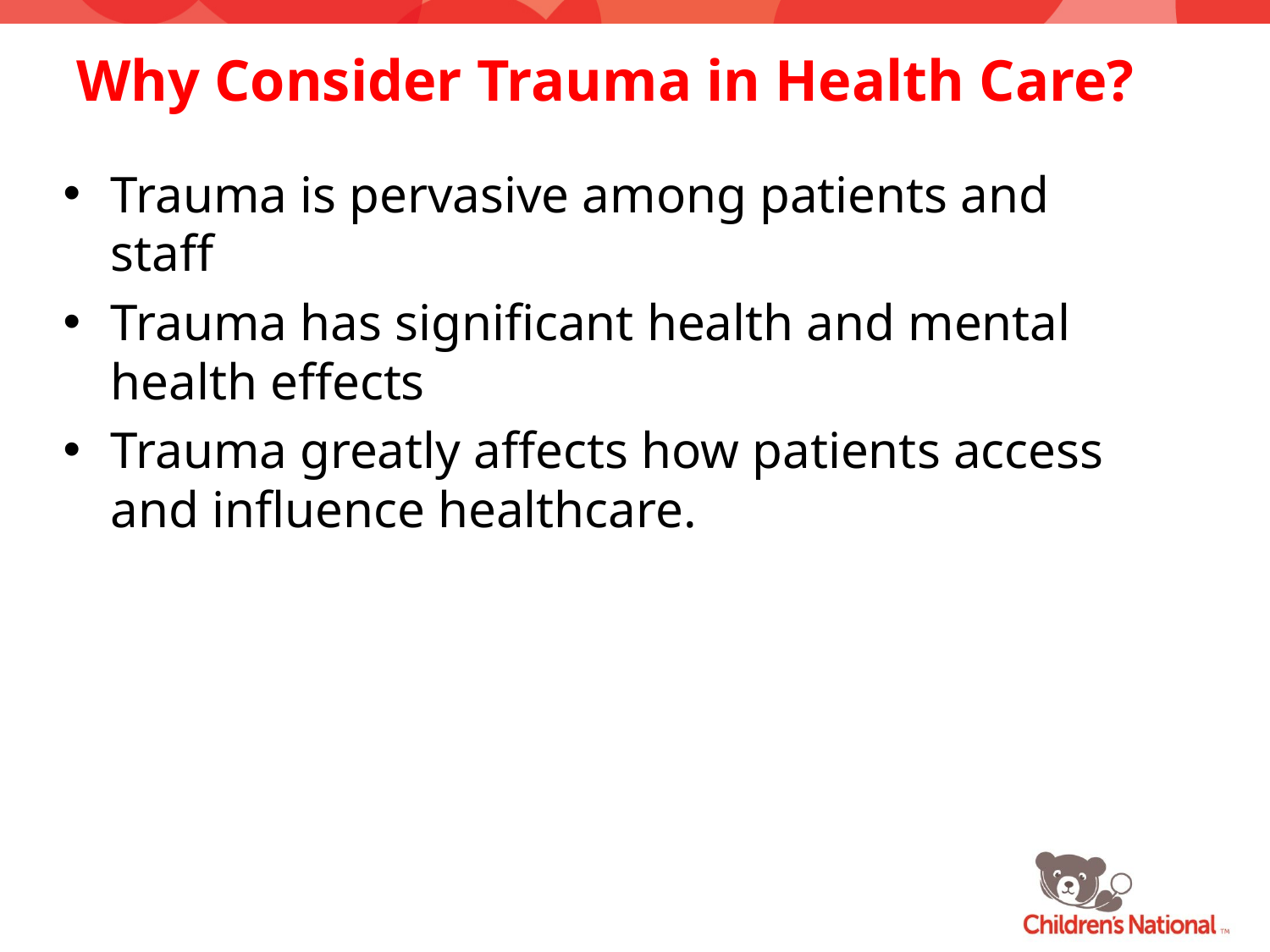

# Why Consider Trauma in Health Care?
Trauma is pervasive among patients and staff
Trauma has significant health and mental health effects
Trauma greatly affects how patients access and influence healthcare.

## Slide 38
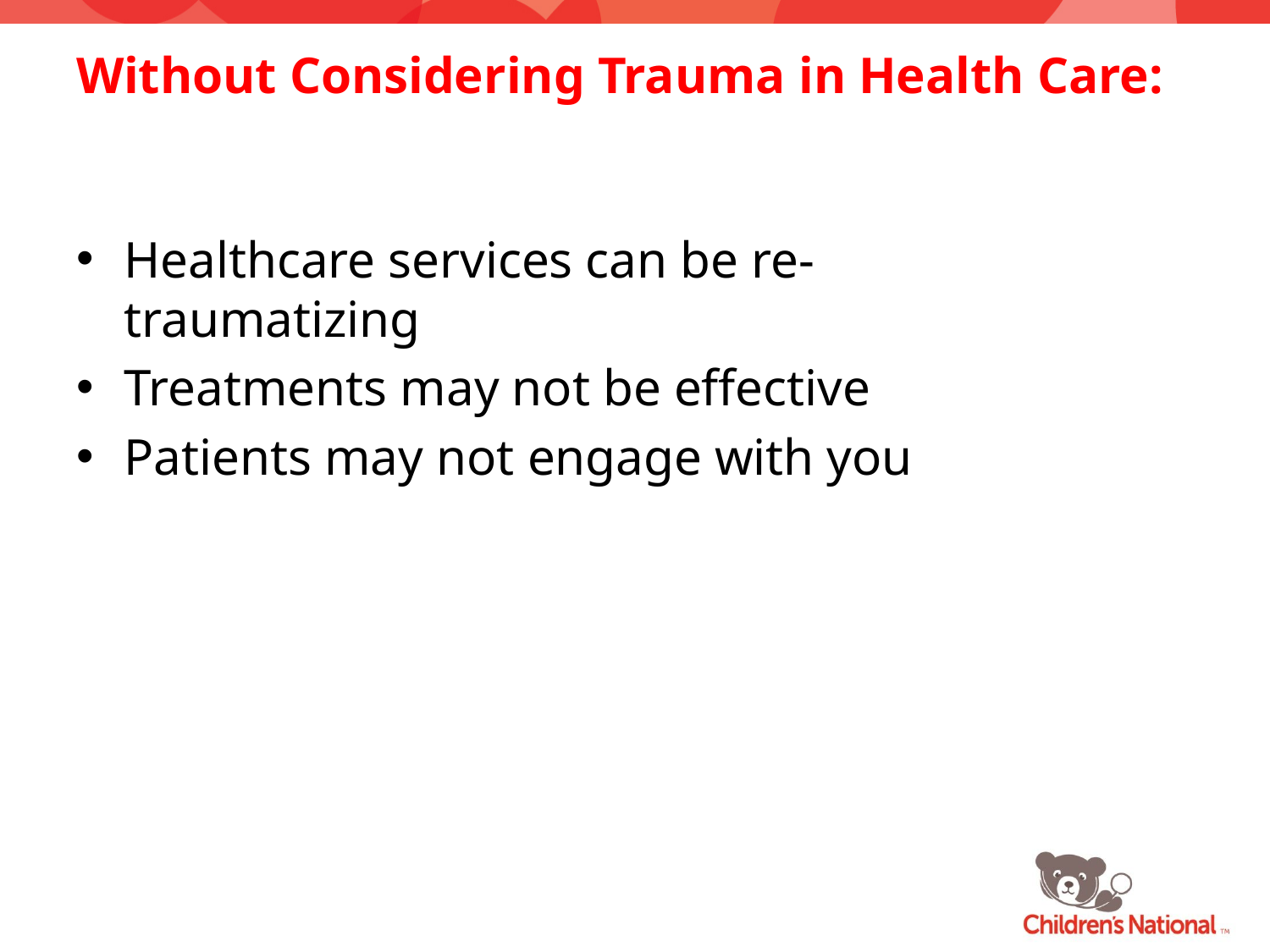

# Without Considering Trauma in Health Care:
Healthcare services can be re-traumatizing
Treatments may not be effective
Patients may not engage with you

## Slide 39
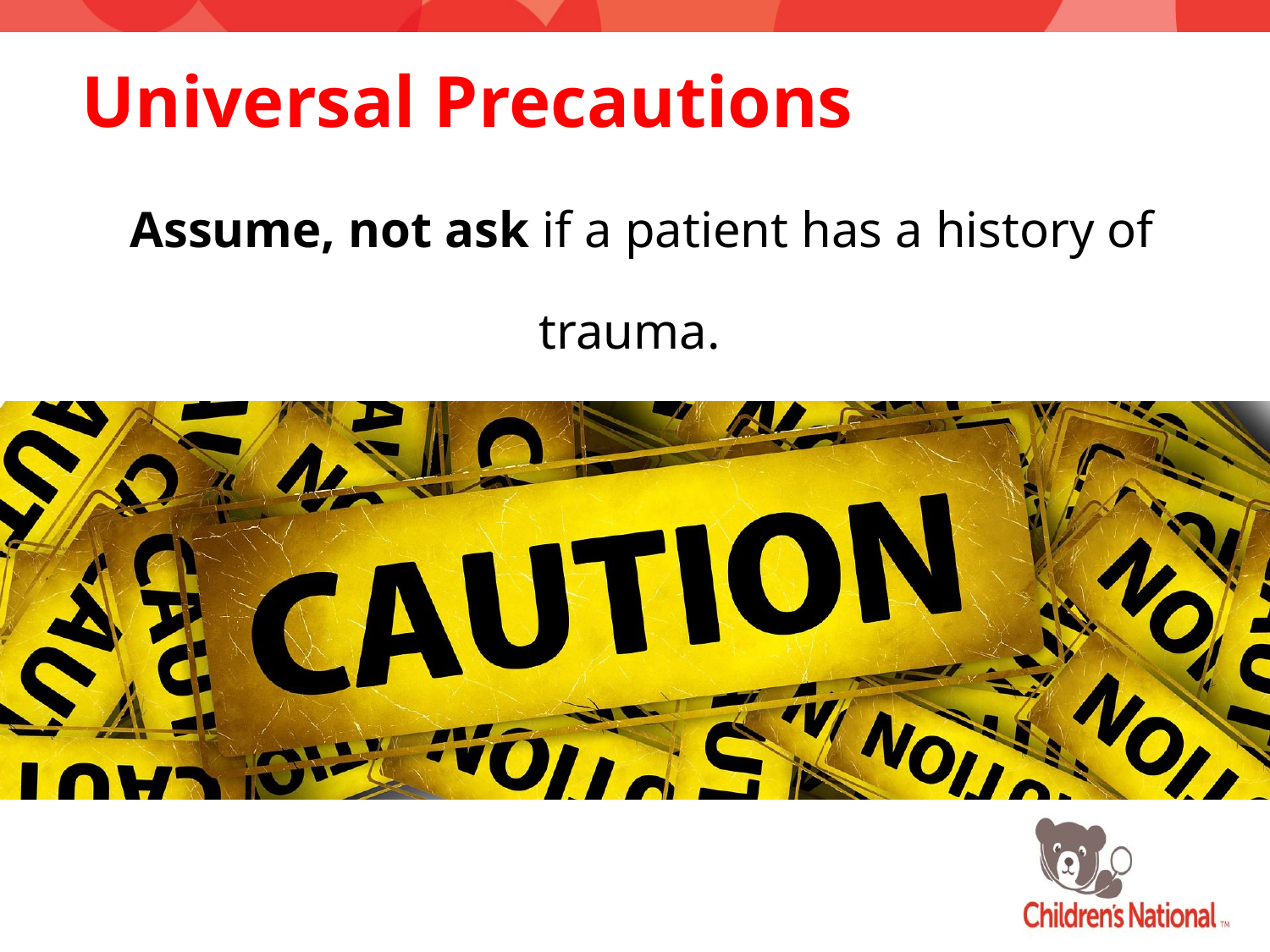

# Universal Precautions
Assume, not ask if a patient has a history of trauma.

## Slide 40
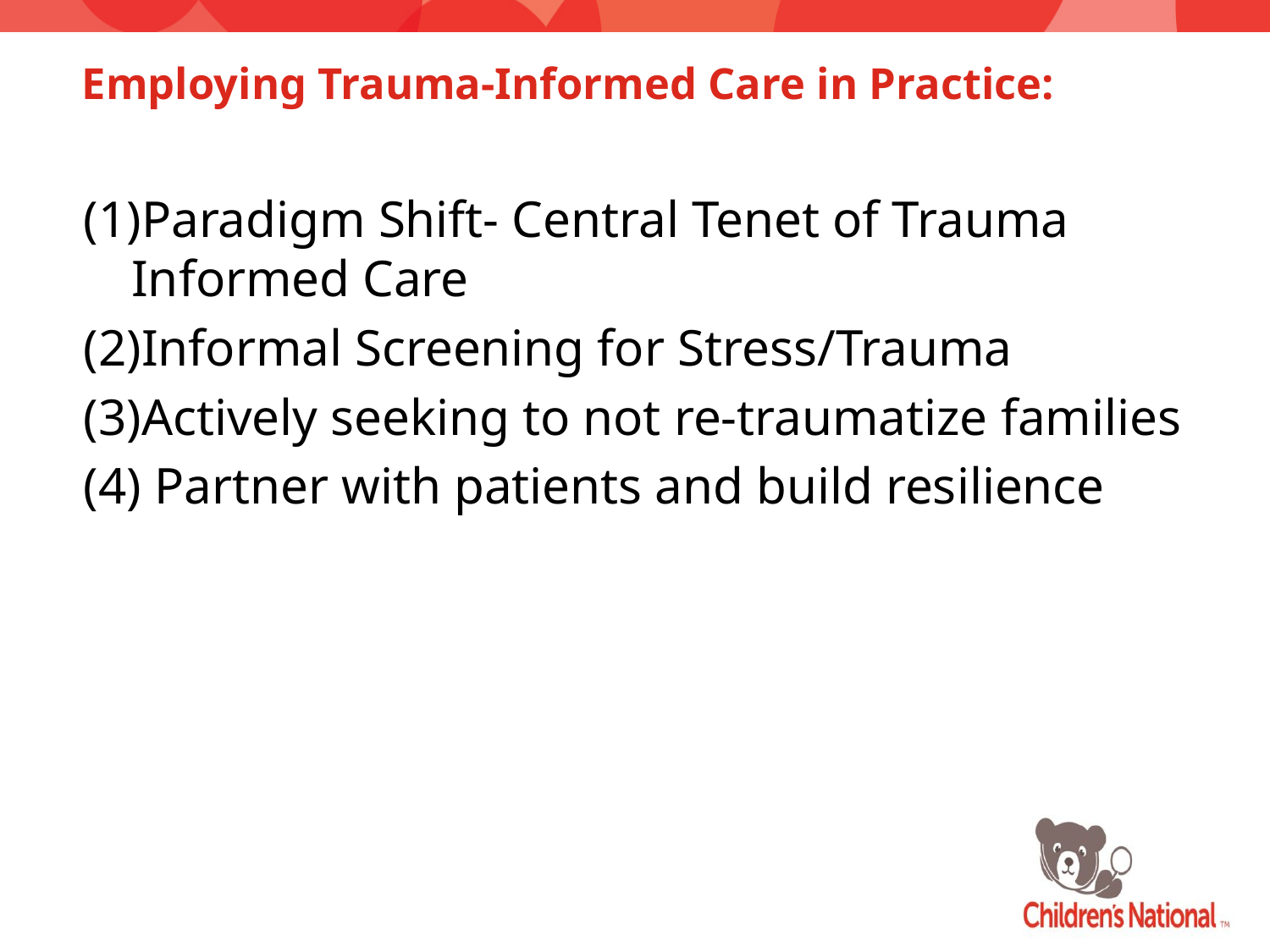

# Employing Trauma-Informed Care in Practice:
Paradigm Shift- Central Tenet of Trauma Informed Care
Informal Screening for Stress/Trauma
Actively seeking to not re-traumatize families
 Partner with patients and build resilience

## Slide 41
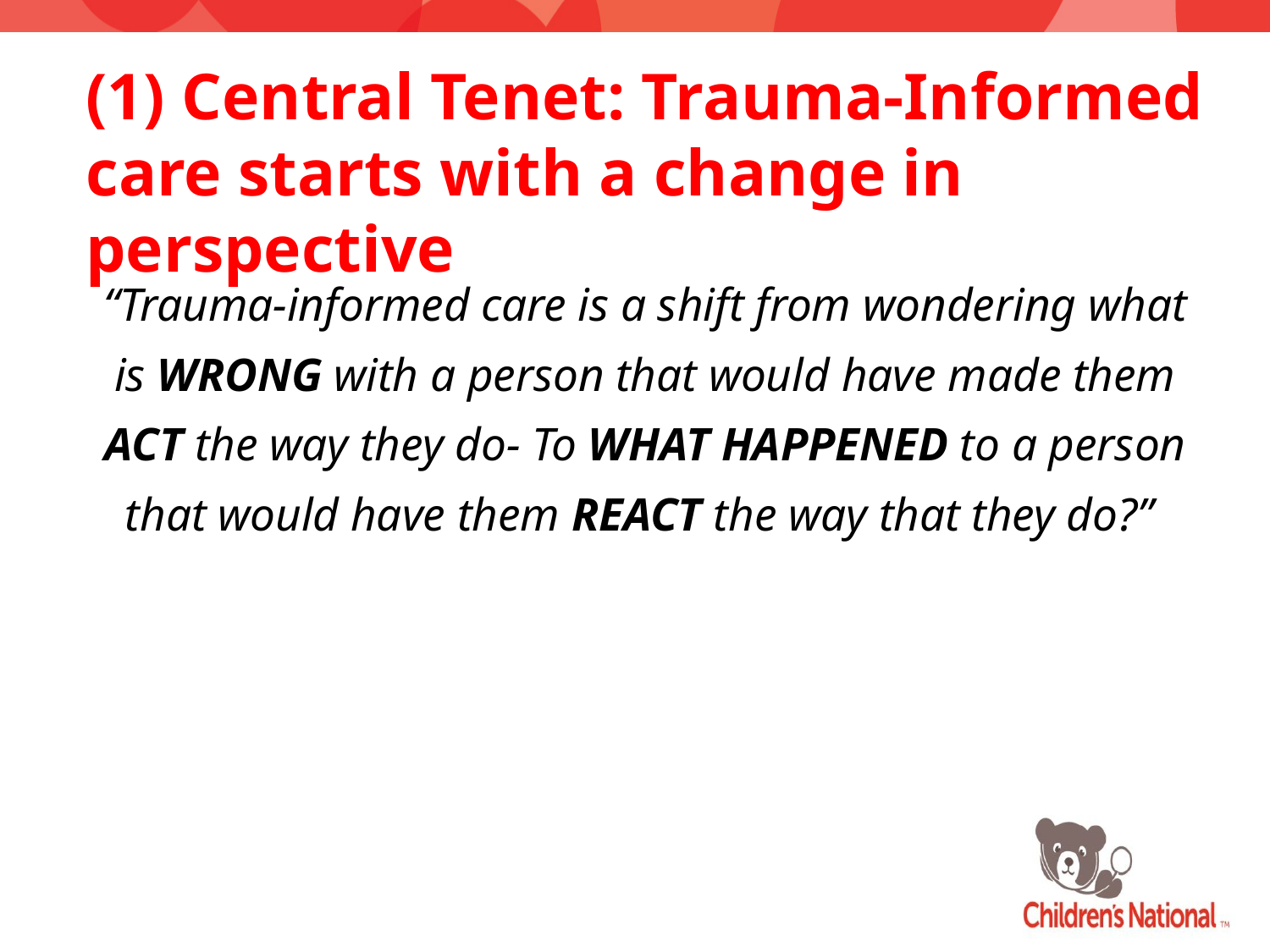

# (1) Central Tenet: Trauma-Informed care starts with a change in perspective
“Trauma-informed care is a shift from wondering what is WRONG with a person that would have made them ACT the way they do- To WHAT HAPPENED to a person that would have them REACT the way that they do?”

## Slide 42
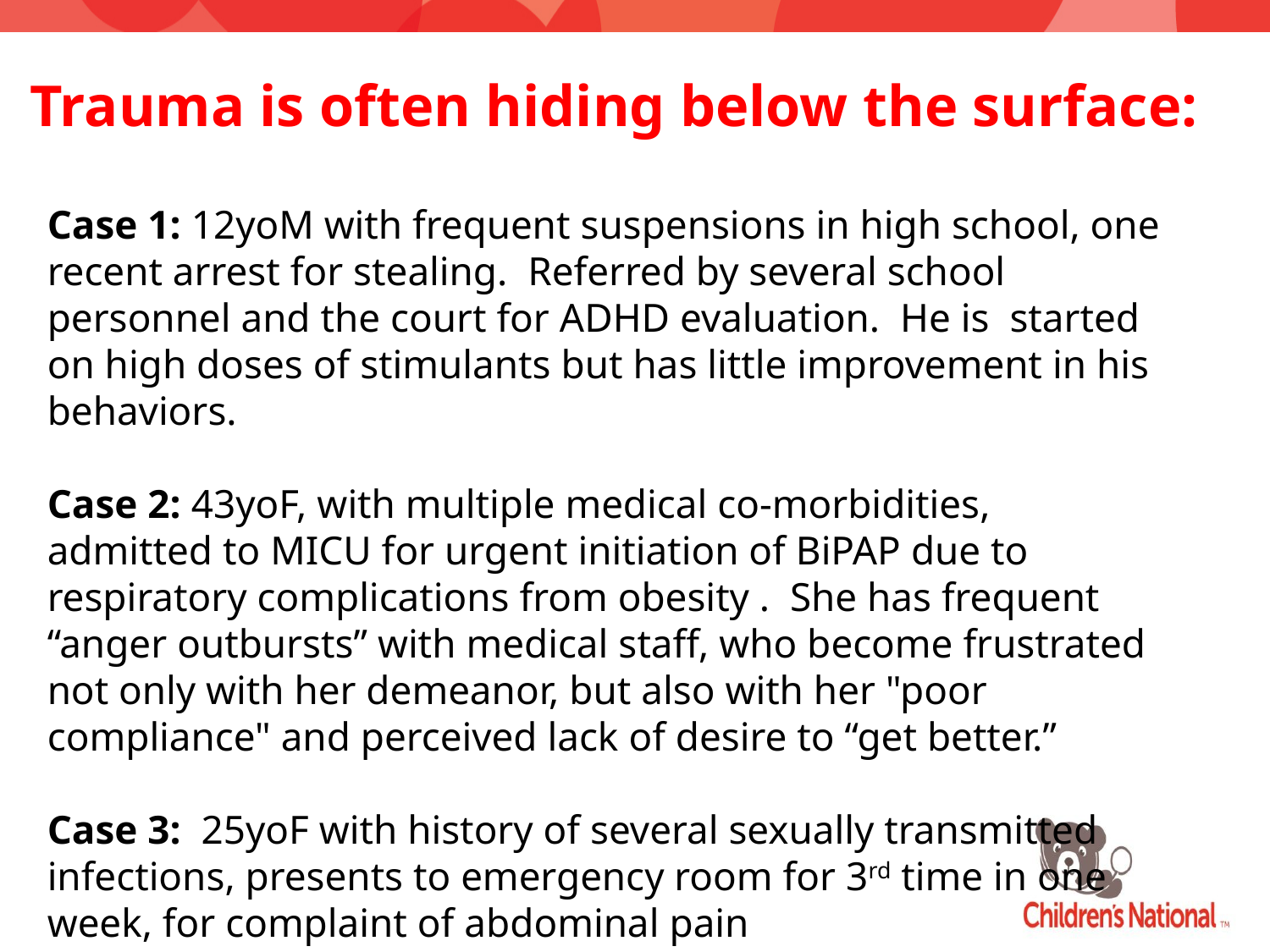

# Trauma is often hiding below the surface:
Case 1: 12yoM with frequent suspensions in high school, one recent arrest for stealing. Referred by several school personnel and the court for ADHD evaluation. He is started on high doses of stimulants but has little improvement in his behaviors.
Case 2: 43yoF, with multiple medical co-morbidities, admitted to MICU for urgent initiation of BiPAP due to respiratory complications from obesity .  She has frequent “anger outbursts” with medical staff, who become frustrated not only with her demeanor, but also with her "poor compliance" and perceived lack of desire to “get better.”
Case 3: 25yoF with history of several sexually transmitted infections, presents to emergency room for 3rd time in one week, for complaint of abdominal pain

## Slide 43
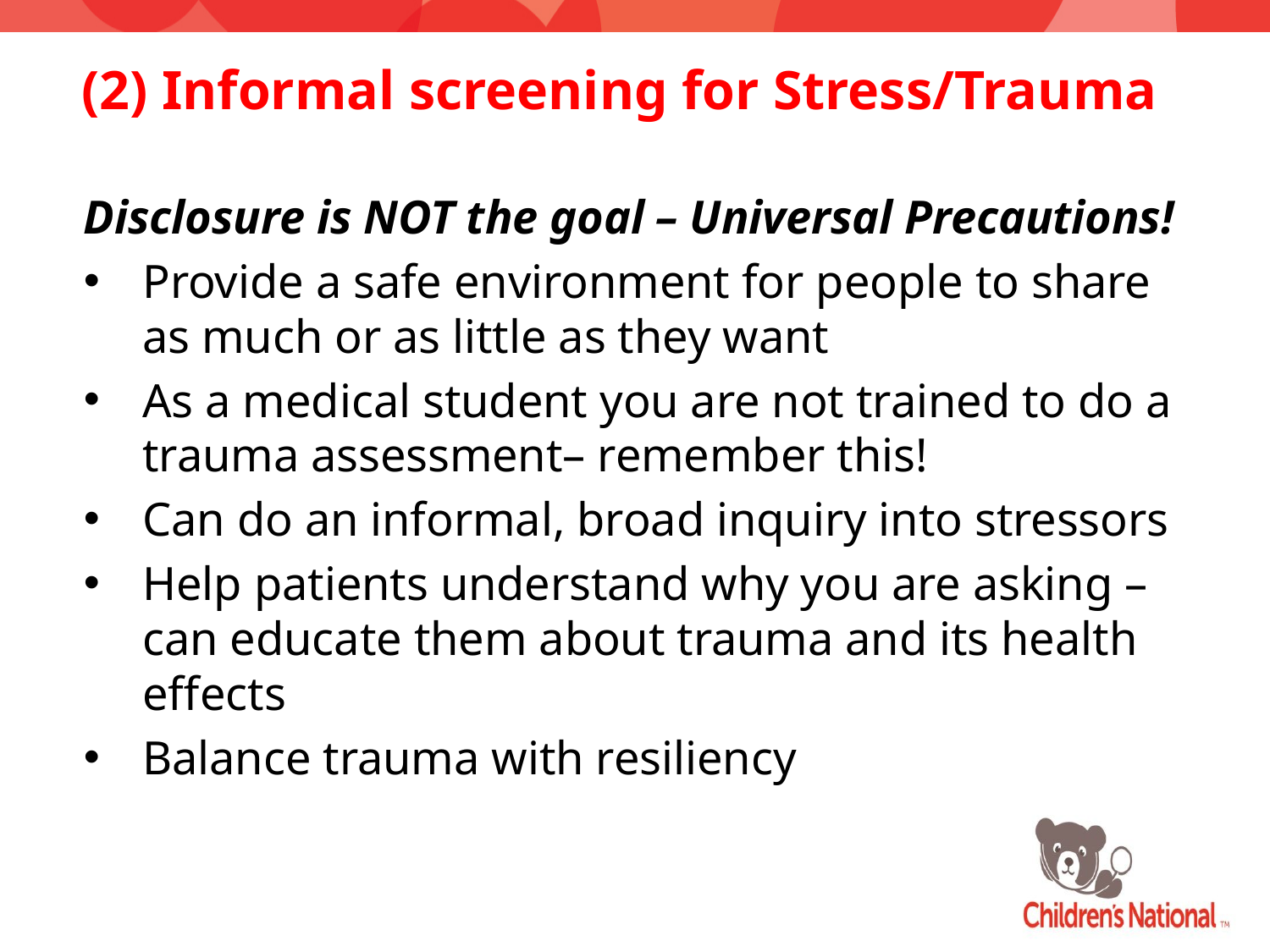

# (2) Informal screening for Stress/Trauma
Disclosure is NOT the goal – Universal Precautions!
Provide a safe environment for people to share as much or as little as they want
As a medical student you are not trained to do a trauma assessment– remember this!
Can do an informal, broad inquiry into stressors
Help patients understand why you are asking – can educate them about trauma and its health effects
Balance trauma with resiliency

## Slide 44
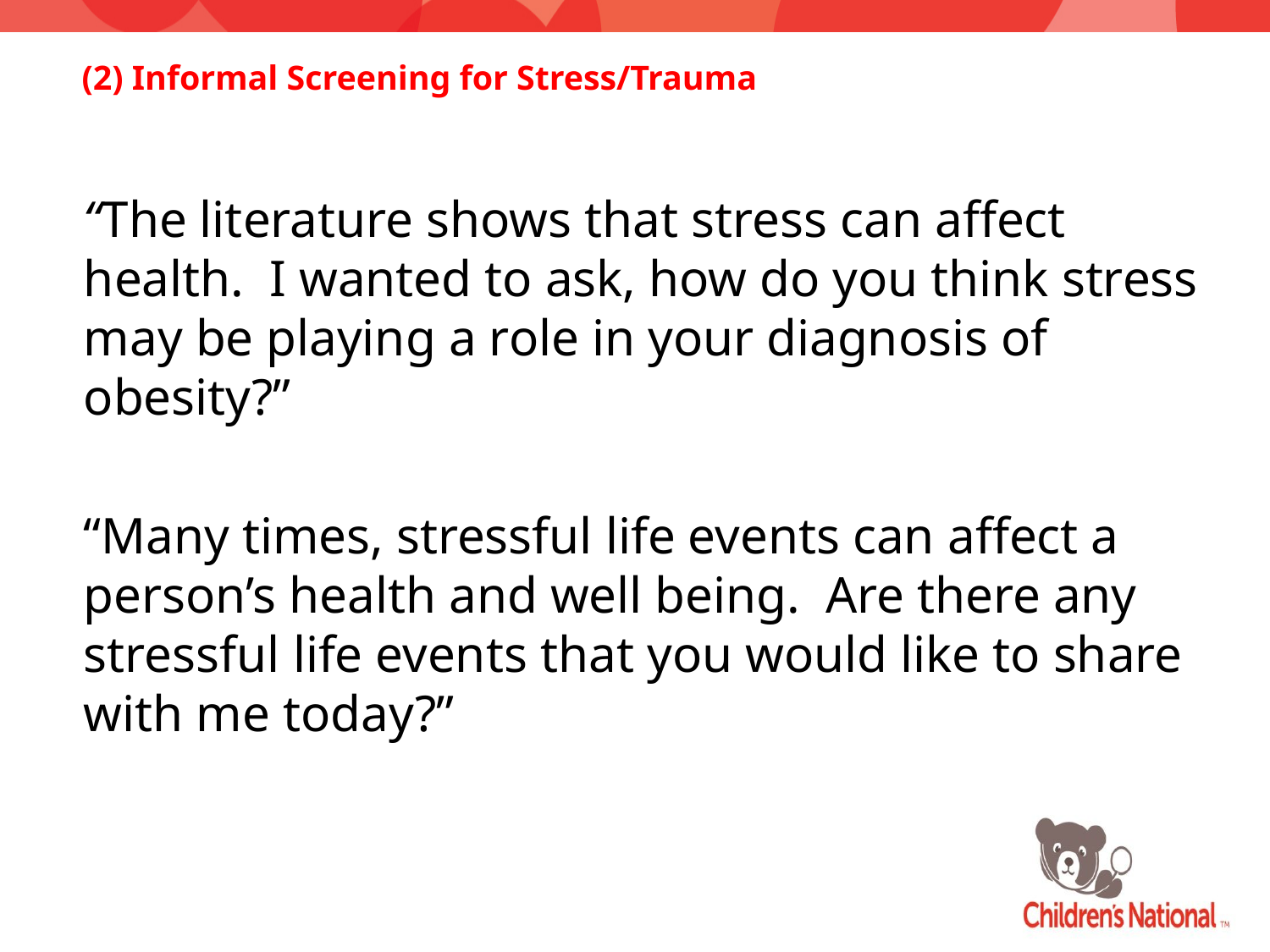

# (2) Informal Screening for Stress/Trauma
“The literature shows that stress can affect health. I wanted to ask, how do you think stress may be playing a role in your diagnosis of obesity?”
“Many times, stressful life events can affect a person’s health and well being. Are there any stressful life events that you would like to share with me today?”

## Slide 45
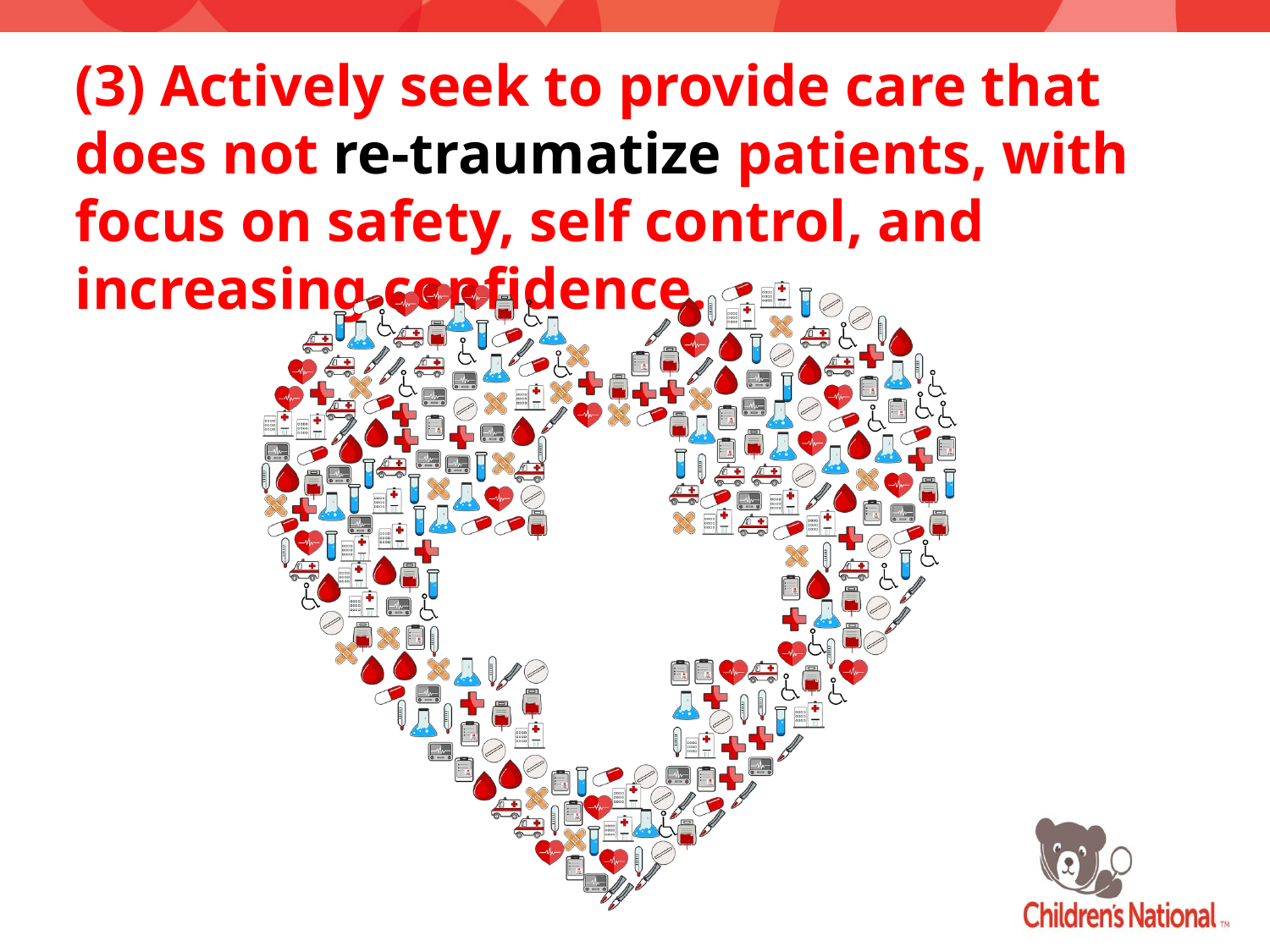

# (3) Actively seek to provide care that does not re-traumatize patients, with focus on safety, self control, and increasing confidence.

## Slide 46
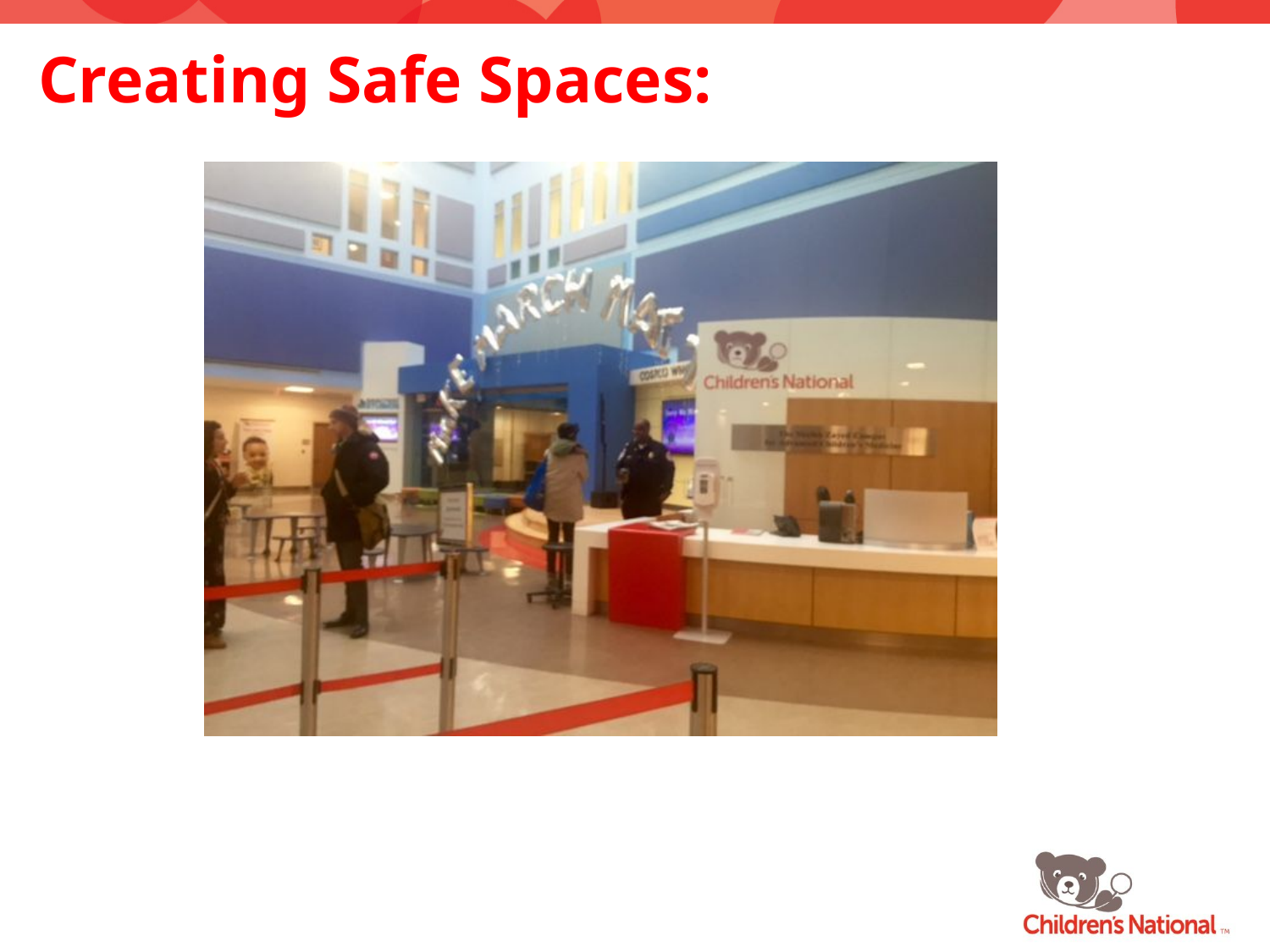

# Creating Safe Spaces:

## Slide 47
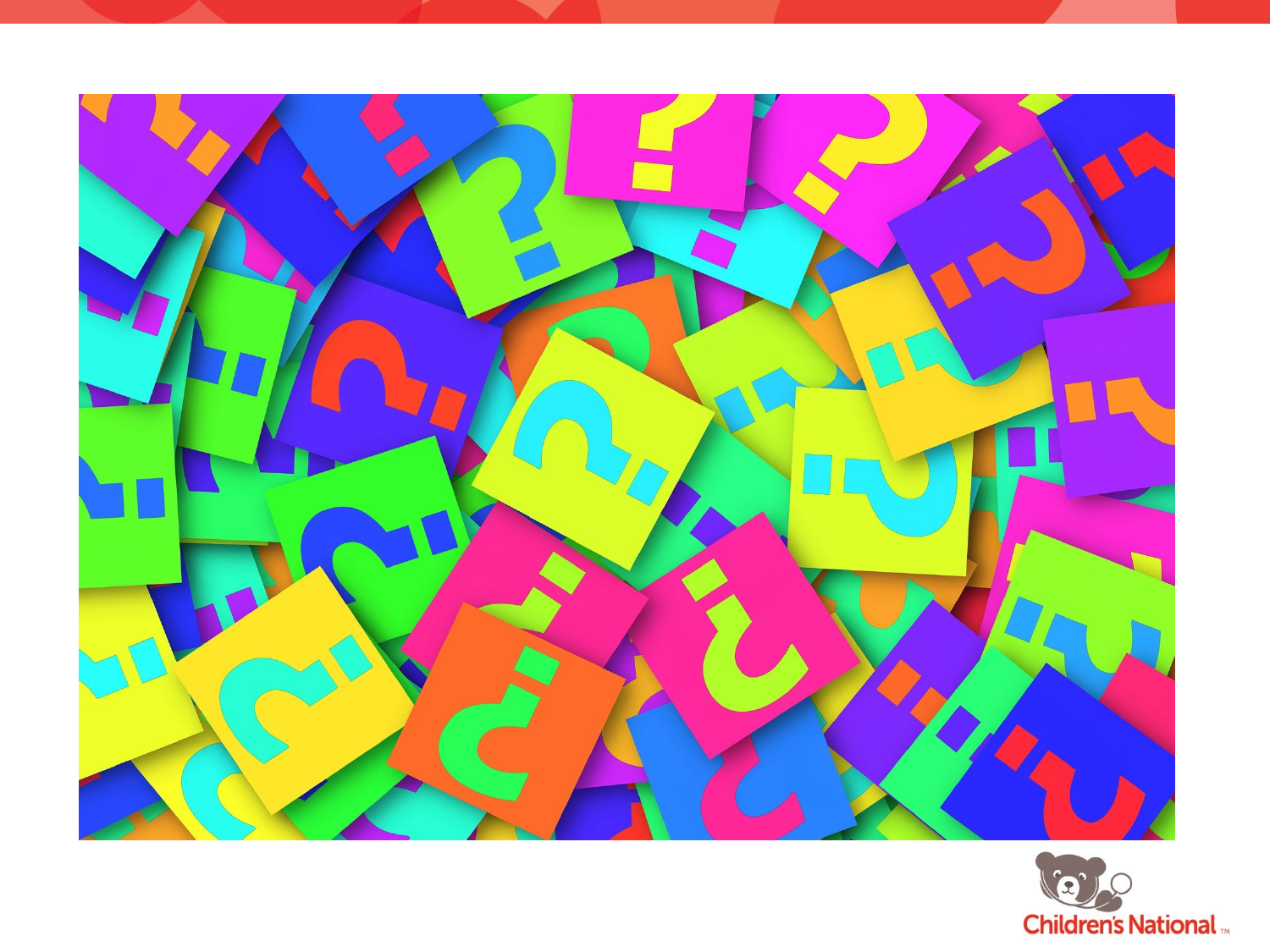

#

## Slide 48
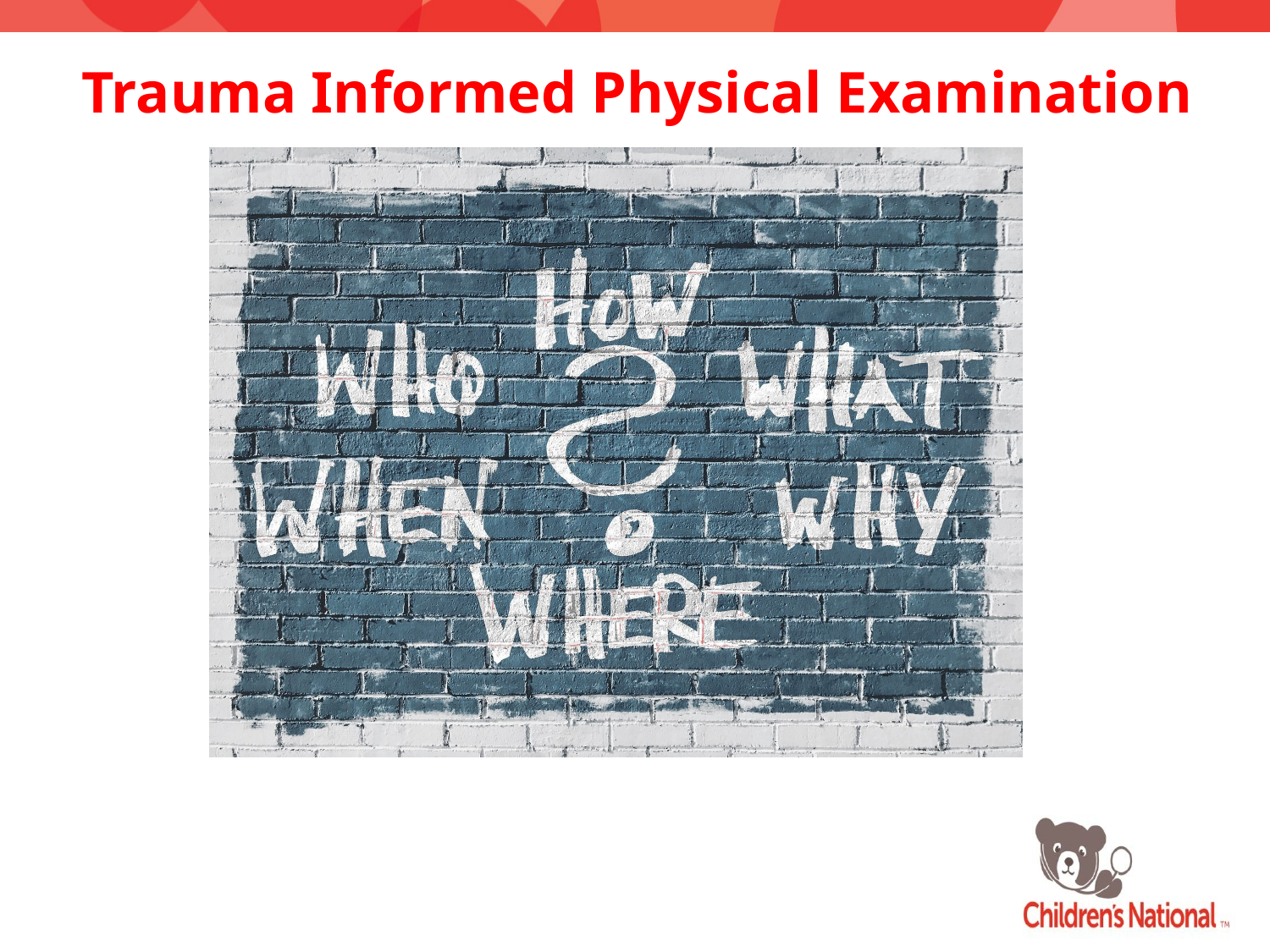

# Trauma Informed Physical Examination

## Slide 49
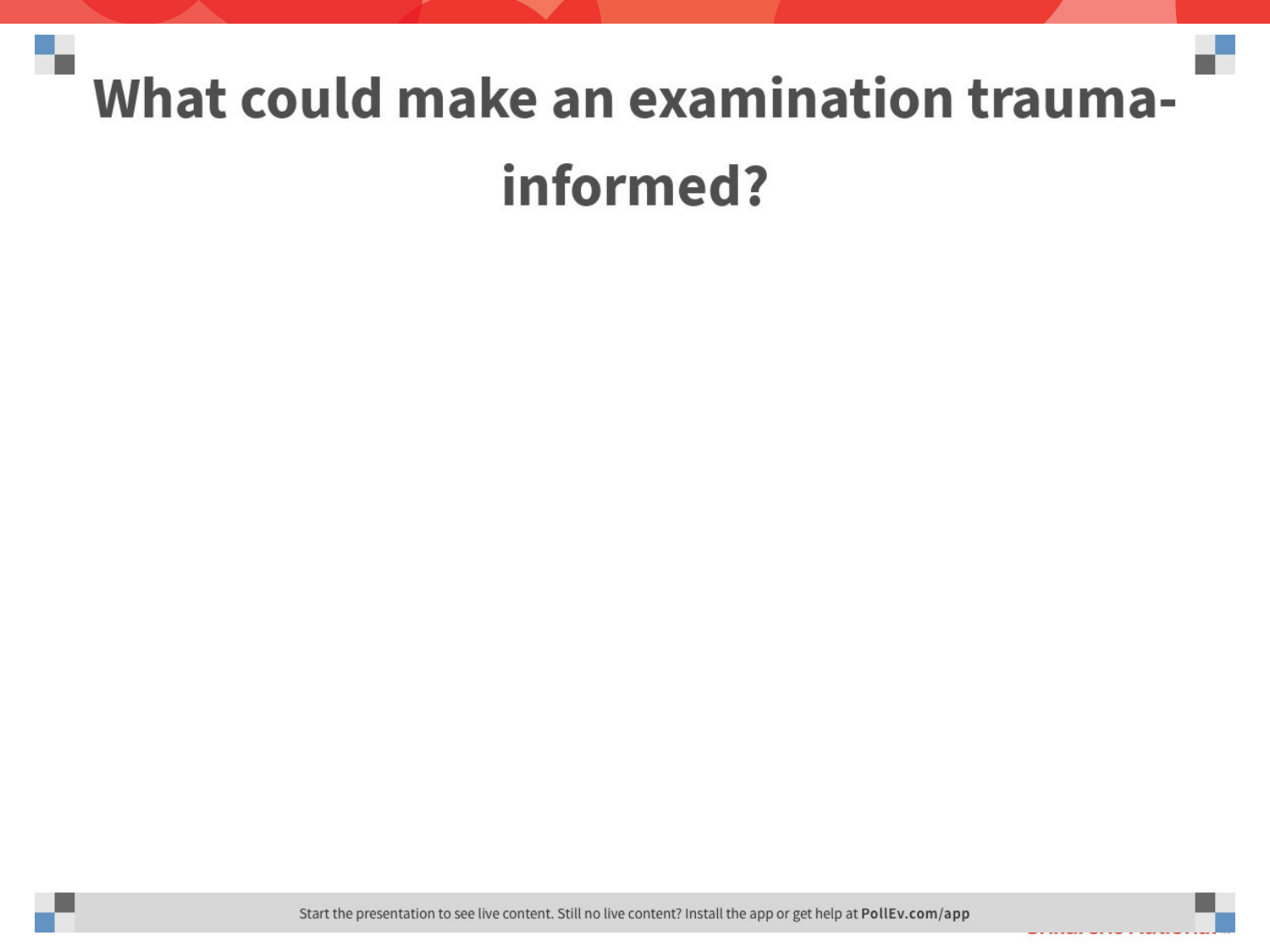

August 31, 2020

## Slide 50
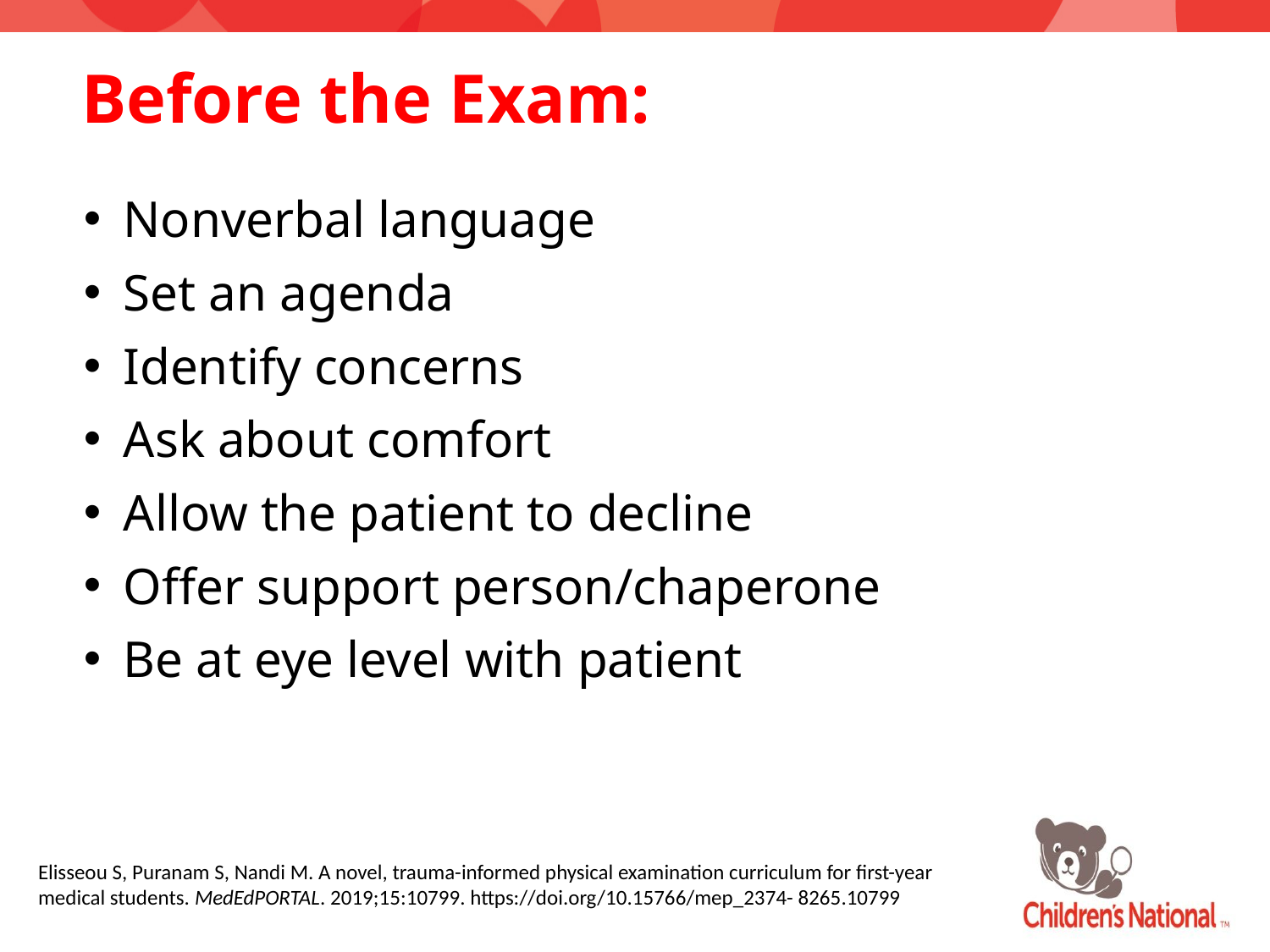

# Before the Exam:
Nonverbal language
Set an agenda
Identify concerns
Ask about comfort
Allow the patient to decline
Offer support person/chaperone
Be at eye level with patient
Elisseou S, Puranam S, Nandi M. A novel, trauma-informed physical examination curriculum for first-year medical students. MedEdPORTAL. 2019;15:10799. https://doi.org/10.15766/mep_2374- 8265.10799

## Slide 51
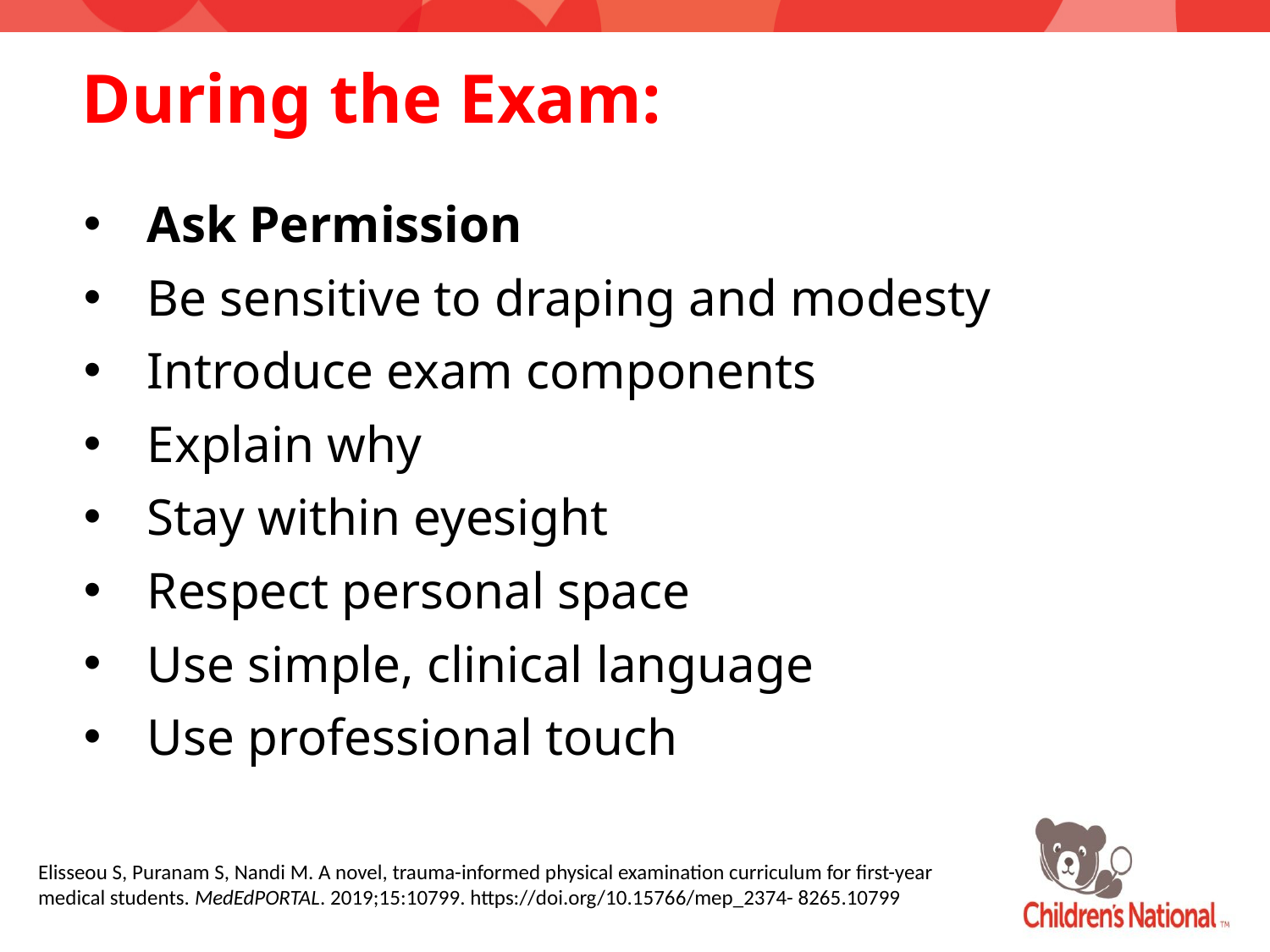

# During the Exam:
Ask Permission
Be sensitive to draping and modesty
Introduce exam components
Explain why
Stay within eyesight
Respect personal space
Use simple, clinical language
Use professional touch
Elisseou S, Puranam S, Nandi M. A novel, trauma-informed physical examination curriculum for first-year medical students. MedEdPORTAL. 2019;15:10799. https://doi.org/10.15766/mep_2374- 8265.10799

## Slide 52
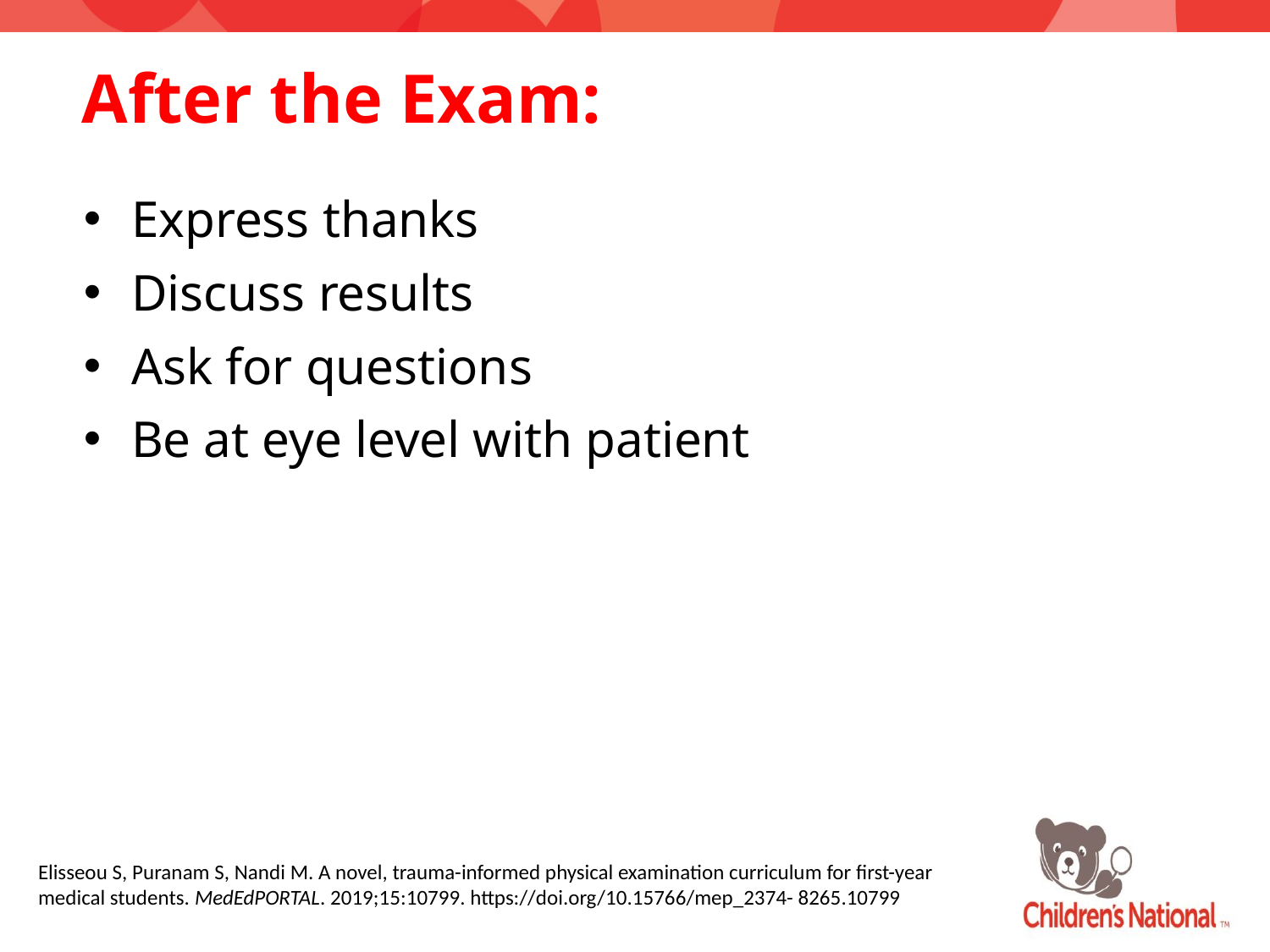

# After the Exam:
Express thanks
Discuss results
Ask for questions
Be at eye level with patient
Elisseou S, Puranam S, Nandi M. A novel, trauma-informed physical examination curriculum for first-year medical students. MedEdPORTAL. 2019;15:10799. https://doi.org/10.15766/mep_2374- 8265.10799

## Slide 53
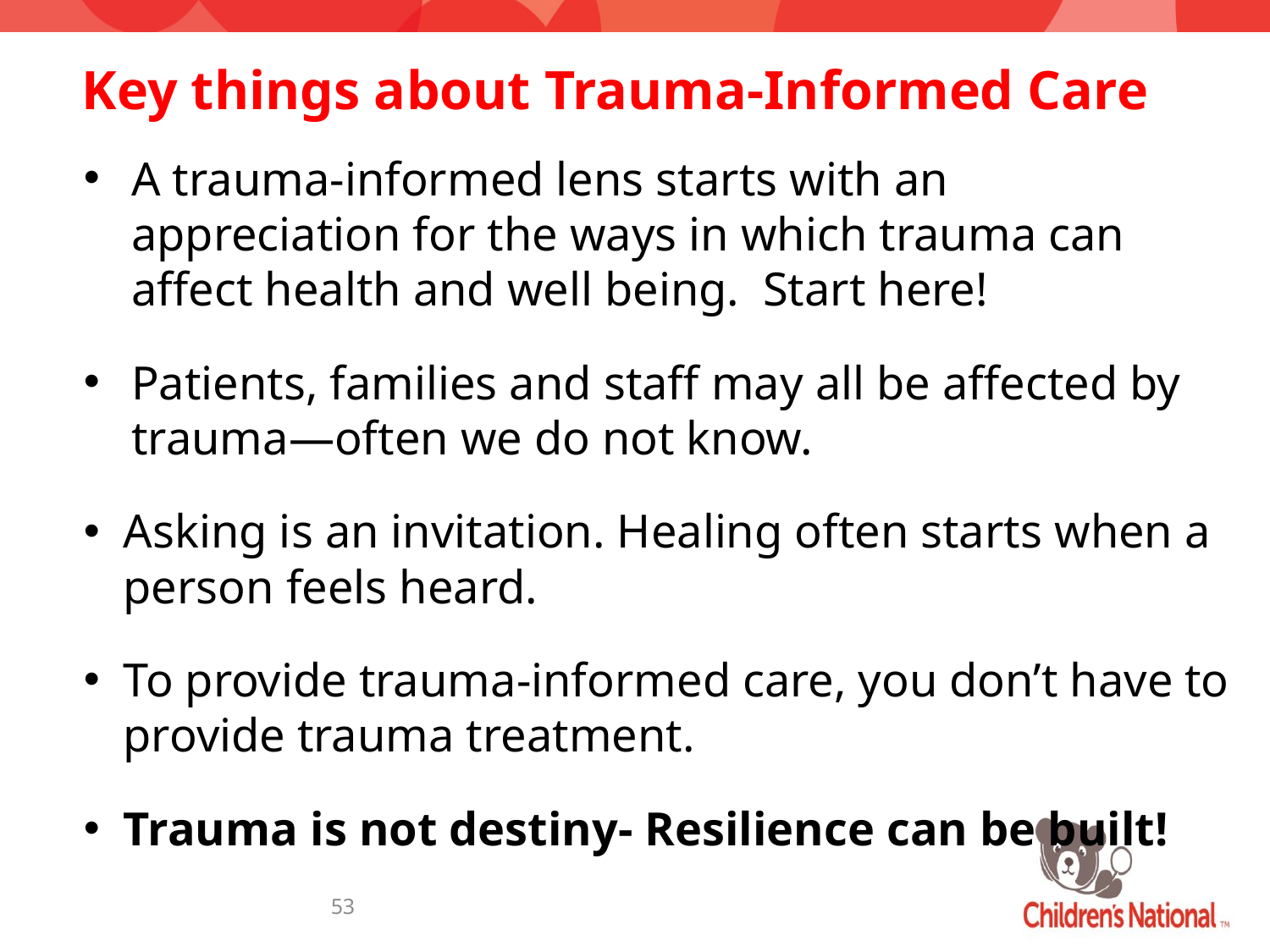

# Key things about Trauma-Informed Care
A trauma-informed lens starts with an appreciation for the ways in which trauma can affect health and well being. Start here!
Patients, families and staff may all be affected by trauma—often we do not know.
Asking is an invitation. Healing often starts when a person feels heard.
To provide trauma-informed care, you don’t have to provide trauma treatment.
Trauma is not destiny- Resilience can be built!
53

## Slide 54
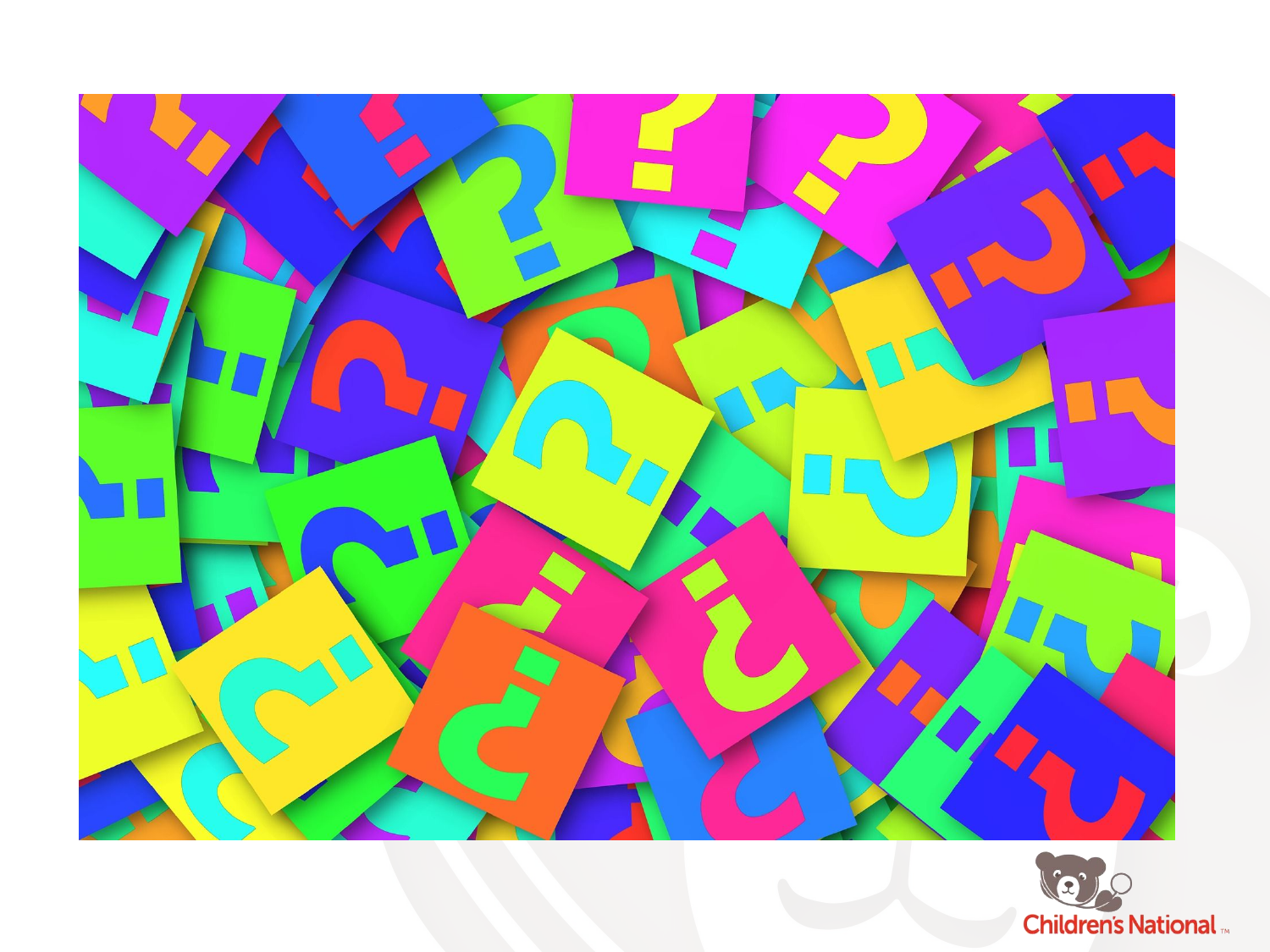

## Slide 55
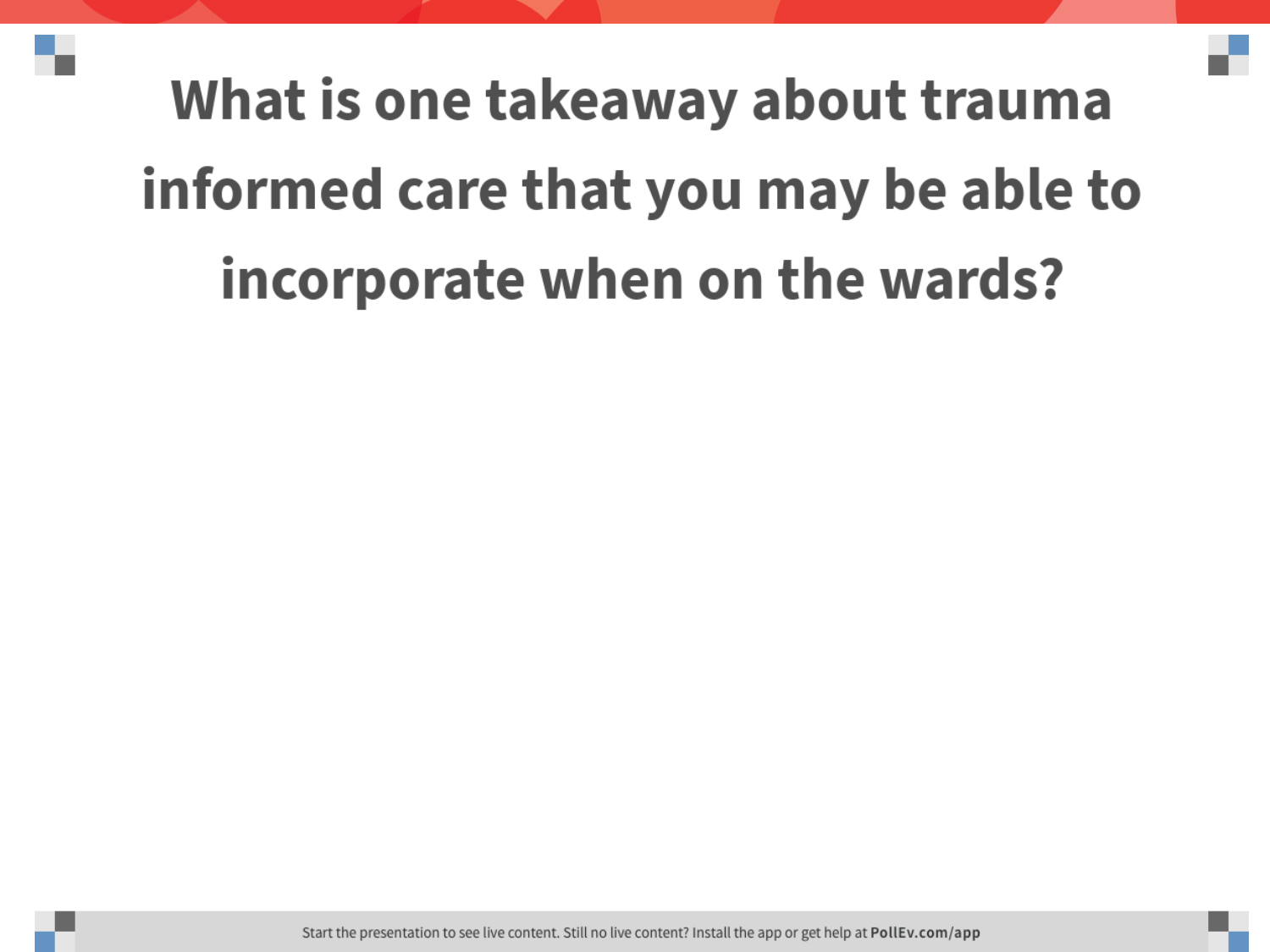

August 31, 2020

## Slide 56
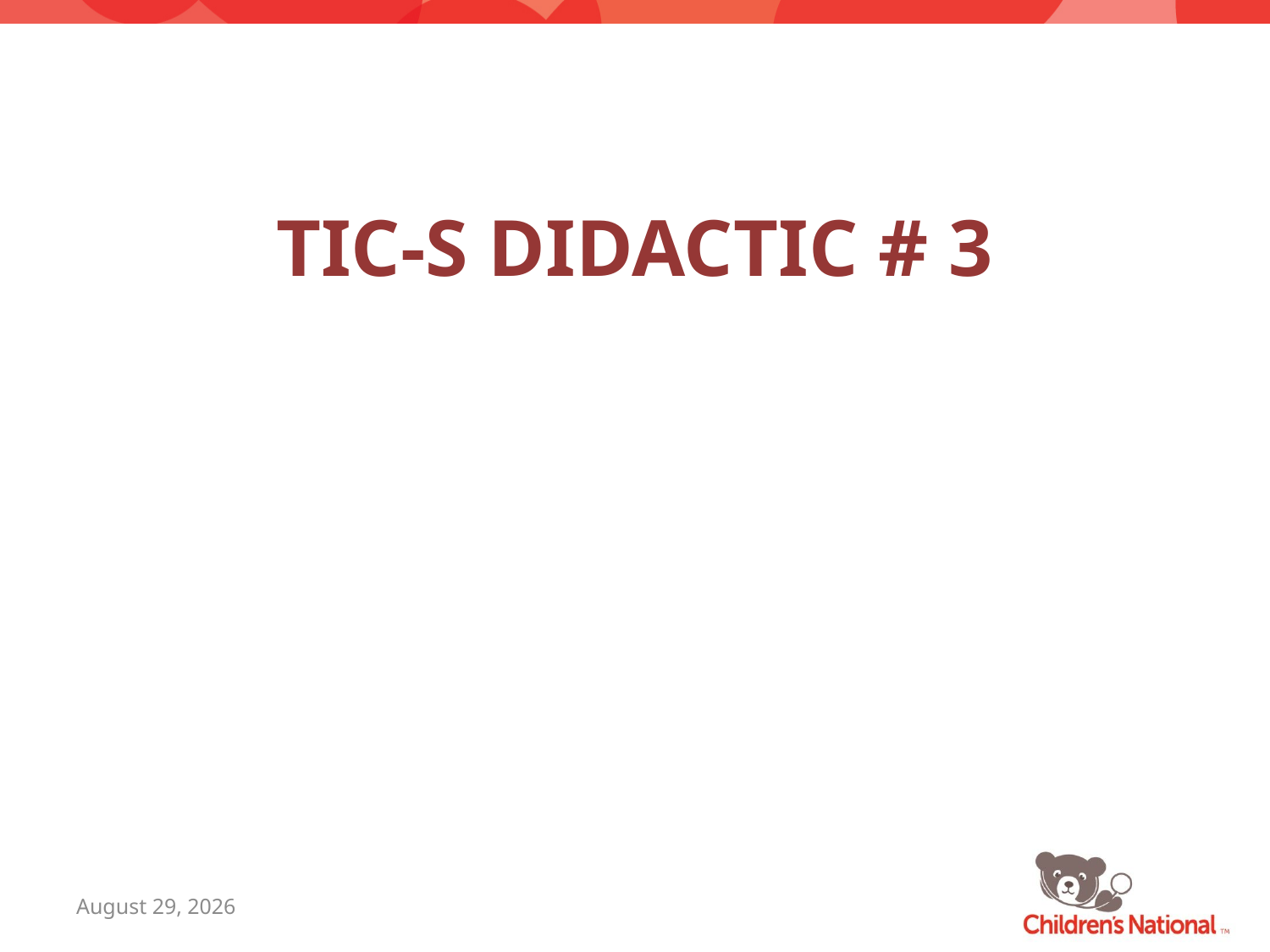

TIC-S DIDACTIC # 3
August 31, 2020

## Slide 57
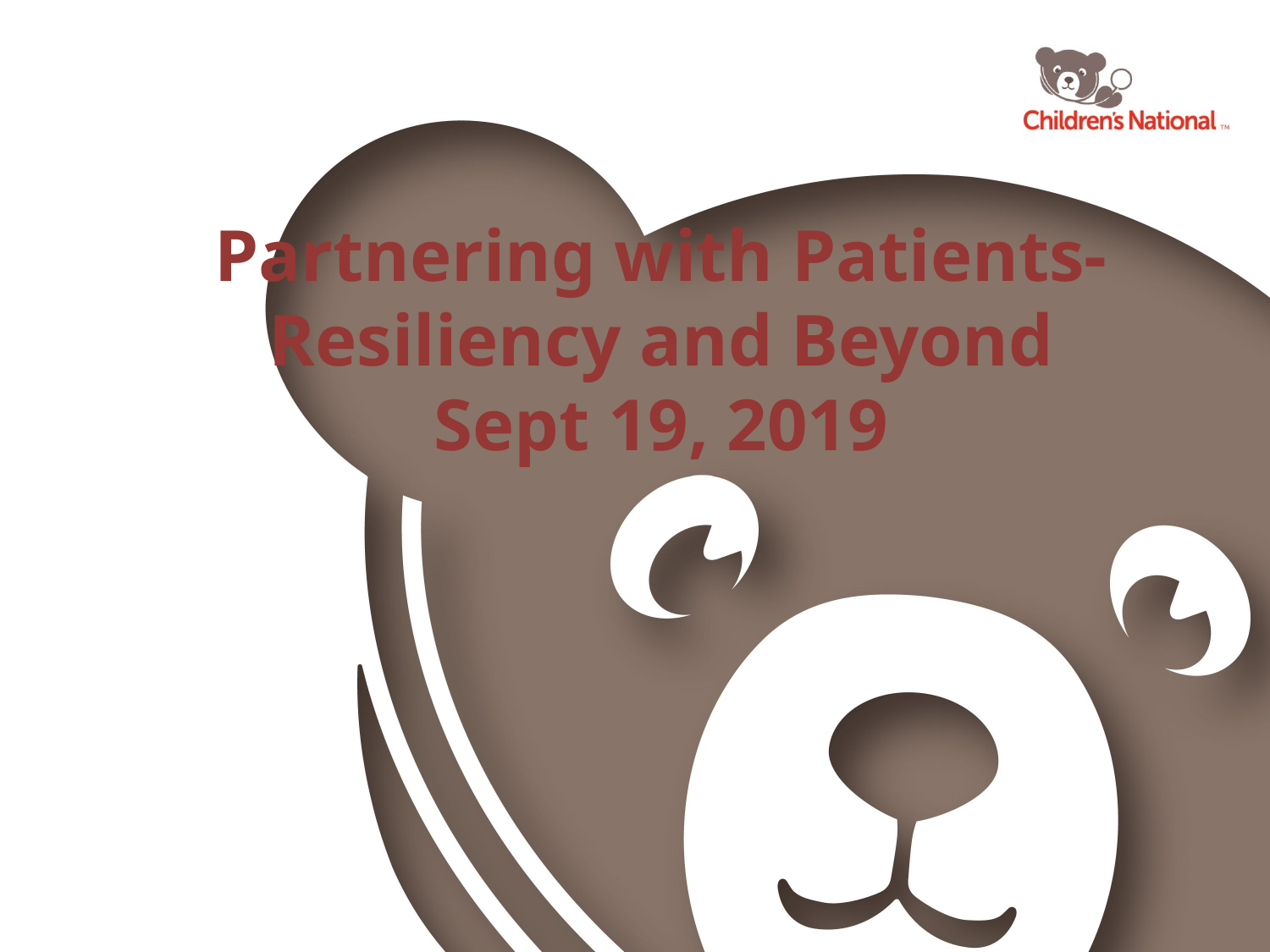

# Partnering with Patients- Resiliency and Beyond Sept 19, 2019

## Slide 58
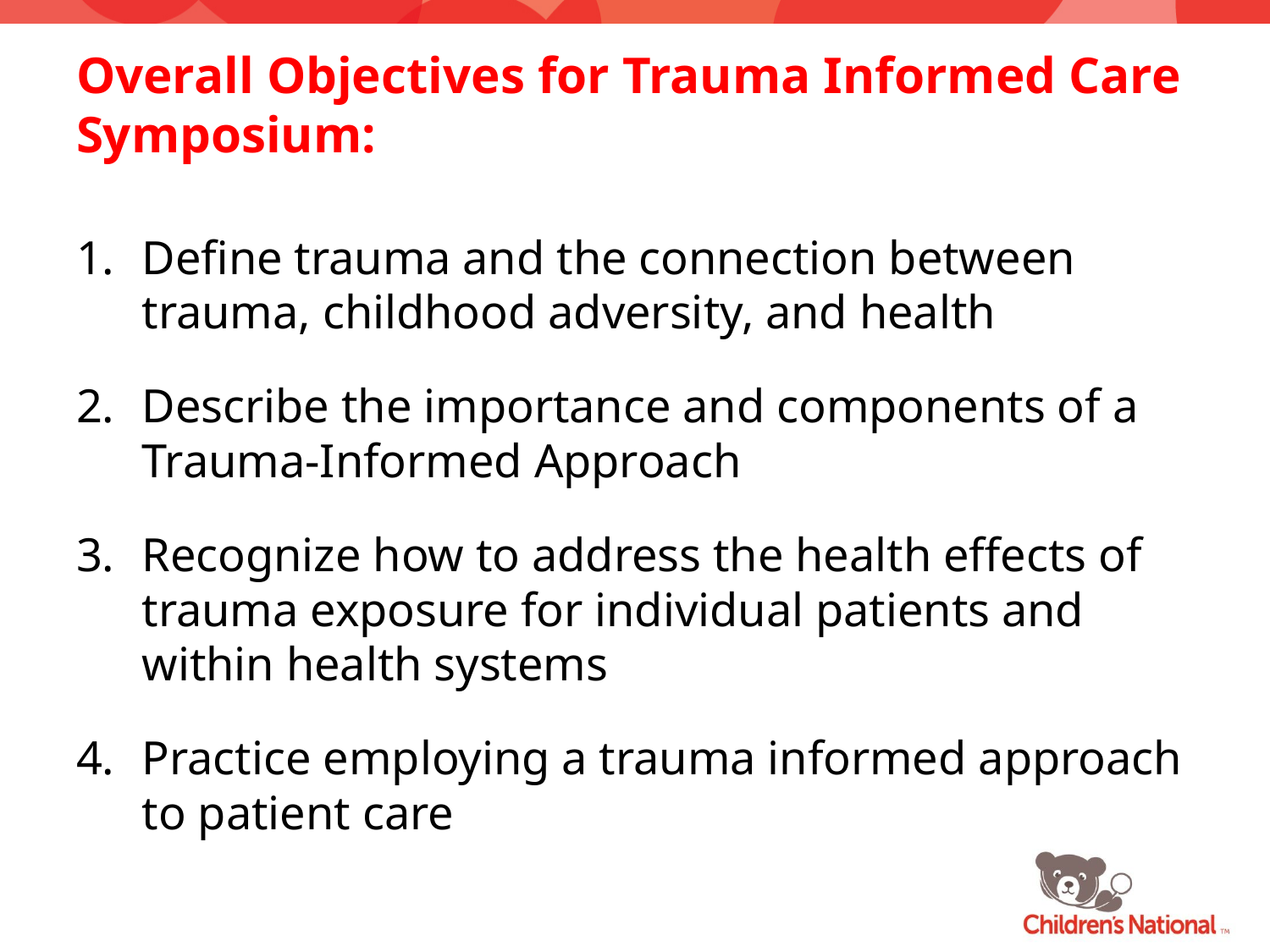

# Overall Objectives for Trauma Informed Care Symposium:
Define trauma and the connection between trauma, childhood adversity, and health
Describe the importance and components of a Trauma-Informed Approach
Recognize how to address the health effects of trauma exposure for individual patients and within health systems
Practice employing a trauma informed approach to patient care

## Slide 59
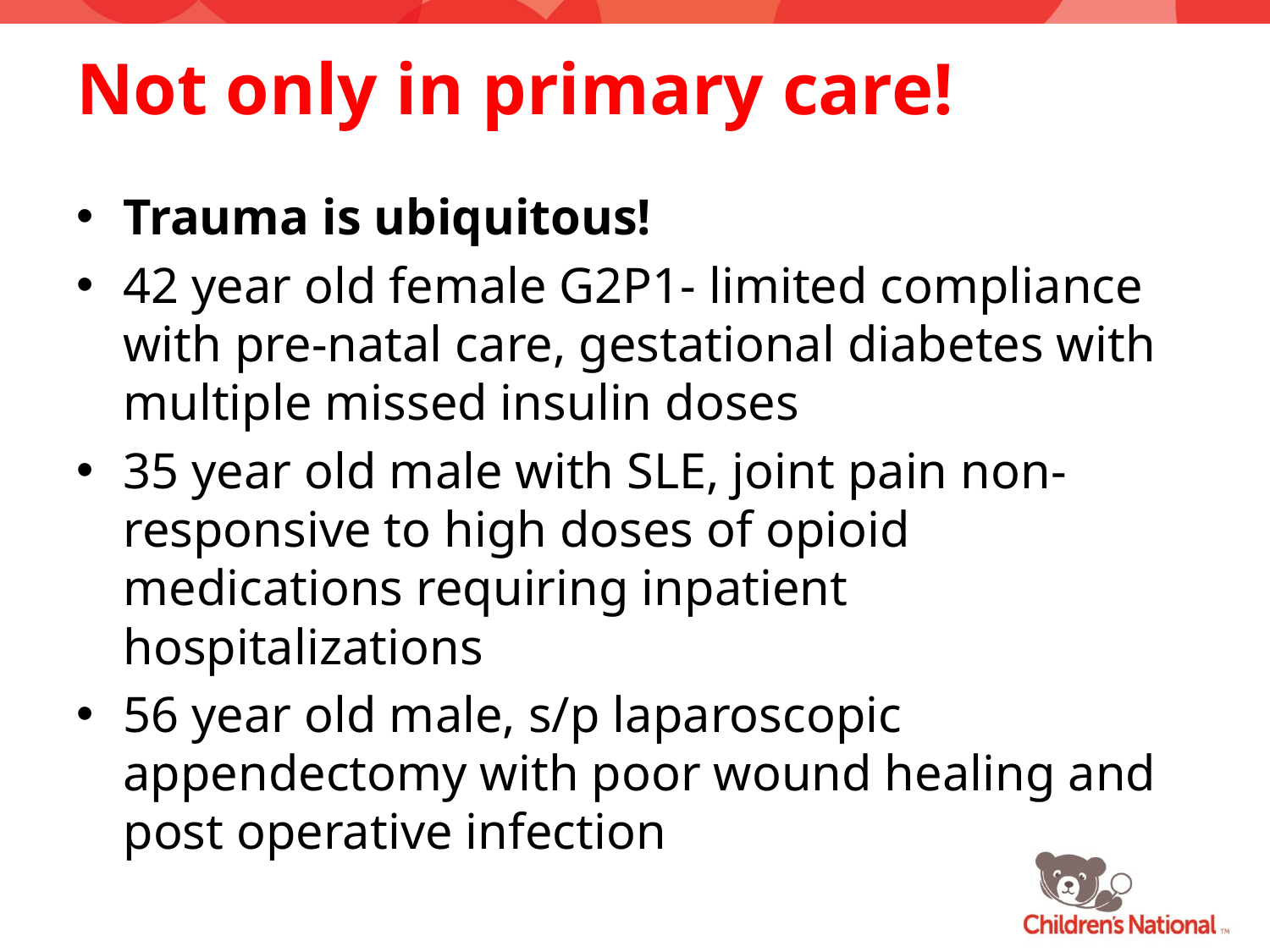

# Not only in primary care!
Trauma is ubiquitous!
42 year old female G2P1- limited compliance with pre-natal care, gestational diabetes with multiple missed insulin doses
35 year old male with SLE, joint pain non-responsive to high doses of opioid medications requiring inpatient hospitalizations
56 year old male, s/p laparoscopic appendectomy with poor wound healing and post operative infection

## Slide 60
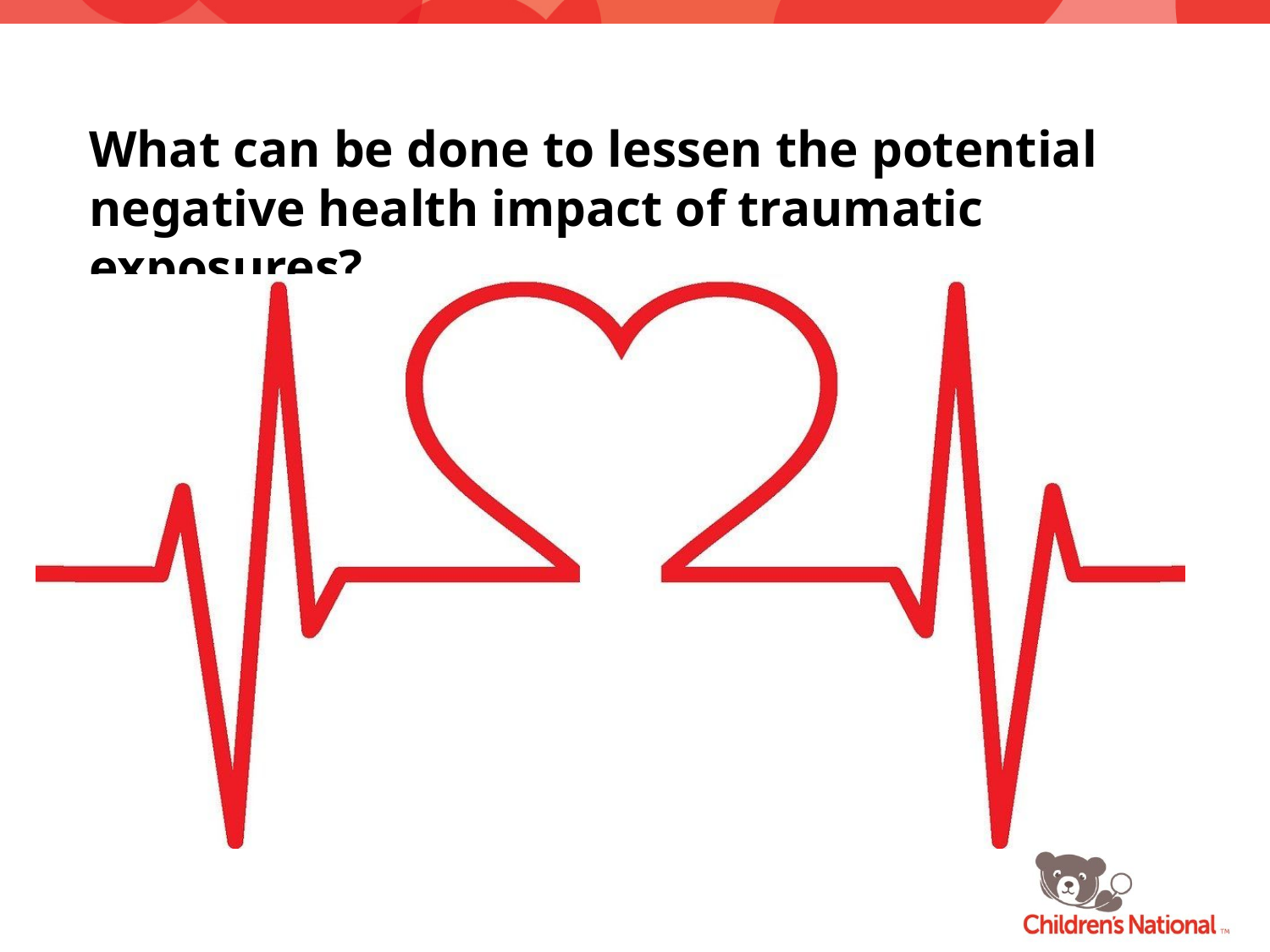

What can be done to lessen the potential negative health impact of traumatic exposures?

## Slide 61
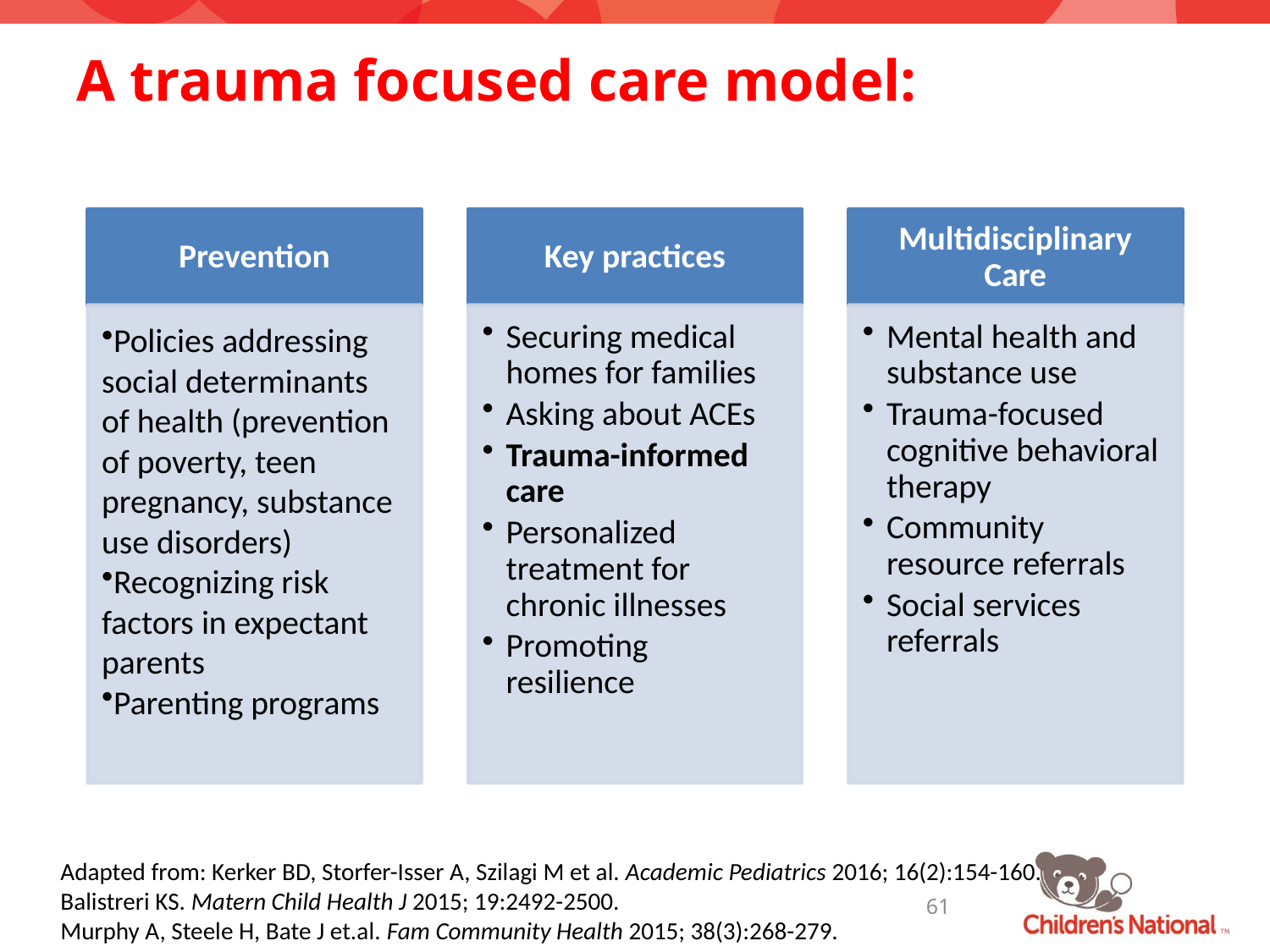

# A trauma focused care model:
Adapted from: Kerker BD, Storfer-Isser A, Szilagi M et al. Academic Pediatrics 2016; 16(2):154-160.
Balistreri KS. Matern Child Health J 2015; 19:2492-2500.
Murphy A, Steele H, Bate J et.al. Fam Community Health 2015; 38(3):268-279.
61

## Slide 62
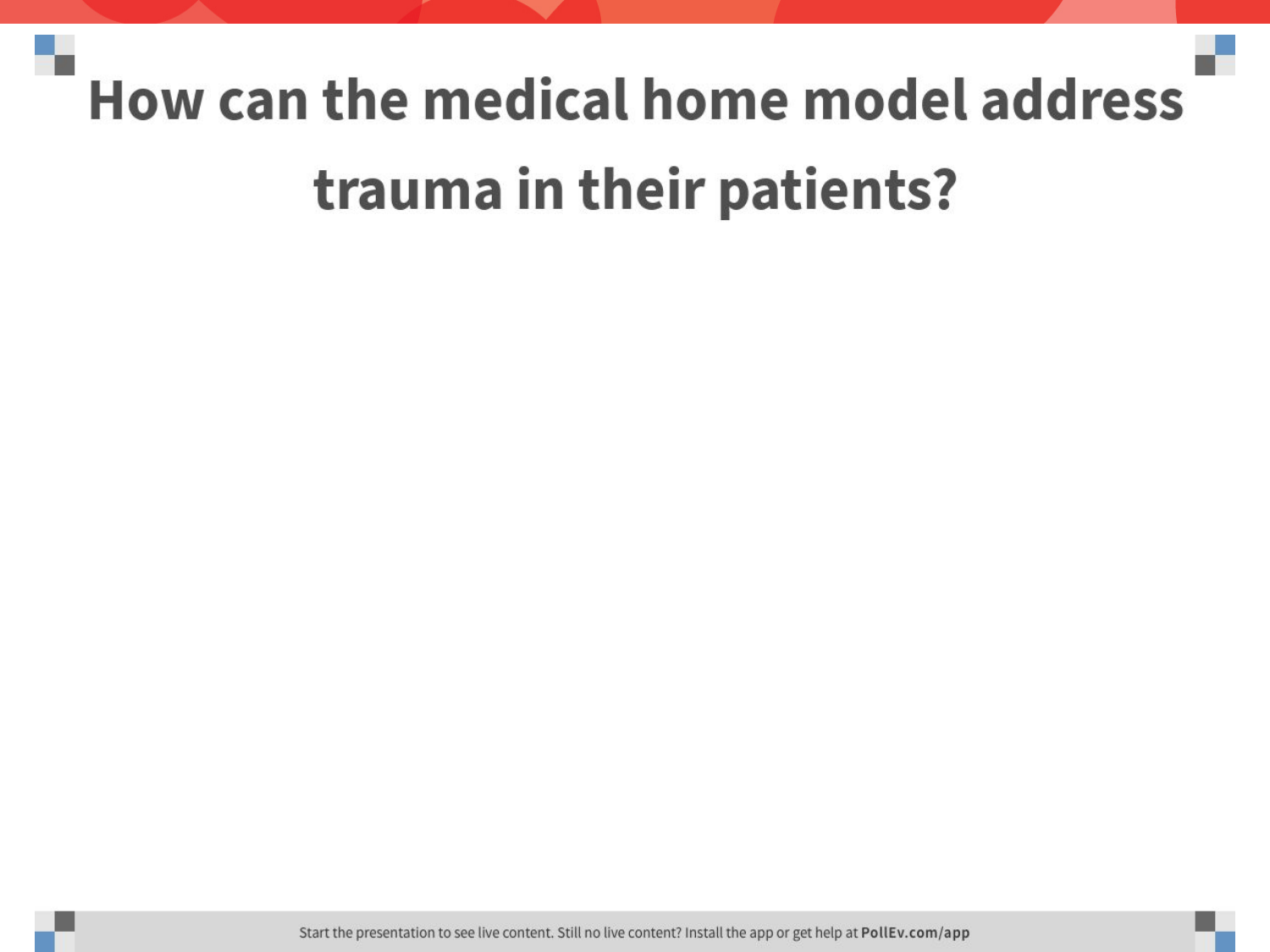

August 31, 2020

## Slide 63
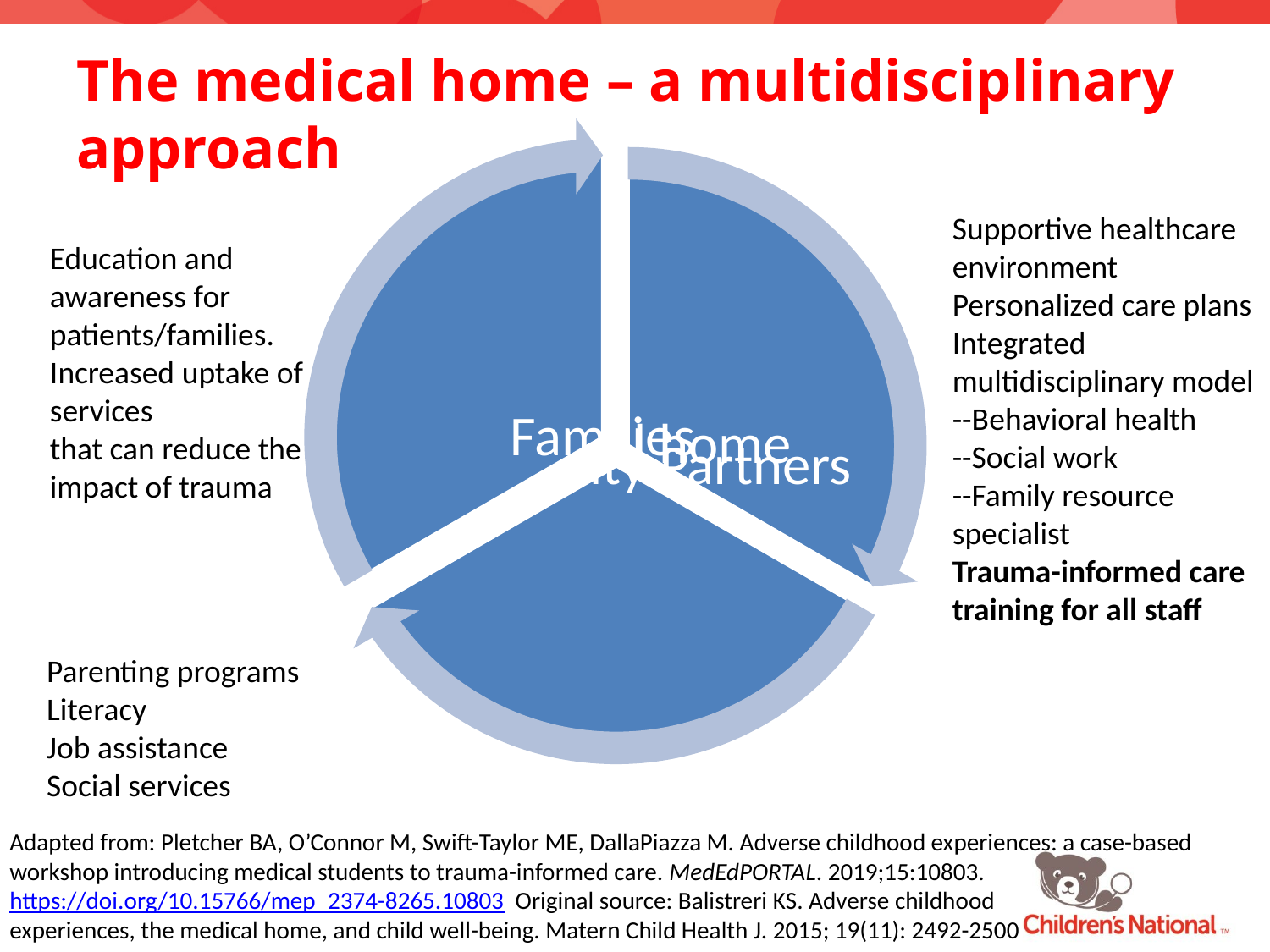

# The medical home – a multidisciplinary approach
Supportive healthcare environment
Personalized care plans
Integrated multidisciplinary model
--Behavioral health
--Social work
--Family resource specialist
Trauma-informed care training for all staff
Education and awareness for patients/families.
Increased uptake of services
that can reduce the impact of trauma
Parenting programs
Literacy
Job assistance
Social services
Adapted from: Pletcher BA, O’Connor M, Swift-Taylor ME, DallaPiazza M. Adverse childhood experiences: a case-based workshop introducing medical students to trauma-informed care. MedEdPORTAL. 2019;15:10803. https://doi.org/10.15766/mep_2374-8265.10803 Original source: Balistreri KS. Adverse childhood
experiences, the medical home, and child well-being. Matern Child Health J. 2015; 19(11): 2492-2500

## Slide 64
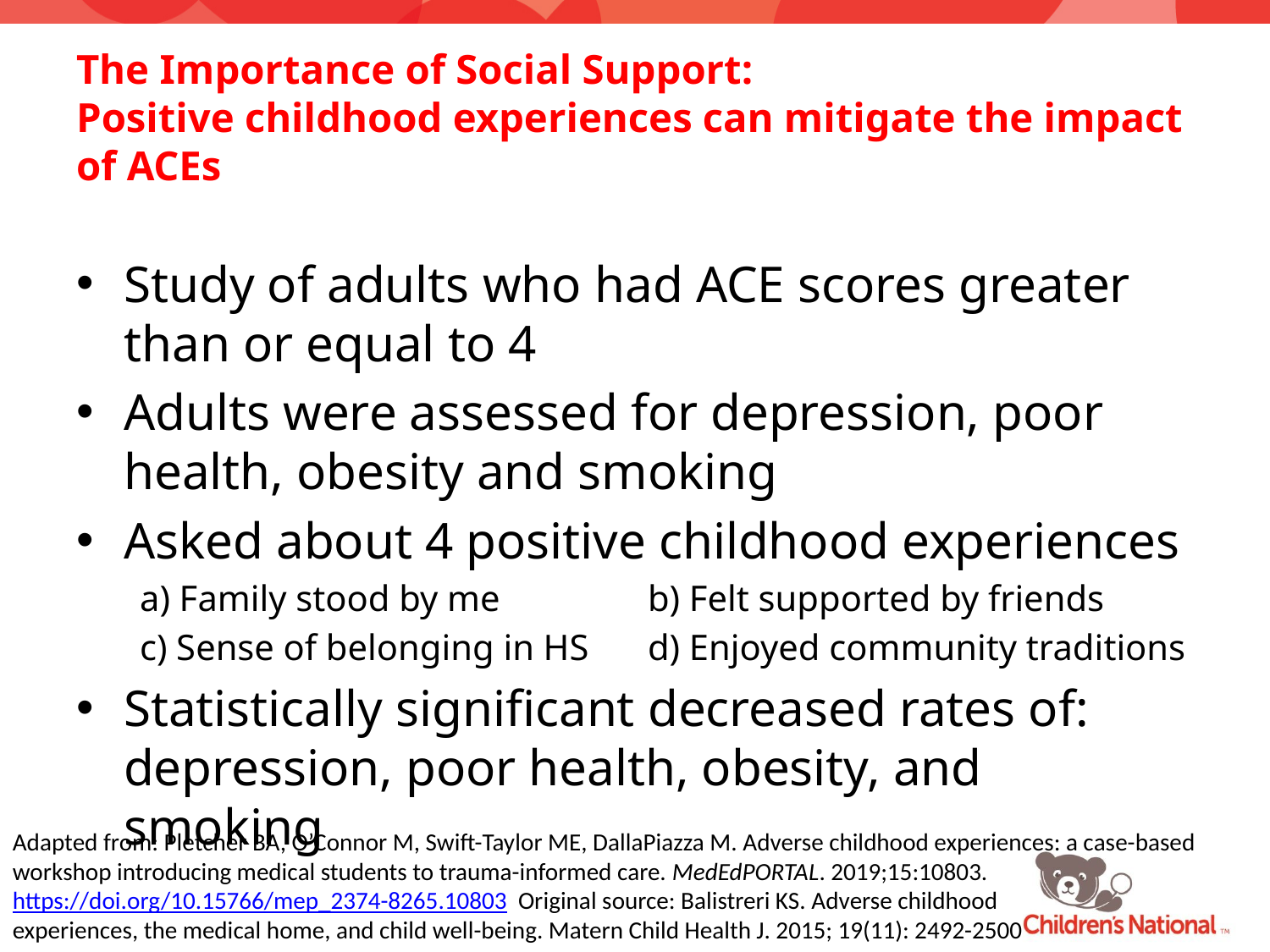

# The Importance of Social Support: Positive childhood experiences can mitigate the impact of ACEs
Study of adults who had ACE scores greater than or equal to 4
Adults were assessed for depression, poor health, obesity and smoking
Asked about 4 positive childhood experiences
a) Family stood by me		b) Felt supported by friends
c) Sense of belonging in HS 	d) Enjoyed community traditions
Statistically significant decreased rates of: depression, poor health, obesity, and smoking
Adapted from: Pletcher BA, O’Connor M, Swift-Taylor ME, DallaPiazza M. Adverse childhood experiences: a case-based workshop introducing medical students to trauma-informed care. MedEdPORTAL. 2019;15:10803. https://doi.org/10.15766/mep_2374-8265.10803 Original source: Balistreri KS. Adverse childhood
experiences, the medical home, and child well-being. Matern Child Health J. 2015; 19(11): 2492-2500

## Slide 65
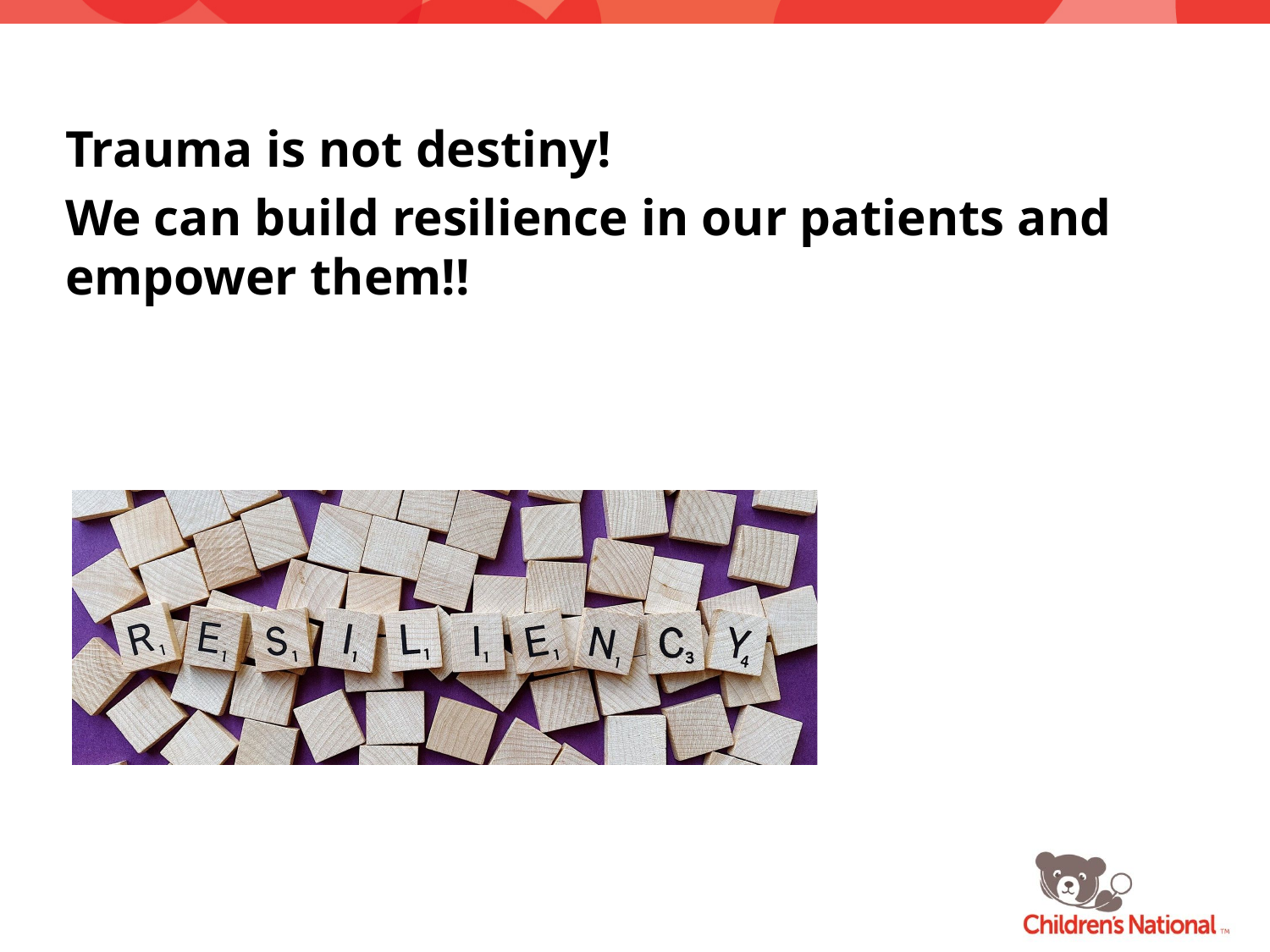

Trauma is not destiny!
We can build resilience in our patients and empower them!!

## Slide 66
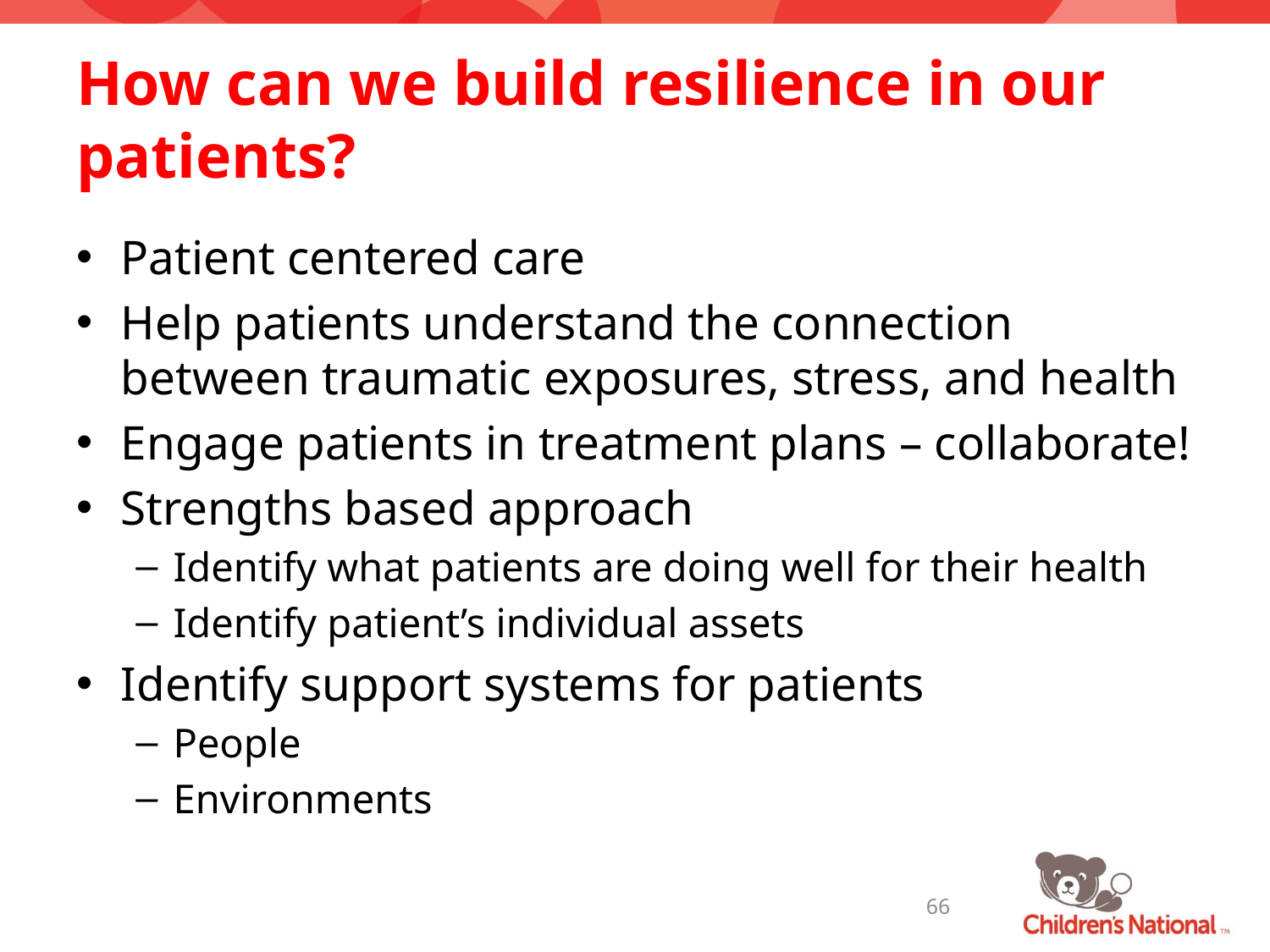

# How can we build resilience in our patients?
Patient centered care
Help patients understand the connection between traumatic exposures, stress, and health
Engage patients in treatment plans – collaborate!
Strengths based approach
Identify what patients are doing well for their health
Identify patient’s individual assets
Identify support systems for patients
People
Environments
66

## Slide 67
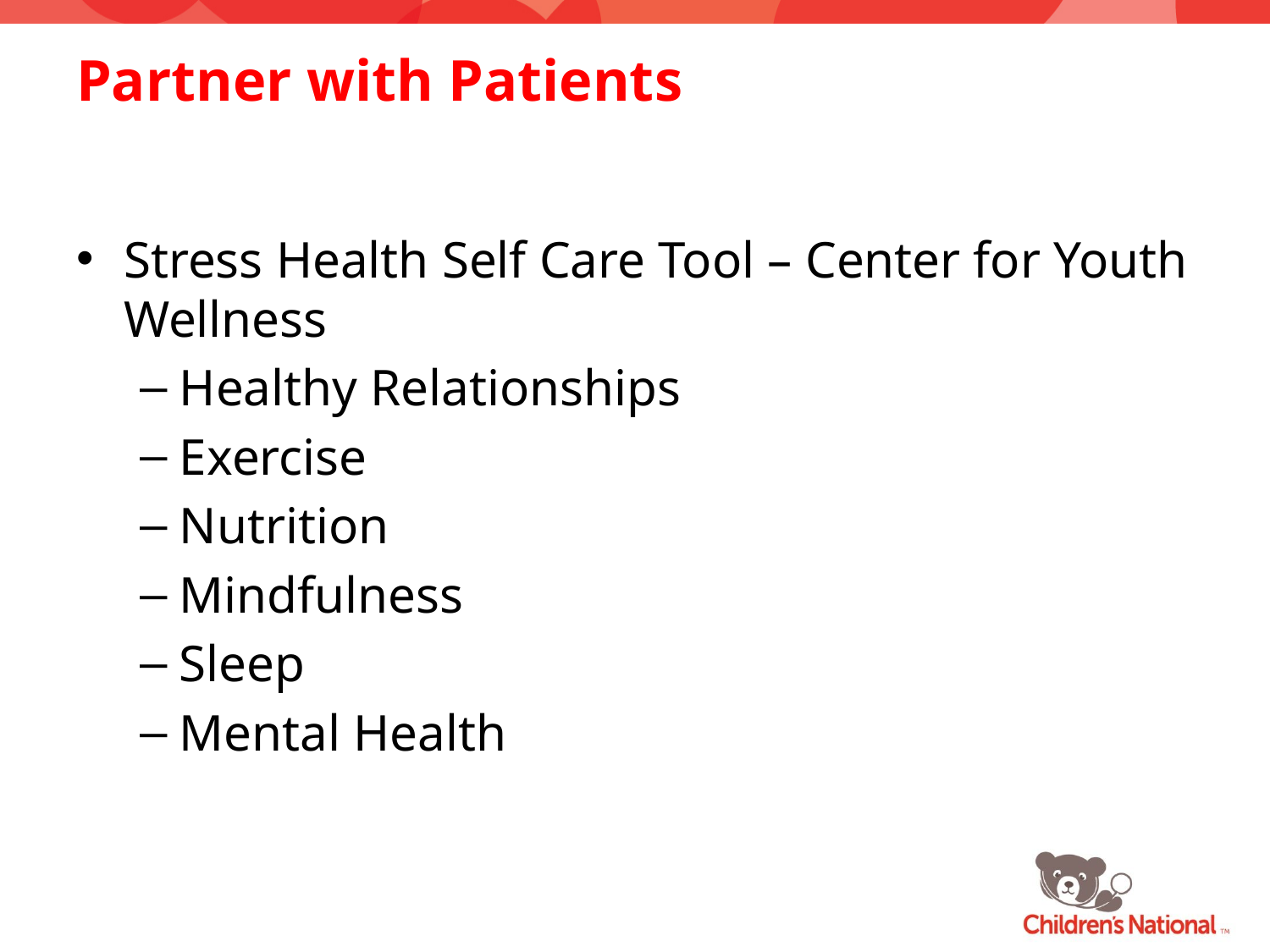

# Partner with Patients
Stress Health Self Care Tool – Center for Youth Wellness
Healthy Relationships
Exercise
Nutrition
Mindfulness
Sleep
Mental Health

## Slide 68
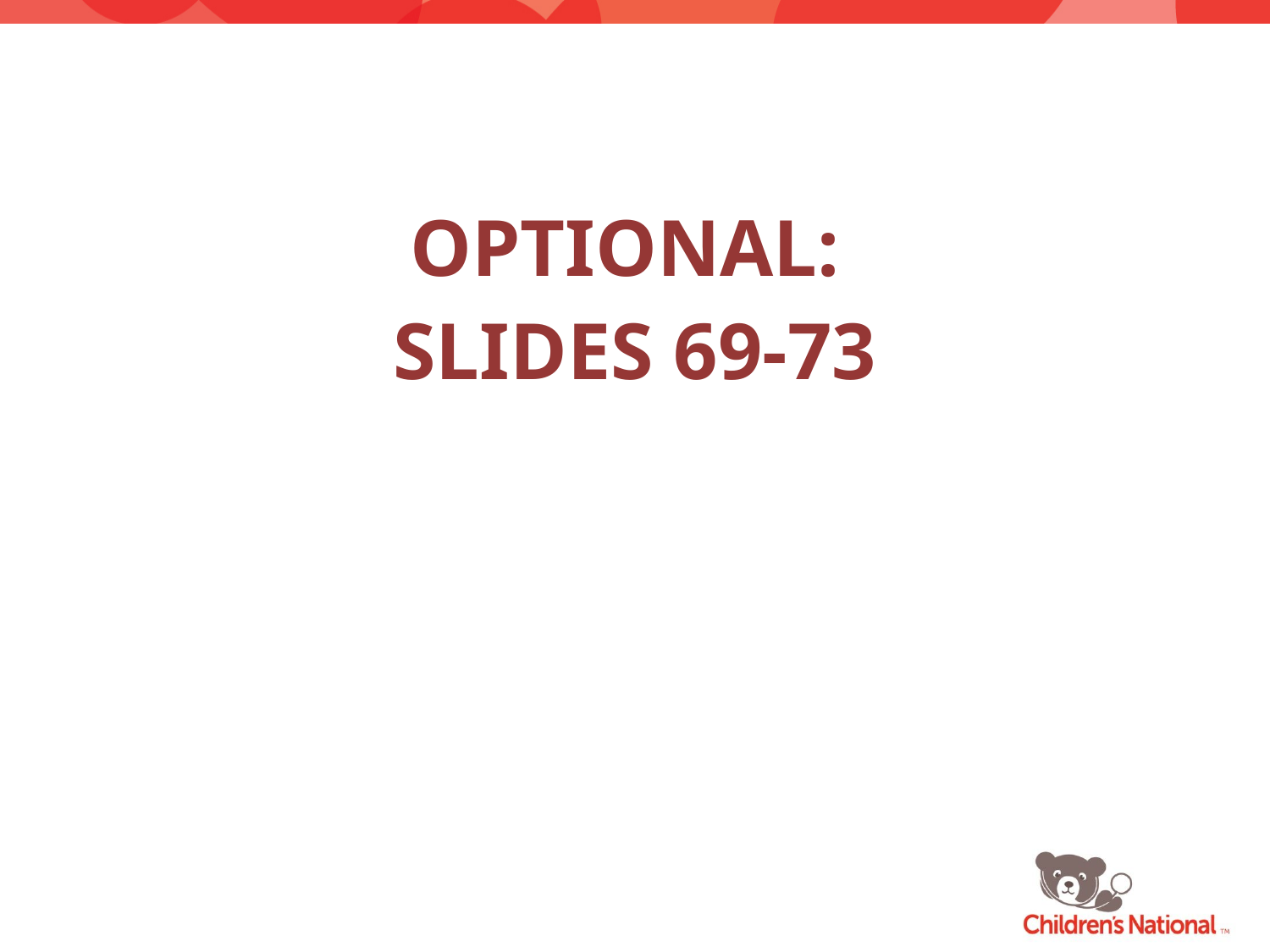

OPTIONAL:
SLIDES 69-73

## Slide 69
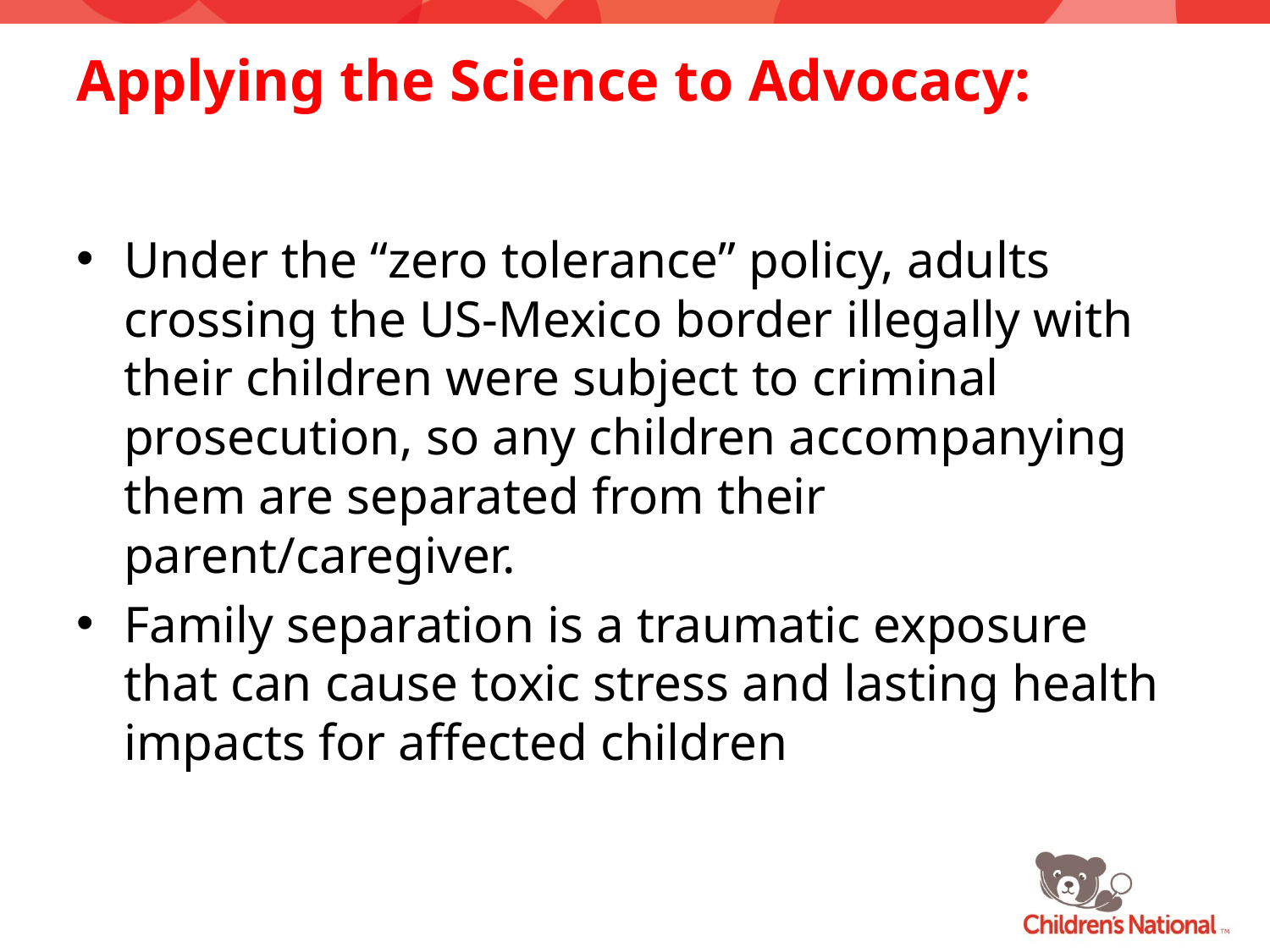

# Applying the Science to Advocacy:
Under the “zero tolerance” policy, adults crossing the US-Mexico border illegally with their children were subject to criminal prosecution, so any children accompanying them are separated from their parent/caregiver.
Family separation is a traumatic exposure that can cause toxic stress and lasting health impacts for affected children

## Slide 70
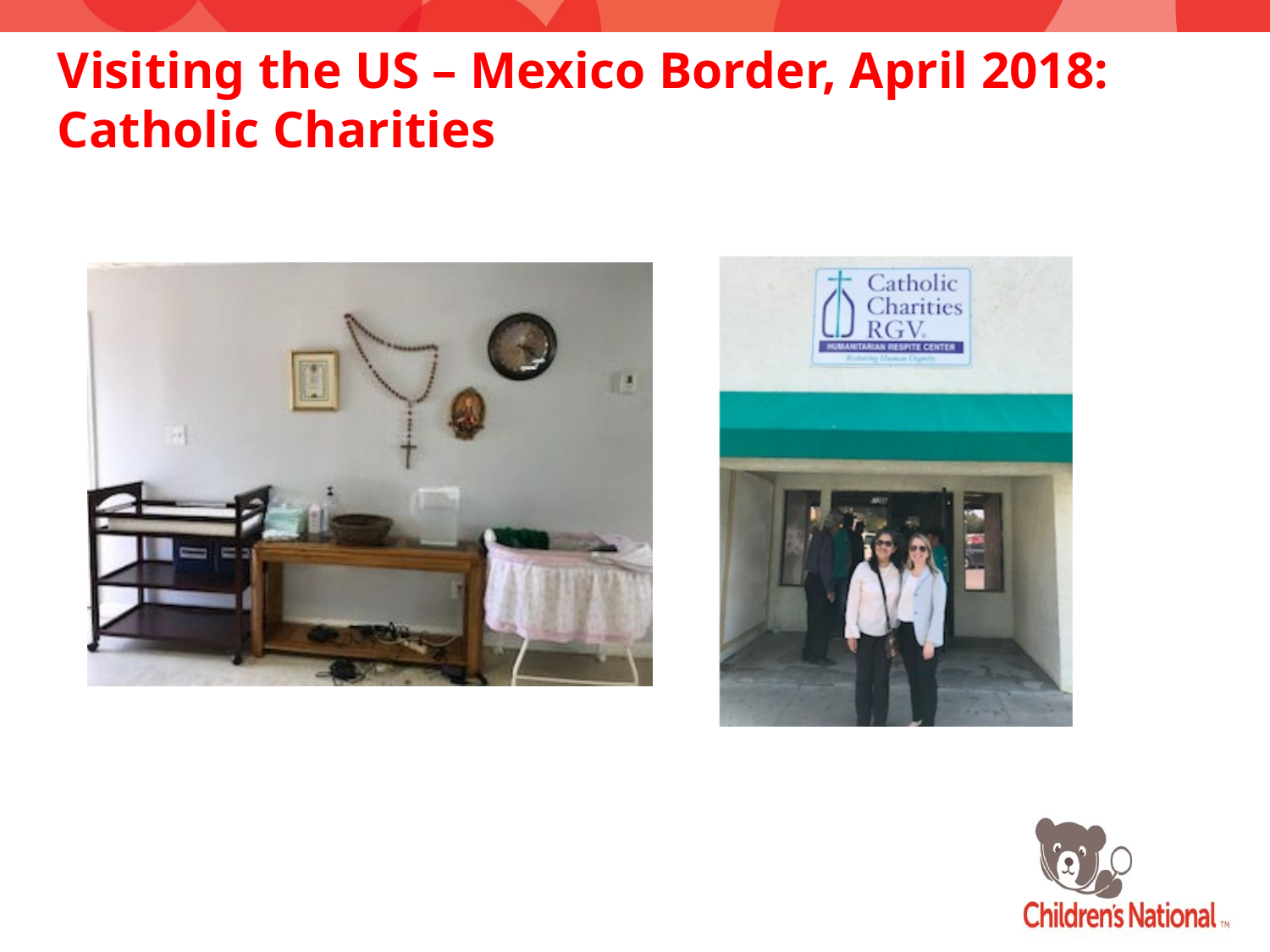

Visiting the US – Mexico Border, April 2018:
Catholic Charities

## Slide 71
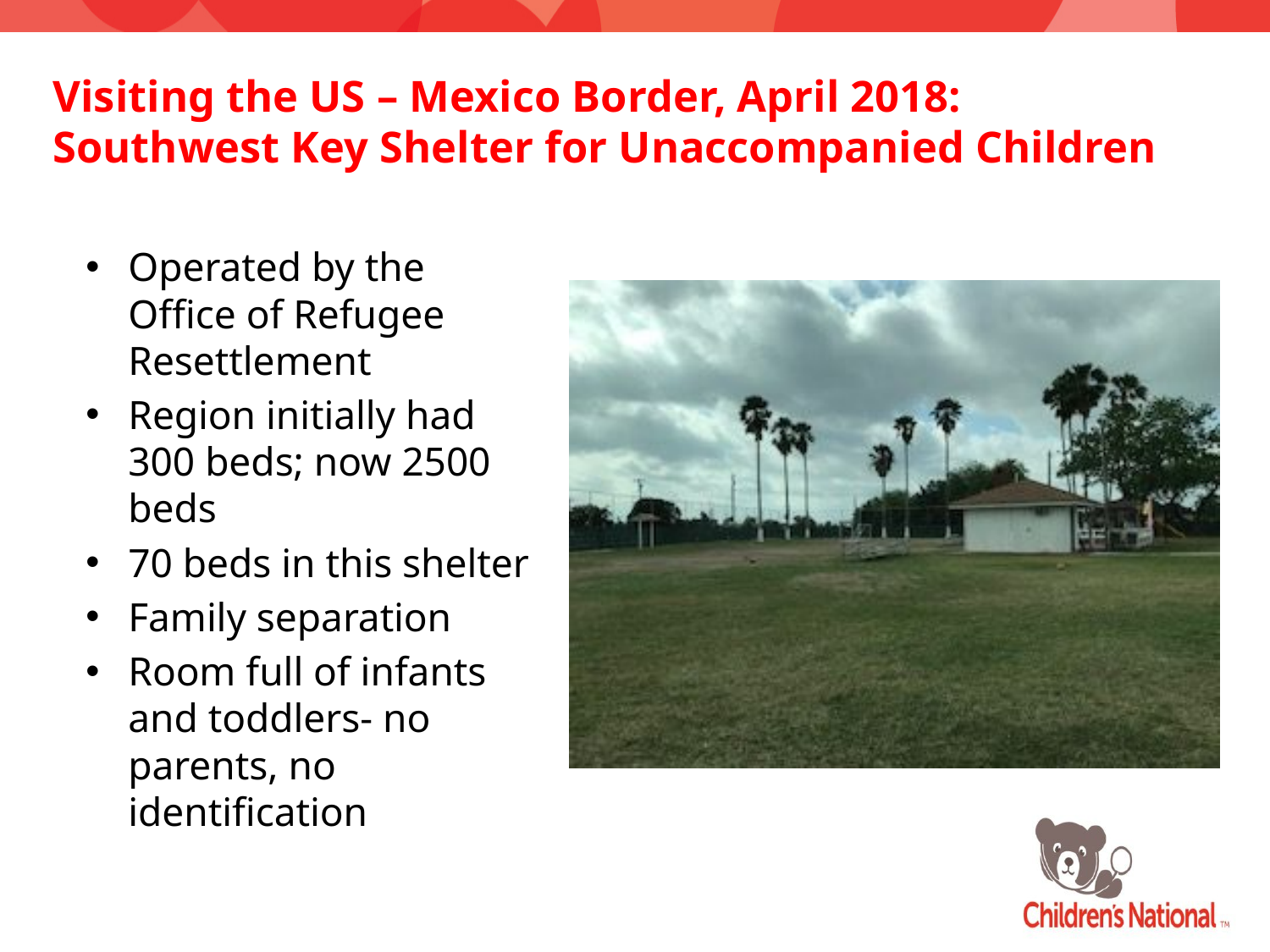

Visiting the US – Mexico Border, April 2018:
Southwest Key Shelter for Unaccompanied Children
Operated by the Office of Refugee Resettlement
Region initially had 300 beds; now 2500 beds
70 beds in this shelter
Family separation
Room full of infants and toddlers- no parents, no identification

## Slide 72
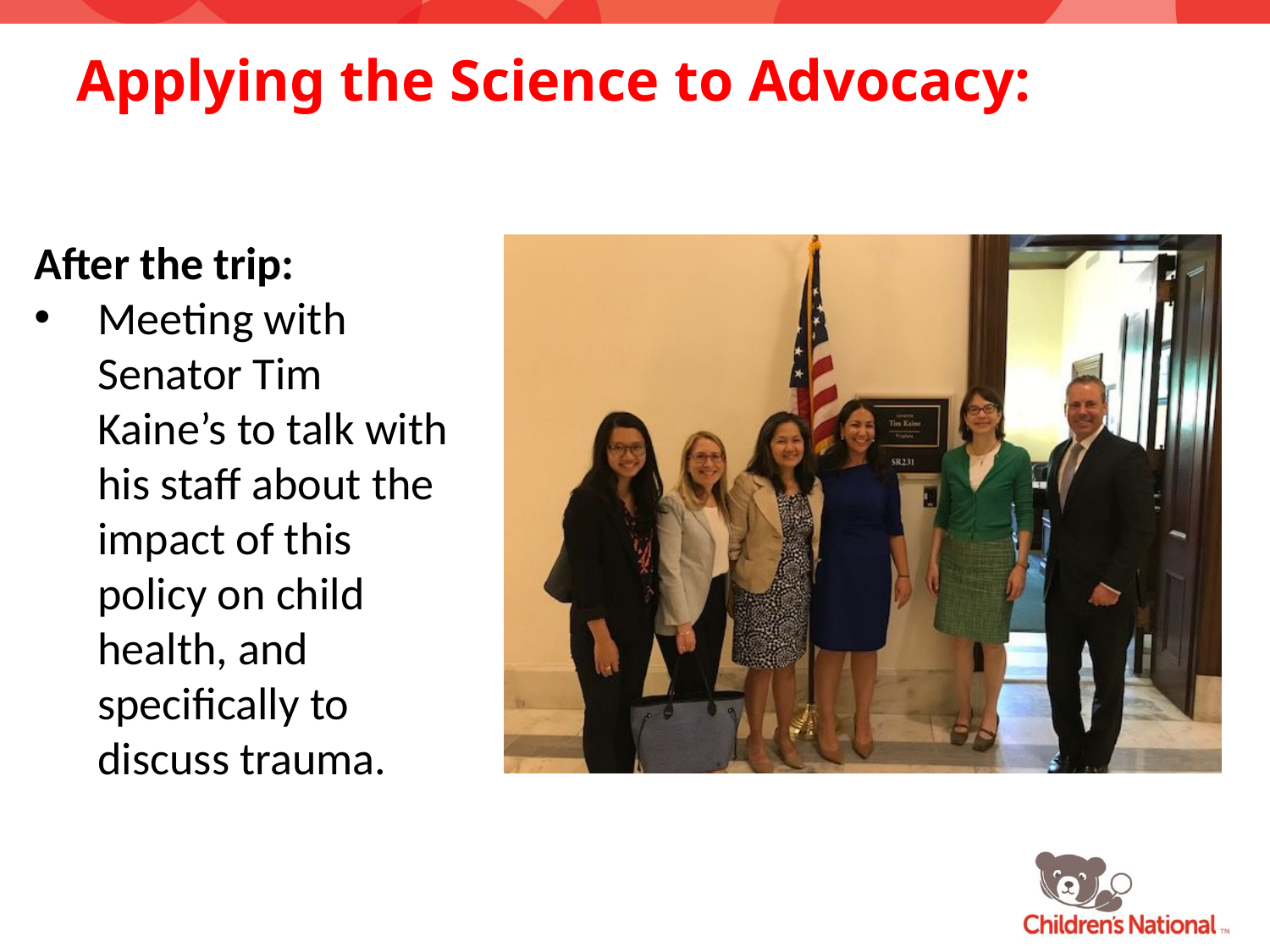

# Applying the Science to Advocacy:
After the trip:
Meeting with Senator Tim Kaine’s to talk with his staff about the impact of this policy on child health, and specifically to discuss trauma.

## Slide 73
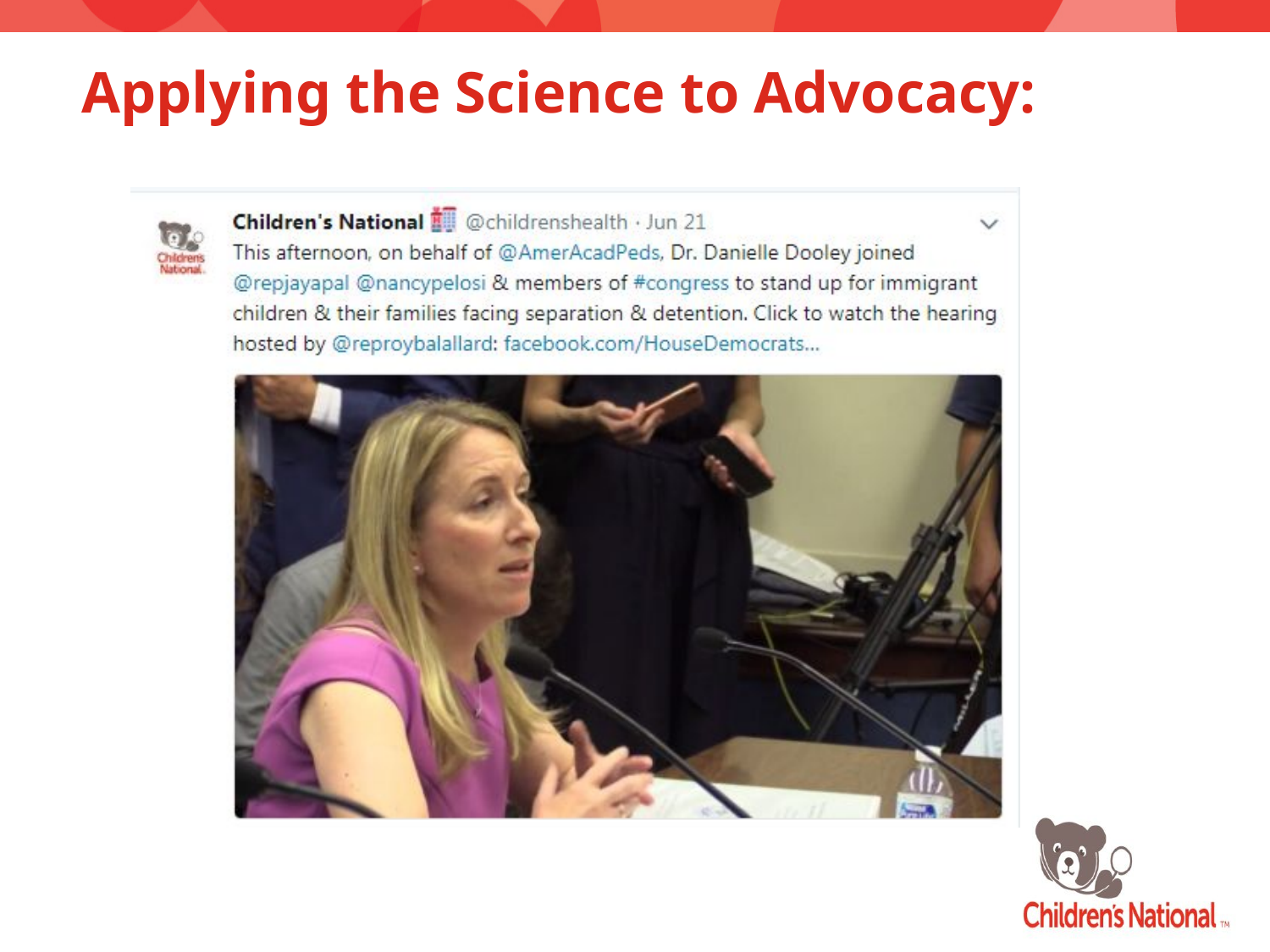

# Applying the Science to Advocacy:

## Slide 74
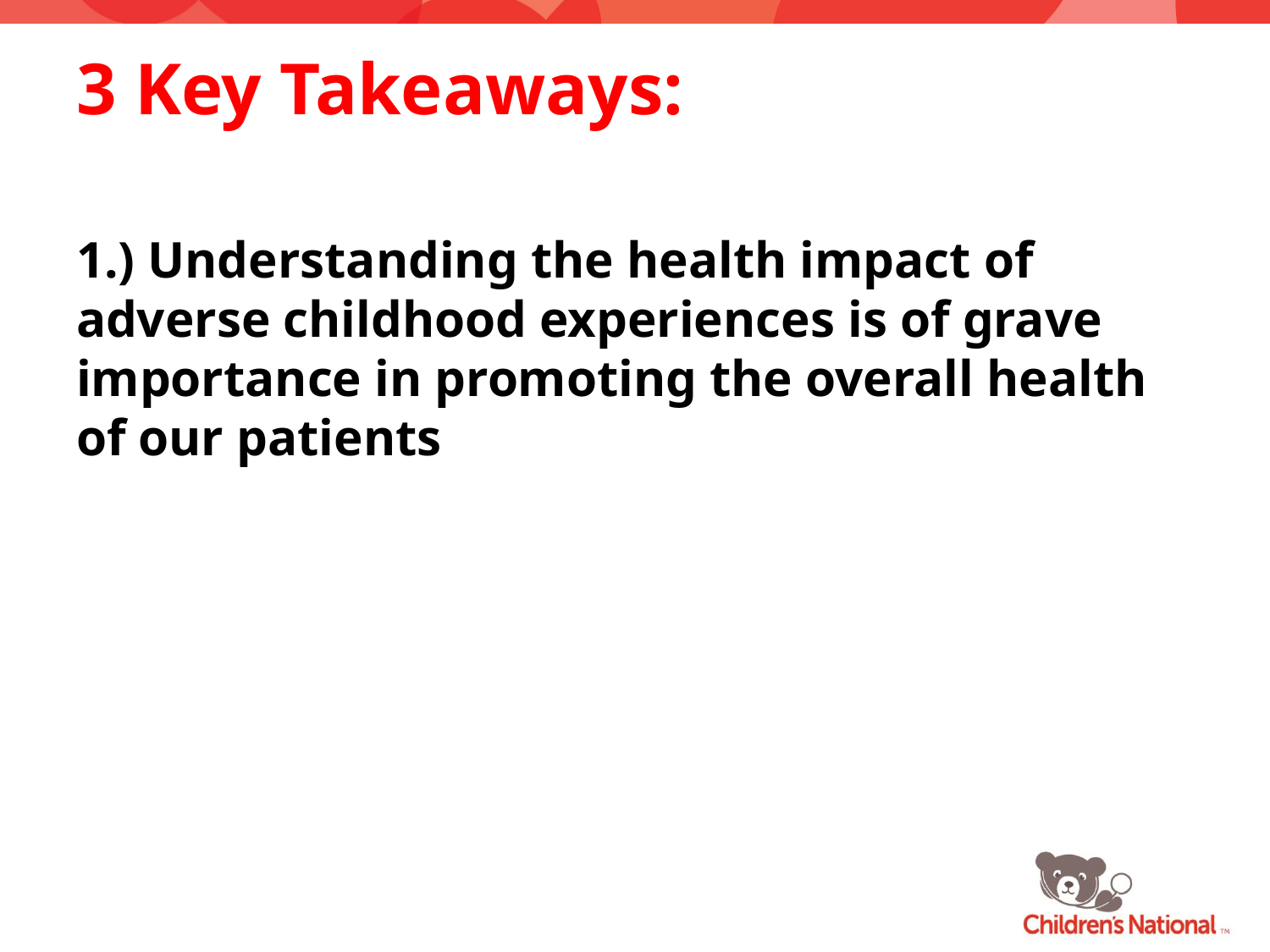

# 3 Key Takeaways:
1.) Understanding the health impact of adverse childhood experiences is of grave importance in promoting the overall health of our patients

## Slide 75
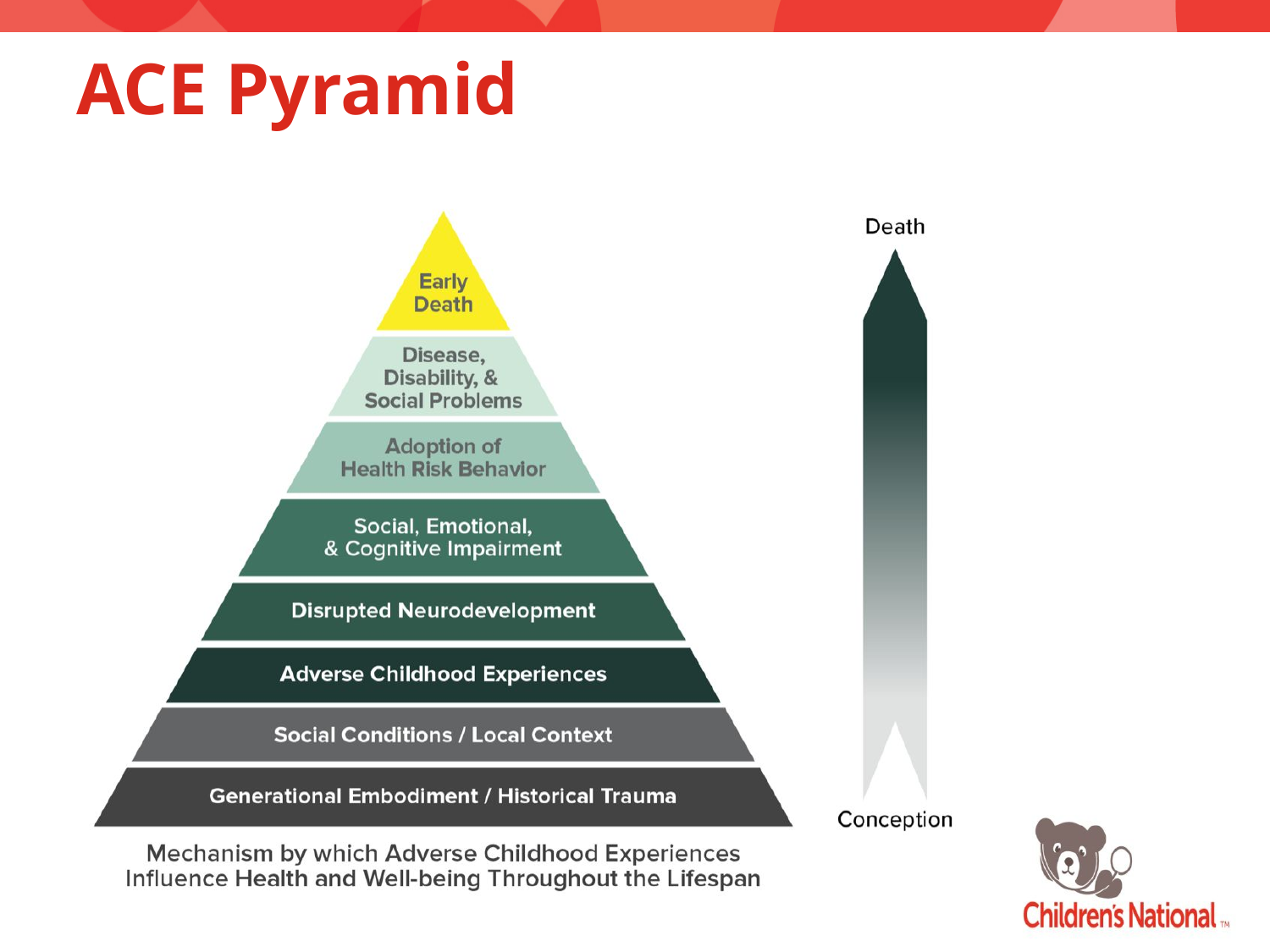

# ACE Pyramid

## Slide 76
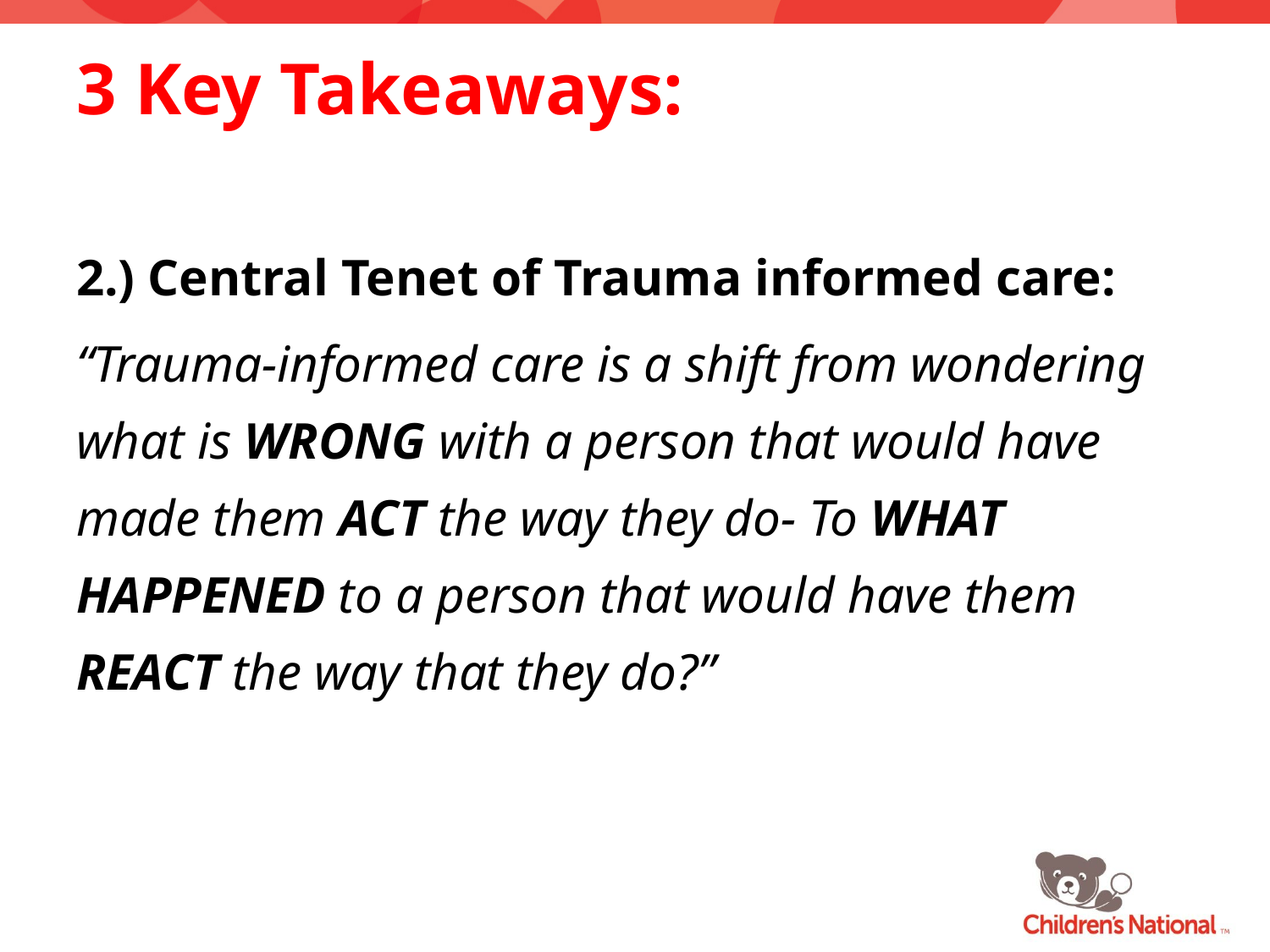

# 3 Key Takeaways:
2.) Central Tenet of Trauma informed care:
“Trauma-informed care is a shift from wondering what is WRONG with a person that would have made them ACT the way they do- To WHAT HAPPENED to a person that would have them REACT the way that they do?”

## Slide 77
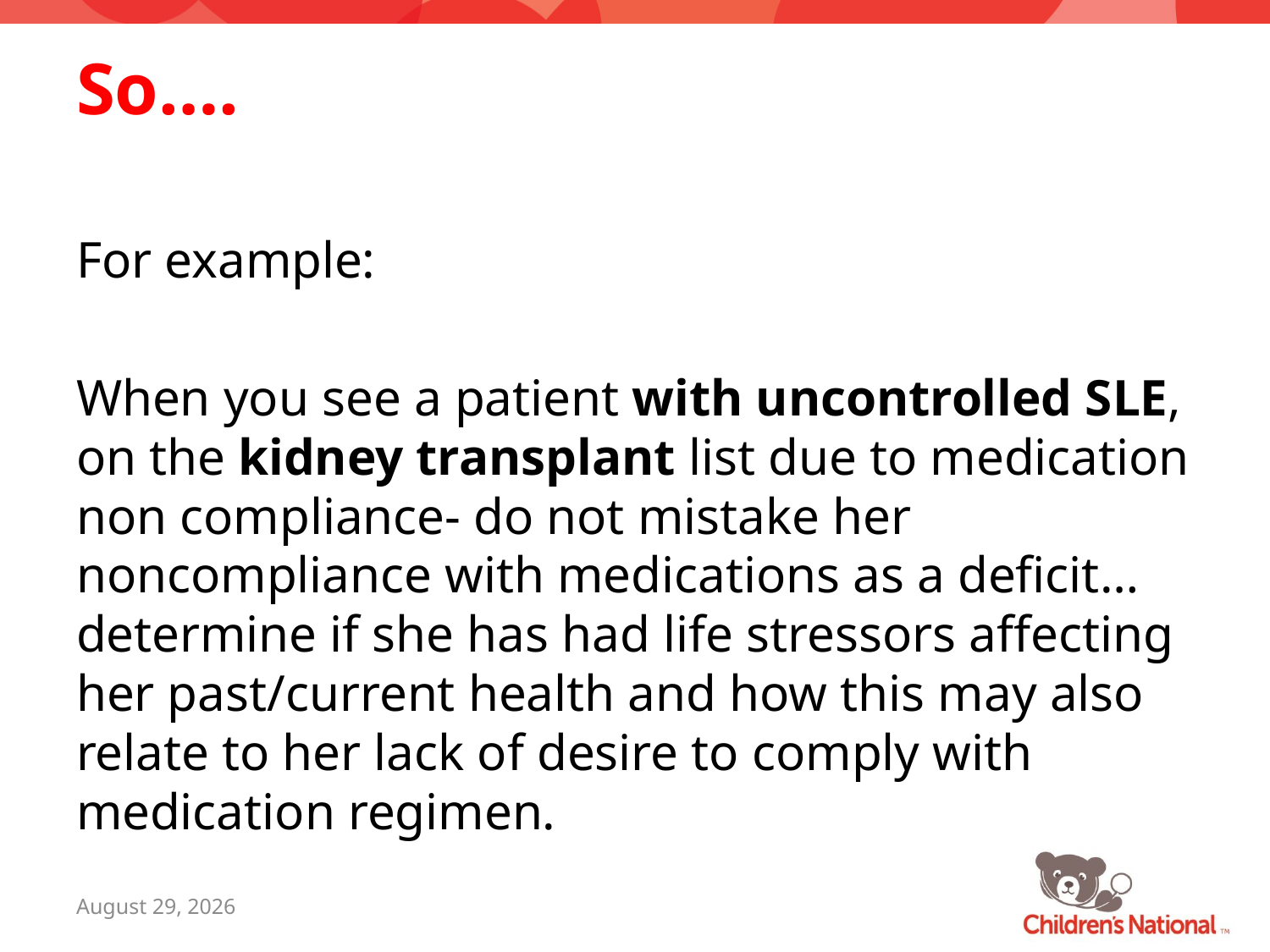

# So….
For example:
When you see a patient with uncontrolled SLE, on the kidney transplant list due to medication non compliance- do not mistake her noncompliance with medications as a deficit… determine if she has had life stressors affecting her past/current health and how this may also relate to her lack of desire to comply with medication regimen.
August 31, 2020

## Slide 78
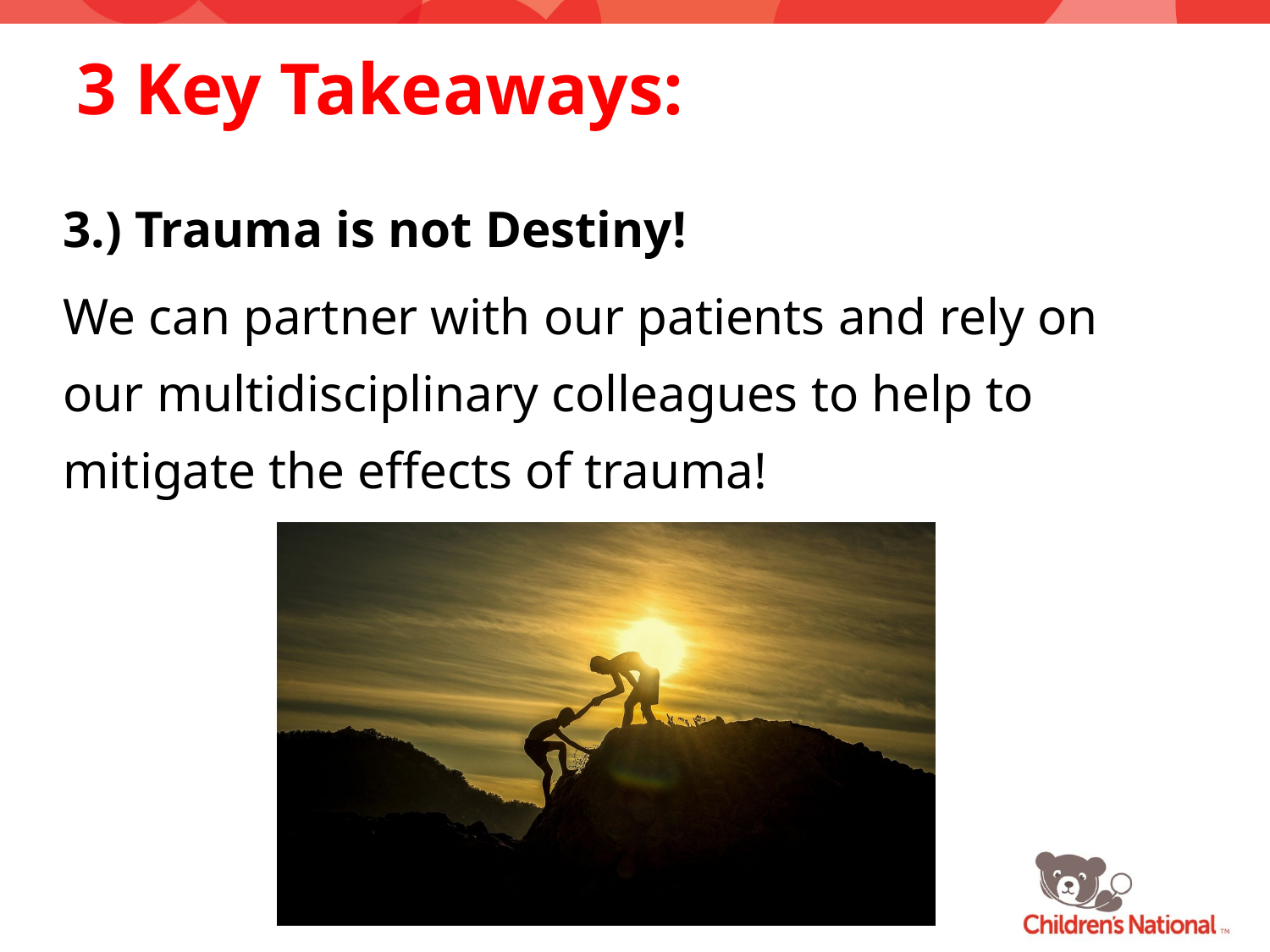

# 3 Key Takeaways:
3.) Trauma is not Destiny!
We can partner with our patients and rely on our multidisciplinary colleagues to help to mitigate the effects of trauma!

## Slide 79
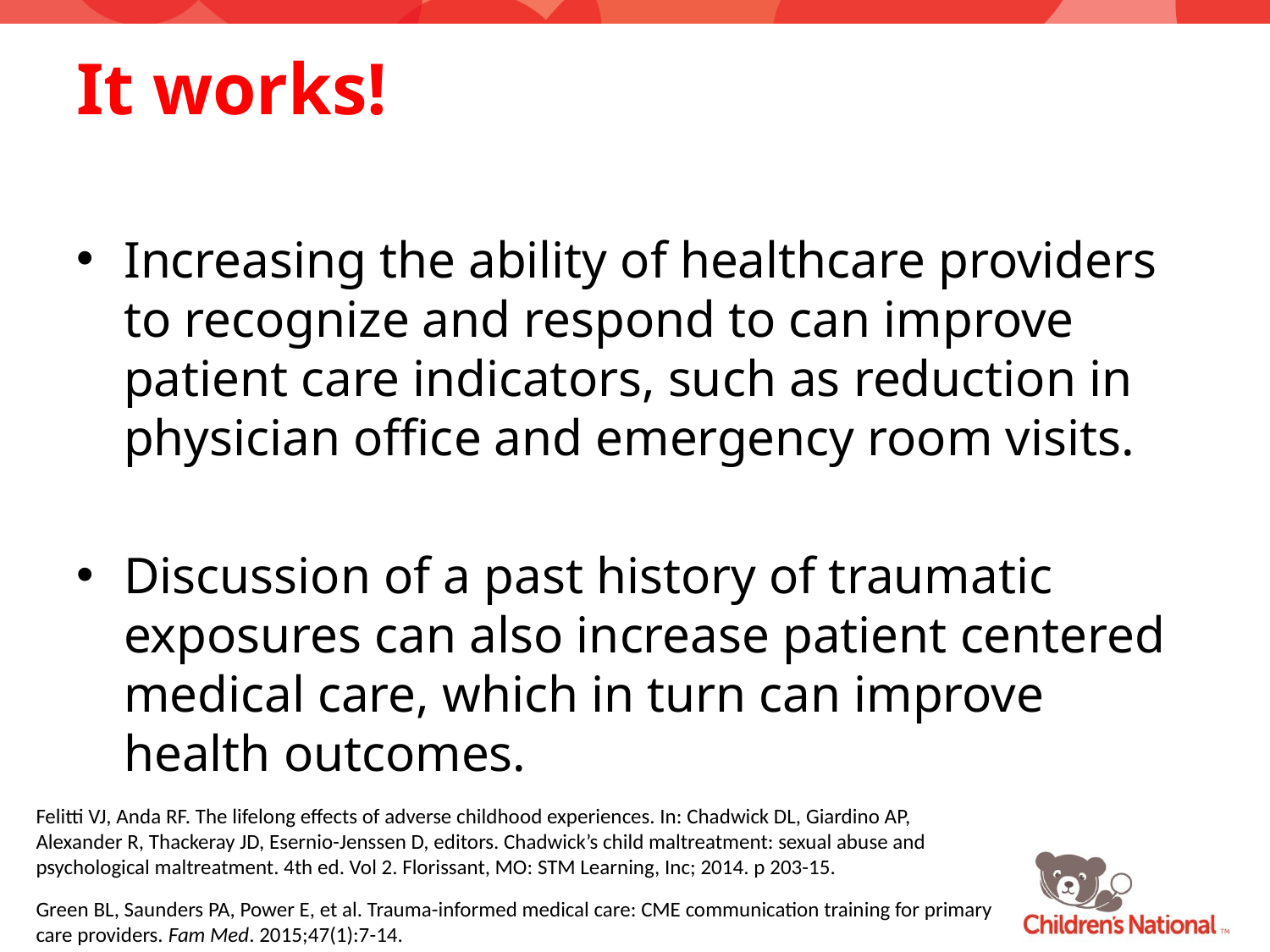

# It works!
Increasing the ability of healthcare providers to recognize and respond to can improve patient care indicators, such as reduction in physician office and emergency room visits.
Discussion of a past history of traumatic exposures can also increase patient centered medical care, which in turn can improve health outcomes.
Felitti VJ, Anda RF. The lifelong effects of adverse childhood experiences. In: Chadwick DL, Giardino AP, Alexander R, Thackeray JD, Esernio-Jenssen D, editors. Chadwick’s child maltreatment: sexual abuse and psychological maltreatment. 4th ed. Vol 2. Florissant, MO: STM Learning, Inc; 2014. p 203-15.
Green BL, Saunders PA, Power E, et al. Trauma-informed medical care: CME communication training for primary care providers. Fam Med. 2015;47(1):7-14.

## Slide 80
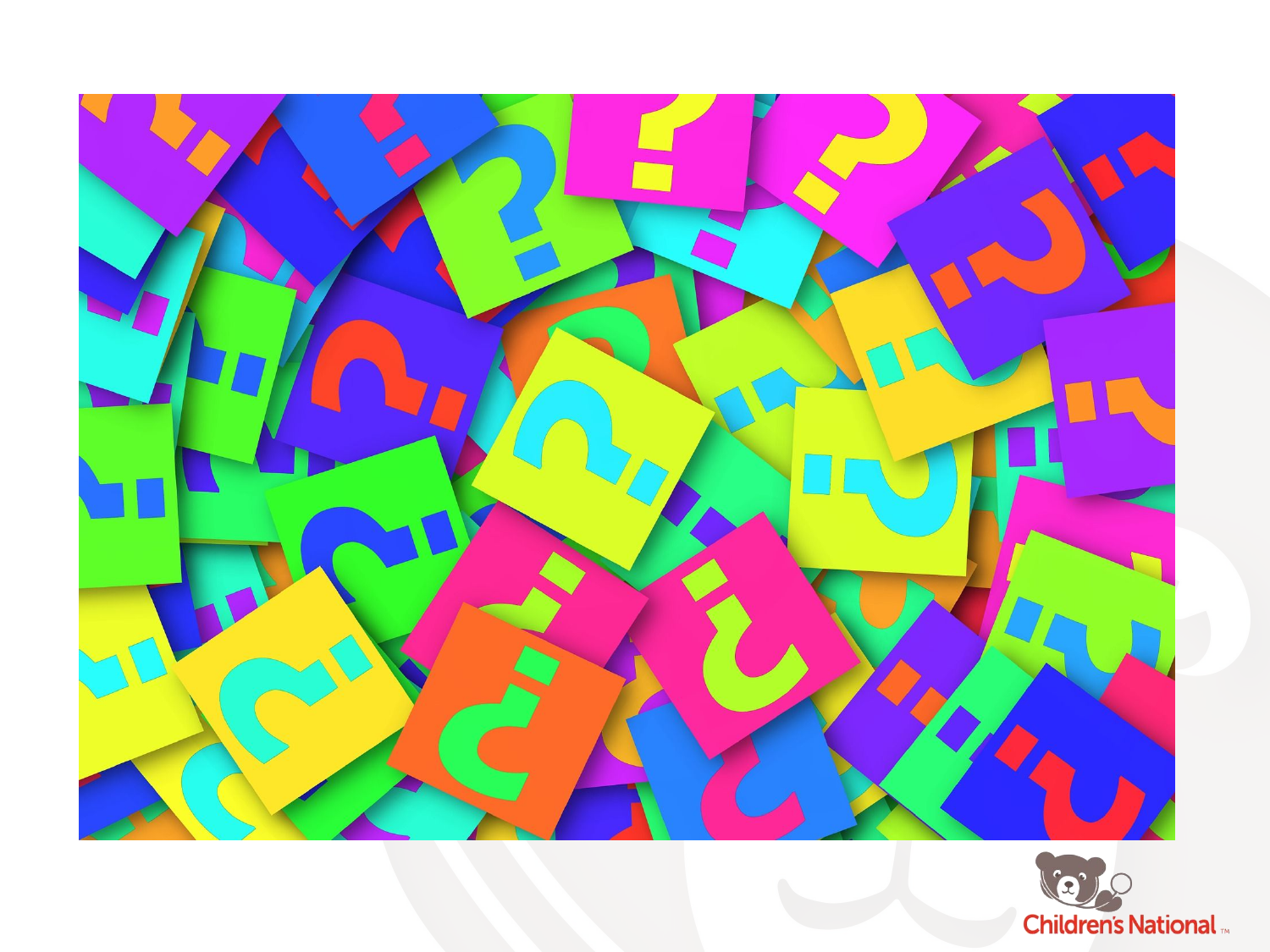

Supplement: Supplementary file 1 — TIC-S PowerPoint.pptxStress Health Self-Care Tool.pdfFacilitator Guide.docxEvaluation.docxFacilitator Prep Slides.pptx [file mep_2374-8265.11061-s001.zip › A. TIC-S PowerPoint.pptx]
