# Supplementary material for: Teaching Trauma-Informed Care: A Symposium for Medical Students
Source: MedEdPORTAL. 2020 Dec 30;16:11061. doi: 10.15766/mep_2374-8265.11061 (PMC7780743; doi:10.15766/mep_2374-8265.11061)
Supplement: Supplementary file 1 — TIC-S PowerPoint.pptxStress Health Self-Care Tool.pdfFacilitator Guide.docxEvaluation.docxFacilitator Prep Slides.pptx [file mep_2374-8265.11061-s001.zip › B. Stress Health Self-Care Tool.pdf]

Set Helpful Goals to Stress Health in Your Family

When a child experiences a lot of Adverse Childhood Experiences (ACEs), their body may make more stress hormones than is healthy. This can lead to problems with a child's physical and mental health such as asthma, being overweight, or behavior problems. Safe and nurturing relationships can protect children's brains and bodies from the harmful effects of stress and adversity. You can help your child be healthier by managing your own stress and helping your child reduce their stress. Healthy nutrition, daily exercise, healthy sleep, practicing mindfulness and getting mental health support can help to reduce stress hormones and prevent health problems. Here are some goals your family can set together to support your child's health (and your own!). [Check the goals that you are picking for yourself and your family.]

- ☐ **Healthy relationships.** We've set a goal of...
  - ☐ Using respectful communication even when we are upset or angry.
  - ☐ Spending more quality together as a family such as:
    - ☐ having regular family meals together.
    - ☐ having regular "no electronics" time for us to talk/play together.
  - ☐ Making time to see friends to create a healthy support system for myself and our family.
  - ☐ Connecting regularly with our community in settings like the YMCA, the library, our place of worship, or a local support group.
  - ☐ Create your own goal: ____

- ☐ **Exercise.** We've set a goal of...
  - ☐ Limiting screen time for fun (not homework) to less than two hours per day, and avoiding all screen time for babies under 2 years old.
  - ☐ Walking at least 20 minutes every day.
  - ☐ Finding a type of exercise that we enjoy and doing it together as a family.
  - ☐ Getting my child involved in a sport, dance class, or other form of routine exercise.
  - ☐ Create your own goal: ____

- ☐ **Nutrition.** We've set a goal of...
  - ☐ Drinking water instead of juice or soda.
  - ☐ Eating 5 vegetables and/or fruits every day.
  - ☐ Choosing whole grains like brown rice instead of white rice and whole wheat bread.
  - ☐ Adding fish oil to our diet.
  - ☐ Create your own goal: ____

- ☐ **Sleep.** We've set a goal of...
  - ☐ Turning off screens 30 minutes before bedtime.
  - ☐ Helping my child go to bed at the same time every night.
  - ☐ Making a routine of reading a book to my child before bed (or, if older, letting my child read to me).
  - ☐ Creating a calm place for sleep by dimming the lights and keeping the noise level down.
  - ☐ Using mindfulness or other stress reduction tools if worry is keeping my child up at night.
  - ☐ Create your own goal: ____

- ☐ **Mindfulness.** We've set a goal of...
  - ☐ Taking moments throughout the day to notice how we're feeling, both physically and emotionally.
  - ☐ Downloading a mindfulness app such as *Calm , Stop, Breathe and Think , or Headspace*.
  - ☐ Practicing mindful breathing during stressful situations.
  - ☐ Creating a routine of prayer, meditation, and/or a moment of gratitude daily.
  - ☐ Attending a yoga class regularly.
  - ☐ Create your own goal: ____

- ☐ **Mental health.** We've set a goal of...
  - ☐ Having a conversation as a family about your beliefs about mental health.
  - ☐ Learning more about mental health treatment options (e.g., counseling, therapy, psychiatric services).
  - ☐ Identifying a local mental health professional.
  - ☐ Scheduling an appointment with a mental health professional.
  - ☐ Create your own goal: ____

Remember, the most important ingredient for healthy kids is a healthy caregiver. Here are some goals that you can set for yourself to help your whole family be healthier..

- ☐ **Self Care.** I've set a goal of...
  - ☐ Making a plan for what to do when I'm feeling stressed out, angry or overwhelmed.
  - ☐ Planning with my partner, friends or family to have time for myself.
  - ☐ Calling for help if I am not safe at home.
  - ☐ Making a regular appointment with my medical provider for preventive care.
  - ☐ Taking the ACE Test for myself and talking to my medical provider about how to protect my health.
  - ☐ Create your own goal: ____

For more information:
www.centerforyouthwellness.org
www.stresshealth.org

CENTER for YOUTH WELLNESS

Bayview Child HEALTH CENTER
