# Supplementary material for: Teaching Trauma-Informed Care: A Symposium for Medical Students
Source: MedEdPORTAL. 2020 Dec 30;16:11061. doi: 10.15766/mep_2374-8265.11061 (PMC7780743; doi:10.15766/mep_2374-8265.11061)
Supplement: Supplementary file 1 — TIC-S PowerPoint.pptxStress Health Self-Care Tool.pdfFacilitator Guide.docxEvaluation.docxFacilitator Prep Slides.pptx [file mep_2374-8265.11061-s001.zip › C. Facilitator Guide.docx]

Appendix C: Facilitator Guide

**CASE DISCUSSION:**

**TRAUMA-INFORMED CARE SYMPOSIUM**

**Context of Session:**

Prior to the case discussion, students had interactive didactics reviewing the link between childhood adversity and health outcomes and introducing the concept of trauma-informed patient care. This session will provide an opportunity to apply the framework of a trauma-informed approach to care to a patient case.

Facilitators: There are three key takeaways from the symposium today:

1. Childhood adversity and traumatic exposure both in childhood and adulthood can affect the health and well-being of patients.
2. The central tenet of a trauma-informed approach to care is a shift from wondering what is “wrong” with a patient to instead exploring “what happened” to a patient.
3. Trauma is not destiny- through partnerships with our patients we can build resiliency.

We encourage facilitators to include their own patient stories through which employing a trauma focused approach to care was useful. During the preceding lecture, we helped student understand that trauma can be referred to as “stressors” for more patient friendly language. You can share how you assess for stressors that may be affecting patient health and/or how you to collaborate with multidisciplinary teams for patient care. You can also share how you explain the connection between trauma, stress, and health to patients.

**Total Time: 85 minutes**

**PART 1: 20 minutes**

**Small GROUP Part 1: 10:35-10:45 (10 minutes)**

**** Divide into groups of 8. Each group will answer all questions****

Joanna is a 40yoF who presents to your internal medicine practice in Washington, DC for a new patient visit for hypertension management. She was discharged from her prior medical home due to multiple missed appointments.

**Medical Diagnoses:**

- Obesity (diagnosed at age 16)
- Hypertension (diagnosed at age 28)
- Pre-Diabetes (diagnosed at age 35)
- Generalized Anxiety Disorder

**Medications:**

- Hydrochlorothiazide for management of hypertension
- Metformin for management of pre-diabetes

**Social History:**

- Lives alone
- No alcohol, drug, or tobacco use
- High school graduate, unemployed former retail employee

**Discussion Question 1:**

What factors may have contributed to the onset of multiple severe chronic illnesses at a young age?^[[1]](#footnote-1)^ *Answers can include adverse childhood experiences, social determinants of health, lack of access to health care, genetic predisposition, difficulty with medication compliance.*

**Discussion Question 2:**

Based on the lectures you heard this morning and the panel you attended last week on community violence, how do you think Joanna would answer the question “how was growing up for you?”1 What might have been some of the adverse childhood experiences that could have impacted Joanna? *Based on the preceding lecture we will expect that the students will answer that growing up was difficult for Joanna. Students may list the 10 original ACEs including physical, emotional, sexual abuse, emotional and physical neglect, incarcerated relative, parental separation, family mental illness, mother treated violently and family substance use. Encourage students to think beyond the original 10 ACEs for additional forms of traumatic exposures, such as discrimination, immigration status, neighborhood/community violence, gender discrimination, etc.*

**Discussion Question 3:**

If Joanna suffered from traumatic exposures, such as adverse childhood experiences, how could that be related to the development of her chronic illnesses? *Answers can include that adversity can lead to toxic stress which can lead to changes in stress hormone levels, neuro dysregulation, increased inflammation, and through these mechanisms organ specific disease.*

**Discussion Question 4:**

If you suspect or want to informally inquire about traumatic exposures: How would you start the conversation and what question(s) would you ask? *Examples from preceding lecture included:*

- *“A lot of research shows that stress can affect health. I wanted to ask, how do you think stress may be playing a role in your diagnosis of obesity?”*
- *“Many times, stressful life events can affect a person’s health and well-being. Are there any stressful life events that you would like to share with me today?”*

**LARGE GROUP PART 1: 10 minutes**

**Have each small group of 8 present their answers to one of the preceding questions to the larger group. One group will present answer to question 1 and question 4.**

****Facilitators to ask for any questions from the group before preceding to Part 2****

**Part 2: 10:55-11:20a**

**Ask student to read the information below.**

Over multiple visits you slowly learn more about Joanna:

Joanna grew up in a household headed by a single mother. She was the youngest of three children, all of whom were half siblings.

Joanna’s mother often worked multiple jobs to try to make ends meet. When she would come home at the end of the day she would be exhausted and would often use alcohol to calm her nerves to help to get Joanna and her siblings to bed. Her mother found this to be an easier way of controlling her history of depression than to be on psychotropic medications. It was also not uncommon for Joanna’s mother to use a belt to discipline the children before bedtime.

Though Joanna longed to see her father and have him in her life, she rarely saw him, as he was intermittently incarcerated.

Due to Joanna’s mother history of alcohol abuse, a pediatrician had called Child Protective Services during a routine well visit for Joanna. This led to her being placed in foster care. Joanna found her foster parent to be verbally abusive and therefore would abscond for weeks at time, where she lived with a man, “Mr. Jones,” who would buy her clothes and food in exchange for sex.

When Joanna turned 21 she was able to exit the foster system and live in public housing. She was attended community college and completed two years of courses, falling short of graduating. She has been working various retail jobs to make ends meet, but due to multiple sick days needed, is unable to keep a steady job. Joanna tells you that she has wanted to keep her appointments with her prior doctor, but it has been difficult for her to manage her long standing anxiety disorder, and to keep on top of all of her responsibilities. She is interested in mental health care for her anxiety but has had difficulty finding a provider that takes her insurance. She states she just can’t understand what she is doing wrong and why she has such “bad luck” with all of her bad health diagnoses.

**SMALL GROUP PART 2: 15 minutes**

****Have each group of 8 students answer all of the following questions.****

**Discussion Question 1:**

What are Joanna’s traumatic exposures? *Parental separation, mental illness in mother, father incarcerated, mother’s substance abuse, physical abuse, and verbal/emotional abuse from foster parents. Total Score: 6. Can highlight that research has shown that an ACE score of 6 or greater leads to 20 year decrease in life expectancy.*

**Discussion Question 2:**

How would you respond to Joanna’s statement about her “bad luck” and explain to Joanna the connection between her past traumatic exposures and her current health?

*Many times, stressful life events can affect a person’s health and well-being. Do you think that the stressful events that you shared with us could be affecting your health? If so, how?*

*Emphasize that the medical diagnoses here are not simply rooted in unhealthy habits. Underscore the biologic effects of stress on the body and how this overall has an effect on the body’s physiology and increases risk for medical co-morbidities.*

**Discussion Question 3:**

How would Joanna’s response and disclosure of a history of trauma alter/change/improve your approach to her care?

- How would you engage Joanna in care planning for her medical diagnoses?
- How could you use the “Stress Health Self Care Tool” during the patient visit, or for follow-up?

*Students can look through “Stress Health Self Care Tool” and should emphasize that they will work collaboratively with Joanna to choose goals in her care and then make a follow-up plan based on these goals. If Joanna is amenable, the plan should also include referral to mental health. Regarding engaging patients, can emphasize that building supportive relationships, resiliency, and healthy habits can help lessen the effect of traumatic exposures on health and well-being.*

**LARGE GROUP PART 2: 10 minutes**

**Have each small group of 8 present their answer to one of the preceding questions to the larger group.**

****Facilitators to ask for any questions from the group before preceding to Part 3****

**Part 3: LARGE GROUP DISCUSSION: 20 minutes**

FACILITATORS will moderate:

1. What are the barriers to employing a trauma-informed approach to care?

*Time, lack of training in trauma assessment, lack of familiarity with how to respond to positive screening questions, lack of referral resources, lack of longitudinal relationship with patients, fear of opening up a “Pandora’s box” with patients (may not be able to manage patient’s or my own emotional reaction).*

1. What would happen to this patient if you do not employ a trauma-informed approach to care?

*Most dramatically, ACE study and subsequent research has shown that those with 6 or greater ACEs have 20 year decreased life expectancy. We can mitigate this statistic by understanding how trauma can affect our patients, collaborating with them to create a plan of care that includes strengths focused approach to care to build resiliency, connect to mental health resources, and encourages healthy habits.*

1. What if anything is unclear about what we have discussed today?

Points to emphasize with students:

- *While this case highlighted the childhood story of a woman who was raised by single mother who struggled to make ends meet, trauma is ubiquitous and is seen across populations, genders, race, ethnicity, socio-economic status, etc.*
- *While this case highlighted the multiple ACEs that Joanna experienced- one ACE can have as significant as an effect on health as having many ACEs. It is not necessarily about the number.*
- *The students saw a lifecourse slide during the preceding didactics. While the ACEs study highlighted the effect of* ***childhood*** *trauma on health, traumas at all times in life can have effects on health.*
- *Students will not always know the traumas their patients have experienced. Disclosure is NOT the goal. Use universal precautions: trauma is ubiquitous. Having a trauma-informed approach and recognizing that the ideals of a trauma-informed approach and building resilience through collaboration and supportive relationships can help to mediate the link between trauma and health.*

**Part 4: 20 minutes**

Reflection/Evaluation

1. Rush, P and Stillerman A. Core Concepts, THEN. Available at: <https://www.thencenter.org/secondary-trauma/>. Accessed April 30, 2020. [↑](#footnote-ref-1)
