# Supplementary material for: Teaching Trauma-Informed Care: A Symposium for Medical Students
Source: MedEdPORTAL. 2020 Dec 30;16:11061. doi: 10.15766/mep_2374-8265.11061 (PMC7780743; doi:10.15766/mep_2374-8265.11061)
Supplement: Supplementary file 1 — TIC-S PowerPoint.pptxStress Health Self-Care Tool.pdfFacilitator Guide.docxEvaluation.docxFacilitator Prep Slides.pptx [file mep_2374-8265.11061-s001.zip › D. Evaluation.docx]

**George Washington University School of Medicine and Health Sciences**

**Patient Populations and Systems 3**

**Trauma Informed Care Symposium Evaluation**

**Sept 19, 2019**

**1 = Strongly Disagree 2 = Disagree 3 = Neutral 4 = Agree 5 = Strongly Agree**

| After today’s symposium, I have increased knowledge regarding how adverse childhood experiences affect health outcomes. | 1 | 2 | 3 | 4 | 5 | N/A |
| --- | --- | --- | --- | --- | --- | --- |
| After today’s symposium, I have a better understanding of trauma informed care. | 1 | 2 | 3 | 4 | 5 | N/A |
| After today’s symposium, I have a better understanding of how to incorporate trauma informed practices during patient interactions. | 1 | 2 | 3 | 4 | 5 | N/A |
| I plan to use the things I learned today in my clinical rotations. | 1 | 2 | 3 | 4 | 5 | N/A |

**Which parts of the symposium were most helpful to increase your knowledge on adverse childhood experiences and trauma informed care?**

**How did the symposium build on other concepts that have been presented in PPS?**

**What surprised you most about what you learned today?**

**What changes to the symposium would you recommend to benefit student learning?**

**How interested would you be in learning about trauma informed physical examination technique?**

**1**= No interest **2** = Somewhat Interested **3**= Interested **4**= Very interested

**Overall rating of the Trauma Informed Care Symposium? (circle response):**

1 (Poor) 2 (Fair) 3 (Good) 4 (Excellent) 5 (Outstanding)

**To help gain information on future topics, please answer the two questions below and then continue to the reflection.**

**How would you rate your interest in learning more about applying trauma informed care principles to care for trafficked children and youth?**

**1**= No interest **2** = Somewhat Interested **3**= Interested **4**= Very interested

**How would you rate your knowledge on issues related to CSEC/DMST/trafficked children and youth?**

**1**= little to no knowledge **2**= Somewhat Knowledgeable **3**= Knowledgeable **4**= Very knowledgeable

**Reflection Questions:**

**(1) Did the content presented today remind you of a specific patient case? If so, how might you now approach that situation differently?**

**(2) How will the information you learned in the symposium today affect your patient care rotations? Please be specific.**
