# Supplementary material for: Teaching Trauma-Informed Care: A Symposium for Medical Students
Source: MedEdPORTAL. 2020 Dec 30;16:11061. doi: 10.15766/mep_2374-8265.11061 (PMC7780743; doi:10.15766/mep_2374-8265.11061)
Supplement: Supplementary file 1 — TIC-S PowerPoint.pptxStress Health Self-Care Tool.pdfFacilitator Guide.docxEvaluation.docxFacilitator Prep Slides.pptx [file mep_2374-8265.11061-s001.zip › E. Facilitator Prep Slides.pptx]

## Slide 1
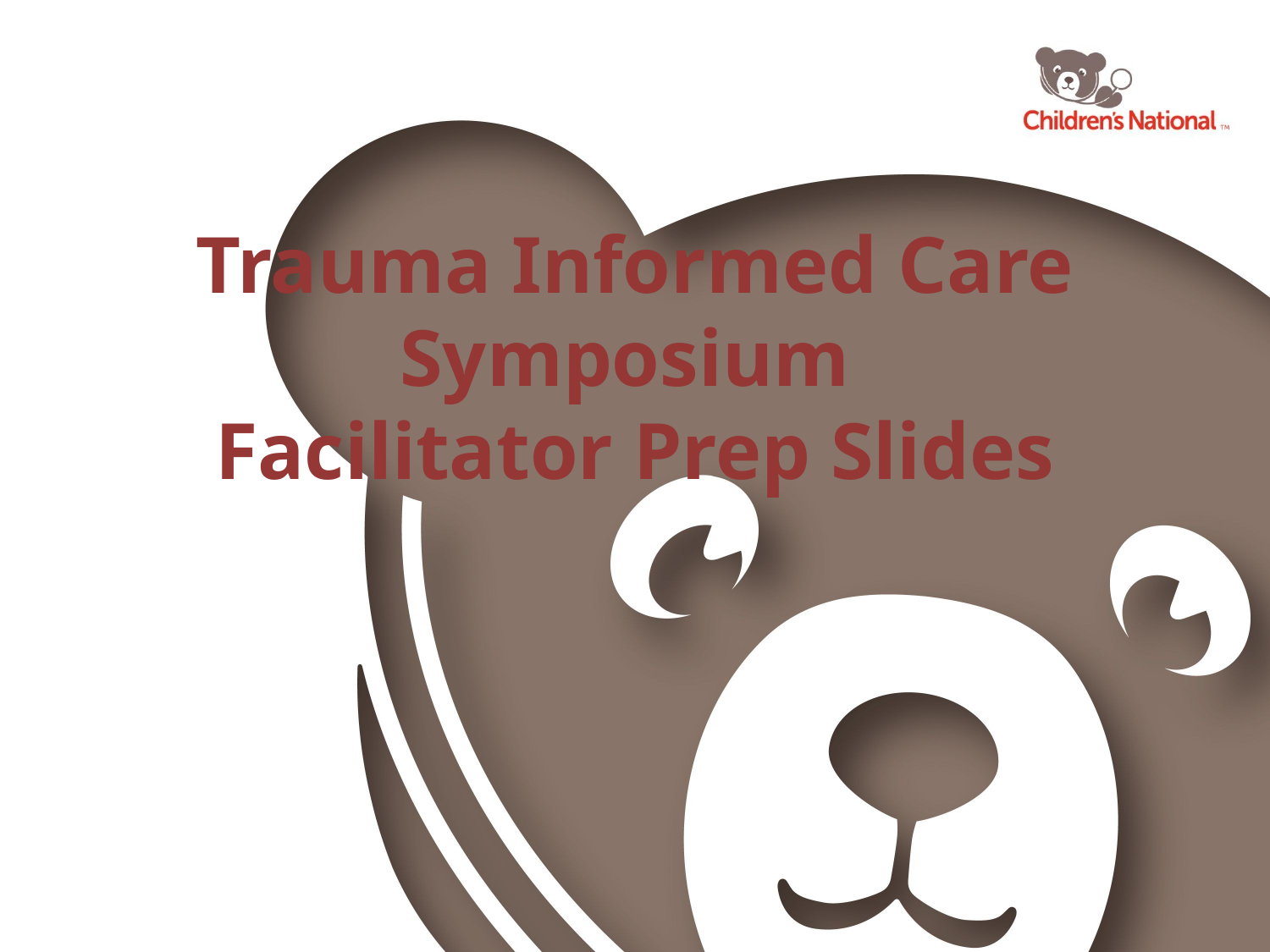

# Trauma Informed Care Symposium Facilitator Prep Slides

## Slide 2
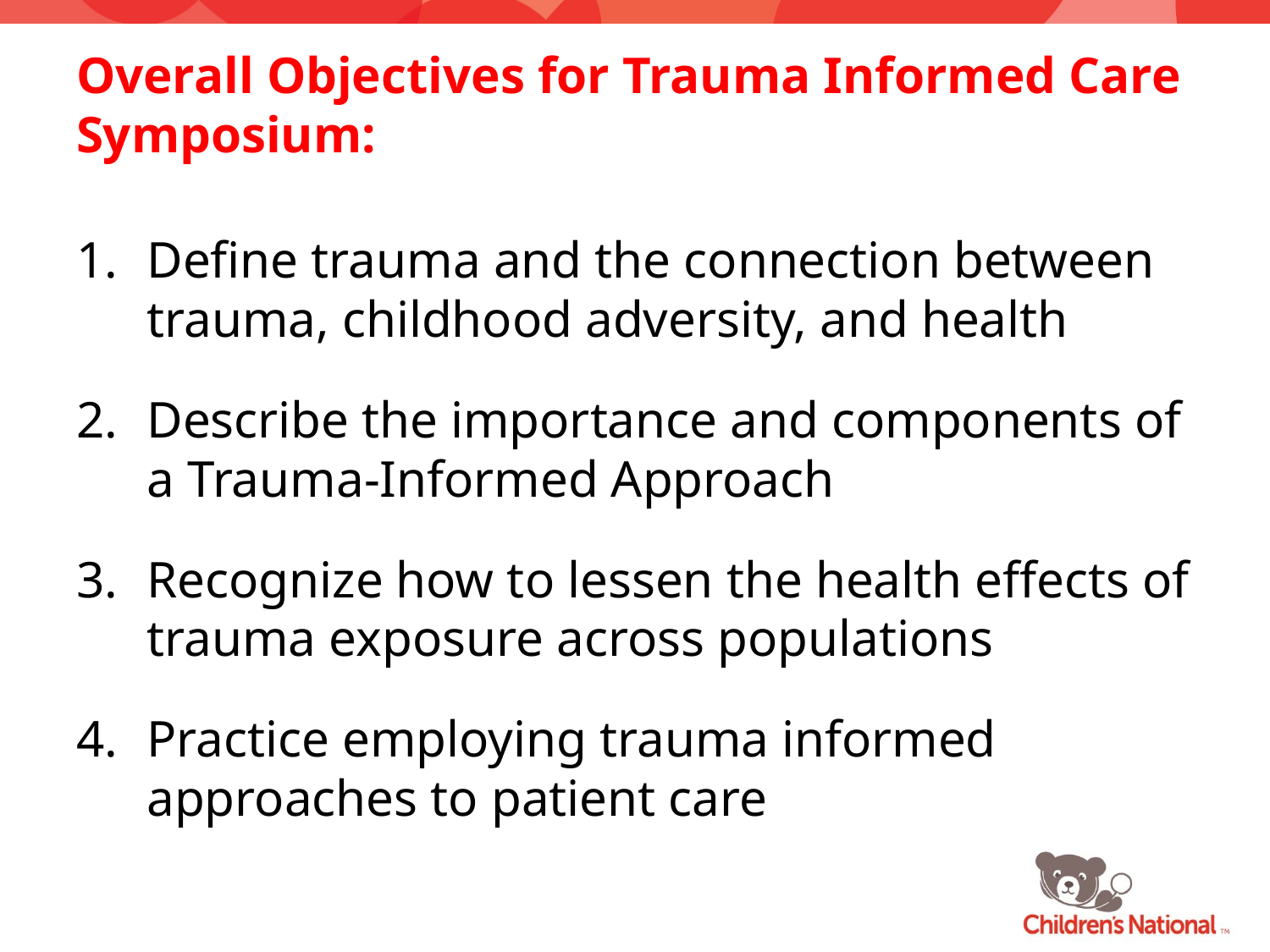

# Overall Objectives for Trauma Informed Care Symposium:
Define trauma and the connection between trauma, childhood adversity, and health
Describe the importance and components of a Trauma-Informed Approach
Recognize how to lessen the health effects of trauma exposure across populations
Practice employing trauma informed approaches to patient care

## Slide 3
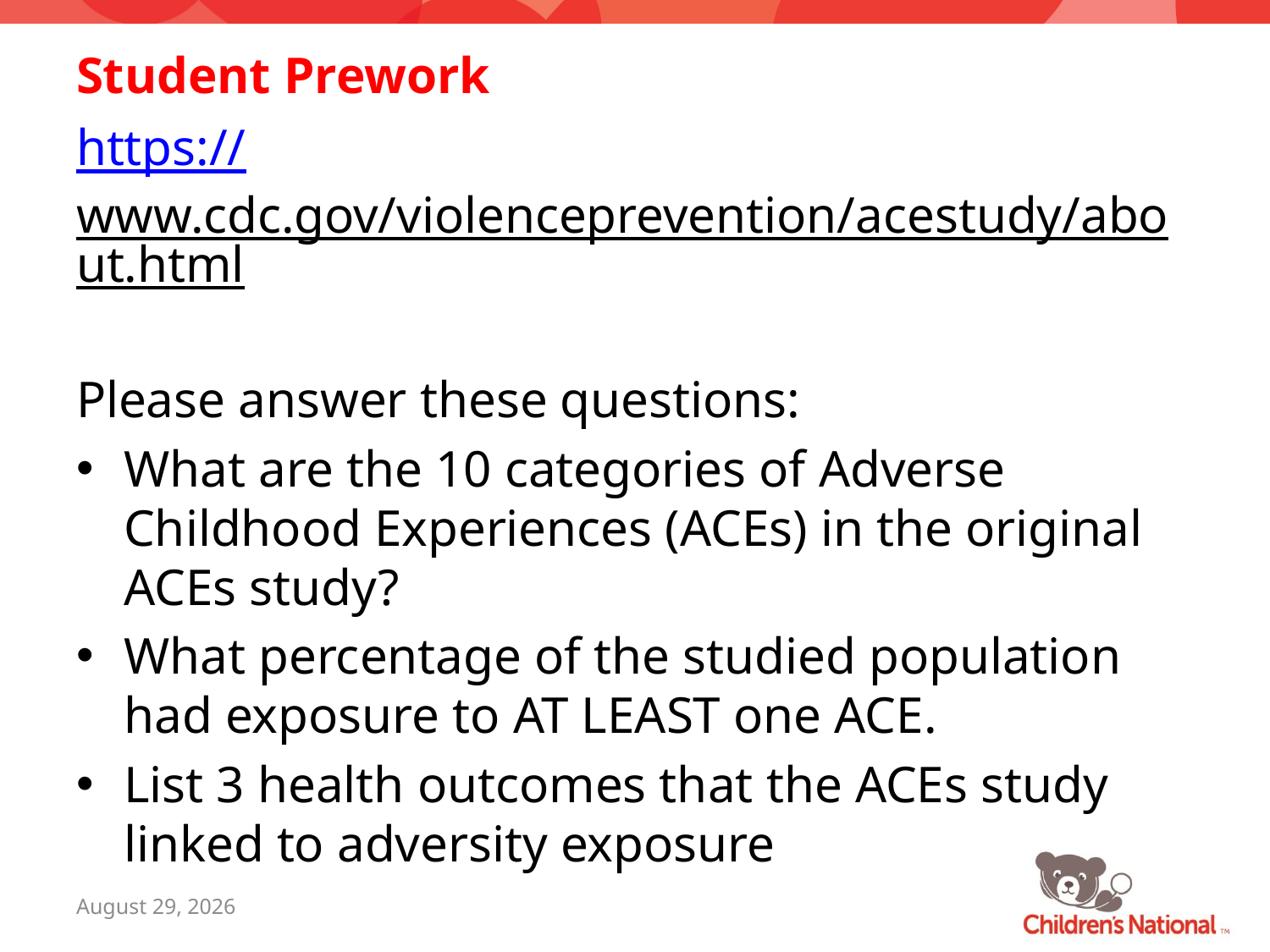

# Student Prework
https://www.cdc.gov/violenceprevention/acestudy/about.html
Please answer these questions:
What are the 10 categories of Adverse Childhood Experiences (ACEs) in the original ACEs study?
What percentage of the studied population had exposure to AT LEAST one ACE.
List 3 health outcomes that the ACEs study linked to adversity exposure
June 4, 2020

## Slide 4
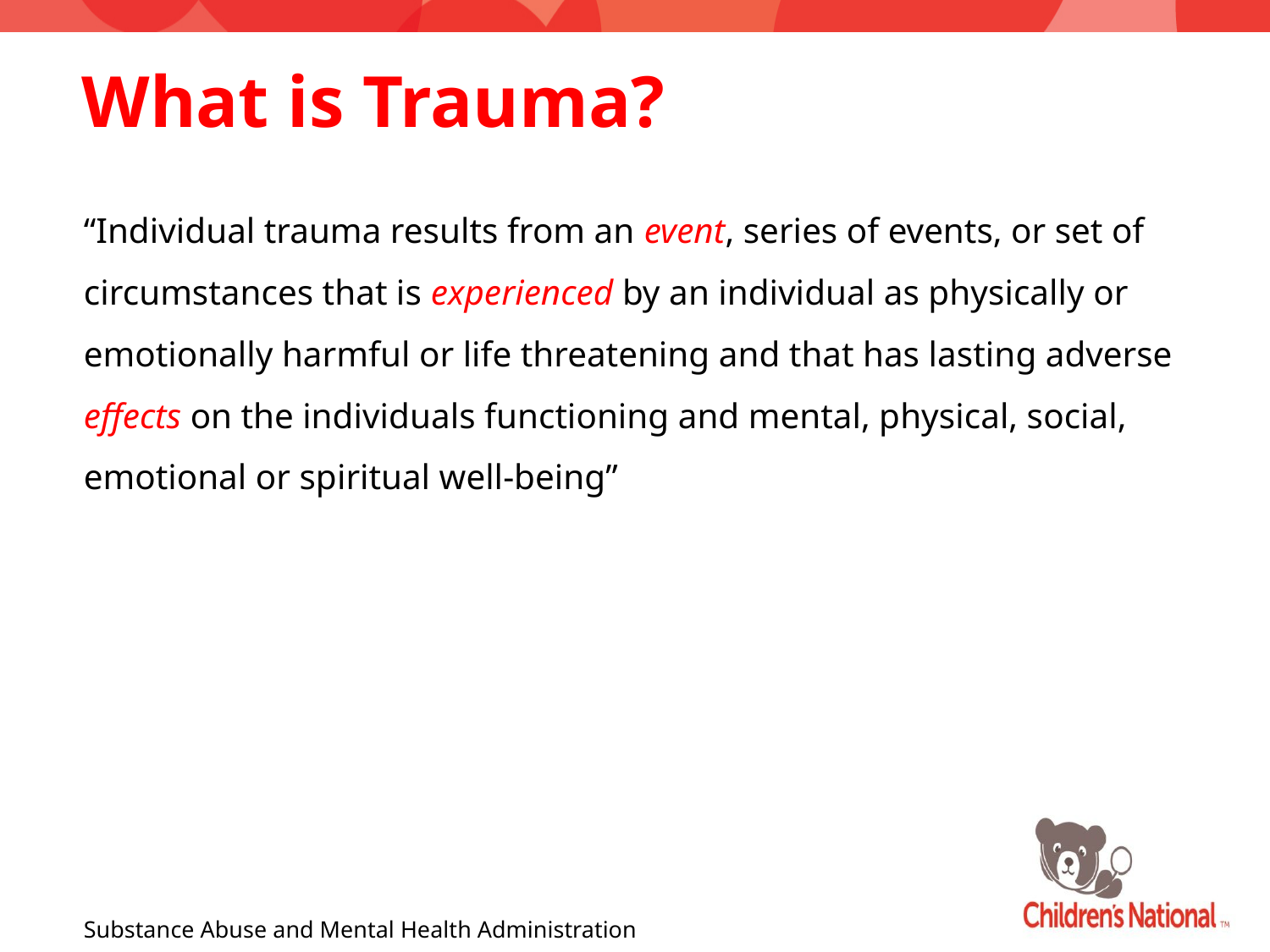

# What is Trauma?
“Individual trauma results from an event, series of events, or set of circumstances that is experienced by an individual as physically or emotionally harmful or life threatening and that has lasting adverse effects on the individuals functioning and mental, physical, social, emotional or spiritual well-being”
Substance Abuse and Mental Health Administration

## Slide 5
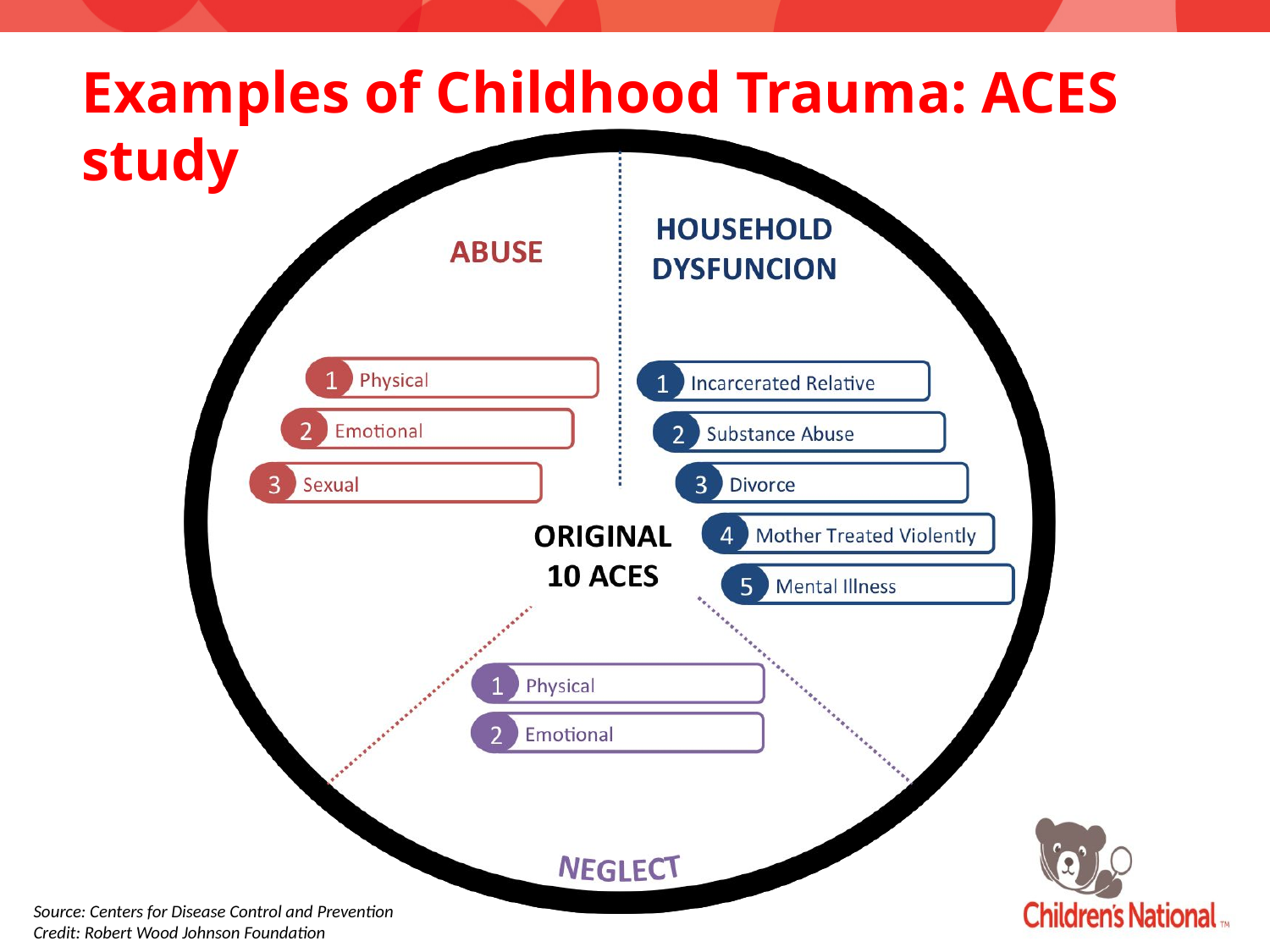

# Examples of Childhood Trauma: ACES study
Source: Centers for Disease Control and Prevention
Credit: Robert Wood Johnson Foundation

## Slide 6
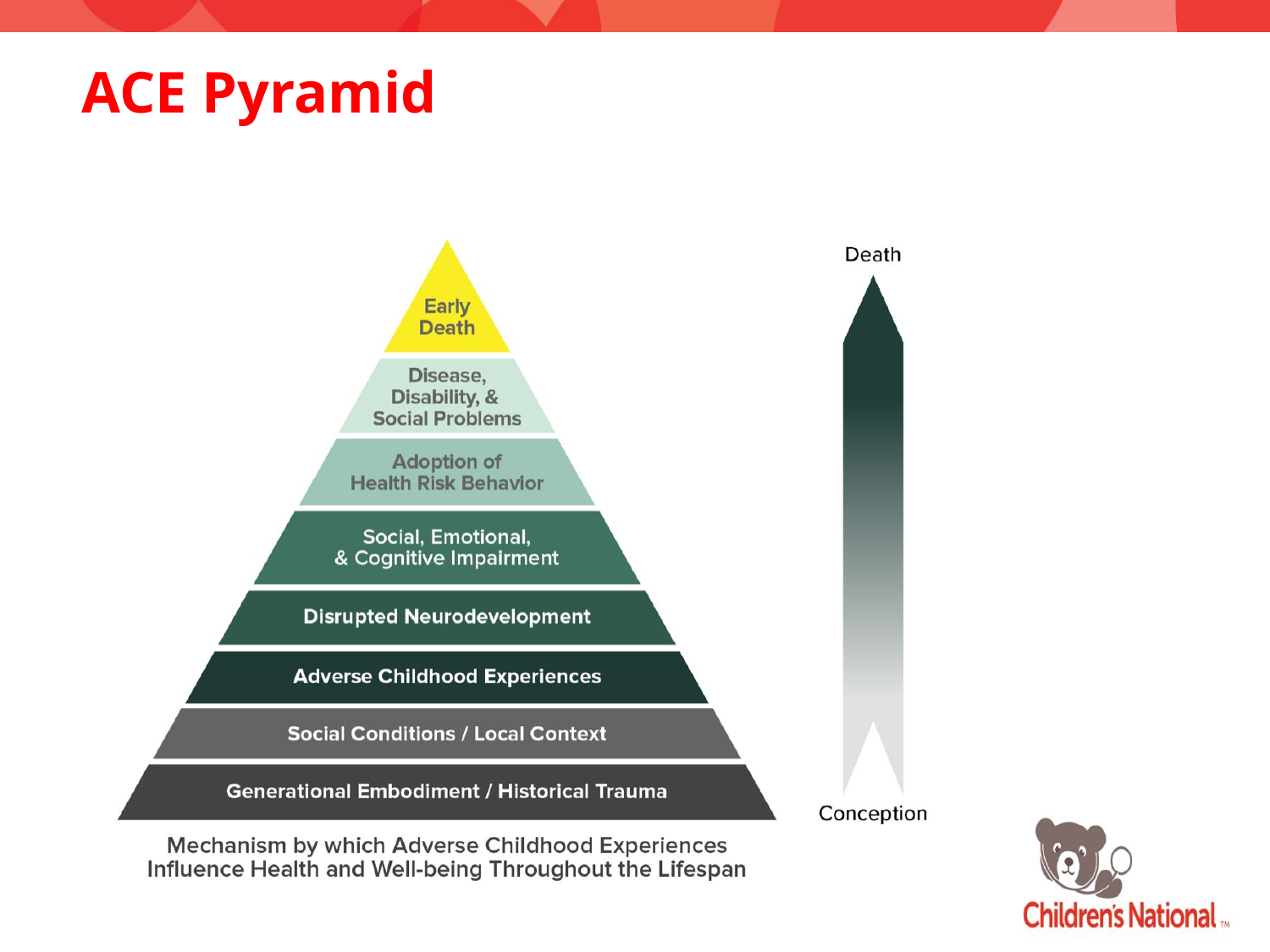

# ACE Pyramid

## Slide 7
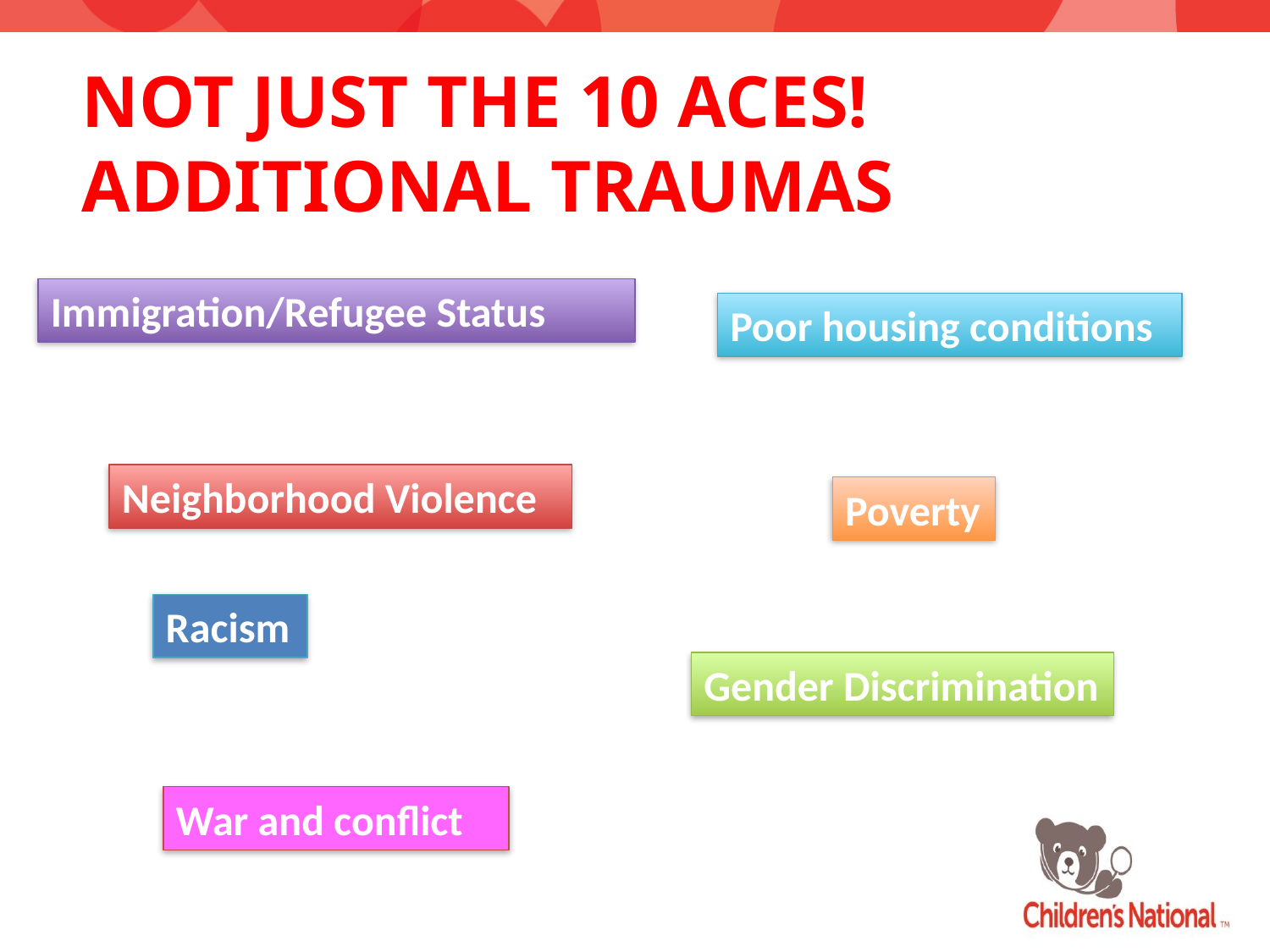

# Not just the 10 ACEs! Additional traumas
Immigration/Refugee Status
Poor housing conditions
Neighborhood Violence
Poverty
Racism
Gender Discrimination
War and conflict

## Slide 8
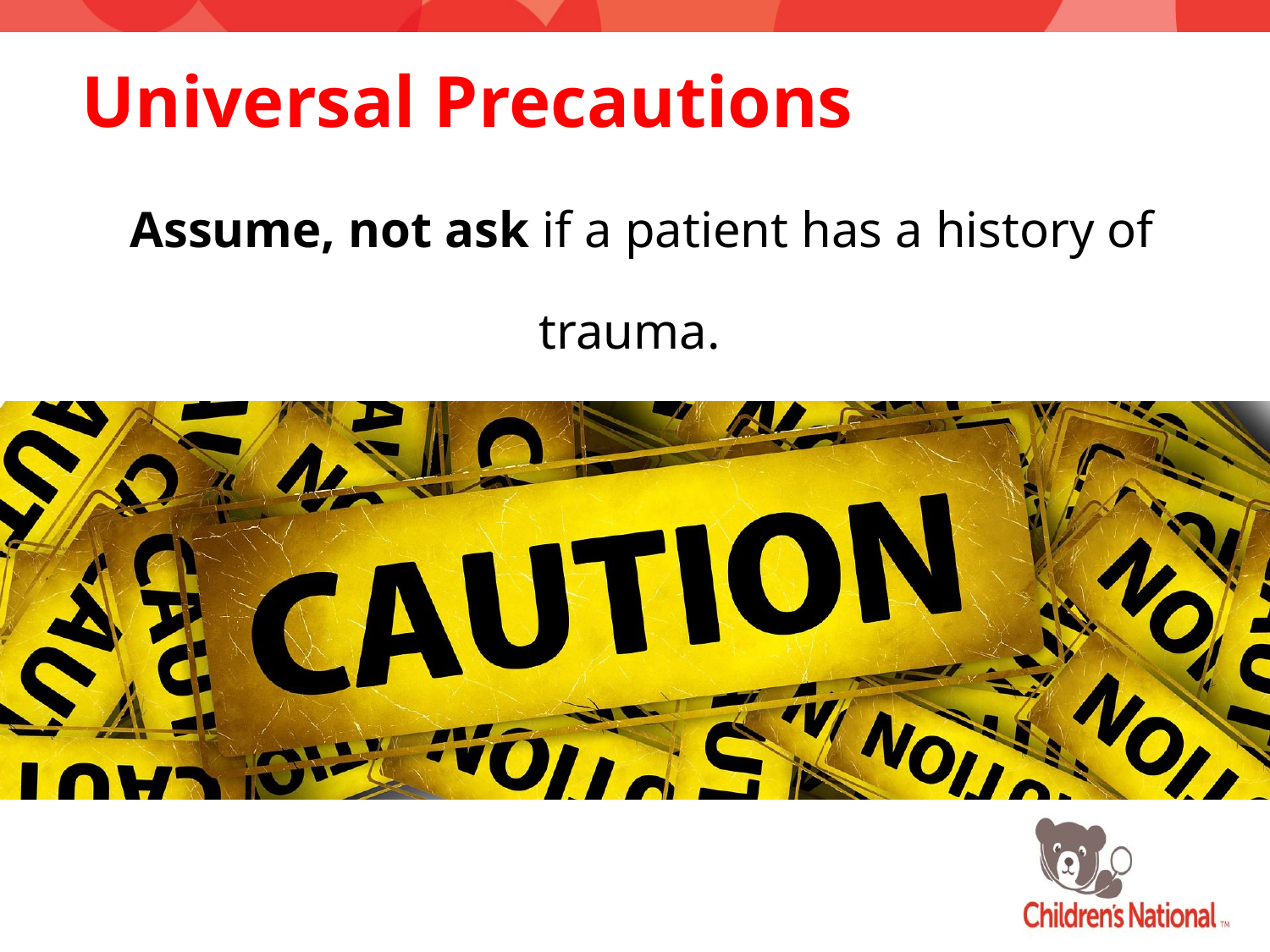

# Universal Precautions
Assume, not ask if a patient has a history of trauma.

## Slide 9
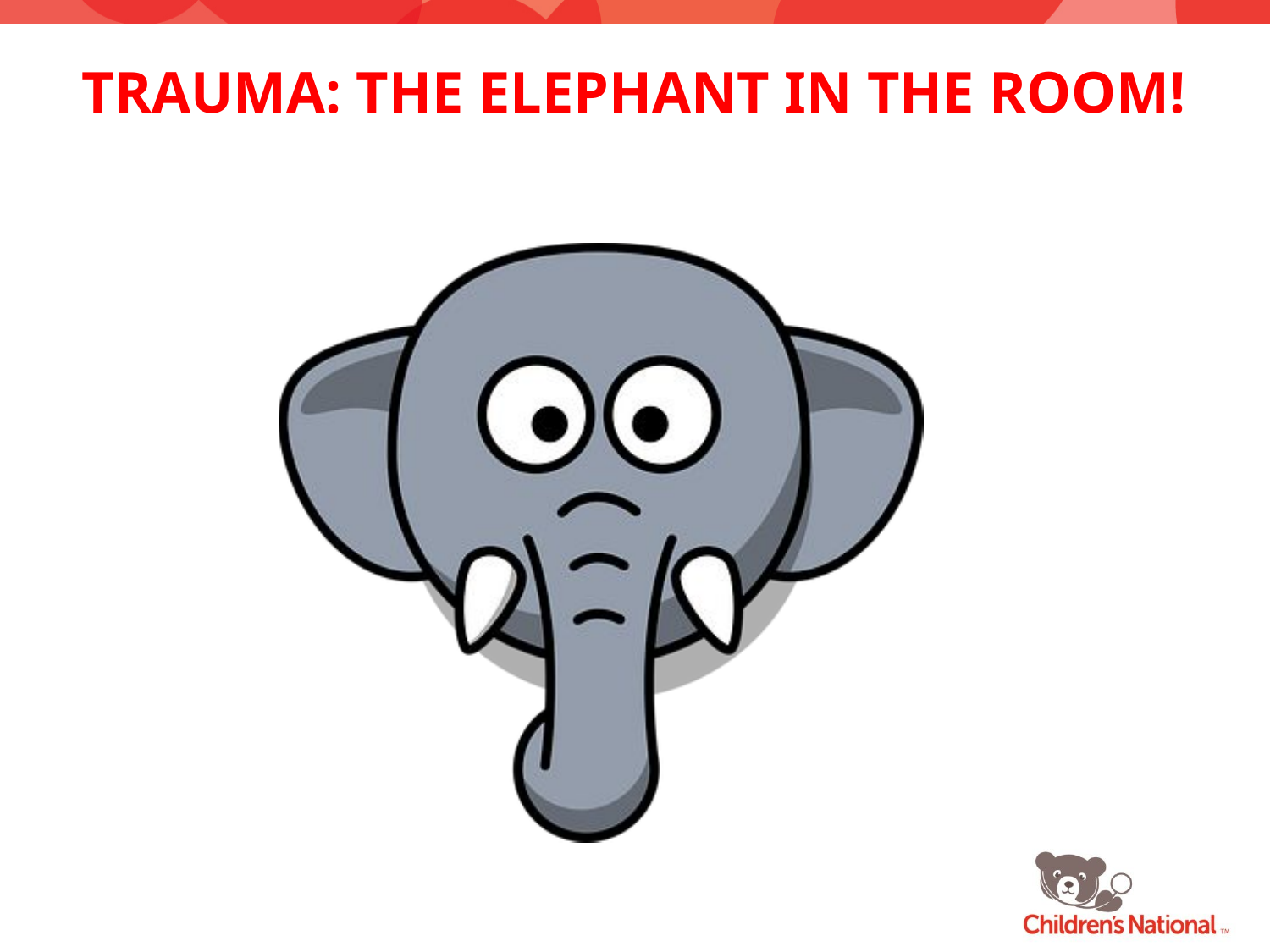

# TRAUMA: THE ELEPHANT IN THE ROOM!

## Slide 10
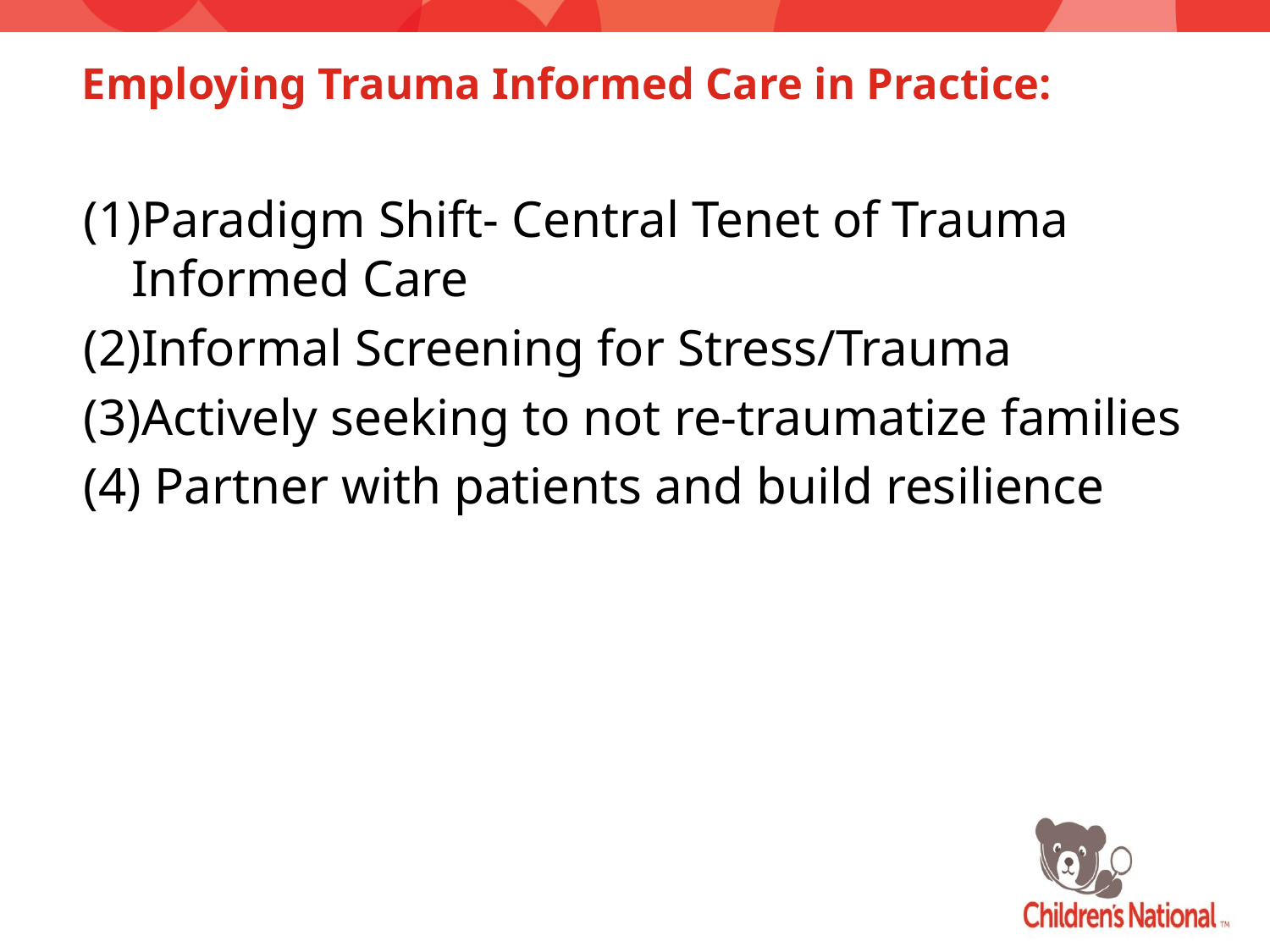

# Employing Trauma Informed Care in Practice:
Paradigm Shift- Central Tenet of Trauma Informed Care
Informal Screening for Stress/Trauma
Actively seeking to not re-traumatize families
 Partner with patients and build resilience

## Slide 11
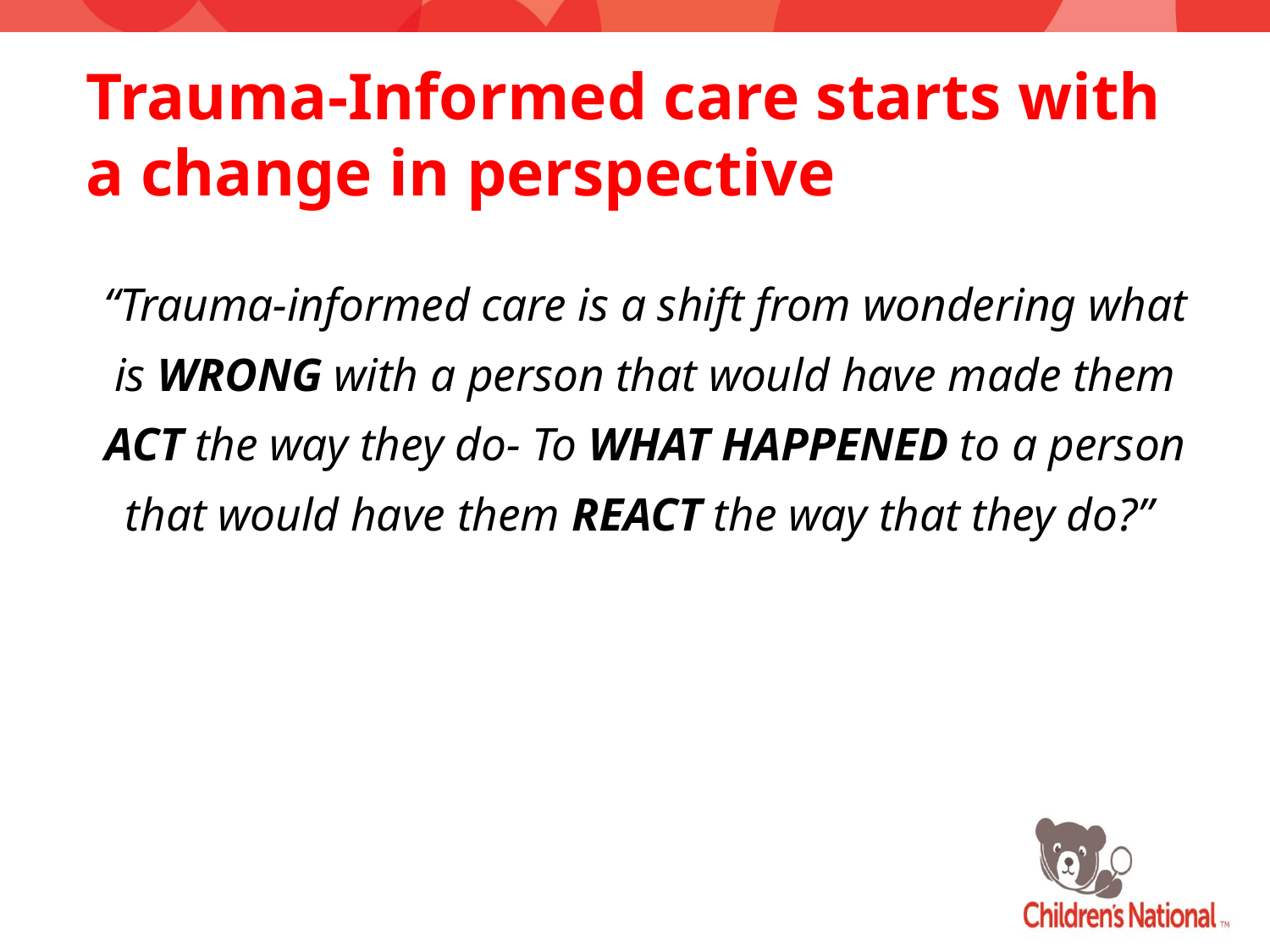

# Trauma-Informed care starts with a change in perspective
“Trauma-informed care is a shift from wondering what is WRONG with a person that would have made them ACT the way they do- To WHAT HAPPENED to a person that would have them REACT the way that they do?”

## Slide 12
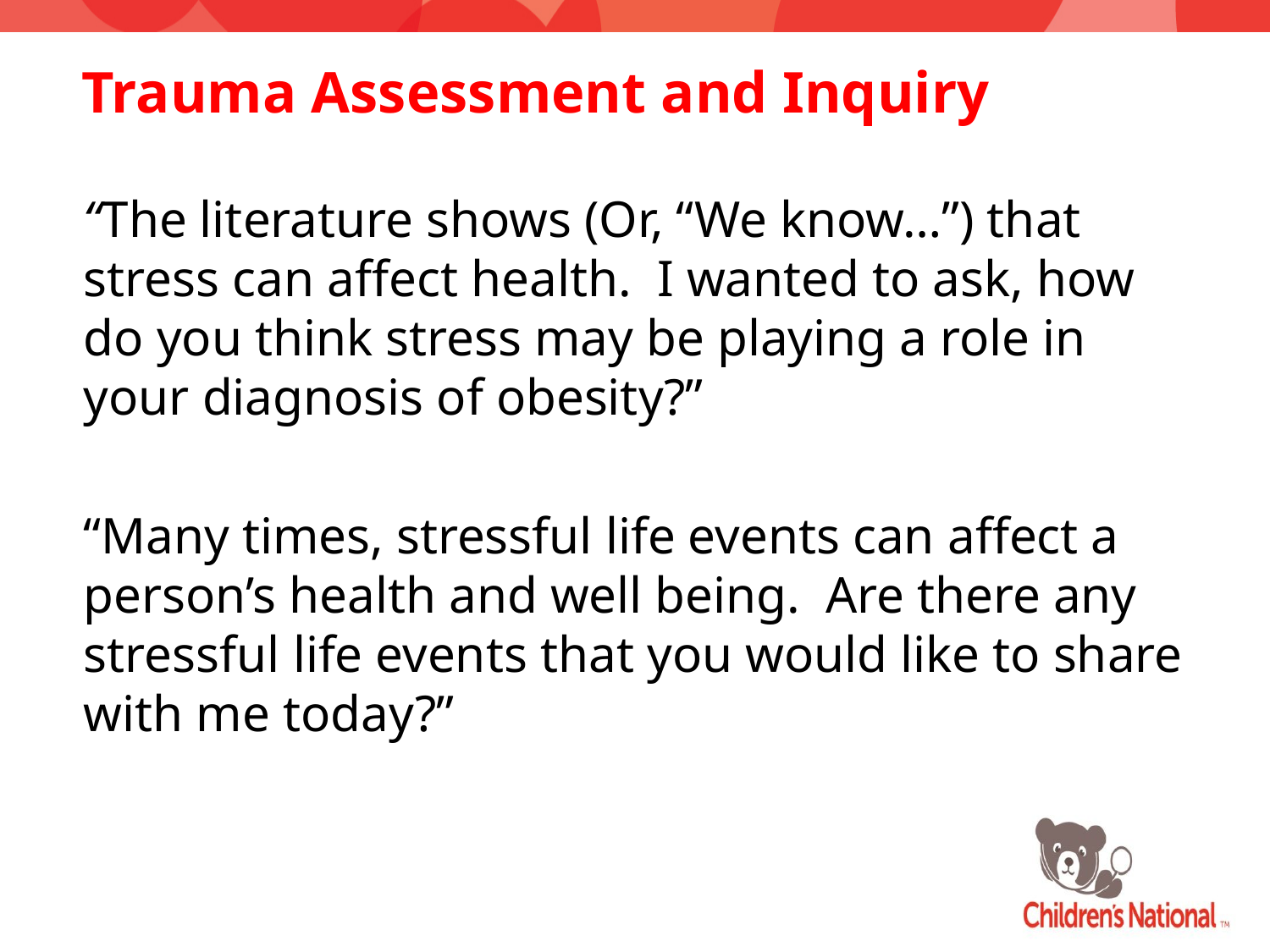

# Trauma Assessment and Inquiry
“The literature shows (Or, “We know…”) that stress can affect health. I wanted to ask, how do you think stress may be playing a role in your diagnosis of obesity?”
“Many times, stressful life events can affect a person’s health and well being. Are there any stressful life events that you would like to share with me today?”

## Slide 13
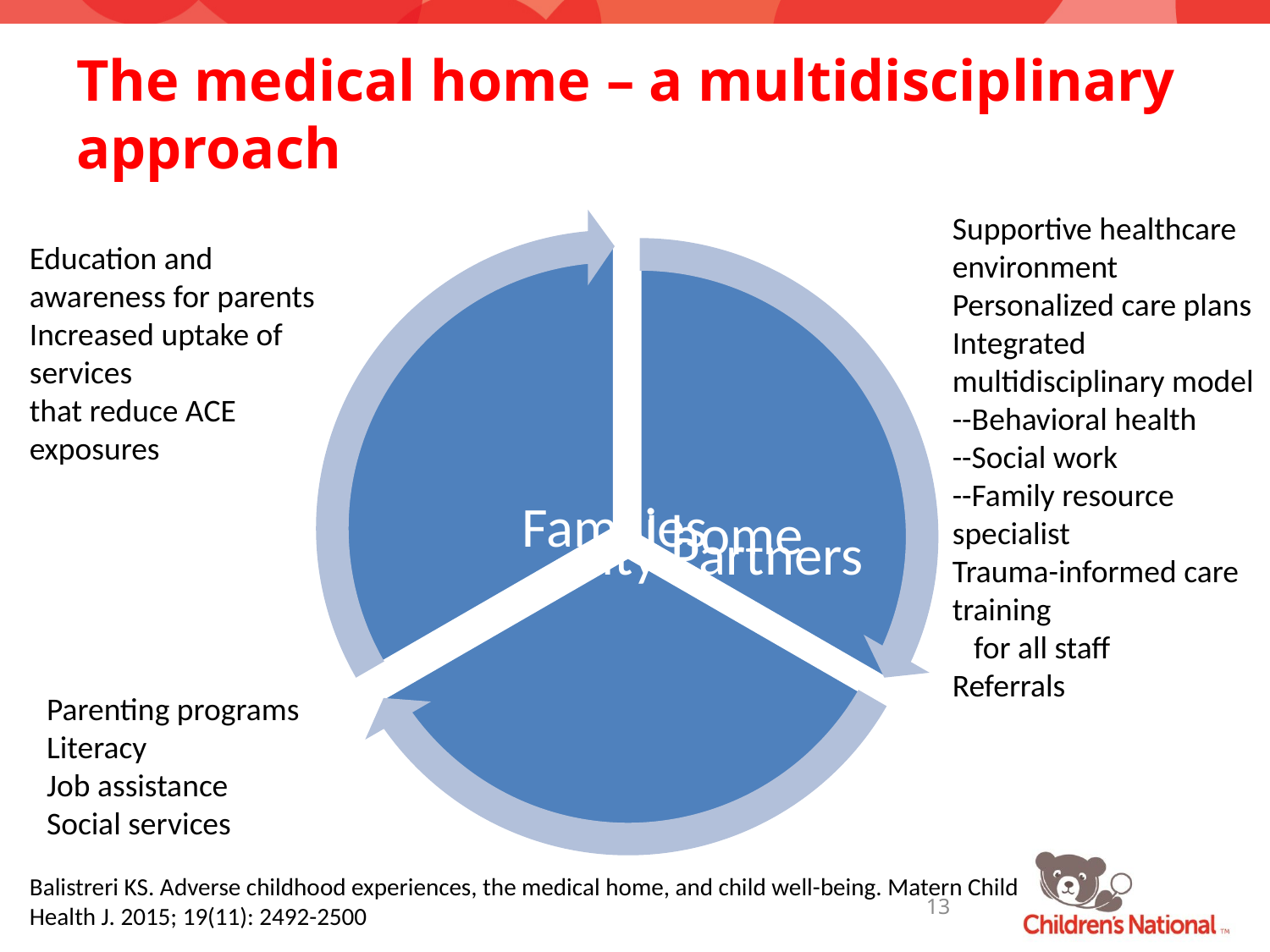

# The medical home – a multidisciplinary approach
Supportive healthcare environment
Personalized care plans
Integrated multidisciplinary model
--Behavioral health
--Social work
--Family resource specialist
Trauma-informed care training
 for all staff
Referrals
Education and awareness for parents
Increased uptake of services
that reduce ACE exposures
Parenting programs
Literacy
Job assistance
Social services
Balistreri KS. Adverse childhood experiences, the medical home, and child well-being. Matern Child Health J. 2015; 19(11): 2492-2500
13

## Slide 14
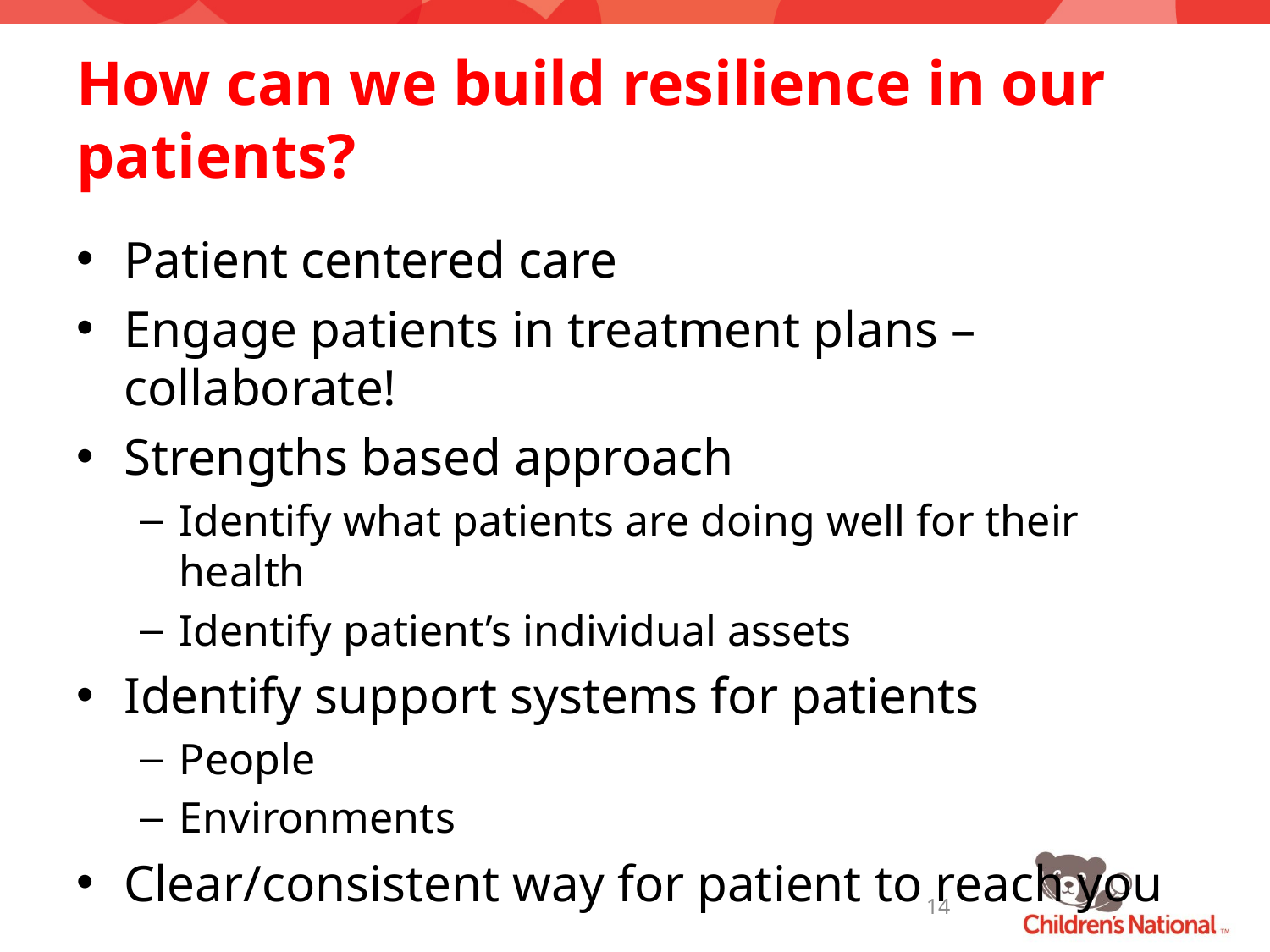

# How can we build resilience in our patients?
Patient centered care
Engage patients in treatment plans – collaborate!
Strengths based approach
Identify what patients are doing well for their health
Identify patient’s individual assets
Identify support systems for patients
People
Environments
Clear/consistent way for patient to reach you
14

## Slide 15
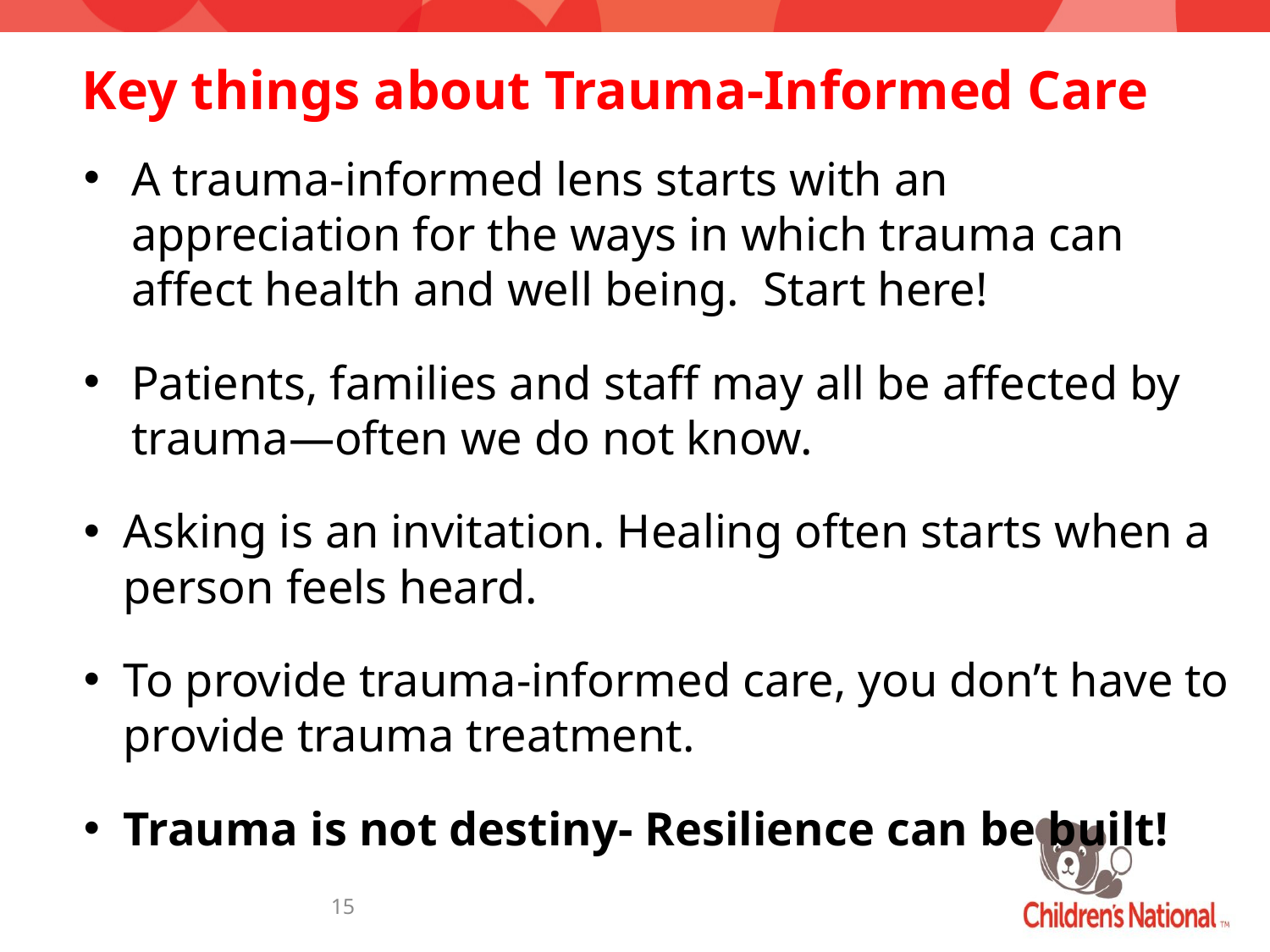

# Key things about Trauma-Informed Care
A trauma-informed lens starts with an appreciation for the ways in which trauma can affect health and well being. Start here!
Patients, families and staff may all be affected by trauma—often we do not know.
Asking is an invitation. Healing often starts when a person feels heard.
To provide trauma-informed care, you don’t have to provide trauma treatment.
Trauma is not destiny- Resilience can be built!
15

## Slide 16
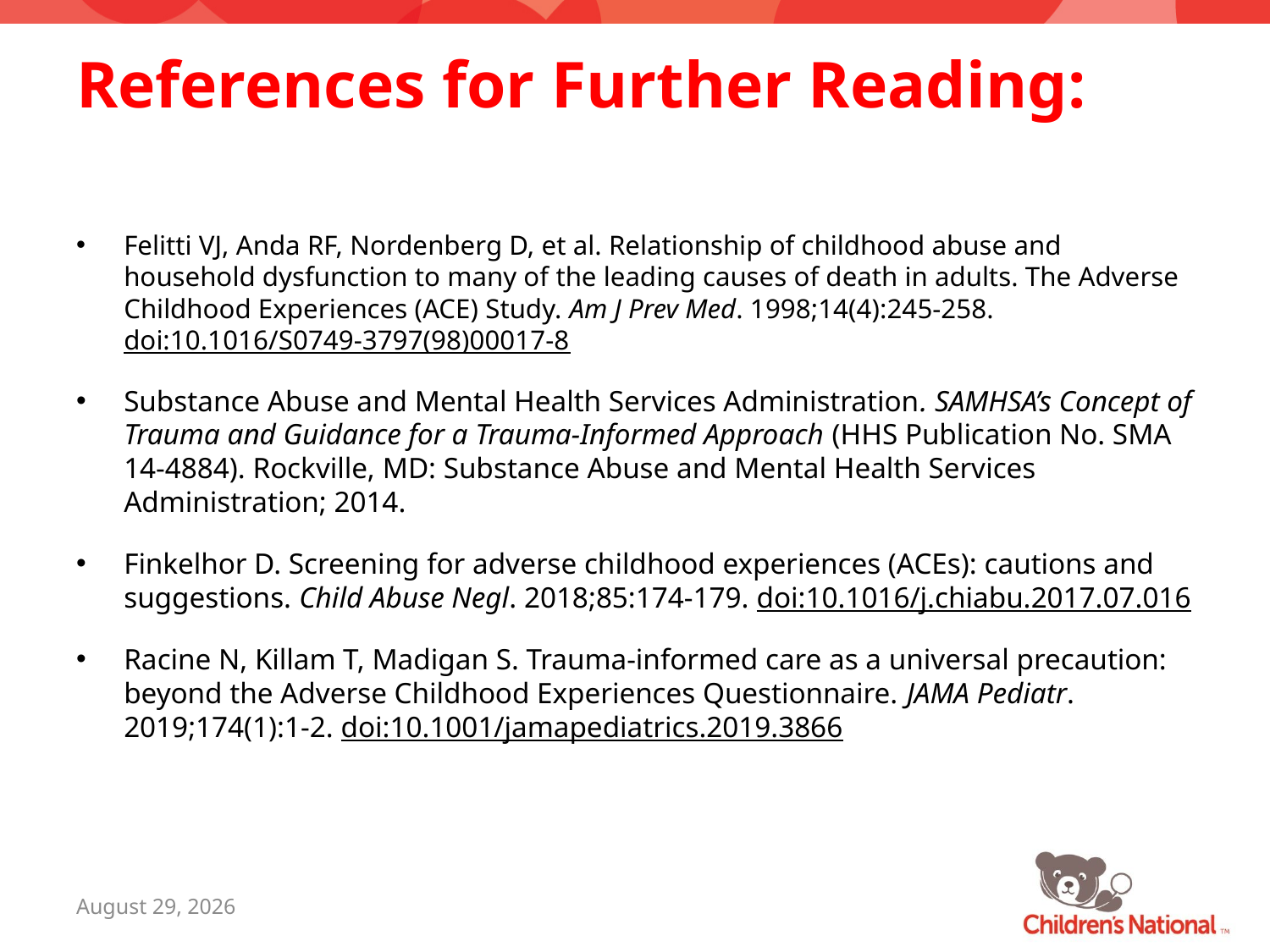

# References for Further Reading:
Felitti VJ, Anda RF, Nordenberg D, et al. Relationship of childhood abuse and household dysfunction to many of the leading causes of death in adults. The Adverse Childhood Experiences (ACE) Study. Am J Prev Med. 1998;14(4):245-258. doi:10.1016/S0749-3797(98)00017-8
Substance Abuse and Mental Health Services Administration. SAMHSA’s Concept of Trauma and Guidance for a Trauma-Informed Approach (HHS Publication No. SMA 14-4884). Rockville, MD: Substance Abuse and Mental Health Services Administration; 2014.
Finkelhor D. Screening for adverse childhood experiences (ACEs): cautions and suggestions. Child Abuse Negl. 2018;85:174-179. doi:10.1016/j.chiabu.2017.07.016
Racine N, Killam T, Madigan S. Trauma-informed care as a universal precaution: beyond the Adverse Childhood Experiences Questionnaire. JAMA Pediatr. 2019;174(1):1-2. doi:10.1001/jamapediatrics.2019.3866
June 4, 2020
